# Dynamically autofocused 3D pulsed laser micromachining enables advanced 3D bioelectronics

**DOI:** 10.1126/sciadv.adz4084

**Published:** 2025-11-07

**Authors:** Massimo Mariello, Joseph G. Troughton, Yixuan Leng, Ziming Wang, Nuzli Karam, Lawrence Coles, José González-Martínez, Kawsar Ali, Daniel J. Rogers, Madeline Lancaster, Christopher M. Proctor

**Affiliations:** ^1^ Institute of Biomedical Engineering, Department of Engineering Science, University of Oxford, OX3 7DQ Oxford, UK.; ^2^ Department of Electrical Engineering, University of Cambridge, Trumpington Street, CB2 1PZ Cambridge, UK.; ^3^ Medical Research Council (MRC) Laboratory of Molecular Biology, Cambridge Biomedical Campus, Francis Crick Avenue, Cambridge CB2 0QH, UK.; ^4^ Department of Engineering Science, University of Oxford, OX1 3PJ Oxford, UK.

## Abstract

Advancing next-generation bioelectronic interfaces requires devices that are soft, miniaturized, and seamlessly integrated with biological tissues. However, conventional fabrication methods, primarily based on UV photolithography, struggle to meet these needs, relying on hazardous chemicals, labor-intensive processes, and planar, layer-by-layer construction. To overcome these limits, we introduce dynamically autofocused 3D pulsed laser micromachining (d-3DPLM) using a nanosecond pulsed near-infrared laser. This approach enables rapid, cost-effective structuring of diverse materials, including thin films, metal foils, and bulk metal blocks, supporting monolithic, multilayer bioelectronics with complex 3D architectures such as microneedles and deployable elements. d-3DPLM achieves high-resolution ablation and patterning for conformable and functional device geometries. Demonstrated applications include electro-haptic patches, in vitro multielectrode diagnostic arrays, and wireless contact lenses for red light therapy. By broadening the design and manufacturing landscape for bioelectronic systems, this versatile method paves the way for improved performance, unique functionality, and enhanced integration with living tissue.

## Teaser

Dynamic laser micromachining enables versatile bioelectronic devices with 3D features and advanced functionality.

## INTRODUCTION

Recent advances in the fabrication of functional bioelectronic microsystems have highlighted specific requirements. Next-generation wearable or implantable devices should ideally be soft, miniaturized, and seamlessly integrated with the surrounding tissue (skin or internal organs). The main techniques now used for fabricating such devices are mostly based on ultraviolet (UV) photolithography, soft lithography, transfer printing, and 3D additive printing of functional inks (*1*–*4*). All these methods are effective but have drawbacks related to low cost-effectiveness, time-consuming operation, and impractical mass production (*5*). High-speed scalable manufacturing is a requirement for the widespread deployment of new bioelectronic devices, with sophisticated designs or multiple functionalities, in medical or IoT applications (*6*). Therefore, there is an increasing demand of novel technologies and fabrication methodologies that can meet the following needs: (i) accurate dimensional control in terms of spatial (thickness and lateral) resolution; (ii) compatibility with different categories of materials, from metals and rigid substrates to polymers and soft/flexible materials; (iii) fast and practical operation to produce multilayered, multimaterial device architectures for different application purposes; (iv) capability of introducing three-dimensional (3D) features into the design of bioelectronic systems. The latter has gained much interest in the recent years from researchers and industry worldwide, especially in the field of implantable electrical interfaces; although bioelectronic devices with 2D and flat electrode structures have been used extensively (*7*), the lack of uniform conformability and adaptability to the nonplanar and soft biological tissues remains a challenge (*8*). 3D bioelectrodes or dielectric patterns can reach deeper regions of tissues, with improved adhesion and enhanced performances (*8*–*11*).

Pulsed laser micromachining (PLM) has been demonstrated as a convenient technique for achieving high-resolution surface patterning (*12*). Previous works have shown that the advantage of short pulsed (<10 ns) and ultrashort pulsed (<1 ps) lasers consist of limiting thermal diffusion and heat spreading in the laser-ablated zone (*13*–*15*). As a recent example, Yang *et al.* (*6*) introduced a high-speed, scanned, picosecond pulsed laser ablation approach (*16*, *17*) combined with physical lamination and/or transfer printing, as a versatile method for fabricating multilayered eco/bioresorbable electronic systems, including complementary metal-oxide semiconductor devices. (Ultra)short pulsed lasers, operating across UV to near-infrared (NIR) wavelengths, offer the capability to finely tune ablation rates and surface characteristics by leveraging the material-specific absorption and reflectivity properties specific to these wavelengths (*18*, *19*). The ablation process is driven by complex mechanisms, including rapid vaporization, melt cavitation, and direct sublimation or plasma ionization (*20*–*24*). These lasers are widely used in precision manufacturing, such as the creation of interconnect traces for flexible circuit boards (*25*) and the development of specific thin-film sensors (*26*–*28*), showcasing their effectiveness in achieving resolutions exceeding 20 μm laterally and around 1 μm vertically. However, current implementations primarily focus on single-layer, or multilayer, functional systems, with recent attempts for high-resolution processing of semiconductor substrates or bioresorbable materials (*6*). In particular, the potential of short PLM and micropatterning for advancing 3D bioelectronics has not been fully explored yet. Their high spatial resolution and material-specific selectivity can enable the precise fabrication of intricate 3D structures tailored for bioelectronic applications. So far, 3D PLM has been used to etch complex topographies on flexible and soft flat substrates, facilitating the development of electrodes and scaffolds that can conform, postfabrication, to the curved and dynamic 3D surfaces of biological tissues (*28*, *29*). Laser-assisted micropatterning has been used to integrate multimaterial systems, including metal interconnects and dielectric layers, into 3D geometries (*30*–*32*). For instance, hybrid approaches combining laser ablation with additive manufacturing have been explored to stack functional layers into multidimensional constructs, enabling the fabrication of bioresorbable devices with complex architectures for temporary implants (*6*). In addition, recent studies (*33*–*36*) have demonstrated the feasibility of using laser ablation to sculpt microgrooves, wells, and pyramidal arrays, which improve the interface with neural tissues by enhancing surface area and charge transfer capabilities.

In all the previous examples, PLM represents a step to create 3D features which are then handled and reshaped to conform to the tissue surface. As yet unexplored in the field of bioelectronics, we thus propose a dynamically autofocused 3D PLM (d-3DPLM), which consists of micromachining advanced devices by ablation and micropatterning, simultaneously conferring them the desired conformable shape, which is not achievable with standard photolithography. The name of the method refers to the dynamic application of the laser autofocus function, i.e., continuously adjusted in real time during ablation and along the predefined 3D envelope surface, to compensate for positional and focal deviations. This approach contrasts with conventional “static” autofocus strategies, in which the focal plane is set before processing and remains fixed throughout. This dynamic implementation is therefore a defining characteristic that differentiates d-3DPLM from standard laser micromachining.

To our knowledge, this innovative approach represents the first use of such a system for fabricating advanced 3D bioelectronic devices. It can change the material-processing paradigms for next-generation microdevices, enabling the direct and monolithic fabrication of 3D bioelectronic circuits onto any sort of substrate or surface, including curved and deformable ones, offering a universal pathway to scalable and customizable device production. Our d-3DPLM process allows direct, maskless fabrication of systems that would be extremely challenging, if not impossible, to realize with planar techniques, such as standard cleanroom lithography.

In this work, we validate and demonstrate the utility of the d-3DPLM approach, showcasing a series of device-level demonstrations involving advanced sensing/stimulating technologies enabled by d-3DPLM, using a NIR nanosecond (ns) pulsed laser system. These include electro-haptic wearable patches with 3D features for enhanced tactile stimulation, tunable-height multielectrode arrays (MEAs) for in vitro diagnostics of biopotential signals of human cerebral organoids, and wireless contact lenses for delivering red light therapy with seamless 3D biointegration. While previous studies have largely relied on UV or femtosecond (fs) lasers, we show that optimized NIR ns processing achieves high precision with scalability and accessibility. This expands the toolbox for laser micromachining in bioelectronics and enriches understanding of NIR ns pulse interactions with diverse materials. The three representative demonstrators highlight the versatility of this platform across materials, architectures, and geometries, serving as proof-of-concept examples and laying the foundation for future 3D bioelectronic designs tailored to complex biological interfaces. These innovations illustrate the wide-ranging potential applications of such technologies across multiple organ systems, emphasizing the versatility and impact of this fabrication approach.

## RESULTS

### NIR d-3DPLM: Innovative approach for 3D bioelectronics

The ns pulsed NIR laser micromachining method described here enables (i) high-resolution cutting and ablation of materials into fine patterns, (ii) selective removal of material from specified regions, (iii) creation of 3D features through sequential ablation and milling steps with different focal planes, and (iv) creation of 3D shapes through a single ablation step along a preestablished 3D surface, all with minimal thermal impact on surrounding structures. A wide range of materials, including polymers, metals, and ceramics, can be processed using these techniques, making them ideal for fabricating advanced 3D bioelectronics. The use of ns pulses at high pulse frequencies minimizes heat buildup, ensuring that the integrity of the materials is preserved while achieving high resolution in both lateral and vertical dimensions.

The choice of laser wavelength also plays a critical role in micromachining, as it governs absorption, resolution, and thermal effects in the target material. Note S1 reports an analysis of trade-offs associated with different laser wavelengths for micromachining bioelectronic materials. UV lasers enable high-precision “cold” ablation in polymers but are limited by lower power and complex optics, while visible wavelengths provide intermediate absorption and ease of handling. NIR lasers, such as the 1064-nm source used in this study, generally offer deeper penetration and higher power but at the cost of larger heat-affected zones and reduced resolution in transparent polymers. We used the NIR laser integrated in the Keyence system due to its accessibility and its suitable trade-off between process speed and sufficient feature fidelity, especially when combined with pulsed operation and optimized scan strategies to mitigate heat accumulation.

**Fig. 1.**
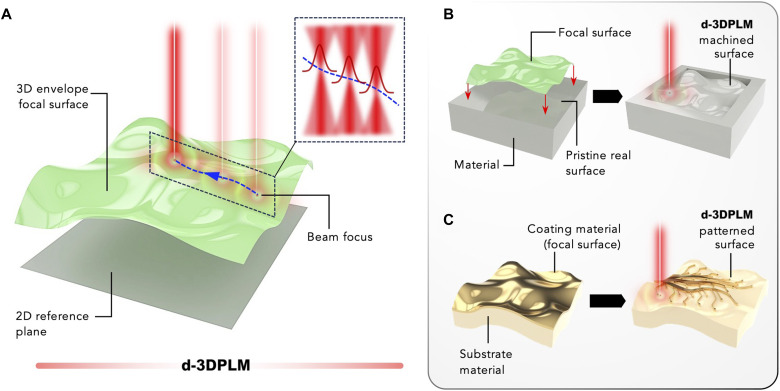
Dynamically autofocused 3D pulsed laser micromachining. (**A**) The figure illustrates the principle of the d-3DPLM process where the focal point of the short-pulse laser beam conformally follows the contours of a 3D envelope surface, with respect to a 2D reference plane. The inset shows the Gaussian distribution of the laser beam. The d-3DPLM process can be used for machining the pristine surface of a material (**B**) or patterning the surface of a coating material (**C**), according to a preset 3D envelope focal surface. In (C) the focal surface ideally coincides with the coating surface.

The proposed technique offers a streamlined alternative to traditional cleanroom-based micropatterning approaches, avoiding the need for complex photolithography or wet processing steps, as well as to more recent micromachining methods which lack 3D adaptability. Figure 1 (A to C) illustrates the generic procedure for a d-3DPLM process, where the focal point of the laser beam conformally follows the contours of a selected 3D surface, which, in the simplest case, can be a plane perpendicular to the beam, or it can consist of complex, more sophisticated open 3D geometries.

The key features of the proposed approach are summarized in table S7, with comparison to current standard techniques to achieve 3D bioelectronic devices and systems. The d-3DPLM process is entirely dry, not involving any photolithography or wet etching steps; it reduces thermally induced damage of the ablated materials due to the short laser pulses; it enables a high-fidelity and high-spatial resolution micromachining of many materials; it allows monolithic and multilayer integration of 3D features into advanced bioelectronic circuits.

Figure S1 illustrates different applications of the proposed d-3DPLM process for the development of advanced 3D bioelectronic systems. Specific embodiments include electro-haptic wearable patches for enhanced tactile stimulation, wireless contact lenses for red light therapy and seamless 3D biointegration, and tunable-height MEAs for in vitro diagnostics of biopotential signals of human neural organoids.

### Characterization of the NIR ns pulsed laser 2D ablation

To characterize the d-3DPLM process, we started with testing the capabilities of the ns pulsed laser system in terms of ablation resolutions. These operations are enabled by directing the focused laser beam, with a Gaussian intensity profile (fig. S2), to remove material through controlled melting, vaporization, or sublimation (see note S2 for a detailed description of the analytical/empirical model used to identify the NIR laser beam size and its radial optical power density). The ablation depth scales with pulse energy and is governed by both laser parameters (wavelength, duration, fluence, and repetition rate) and material properties (thermal conductivity, heat capacity, density, and vaporization enthalpy). Empirically, the ablation depth follows a power-law dependence on fluence, with coefficients varying by material and exposure duration, while cumulative pulsing introduces additional effects such as redeposition and heat accumulation. Radial ablation profiles follow the Gaussian intensity distribution, consistent with experimental single-spot depth measurements. Overall, the model captures the key regimes of ns pulsed ablation while acknowledging limitations in ultrathin films where some thermal properties (e.g., heat capacity) may deviate from bulk.

The main contribution to the ablation is the material sublimation due to a rapid heat generation by the laser beam in the bulk, rising the temperature over the exposed area (radially) and in depth. In the ns regime, thermal diffusion lengths (100 nm to 10 μm) are larger than for picosecond/fs pulses (see note S3) but remain comparable to the lateral machining resolution (~10 μm), explaining the presence of melt zones and recast layers observed experimentally (see note S4 for a detailed discussion of physical mechanisms occurring during laser ablation). Finite element modeling (FEM) analysis (considering a Gaussian source term and Beer-Lambert absorption) confirms that pulse duration strongly affects the temperature profile within the ablated volume: for instance, the surface of a stainless steel block hit by one single NIR ns laser pulse reaches the maximum temperature, on the beam axis, of 49° and 1448°C for 1-ns and 1-μs duration, respectively (note S3 and fig. S3). Longer pulses increase heat accumulation and broaden the heat-affected zone, while ultrafast pulses minimize thermal damage through athermal ablation. The key laser parameters for ablation include average power, scanning speed, pulse frequency, and number of repetitions. The laser fluence (related to the pulse frequency and the pulse energy; fig. S2B) determines to what extent the material undergoes melting and/or vaporization (*37*). The nature of the material also strongly affects its response to the laser pulse, as confirmed by single point damage threshold tests performed on stainless steel blocks and copper foils (fig. S10): Ablation spot areas of the same order of magnitude can be obtained on both materials but with different (continuous) ablation times and fluence values.

All the aforementioned parameters, as well as the fill interval (the spacing between adjacent scan lines) or the hatching pattern, determine the process yield, i.e., ablation depth and edge quality (divergence between projected and effective width) (fig. S7A). In particular, linear (slant) and cross-hatched hatching patterns ensure more uniform ablation morphology compared to spiral hatching (fig. S6). Figure S7B provides a comprehensive overview of the influence of the pulse frequency, power, and scanning speed onto the ablation depth for stainless steel, with the profile measurements reported in figs. S8 and S9. The thickness reduction exhibits a monotonic increase with higher average power, while it decreases with increasing scanning speed or frequency. Notably, when the average power remains constant, a decrease in frequency corresponds to an increase in peak power. The minimum reduction in thickness is within ~100 nm to 1 μm for the NIR ns pulsed laser system and for stainless steel.

**Fig. 2.**
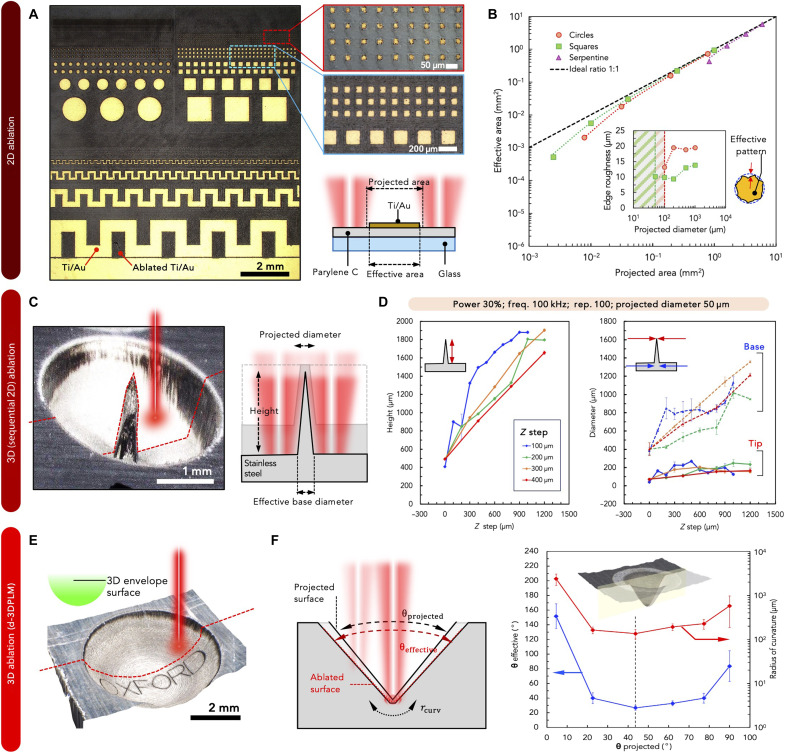
Characterization of the NIR laser ablation process and of d-3DPLM. Representative characterization cases for 2D laser ablation on thin films (**A** and **B**), sequential 2D ablation (**C** and **D**), and 3D ablation (d-3DPLM, **E** and **F**) on stainless steel. (A) Optical image of a thin-film parylene C/Ti/Au patterned by NIR laser ablation with different shapes (circles, squares, and serpentines). The scheme shows the ablation process, indicating the projected and effective width of the target pattern. (B) Plot of the effective area of different patterns (circles, squares, and serpentines) as the function of the projected area. The dashed line in the main plot corresponds to the ideal 1:1 ratio. The inset shows the edge roughness of the squares and circles versus the projected diameter. The dashed areas represent the values of projected diameter for which it is not anymore possible to detect a pattern (in-plane resolution). The scheme shows how the edge roughness is extracted. (C) Optical 3D image of a MN created by NIR sequential laser ablation (varying the *z* depth) on stainless steel. The scheme shows the MN height, the projected and effective base diameter. (D) Representative characterization of the MNs obtained by ablation at set laser parameters (power, 30%; pulse frequency, 100 kHz; repetitions, 100) and a projected diameter of 50 μm: The plots report the height, the effective base/tip diameters versus the *z* steps, for different *z* step sizes (100, 200, 300, and 400 μm). (E) Example of a hemispherical shape created by d-3DPLM on stainless steel, using a 3D hemispherical envelope surface. (F) Characterization of the d-3DPLM angular resolution. The scheme shows laser ablation of a convex conical shape, indicating the effective radius of curvature and the effective and projected full-apex angle ( $\theta_{\text{projected}},\theta_{\text{effective}}$ ). The plot reports the effective angle and the radius of curvature versus the projected angle.

Besides stainless steel blocks (used to create tunable-height 3D MEAs), similar analyses were performed for the other material systems selected for this study, which comprise metal thin films [Ti (10 nm)/Au (100 nm)] deposited onto polymeric thin substrates (parylene C, 5 μm) (used to fabricated wearable electro-haptic patches) and metal laminate foils (Cu, 116 μm) on soft elastomers [poly(dimethylsiloxane) (PDMS)] (adopted for the fabrication of wireless contact lenses). These choices are representatives of the main combinations of materials (thin films, foils, and bulky blocks) generally used for advanced bioelectronics. Each material has a specific and unique response to the interaction with the laser beam, which needs to be preliminarily assessed (fig. S10D). A comprehensive description of laser ablation on parylene C/Ti/Au and Cu laminate foils on PDMS is reported in note S5, including the analysis of the edge quality based on different sample shapes (Fig. 2, A and B).

The theoretical lateral resolution of the ns pulsed laser system can be assumed as the full-width at half maximum (~25 μm), as it follows from the use of a 30-μm-diameter, Gaussian laser spot (see note 2 and fig. S2A). The actual spatial resolution always depends on the material response to the laser ablation, which is affected by its thermal properties. In particular, on the same plane of ablation (2D ablation), overlapping scans can provide additional capabilities in resolution: To demonstrate this, we created ribbon shapes (with 5-mm lengths) on stainless steel blocks (fig. S22A), by separating two adjacent laser scans by a preset projected width ( $w_{P}$ ) (see fig. S20A). We selected 14 ribbons with $w_{P}$ in the range 50 to 500 μm. Different scenarios can be identified (fig. S20B) and observed experimentally (fig. S21). Reducing $w_{P}$ affects the shape of the cross-sectional area of the ribbons: When $w_{P}$ is larger than the beam diameter, the shape is trapezoidal, with gradually tapered edges due to the Gaussian intensity distribution of the beam, whereas it becomes triangular when the $w_{P}$ is comparable to or smaller than the beam size, and the ribbon’s height, after an initial increase due to material recasting, decreases with further reducing $w_{P}$ (figs. S22 and S23). With this approach the lateral resolution is defined as the projected width below which the effective width deviates from an ideal 1:1 ratio (right-side plots in fig. S24): Specifically, minimal feature sizes of 23 μm could be achieved on steel. It should be noted that this also depends on the laser parameters: Lower powers or higher scanning speeds lead to a gentler ablation and a smaller ribbon’s effective width (figs. S23 and S24). The same experiments were conducted on the other material systems (see figs. S15 to S19): After sweeping the laser parameters within representative ranges, we found an actual lateral resolution of 10 μm on Ti(10 nm)/Au(100 nm)/parylene C and 22 μm on Cu (116 μm)/PDMS.

### Characterization of the NIR ns pulsed laser 3D ablation and the d-3DPLM process

The formation of a ribbon with a trapezoidal or triangular cross section through laser ablation depends on the spatial arrangement of parallel laser scans within the same ablation layer (2D ablation) as well as the vertical separation between successive layers (sequential 2D ablation). Similar considerations hold when considering microneedle (MN; axial-symmetric) shapes created by sequential 2D ablation starting from a projected cylindrical shape (Fig. 2C and fig. S27). The theoretical spatial (out-of-plane) resolution of the NIR ns pulsed laser is given by the Rayleigh range of the laser beam ( $z_{R}$ ), which is ~29.5 μm (thus with a depth of focus of ~59 μm; fig. S2A): This affects the consistency of ablation across layers (fig. S20B). If the separation between layers is comparable to or smaller than $z_{R}$ , uniform material removal occurs, reinforcing the ribbon or cylinder’s quasi-rectangular shape. However, if the layer spacing exceeds $z_{R}$ , defocusing effects reduce ablation efficiency, leading to irregularities or incomplete layer melting. If the laser parameters are strong enough to create an ablation depth larger then $z_{R}$ with a single layer, then the ablation narrows the cross-sectional shape of the ribbon/cylinder, yielding a narrower trapezoid or a shorter triangle. In some cases, material recast at the edges can lead to the formation of a raised ribbon/MN, where redeposited material accumulates along the ablated trenches, resulting in a final structure that is higher than the nominal ablation depth. In light of the above, we found a vertical resolution of ~10 μm for stainless steel and ~5 μm for Cu foils.

We characterized the height, effective base, and tip diameters of MNs formed out of stainless steel blocks, systematically varying the laser parameters in-plane (fig. S28) as well as the layer spacing along the *z* direction (Fig. 2D and figs. S29 and S30). This is the simplest version of the d-3DPLM method, where a 2D pattern is used as envelope surface for sequential 2D ablation. The final MN height, as well as its progressive evolution during the ablation process, is affected by the number of and distance between ablation layers. We characterized MNs with projected diameters of 50 to 1000 μm, and we selected different *z* steps (100 to 400 μm). Notably, the height follows a quasi-linear trend when the *z* step is comparable to the ablation depth induced by the first ablation layer: For the selected laser parameters shown in fig. S29, this occurred with *z* step equal to 300 μm. Reducing the projected width (20 to 50 μm) requires reduction of the *z* steps as well, to achieve finer MN form factors (fig. S30). The effective base diameter is influenced by the lateral beam overlap between adjacent scans and increases with increasing the number of ablation layers (Fig. 2D). The increase in the tip diameter with increasing ablation layers is less pronounced, and it was minimized by optimizing the focal position and reducing the scan step size in the final ablation layers to achieve finer resolution (Fig. 2D): Therefore, tip diameters of ~20 μm were achieved, with projected diameters of 30 μm and *z* steps of 50 μm.

In the context of thins films, the spatial vertical resolution relates to the ability to reduce the thickness of the upper layer with minimum damage to the underlying materials. We assessed this parameter on our Au/parylene C material system, for which a minimum depth of damage of ~35 nm was achieved to the parylene substrate (fig. S16).

Next, we characterized the ns laser capabilities of ablating along a 3D envelope surface (Fig. 2E and fig. S25, A to C). For the sake of demonstration, we chose three different examples of surfaces, i.e., a convex hemisphere, a cone, and a concave hemisphere. After inputting the envelope surface into the control software of the laser system, it was possible to engrave those surfaces into a stainless steel block through sequential ablation layers. The focal point of the laser beam follows the contours of the selected surface: Ablation occurs at the intersection curve between the 3D envelope surface and the real surface of the material, within the Rayleigh range. Thus, lowering the *z* level of each ablation layer shifted the 3D envelope surface downward, progressively engraving the material and creating the target surface profile (fig. S25, D to F).

The capability of ensuring the ablation fidelity between the selected 3D envelope surface (projected surface) and the effective surface engraved into the material depends on the angular resolution of the d-3DPLM process. This is crucial to understand how precisely the laser system can replicate sharp, angular features at different inclinations, ensuring high-fidelity 3D microfabrication. To evaluate this parameter, a set of conical structure was fabricated using the ns pulsed NIR laser system, with different sets of laser parameters. Each structure was designed with a distinct full-apex angle, ranging from 90° down to approximately 4°, and was subsequently characterized by measuring both the actual apex angle and the radius of curvature at the cone tip using 3D optical profilometry (fig. S26, A and B, and note S9).

As the designed apex angle was reduced from 90° to ~42°, the ablated geometries maintained good agreement with the projected shapes (Fig. 2F and fig. S26, C and D). The measured apex angles were slightly lower than the intended values, likely due to inherent tolerances in laser-material interaction, minor energy diffusion, and cumulative effects of pulse overlap. In this regime, the effective ablation closely followed the expected conical geometry, indicating that the laser system’s vertical (*z* axis) and lateral (*xy* plane) resolutions were sufficient to resolve and construct angled profiles within this angular range. However, as the apex angle was further reduced below ~42°, notable deviations emerged. Rather than becoming sharper, the engraved cones began to exhibit increasing apex angles and larger tip radii, suggesting a breakdown in the fidelity of the shape transfer. This degradation is attributed to limitations imposed by the optical focusing conditions, specifically the Rayleigh range of the laser focus. As the cone angle narrows, a larger portion of the target geometry falls outside the laser’s effective focal volume, and the shallow Rayleigh range reduces the depth over which effective ablation can occur without defocusing. Furthermore, the interplay between vertical and lateral resolution becomes increasingly unfavorable at sharp angles. While the lateral resolution is defined by the beam waist, the vertical resolution is governed by the axial extent of the focal spot and the material’s ablation threshold. At narrow apex angles, the focal spot overlaps insufficiently with the inclined surfaces, reducing energy density at the targeted depth and leading to incomplete material removal. These limitations result in a loss of angular definition and decreased tip curvature, highlighting the resolution trade-offs inherent to volumetric laser microfabrication at extreme geometries. Therefore, a 3D angular resolution of ~35° to 42° was obtained for stainless steel and Cu foils. Reducing the thickness of the substrate material limits the possibility of evaluating the angular resolution of laser ablation: This holds true for coated thin films, where the d-3DPLM capability lies in the ability to precisely pattern the film directly on the surface of a substrate that already has a predefined 3D shape. In that case, the same angular resolution should be taken into account when attempting to pattern tilted surfaces or sharp angles.

Last, the influence of surface curvature on micromachining lateral resolution is markedly different when comparing conventional 2D ablation with the d-3DPLM approach. To this purpose, we tested the lateral resolution on stainless steel specimens with different radii of curvature (fig. S31). In a standard process, increasing curvature leads to notable resolution loss, since only the central region remains in focus (effective focused-ablation area), while peripheral areas suffer from defocusing. By contrast, in d-3DPLM, the focal point is continuously maintained along the curved surface, so resolution (minimal feature size) is maintained and only slightly affected (~8% increase going from 20- to 2.5-mm radius), mainly by minor defocusing or angular incidence effects. This highlights how d-3DPLM enables feature definition on nonplanar substrates with an effective resolution comparable to flat-surface ablation, offering a direct route for extending micromachining to complex 3D geometries.

### Wearable electro-haptic patches with millineedle electrodes for tactile stimulation

Electro-haptic devices play a crucial role in health care by providing controlled electrical stimulation to restore or enhance sensory perception (*38*). They are used in prosthetics to deliver tactile feedback, improving motor control for amputees, and in neurorehabilitation to aid recovery from nerve damage or stroke (*39*, *40*). These devices also enable noninvasive pain management and therapeutic stimulation for conditions like neuropathy and Parkinson’s disease (*41*). By integrating advanced materials and 3D architectures, electro-haptic systems can offer more precise, customizable, and comfortable stimulation, enhancing patient outcomes and expanding applications in assistive technology, rehabilitation, and human-machine interaction (*42*, *43*).

**Fig. 3.**
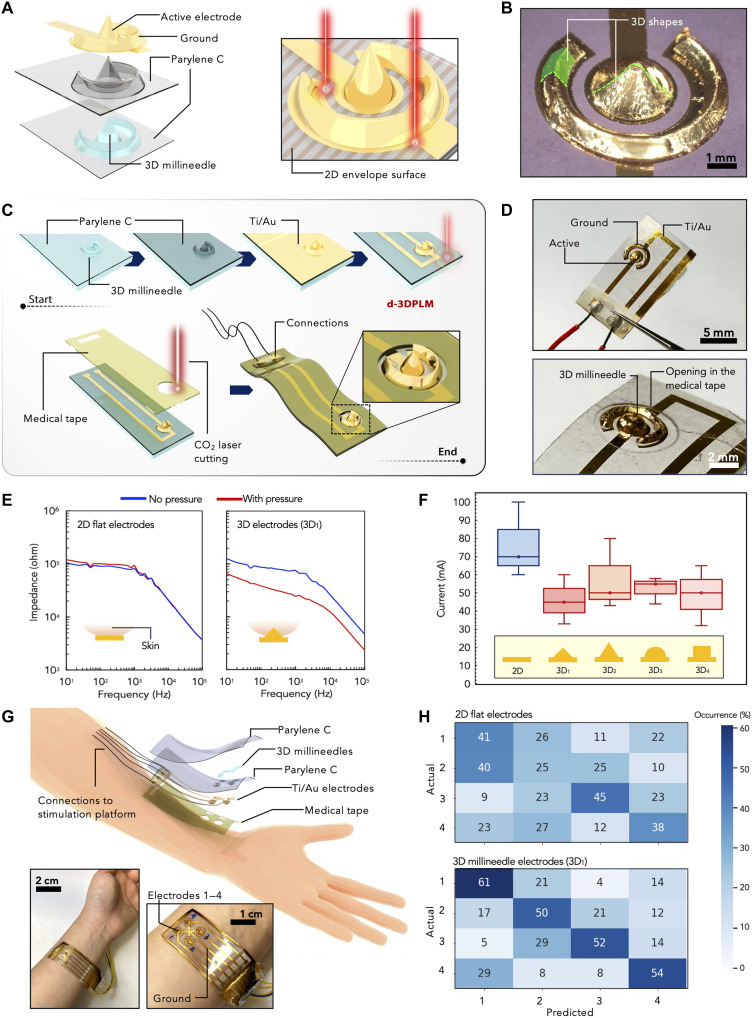
Electro-haptic patches with millineedle electrodes, patterned through d-3DPLM, for tactile stimulation. (**A**) Exploded view of a 3D millineedle electrode based on 3D printed structures coated with parylene C and metalized with Ti/Au thin films. These are then patterned with the laser micromachining process. (**B**) Photos of the 3D millineedle electrodes after the d-3DPLM process. (**C**) Illustration of the main steps of the fabrication process of a flexible electro-haptic patch with a single electrode, highlighting the use of the laser in the d-3DPLM process. (**D**) Photo of a flexible electro-haptic patch with a single electrode. (**E**) Contact impedance spectroscopy of the electro-haptic electrodes on the skin. (**F**) Threshold current for 2D and 3D electrodes in electro-haptic devices tested on the finger and palm of the selected volunteers. (**G**) Illustration of a flexible multielectrode electro-haptic patch worn on the wrist. The exploded schematic shows the different layers of the device including four pair of active-ground electrodes. (**H**) Confusion matrix for the blind tests conducted with the four-electrode patch worn on the volunteers’ wrist.

However, the introduction of 3D features, such as sharp 3D electrodes, is not compatible with standard photolithography or layer-by-layer wet techniques. Despite few attempts to achieve 3D haptics (*44*, *45*), current state-of-the-art devices are still based on flat electrode–based tactile interfaces, which often suffer from inconsistent stimulation due to poor electrode-skin contact. Our d-3DPLM process provides a useful approach to integrating customizable 3D features into flexible devices, enabling rapid prototyping of electro-haptics with small footprints and compact form factors. This combines the advantages of thin films, i.e., flexibility, conformality, lightweight design, and high biomechanical matching with the host tissue, with enhanced electrotactile stimulation (improved charge injection, reduced impedance, and deeper, more localized effectiveness) induced by the 3D elements. To that end, we propose thin-film, wearable electro-haptic patches with 3D millineedle electrodes, whose fabrication was made possible through the described laser micromachining process. The process flow is based on sandwiching a 3D-printed millineedle structure between two layers of parylene C (5 μm) and then depositing a Ti/Au conductive film onto the parylene superstrate (Fig. 3(A) and fig. S32). Through the d-3DPLM process performed along a 2D envelope surface, it was possible to pattern the Ti/Au layer into functional electrodes (active and ground electrodes), defining their geometry, selectively removing excess material, and structuring the conductive traces. 2D and 3D electrodes were patterned through this approach (Fig. 3B): Different 3D shapes were fabricated, i.e., cones, domes, and cylinders (fig. S33). A medical-grade adhesive encapsulation was patterned using a CO_2_ laser and transferred on the patterned device, ensuring conformal attachment to curved body surfaces while maintaining mechanical stability (Fig. 3C). A single device includes an active 3D millineedle electrode and a 3D ring as ground electrode (Fig. 3D).

To evaluate device performance, we conducted electrophysiological and electrical characterization studies. Electrochemical impedance spectroscopy (EIS) (Fig. 3E and fig. S34) revealed that the contact impedance between the electrodes and the skin (finger) was lowered by the 3D features, indicating improved electrical coupling and enhanced charge transfer, with respect to the 2D counterparts. Without any applied pressure, 2D and 3D electrodes exhibit a contact impedance of ~80 kilohm (at 1 kHz): Applying a moderate level of pressure [~50 to 80 kPa (*46*); see fig. S35 for details on the pressure quantification], the impedance of 2D electrodes remains unchanged, whereas the 3D electrodes exhibit a lower value of ~25 kilohm (at 1 kHz). This can be ascribed to the larger contact surface appearing when pressure is applied on the finger, leading to a greater skin deformation and conformal contact with the 3D millineedle electrode. Threshold current measurements (Fig. 3F) compared the stimulation efficacy of 2D planar electrodes versus 3D millineedles on the finger skin (without additional pressure), to determine the optimal design for efficient neural activation. The threshold was defined as the minimum current level inducing a detectable sensation in the volunteers. The average minimum threshold current level across four volunteers was higher for 2D electrodes (~70 mA), compared to 40 to 50 mA for 3D electrodes. Only modest differences in threshold current were observed between 3D electrodes of varying shapes including rounded dome, pillar, and pointed dome. We postulate the lower threshold current with 3D electrodes compared to 2D electrodes results from the larger contact surface with the skin and a deeper penetration of the stimulation current. FEM simulations in fig. S36 (see note S6) are consistent with the enhanced stimulation capability of the 3D electrodes compared to the 2D counterparts: Because of the tip effects, the distribution of electric field and current density is sharper for the 3D electrodes, leading to a more concentrated region on top of the 3D millineedle and therefore a more effective penetration in the dermis. To sum up, the performance differences between 2D and 3D electrodes become most evident under applied pressure, yet the true advantage of the 3D geometry lies beyond impedance reduction. Specifically, the 3D electrodes achieve lower current thresholds for perceptual activation and more efficient current delivery, related to the spatial relationship between electrodes and cutaneous nerve endings, as shown in threshold measurements and current density simulations. While the FEM model does not directly predict firing thresholds, the field gradients and isopotential contours support the experimentally observed reduction in perceptual thresholds.

This improvement arises from the enhanced conformability of the 3D geometry, its ability to maintain more stable skin contact under nonideal conditions, and its capacity to focus the electric field, all of which translate into more current-efficient stimulation and reduced power demand.

Electrode area further influences this relationship: Larger 2D electrodes spread current across a wider region, lowering density and raising the required amplitude, whereas smaller 2D electrodes increase density but at the expense of higher impedance and reduced charge injection. The 3D structures mitigate this trade-off, sustaining localized high current density while preserving stable contact. For fairness, electrode dimensions were kept consistent between 2D and 3D designs, ensuring that geometry, specifically height, curvature, and conformability, was the primary variable. Overall, the lower thresholds observed with 3D electrodes can be attributed to their enhanced conformal contact and field localization, which enable more efficient and reproducible stimulation across nonflat surfaces. These functional benefits are directly tied to our fabrication method: The d-3DPLM process enables direct-write patterning of flexible substrates integrated with 3D features, allowing the realization of millimeter-scale electrodes with spatial precision in a few steps. This capability, which is difficult to achieve with conventional lithography or transfer printing, highlights not only the advantages of the 3D geometry itself but also the unique role of the laser-based approach in enabling rapid, custom, and versatile prototyping of such devices.

As a practical demonstration, a wearable prototype with four active-ground electrode pairs was then applied to the wrist and connected to an external stimulation platform (Fig. 3G and fig. S37). The device exhibited good flexibility (Young’s modulus of ~3 GPa; fig. S38), with confirmed electrode impedance values (fig. S39A). Blind tests were conducted while randomly activating two pairs of electrodes and asking the volunteers to report which electrodes they believed to be stimulating. The confusion matrix plot in Fig. 3H (and fig. S39B) correlates the user feedback with the predicted/actual activated electrode, providing insights into the system’s efficiency. We defined as positive feedback the scenario where the volunteer guessed correctly the numbers of stimulating electrodes. The findings demonstrate that the introduction of the 3D millineedle structures favors a higher occurrence of positive feedback compare to 2D electrodes, with occurrence probability of 50 to 61% which allows envisioning the future development of high-performance, flexible devices for high-resolution haptic feedback systems.

Benchmarking against both motion artifacts and standard gel electrodes also provides critical context for evaluating the performance of our 3D skin-interfacing electrodes. The 3D structures maintain stable signal quality under movement and skin deformation (shift, rotation, or twist), largely due to their mechanical interlocking and adhesion with the skin (fig. S40, A and B). Analyses of contact impedance further allow comparing these 3D electrodes’ performance with standard 2D counterparts coated with a commercial conductive gel, specifically formulated for wearable electrodes (fig. S41, A to D). In particular, 2D gel–Au electrodes exhibit contact impedance of ~5.4 kilohm (at 1 kHz), lower than that of 3D (dry) electrodes. Adding the conductive gel onto the 3D electrodes induces a reduction of impedance down to ~2.6 kilohm (at 1 kHz). The current threshold follows the same trend and exhibits a reduction due to the presence of the gel, compared to the dry counterparts (fig. S41E). The 3D gel–coated electrodes also exhibit improved impedance stability (lower signal fluctuations and motion artifacts) compared to the 2D gel–coated electrodes (fig. S40C). Therefore, the 3D electrodes outperform the 2D counterparts not only in the dry state but also with the gel interface, for which standard electrodes often suffer from drying or delamination. This highlights their potential not only for electrotactile stimulation, where stable skin–electrode coupling is crucial, but also for electrophysiological applications, as their reduced motion artifacts and favorable impedance characteristics suggest improved signal reliability and robustness during recording.

We should lastly note that although the 3D millineedle structures were produced by 3D printing, their integration with thin-film devices relied on laser-based patterning, which overcomes the limitations of lithography on nonplanar surfaces. While the electro-haptic device example presented here corresponds to the simplest d-3DPLM case (with a planar envelope surface), overlapping with conventional 2D micromachining, our approach can be used on preshaped curved or sloped patches by dynamically adjusting focus in real time, something not achievable with conventional tools. In addition, this capability could open the possibility of defining conductive pathways on the same 3D millineedle arrays, offering routes to higher electrode densities and more versatile device architectures.

### Wireless contact lenses for light therapy with seamless 3D biointegration

**Fig. 4.**
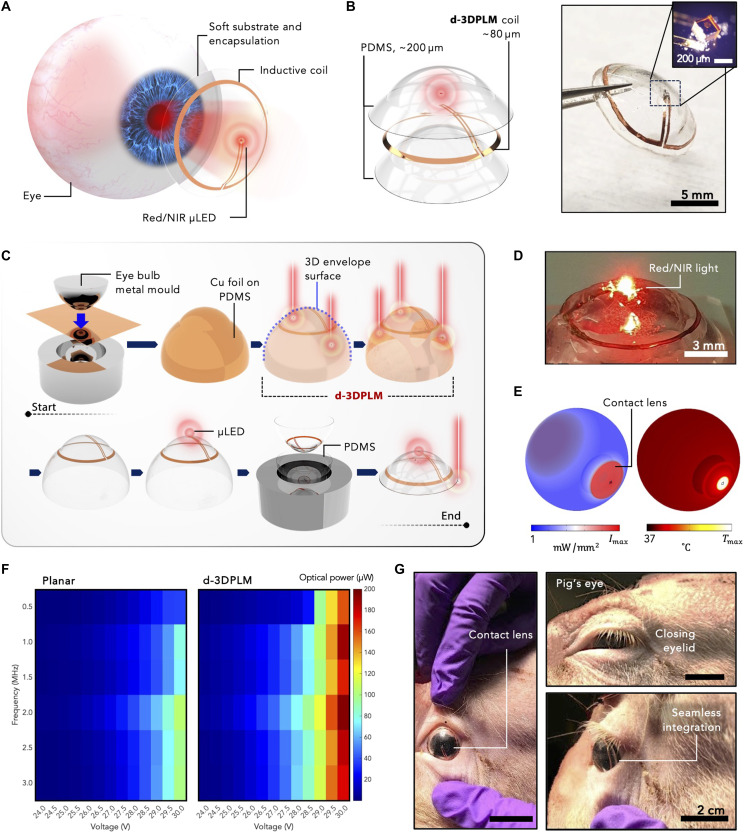
Wireless μLED-integrated contact lenses fabricated through d-3DPLM. (**A**) Conceptual scheme of light therapy delivered by a wireless contact lens with an integrated μLED. (**B**) Exploded view of the structure of the wireless contact lenses microfabricated through the d-3DPLM process. The photo and the inset show the real device and a magnified optical micrograph of the μLED, respectively. (**C**) Fabrication process flow of the contact lenses. (**D**) Red/NIR light emitted by the μLED of the contact lens during wireless operation. (**E**) Light and heat distribution due to the μLED emission, according to FEM simulations. (**F**) Comparison of the optical power emitted by the μLED, between the planar and d-3DPLM configuration [for the single-turn design (D1)], in terms of input voltage and frequency (heatmaps). (**G**) In vivo implementation of the wireless contact lenses on a pig’s left eye, indicating optimal conformality and seamless biointegration. Illustrations entirely created by authors (software: Blender).

Smart bioelectronic contact lenses have been proposed for real-time biosensing, drug delivery, and augmented vision capabilities. They may enable continuous monitoring of tear biomarkers for disease detection and personalized medicine prescription while also supporting ocular drug administration and augmented reality applications (*47*–*50*). However, ensuring conformality and shape adaptability to the eye’s curved, dynamic surface remains a major challenge (fig. S42). On one hand, rigid electronics cause discomfort and instability, necessitating flexible, stretchable materials like ultrathin polymers and liquid metals (*51*–*53*). On the other hand, while these advances improve biointegration, challenges in adhesion, durability, and scalable fabrication persist. Shaping soft and flexible materials to the radially symmetric and curved surface of the eye is a nontrivial challenge. Addressing this is crucial for clinical translation and widespread adoption of smart bioelectronic contact lenses (*54*). Our d-3DPLM approach offers a solution to this, by sculpting the bioelectronic circuits directly along the surface of a 3D eye model, avoiding the need for postfabrication reshaping. By way of example, we present here a soft, wireless contact lens integrated with a μLED for red light therapy, which can be used for the treatment of diabetic retinopathy or other conditions (Fig. 4A) (*55*).

The device consists of a red/NIR LED (wavelength: 635 nm) integrated into an inductive coil for wireless powering. The coil is made out of a copper laminate, embedded in a soft PDMS elastomeric matrix (Fig. 4B). The detailed fabrication process is illustrated in fig. S49. In contrast to previous attempts at producing soft, smart LED contact lenses through photolithography on planar substrates and then encapsulated into silicon elastomers in a contact lens mold (*55*), we first created a metal negative mold of the eye bulb which was used to hot press a flexible copper laminate foil, conferring the desired shape of the contact lens (Fig. 4C and fig. S43). After that, PDMS was cured on the bottom side of the thermoformed metal foil, and a subsequent d-3DPLM process was performed to ablate the foil until the design of an inductive receiver coil was achieved. The laser ablation occurred along the 3D surface of the eye bulb, thus conferring the target shape of the final device without the need for any further shaping steps. Next, the μLED was integrated into the receiver coil using a low-temperature solder (fig. S47), the optoelectronic characterization of which is included in fig. S48. A top PDMS encapsulation completed the device, whose outline was lastly shaped by laser cutting. The d-3DPLM process yields a contact lens with a predefined curved shape, unlike other flat (rigid or flexible) counterparts (fig. S44), exhibiting an axial compression stiffness of 0.3 N/mm (fig. S45). To wirelessly transfer electrical power to the smart contact lens, a transmitter coil was set up in a customized powering platform (figs. S50 and S51), with the circuital scheme in fig. S54. To fully validate the d-3DPLM method, we fabricated four coil designs, i.e., with a single wide or narrow turn, with three turns; and a double-layer three-turn coil (fig. S46 and table S8). Adding more turns or adding a second layer of turns introduced an additional step of lamination and d-3DPLM ablation; this increases the complexity of the design, but it is enabled smoothly by the ease of laser ablation.

Under wireless operation, the μLED could emit an intense light illumination (Fig. 4D), which is transmitted through the PDMS layers and can reach the underlying ocular tissue, confirmed by the FEM simulation of light intensity (Fig. 4E and fig. S53A; see details in note S8). The μLED operation induces a minimal heat generation, below the threshold for tissue damage (*56*) and thus not harmful for the eye (Fig. 4E and fig. S53, B and C): With a set optical power of 50 mW/mm^2^, the highest temperature rise was ~5°C at ~100-μm distance from the eye bulb surface, after 1 s of continuous emission. This is in agreement with previously reported work (*57*). However, the device is aimed at operating in a pulsed mode; thus, the thermal effect on the tissue is expected to be notably lower, well within safe limits. The light intensity was measured, using a photodetector within the wireless test setup (fig. S50, B and C), for the fabricated d-3DPLM contact lenses and their planar counterparts, varying the applied voltage and the operation frequency. A comprehensive overview of the measured optical power in terms of input voltage and frequency (in a representative range: 0.5 to 3.0 MHz) is reported in figs. S54 and S56, for the four receiving coil designs. The optical power increases with the applied voltage due to an increase in the induced current in the coils (fig. S52), while the frequency dependence is more irregular, likely due to the absence of a resonant capacitor. Compared to the 2D devices, the 3D contact lenses generally exhibited higher optical power (up to ~200 μW) at the same voltage and frequency (Fig. 4F). This can be ascribed to the curved shape of the coil with the turn(s) laying partially closer to the transmitting coil, resulting in increased induced current. In this nonresonant inductive power transfer configuration, the relative distances between transmitting coil, receiving coil, and photodetector affect the resulting emitted optical power (fig. S55). In addition, the coil geometry affects the inductance, coupling efficiency, and frequency response (see note S7). For planar 2D coils, the inductance follows Wheeler’s formula, i.e., $L\approx \mu_{0}N^{2}r^{2}/\left(8r+11d\right)$ , where $\mu_{0}$ is the permeability of free space, N is the number of turns, r is the mean coil radius, and d is the coil trace width. In a 3D shape, especially with multiple layers or turns, the coil is an intermediate between a planar inductive coil and a single-layer solenoid, whose inductance is given by $L=\mu_{0}N^{2}A/l$ , with A cross-sectional area and l length of the coil. The combination of higher cross-sectional area and length causes an increase in the coil inductance, also due to increased spatial volume and better magnetic flux capture. Thus, a higher inductance in 3D coils allows for better low-frequency operation, while the 2D counterparts favor higher frequency operation due to their lower inductance. While the 2D coils suffer from weaker coupling with the transmitter, unless perfectly aligned, the 3D shape enables a stronger coupling, leading to higher mutual inductance and better power transfer efficiency. Our fabrication process is compatible with the integration of capacitors or more complex tuning circuitry in the lens to achieve a resonant wireless power transfer (see note S7 and fig. S57D). Our d-3DPLM process can be used seamlessly to design and fabricate both resonant and nonresonant coils, with no technological difference in the underlying fabrication steps. This versatility allows the choice of coupling strategy to be determined purely by application requirements while leveraging the same rapid and flexible prototyping workflow.

We lastly demonstrate the enhanced 3D surface conformality of the fabricated μLED-integrated contact lenses: They were placed on pig’s eyes (a single-turn contact lens is reported as an example in Fig. 4G and fig. S58). The preformed 3D shape of the devices provided superior conformality and seamless 3D biointegration without need of any further manipulation or bending steps. The customized design enabled by the d-3DPLM process allows for simple adaptation of the contact lens to the real shape of the eye and corneal topography. This advantage brings a transformative potential for advanced treatments of diabetic retinopathy and other conditions.

### Tunable-height 3D MEAs for in vitro recording of cerebral organoid activity

Cerebral organoids provide valuable models for studying neuronal development, network formation, and neurological disorders (*58*). Their spontaneous electrophysiological activity is a key indicator of neuronal function, making high-resolution, 3D electrophysiological recording a crucial tool for both fundamental neuroscience and disease modeling (*59*, *60*). While traditional MEAs have been widely used for in vitro recordings (*61*), their planar nature limits their ability to fully capture the complex 3D network activity of cerebral organoids. Efforts to develop 3D-compatible MEAs have led to solutions such as flexible buckled (*62*), rolled (*63*), and kirigami-inspired (*64*) MEAs, as well as stretchable MEAs (*65*), but these often require intricate fabrication steps, external actuators, and chemical treatments that could interfere with cell viability, experimental reproducibility, and considerable amounts of time to achieve an optimal tissue biointegration.

**Fig. 5.**
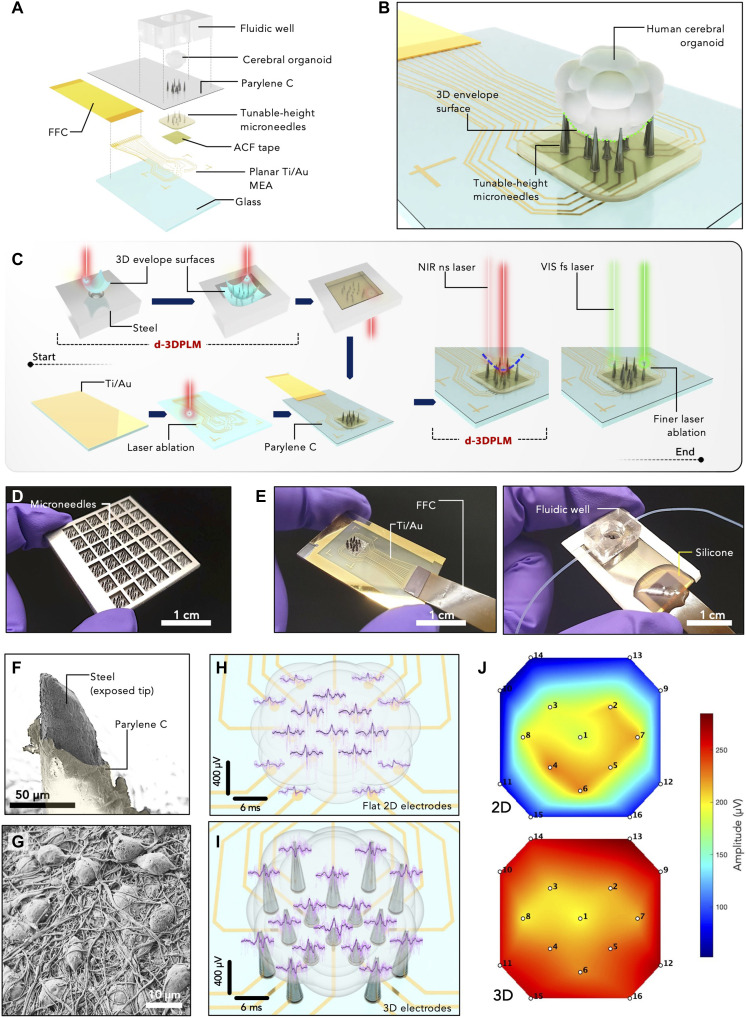
Tunable-height 3D MEAs, fabricated through d-3DPLM, for in vitro recording of organoid activity. (**A**) Exploded view of the 3D multielectrode array device including the cerebral organoid on top of the MN electrodes. FFC, flexible flat cables. (**B**) Illustration of the cerebral organoid on top of the MN electrodes with tunable height, fabricated through the d-3DPLM process along the 3D envelope surface highlighted in green. (**C**) Schematic of the main steps of the fabrication process for the 3D MEAs, highlighting the use of the laser ablation. The visible fs pulsed laser is used in the end to perform a finer ablation step to fully expose the tip of the electrodes with the desired area. VIS, visible. (**D** and **E**) Real photo of the 3D MEAs, after fabrication (D) and of the complete device, after assembly of fluidic well (E). (**F**) False-color SEM image of an MN electrode with the tip exposed form the parylene encapsulation. (**G**) SEM micrograph of the neuronal cells on the surface of the whole cerebral organoid. (**H** and **I**) Electrophysiological recordings of the cerebral organoids placed on top of the 2D (H) and d-3DPLM (I) MEAs. The overlaid LFP waveforms (20 peaks in purple with the mean waveform in black) were detected by the 16 electrodes in a 10-min recording. (**J**) Heatmaps showing the space distribution of the voltage amplitude of the electrophysiological signals detected from the cerebral organoids on the 2D and 3D MEAs.

The d-3DPLM process can enable cleanroom-free prototyping of MEAs with customizable 3D properties. We demonstrate a tunable-height 3D MEA for high-fidelity, noninvasive cerebral organoid recordings. This platform features stainless steel MN electrodes monolithically fabricated and integrated with laser-patterned Ti/Au interconnects on a glass substrate (Fig. 5A). Our key innovation lies in customizing MN height based on a predefined 3D shape, achieved in a single laser-ablation process. The tunable heights allow precise of electrode position relative to the organoid surface, ensuring optimal contact and preserving the organoid’s 3D architecture (Fig. 5B and fig. S62). Unlike self-folding or stretchable MEAs, our approach is deployable on preformed organoids without tissue or device manipulation, offering a scalable solution for next-generation neurodevelopmental research.

To create the electrodes, we developed a microfabrication process flow in which MN-shape pillars were initially milled down from a block of stainless steel, through NIR ns pulsed laser ablation (Fig. 5C and fig. S59). Different sets of laser parameters were used to refine the conical shape of the MN (figs. S27 to S30), with apical tip diameters down to 15 to 25 μm and heights ranging from ~300 μm to 2.8 mm (representative MN electrodes are visible in Fig. 5D and figs. S60 and S64). The ablation process for creating the MNs included steps to increase the ablation depth (height) and others to refine the MN tip with a reduced projected diameter. After achieving the desired heights and tip diameter, the engraved cavity hosting the MNs was filled with a water-dissolvable poly(vinyl alcohol) (PVA) matrix and then flipped upside-down to undergo a second step of laser ablation (fig. S61). This process was used to extricate the MNs from the rest of the steel block. The array of MN electrodes was then integrated onto a laser-patterned Au planar MEA using a room temperature anisotropic conductive film (ACF) tape (Fig. 5E and figs. S63 and S66), with an adhesion of ~0.25 N/mm (fig. S67). Afterward, a 5-μm-thick parylene C coating conformally encapsulated the 3D electrodes and the rest of the device. A further mild laser ablation was performed in two steps to deinsulate the electrodes’ tips. A first gentle d-3DPLM process through the ns pulsed laser system was carried out over the envelope area tangential to the tips of the 3D electrodes and defocused by 10 μm above the tips. This allowed removal of part of the thin-film parylene C encapsulation. A second more precise step was performed through a fs pulsed laser system with the beam focused on the tip of each electrode and a projected diameter of 20 μm. This step was used to polish the electrodes’ tips, removing any residues of parylene remaining adherent to the rough steel surface and accurately outline the electrode’s tips (Fig. 5F and fig. S65), creating a regular and reproducible electrode surface and providing the desired size ( ≲50 μm in diameter) and morphology for the electrophysiological recordings. To facilitate the organoids’ culture medium replacement, we integrated the array within a PDMS fluidic well, comprising an inlet and outlet for the integration of the device to a system for medium recirculation (Fig. 5E).

We performed EIS on the integrated MN electrodes in phosphate-buffered saline (PBS), comparing them with a planar array of Au MEAs. The impedance amplitude for a standard planar array of Au MEAs (fig. S68A) was consistent with previously reported values for thin-film Au electrodes (*66*), exhibiting reproducible values both within the same device and across different devices, with an average impedance amplitude at 1 kHz within 20 to 30 kilohm (reproducible for *n* = 3 devices, for a total of 48 recording electrodes with a surface area of 1256 μm^2^). The integration of the 3D stainless steel electrodes induced reduced the average impedance amplitude at 1 kHz to 4 to 6 kilohm (reproducible for *n* = 6 devices, for a total of 96 recording 3D electrodes), without any encapsulation, thus with a surface area of ~9 × 10^5^ μm^2^ for MNs with 300-μm height, 500-μm base radius, and 40-μm tip radius (fig. S68B). Figure S68C shows the EIS spectra for the encapsulated MN electrodes with exposed tips, where the exposed surface area is ~5 × 10^3^ μm^2^, yielding an average impedance amplitude at 1 kHz in the range 30 to 150 kilohm; the wider variability can be ascribed to deviations from an ideal tip de-insulation process. Cyclic voltammetry (CV) was performed to assess the charge storage capacity (CSC) of the 3D electrodes (fig. S68, D and E). The 3D MNs exhibited enhanced ability to reversibly accumulate and release charge, with a CSC of 1.4 nC/μm^2^ compared to 0.2 nC/μm^2^ for the planar MEAs (fig. S68F). The 3D features, with larger exposed surface area, showed improved electrochemical performances, facilitating charge transfer at the electrode-electrolyte interface.

Following the successful fabrication and electrochemical characterization of the 3D MEAs, we conducted electrophysiological recordings as a proof of concept for the devices to capture neuronal activity in brain spheroids. For this purpose, we selected a 1-year-old human cerebral organoid and delicately transferred it onto a 3D MEA (fig. S69). As control, whole organoids were placed also on flat 2D MEAs (fig. S70). Additional culture medium was used to fill the fluidic well and keep the organoid soaked and hydrated (fig. S71). The organoid (without medium and fixed after the experiments; fig. S72A) laying on the electrodes exhibits a conformal integration with the 3D MN electrodes, in contrast to the 2D MEA (fig. S72, B to D) retaining its shape and its microscopic neuronal network (Fig. 5G and figs. S73 and S74). Immunofluorescence imaging confirmed the presence of live neuronal nuclei, axons, and astrocytes (fig. S76). In the case of too small organoids, they were sliced and cultured through the air-liquid interface cerebral organoid (ALI-CO) protocol (see Materials and Methods and the Supplementary Materials). This allowed us to perform the experiments also on internal parts of the smaller organoids’ body where neuronal activity is generally less pronounced (without appreciable differences in the detected signals). During the recordings, a small volume of ALI-CO culture media (<200 μl) was used to fill the fluidic well that hosted the array. After 10-min stabilization in an incubator at 37°C, the device was connected to a commercial electrophysiological recording platform (Intan) (fig. S75). 3D recording of spontaneous local field potential (LFP) waveforms with distinct patterns was obtained across the curved surface of the spheroid, with an average LFP duration of ~1.5 ms and amplitudes up to ~200 μV (fig. S77), comparable with prior works (*67*). Figure 5 (H to J) compares the electrophysiological recordings between the d-3DPLM MEAs and their 2D counterparts. The planar 2D MEAs only capture signals from the region of the organoid that is in direct contact with the electrodes, leading to limited spatial coverage and weaker signal amplitudes due to poor interface with deeper neuronal networks. In contrast, the tunable-height 3D conformal MEAs, which follow the natural curvature of the entire organoid, provide a more comprehensive recording of neuronal activity by making direct contact with a larger surface area and accessing signals from multiple orientations. This configuration enables higher signal fidelity and more accurate mapping of spontaneous and evoked neuronal network activity. In addition, 3D electrodes better preserve the natural, free-floating state of the organoid, reducing mechanical stress and maintaining physiological-like conditions, which can lead to more representative electrophysiological measurements of developing neuronal circuits.

## DISCUSSION

This study highlights the potential of pulsed laser–based microfabrication, particularly ns pulsed NIR lasers, in advancing 3D bioelectronics. Successful device-level demonstrations include electro-haptic wearable patches, MEAs, and wireless contact lenses, showcasing the precision and versatility of d-3DPLM in creating high-resolution, low–thermal damage bioelectronic systems.

Electro-haptic patches for tactile stimulation based on customized 3D features mark a major step in wearable bioelectronics, offering flexibility and minimal invasiveness. The 3D-printed millineedle electrodes enhance the stimulation capabilities, achieving designs not possible with standard photolithography, and allow stimulation at higher resolutions and with lower power requirements than previously possible. These 3D electrodes are designed to rest on the skin surface without penetrating it. Their geometry facilitates conformal contact and improved signal acquisition through their 3D architecture, but no transdermal penetration occurs during use. The devices are secured using mild, skin-safe adhesives to ensure stable placement during recording. This approach avoids insertion or dermal disruption, offering clear advantages in terms of user comfort and noninvasiveness.

Wireless contact lenses for red light therapy demonstrate bioelectronic potential in diagnostics and therapy, particularly for eye strain and inflammation. The preformed design of the conformable shape of the contact lens, enabled by the d-3DPLM, allows a custom-fit design that supports personalized medicine and advanced ocular treatments. By matching the device shape to that of the patients’ eye bulb, this approach represents a previously unidentified paradigm in the fabrication of smart devices for the treatment of ocular and other related conditions. We note that the fabrication of coil structures on wireless contact lenses revealed challenges such as residual stress, partial delamination, and laborious lamination of PDMS encapsulations, which highlight the developmental stage of this approach. Although the method avoids common issues associated with thin-film–based techniques, including wrinkling, curling, and geometric asymmetries, it does not inherently outperform established strategies such as transfer printing of serpentine thin films. Instead, the primary value lies in demonstrating that d-3DPLM enables direct prototyping of integrated wireless components on curved, nonplanar substrates without the need for wet chemistry, masks, or transfer steps. This capability emphasizes accessibility, flexibility, and adaptability in form factor, making the technique particularly suited to exploratory research and early-stage device development. While systematic benchmarking against transfer-printed coils of equivalent geometry remains a future direction, d-3DPLM should be regarded as a complementary route, one that provides distinct advantages in speed, customizability, and integration on soft or geometrically complex substrates.

Similarly, the tunable-height 3D MEAs provide improved spatial resolution for monitoring cellular and tissue activity, promising more accurate in vitro diagnostics for biopotential signals in neurological and cardiac research. By integrating MN electrodes with a 3D architecture and a tunable height, these arrays provide an enhanced ability to monitor electrophysiological signals with enhanced accuracy compared to flat, 2D counterparts, leaving the tissue microstructure intact.

The fabrication of the 3D MEAs in this study illustrates the complementarity between d-3DPLM and fs laser micromachining. This hybrid approach reflects a realistic balance between processing speed, accessibility, and resolution. Specifically, d-3DPLM excels in rapidly shaping and patterning curved substrates over millimeter-scale depth variations, whereas fs laser micromachining provides the ultrahigh precision necessary for fine features where thermal effects must be minimized. Rather than a limitation, this combination highlights the complementary strengths of different laser modalities, analogous to hybrid additive-subtractive strategies widely used in microfabrication. d-3DPLM extends fabrication to nonplanar and mechanically compliant substrates that fs laser systems alone cannot readily process, while fs laser ablation ensures resolution in critical regions. After the two-step process, the MN electrode tips result in clean and well-defined exposed geometries (fig. S65, A and B); in contrast, tips processed only with the fs laser exhibit irregular, undersized openings and poor edge definition (fig. S65C), underscoring the need for the hybrid approach to achieving reliable electrode exposure. Together, these techniques provide a versatile platform for realizing advanced 3D bioelectronic devices.

We also highlight that this work was designed as a proof of concept to demonstrate the integration of laser-patterned 3D electrodes on a single platform for electrophysiological recording on cerebral organoids, rather than as an optimized recording solution. Long-term stability of organoid positioning remains a known challenge in 3D electrophysiology; however, immunohistochemistry and scanning electron microscopy (SEM) analyses confirmed that viable tissue-electrode contact was maintained throughout our experiments.

There are several limitations that must be addressed before these technologies can be widely applied. First, while the ns pulsed NIR laser technique enables precise micromachining, the scalability of the process to larger or more complex device architectures remains a challenge. Table S9 lists the processing times, yields, and degree of automation for the fundamental ablation patterns, and note S10 discusses the scalability and reproducibility of the NIR laser ablation process through quantitative metrics. As the devices grow in size or complexity, maintaining the required precision and ensuring consistent quality become more challenging.

Besides that, we acknowledge that the spatial resolution of the fabricated 3D electrodes presently lags behind that of their 2D counterparts. This limitation primarily arises from trade-offs inherent to laser-matter interactions and the intrinsic resolution constraints of the NIR ns laser. Specifically, achieving a high density of 3D electrodes is not feasible at the reported size scales (e.g., millineedle electrodes for haptic devices) or with the chosen material system (stainless steel MN electrodes) and the used laser wavelength/pulse durations. Nevertheless, the present 3D demonstration serves as a proof of concept, highlighting the capability of d-3DPLM to produce complex geometries, a distinct advantage over conventional planar lithography. We view this as a foundational step toward improving resolution, with potential pathways including laser parameter tuning, integration with complementary fabrication techniques, and miniaturization of the electrode designs.

In addition, the interaction between the laser and certain materials, particularly biologically active or water-soluble materials, requires careful optimization to prevent degradation or unwanted effects during fabrication. Moreover, extensive ablation of particularly hard and brittle materials (such as metal blocks or advanced ceramics) can be time consuming and may be rate limiting to in a scaled-up manufacturing process.

Last, we acknowledge that certain aspects of the device fabrication, such as the integration of 3D-printed millineedles, or of the 3D MN electrodes within the MEA fabrication process, require multistep assembly. This may limit full automation and introduce variability in device resolution. The current differences in resolution and form factor primarily arise from curvature-induced focal misalignment at extreme geometries and mechanical tolerances in multimaterial stacking, rather than fundamental limits of the laser process itself. These challenges can be mitigated by integrating automated alignment and focusing modules, as already common in industrial laser micromachining. In practice, a fully automated workflow could include pick-and-place tools to align and stack all components, including 3D MEA electrodes after ablation, in a single step, paving the way toward scalable and reproducible device manufacturing.

Nonetheless, the core d-3DPLM process remains fundamentally monolithic, in that the patterning and micromachining of all functional layers (e.g., metal interconnects, insulators, and polymers) are accomplished in a single, solvent-free, maskless workflow using a dynamically focused laser. The monolithic nature of the process is exemplified in the fabrication of 3D MEA electrodes, where a single ablation step yields MN electrodes of varying heights in one batch. Table S10 lists the processing times and yields for the complete fabrication processes of the devices presented in this work, including the number of manual interventions for the assembly, which contribute to reduce the overall yield. It is important to distinguish between the inherent scalability of the laser patterning process and the prototyping-specific assembly steps used in this study. For instance, the 3D-printed scaffold in the electro-haptic patches functions solely as a passive structural support rather than a patterned component and could, in future implementations, be replaced with bioresorbable or injection-molded structures to enhance scalability.

In summary, by leveraging laser micromachining with surface-conformal focal tracking, we introduce a scalable, precise, and fully automated solution for customizable 3D bioelectronics. The presented d-3DPLM approach allows for the fabrication of highly tunable 3D structures without the need for lithographic masks or multistep etching processes. The ability to sculpt complex geometries in a single monolithic step provides notable advantages in the development and application of future bioelectronic interfaces.

## MATERIALS AND METHODS

### Experimental design

Short PLM enables microfabrication of advanced 3D bioelectronics. We detail below the fabrication and characterization of three main system-level demonstrations, i.e., electro-haptic wearable patches, MEAs for in vitro diagnostics, and wireless contact lenses for red light therapy.

### Materials

Materials used in this study included standard rigid/flexible substrates, conductors, and encapsulants. Glass substrates for 3D MEAs consisted of glass microscope slides. Polymer substrates consisted of PDMS [Sylgard 184, Dow, ratio 10 (base):1 (curing agent)] and poly(2-chloro-p-xylylene) (parylene C). The latter was provided by Specialty Coating Systems (SCS) in the form of dimer powders. Parylene C deposition process was performed by a room temperature CVD equipment (SCS, PDS 2010 Labcoater 3 system model). The powdered dimer vaporized at a temperature of ~175°C and at a pressure of 15 × 10^−3^ torr to undergo a pyrolysis and be reduced in monomers; then, the polymerization of the gaseous monomers occurred at ~700°C; the gas entered the deposition chamber at 20° to 25°C and a conformal polymeric coating deposits on the substrate. An amount of 2 g of dimer powder yielded approximately a deposited layer with a thickness of 1 μm, and the process lasted ~2 hours. Conductive materials included stainless steel (AISI 304, RS Components), copper laminates Cu (18 μm)/Kapton (80 μm)/Cu (18 μm) (RS Components), Ti/Au thin films derived from Ti (99.995% pure, 2.00″ diameter, 0.250″ thick), and Au (99.99% pure, 2.00″ diameter, 0.0625″ thick) sputter targets (Kurt J. Lesker). Reactive sputtering (NanoPVD, Moorfield Nanotechnology) yielded uniform layers of metal (materials: Ti and Au) on the previous substrates (glass and parylene C). Room temperature ACF tape (RF EMI shielding tape 9703, 3M) was used as conductive adhesive for the 3D MEAs. Flat flexible ribbon cables (RS Components) were attached to the MEA devices using an ACF heat bonding machine (3T830B, 3T Frontiers Pte. Ltd.) with a high-temperature ACF tape (AC-7813KM-25, Hitachi Chemical).

### NIR nanosecond PLM process

The micromachining processes described in the work were performed using a commercial ns pulsed YVO_4_ laser system (wavelength: 1064 nm; pulse duration: 5 to 100 ns; beam diameter: 30 μm; maximum processing area: 50 mm by 50 mm; maximum output power: 13 W; pulse repetition rate: 1 to 400 kHz; MD-X2025A Keyence Laser). The average power (0 to 100%), scanning speed (100 to 1000 mm/s), pulse frequency (1to 400 kHz), and the number of repetitions were adjusted according to different application scenarios. The micromachining process enabled by this laser allowed four operation modes: (i) material ablation, consisting of controlled, patterned thinning; (ii) complete removal, consisting of etching of an entire material layer; (iii) shaping, consisting of cutting and patterning of the outline and/or inner shapes in the device designs; (iv) creation of 3D shapes through a single ablation process along a preestablished 3D surface, plugged into the software (Marking Builder Plus) as .stl file (d-3DPLM).

### Characterization of the laser micromachining process

#### Cut outlines and ablated thickness

Square outlines and square regions of the tested material (1 mm by 1 mm) were respectively cut and ablated using various laser settings (average power, scanning speed, pulse frequency, and the number of repetitions) at fixed grid distance (1 mm). To determine the ablated thickness, we used optical microscopy (Keyence VHX-7000 Digital Microscope, with 3D mapping capabilities) and mechanical probing (Dektak XT-A profilometer, Bruker).

#### Spatial in-plane (lateral) resolution

Ablation with different projected widths between two adjacent laser scans created ribbon shapes on stainless steel blocks. Ribbon shapes (length: 5 mm) were achieved by separating two parallel ablated regions with well-controlled projected widths. Different sets of parameters (average power, scanning speed, pulse frequency, number of repetitions) were used to characterize in details the lateral resolution. The same analysis was carried out for ablation of Au thin films on glass and parylene C substrates and for the copper laminates on PDMS. Optical microscopy (Keyence VHX-7000 Digital Microscope) was used to measure the widths and cross-sectional profiles of the ribbon shapes on stainless steel, as well as the ablated regions on the other materials (Au and Cu).

#### Spatial out-of-plane (vertical) resolution

Ablation of a square area (1 mm by 1 mm) on stainless steel blocks was performed on different focal planes (*z* direction), distanced by preset steps (from 10 to 400 μm). The ablation produced wells in the material, and the depth of these wells was measured using optical microscopy (Keyence VHX-7000 Digital Microscope) and mechanical profilometry (Dektak XT-A, Bruker). The minimum detectable change in the well’s depth determined the out-of-plane resolution of the laser ablation. Concerning the other materials, i.e., thin films on parylene C substrates, characterization involved studies of damage to the bottom polymeric layer (5 μm) when ablating the top metal thin film (200 nm). After the ablation and the complete removal of the thin metal, the residual area was 3D-scanned optically (Keyence VHX-7000 Digital Microscope), and the depth of the ablated region was recorded. Comparing this parameter with the original thickness of the top layer provides a useful indication of how much damage is imparted to the underlying substrate and how much selective the ablation is to the top layer.

#### FEM of the laser ablation

COMSOL modeling was adopted to analyze and simulate the energy absorbed from ns pulsed laser radiation during the ablation process. We used the transient heat transfer module, considering a tetrahedral mesh and a laser beam of 30-μm diameter. The absorbed energy was modeled considering a body heat flux in the material region of the laser spot, spread according to a Gaussian distribution. Other parameters used for this study regard the thermal properties of the materials: thermal conductivity and thermal diffusivity of stainless steel, glass, Au, and parylene C. A detailed explanation is reported in note S3.

#### Dynamically autofocused 3D pulsed laser micromachining

To achieve precise, tunable, and monolithic fabrication of 3D shapes, we exploited the 3D marking functionality of the ns pulsed laser system, which enables ablation along complex topographies by directly integrating a 3D surface (.stl file) into the laser marking software (Marking Builder Plus). This method ensures that the focal point of the laser beam dynamically followed the curvature of the inputted surface, regardless of the position relative to the target material. Adjusting the original position of the material top surface allows for spatially controlled material removal without additional postprocessing. This method can be used both in raster (ablative) and in cutting mode. We validated it both with the ablation of 3D MEAs and for the shaping of the wireless coils for smart contact lenses.

### Fabrication of wearable electro-haptic patches with millineedle electrodes

Electro-haptic patches were fabricated in rigid and flexible forms. For the rigid devices, microscope glass slides were used as substrates. 3D elements with different sizes and shapes (cones, cylinders, and pyramids) were 3D printed (Formlabs 4, Clear resin V4.0) and glued onto the glass. Parylene C (5 μm) was deposited by room temperature CVD (SCS, PDS 2010 Labcoater 3) to encapsulate the 3D printed structures. Next, Ti (10 nm)/Au (100 nm) layers were deposited onto the parylene C layer by reactive sputtering (NanoPVD, Moorfield Nanotechnology). The metal thin films were patterned through the d-3DPLM process using a ns pulsed laser system (MD-X2025A Keyence Laser), with the following parameters: average power, 30%; scanning speed, 1500 mm/s; pulse frequency, 100 kHz; spot variable, 0; repetitions for a single layer, 3; number of layers, 1; 3D envelope surface, 2D plane. In parallel, a 90-μm-thick medical tape (1577, 3M DC Medical “2-in-One” Differential Polyester Film Tape) was laser-cut with a CO_2_ laser (GravoGraph Laser Solution LS900, 10.6-μm wavelength), opening areas for exposing the electrodes and the connection pads. The following parameters were used: average power, 40%; scanning speed, 1000 mm/s; number of layers, 1. The medical tape was then used as encapsulation and manually aligned on top of the Au/parylene C layers. The devices were electrically connected with electrical wires using an Ag paste (SA155D, DZP Technologies); the connections were lastly encapsulated with biocompatible silicone (Dowsil 734). The flexible version of the electro-haptic devices was obtained following the same process, but instead of attaching the 3D structures directly onto the glass substrates, a preemptive parylene C deposition (5 μm) was performed on glass coated in 3% soap solution (Micro 90, VWR). After completing the fabrication process, the device was peeled off from the glass, connected, and sealed.

### Characterization of wearable electro-haptic patches with millineedle electrodes

#### Optical microscopy

The 3D millineedle electrodes were observed through 3D optical microscopy (Keyence VHX-7000 Digital Microscope).

#### Mechanical characterization

The mechanical stiffness of the fabricated electro-haptic patches was measured using a force displacement machine (Mark-10 Test Frame F-105) with a load cell of 500 N. Uniaxial tensile tests were performed with a speed of 1 mm/min, until break occurred.

#### Contact (dry) impedance spectroscopy

The electrochemical impedance was recorded with an impedance analyzer (PalmSens4 potentiostat/galvanostat, Alvatek) with PSTrace 5.9 between 1 Hz and 1 MHz, with a driving ac voltage amplitude of 0.01 V and a stabilization time of 100 s. A two-electrode setup was adopted, with the device’s active electrode being the working electrode and the device’s ground electrode being the counter and reference electrode (i.e., two-electrode setup). During the measurements, the device was in contact with the skin of the volunteer, with an applied pressure of ~50 to 80 kPa (deriving from an applied force of ~5 to 8 N over a finger contact area of ~1 cm^2^). To quantify the pressure, a commercial thin-film resistive pressure sensor (RP-S40-ST, SEN0296) was used, and its calibration curves were used to identify the range of pressures applied on the electro-haptic patches. The experiments were also conducted using a conductive gel (Weaver Ten 20 Conductive, Neurodiagnostic electrode paste) as interface between the electrodes and the skin.

#### In vivo electrical stimulation protocol for finger and palm

Biphasic pulsed direct current stimulation was performed by connecting the device’s electrodes (active and ground) to an electronic interface based on a source-measure unit (Keithley 2612B) controlled through a customized Python-based script. Pulses of different current levels in the range of 30 to 100 mA were applied at a frequency of 100 Hz, with a pulse width of 200 μs, for the 2D and 3D electrodes, respectively. The devices were placed onto the volunteer’s skin, with the finger or palm fixed in a 3D printed custom mold. This allowed performing accurate and reproducible measurements. Current amplitudes were increased stepwise and gradually until the volunteer could feel the stimulation effect. This current level was taken as the stimulation threshold.

#### In vivo blind tests for haptic recognition on the wrist

We recruited four volunteers (ages 22 to 40) for the user study. They were required to wear the flexible electro-haptic patch on the left wrist (fig. S37) to perceive the haptic feedback and tell which electrode was triggered to stimulate the wrist. The volunteers were blinded to the true stimulation site. For each stimulation, there were four different sites, and they were triggered randomly in pairs and repeated five times. The center-to-center distances of every two electrodes were fixed at 11.6 mm. Stimulation was applied to each electrode using the same parameters as above, setting the current at the sensation threshold recorded for each individual. The testing on volunteers was performed with their full, informed consent. All the human experiments were performed under the University of Oxford Medical Sciences Interdivisional Research Ethics Committee, reference R95025/RE002. All research was performed in accordance with the relevant guidelines and regulations.

### Fabrication of wireless μLED-integrated contact lenses

The fabrication process is depicted in fig. S49. An aluminum (Al) mold was machined through CNC tools, conferring the negative shape of the eye bulb. An Al piston with the same positive profile was machined in the same way. The two pieces were assembled to create a system that could hold in place a flexible Cu laminate foil and simultaneously hot press (thermoforming) it with the piston. This step (80°C, 2 min) conferred to the foil the desired shape for the contact lens. After performing this step, a thin layer (~200 μm) of PDMS was cured (80°C, 30 min) on the bottom side of the thermoformed metal foil and on the top of the piston. This layer of PDMS represented the substrate of the contact lens. After thermoforming, a d-3DPLM process was performed with these parameters (average power: 80%, scanning speed: 200 mm/s, pulse frequency: 100 kHz, spot variable: 0, repetitions for a single layer: 20, number of layers: 4, *z* steps: 50 μm) to ablate the foil and achieve the target design of an inductive receiver coil. A mild sonication (80 kHz, 10 min) in isopropanol was carried out to remove any residues of the ablation process. Next, a red/NIR μLED (EOLC-635-34, wavelength of 635 nm, EPIGAP Optronic GmbH) was integrated into the coil at the center of the contact lens through pick-and-place method. A low-temperature soldering process was adopted by manually dispensing bumps of a Sn/Bi/Ag solder paste (Chipquik, 90, catalog no. SMDLTLFP10T5) onto the exposed connection pads, then positioning the μLED accurately, and lastly reflowing at 165°C in a hot oven to produce the electrical connection. To hermetically protect the μLED and fixing it mechanically, so as to avoid undesired out-of-plane buckling deformations or damages, a droplet of biocompatible silicone (Dowsil, 734) was pneumatically printed onto the μLED, let it spread for 2 min, and cured at room temperature for 30 min. Using the same piston-cylinder system, a PDMS layer (~200 μm) was cured (80°C, 30 min) on top of the μLED-integrated contact lens, serving as top superstrate. After that, the external outline of the contact lens was shaped by laser cutting (average power: 30%, scanning speed: 1000 mm/s, pulse frequency: 10 kHz, spot variable: 0, repetitions for a single layer: 50, number of layers: 1, *z* steps: 0).

### Characterization of wireless μLED-integrated contact lenses

#### Characterization and FEM of the μLEDs

The brightness and spectrum of the red/NIR μLEDs, wired to a dc power supply (Delta Elektronika, E300-0.1), were analyzed with a spectrophotometer (Ocean optics USB2000+, 20.8 software). The intensity of the light was measured with a photodiode power sensor (Thorlabs, PM100D). The μLEDs were characterized both in wired and wireless configurations. FEM analyses of the thermal and optical emission of the μLEDs were carried out in COMSOL Multiphysics.

#### Mechanical characterization

The mechanical rigidity of the fabricated contact lenses was measured using a force displacement machine (Mark-10 Test Frame F-105) with a load cell of 2.5 N. Uniaxial compression tests were performed with a speed of 50 mm/s, in the center of the coil (i.e., at the μLED site), deforming the lens inward.

#### Power transmission efficiency measurement

The wireless power transmission system consisted of a power amplifier and a custom transmitter coil. The power amplifier generates a square wave voltage of varying magnitude (5 to 100 V) and frequency (100 kHz to 5 MHz) from a dc supply by a full-bridge switch mode power converter. The transmitter coil has four turns of 300 × 46 AWG litz wire, and it measures 2.5 cm OD and 1.5 cm ID. The use of gallium nitride (GaN) transistors for the full-bridge and litz wire for the transmitter coil ensures very low power loss in the system.

#### Electrical characterization

The self-inductance of the fabricated coils was measured using an LCR phase sensitive meter (N4L PSM1735, NumetriQ). The mutual inductance between the transmitting and receiving coils was measured with the same tool, using two series configurations with positive or negative coupling. The input/output power at the transmitting coil was determined while simultaneously recording the coil voltage and current waveforms, respectively with an oscilloscope voltage probe connected in parallel and with an oscilloscope magnetic flux-based current probe (Agilent, N2783A) powered by a Keysight N2779A power supply. The oscilloscope used for this purpose was Keysight InfiniiVision 4000 X.

#### In vivo implementation

In vivo testing used porcine models with similar eye bulbs to those of humans. As in (*7*), the testing was carried out at the Bancroft Centre, University of Cambridge. At the University of Cambridge, the animal procedures were carried out in accordance with the UK Animals (Scientific Procedures) Act, 1986. Work was approved by the Animal Welfare and Ethical Review Body of the University of Cambridge and was approved by the UK Home Office (project license no. PP6076787). Commercial hybrid pigs (Large White × Landrace × Duroc), with a mean body weight of 50 kg, were enrolled in the study. Before the procedures, the animals were considered healthy on the basis of clinical examination. The animals were deeply sedated intramuscularly with a mixture of tiletamine-zolazepam (3 mg/kg) and dexmedetomidine (0.015 mg/kg) and euthanized upon intravenous barbiturates overdose (thiopental sodium, 60 mg/kg). The healthy animals were used also for other experimental protocols (*7*). To that purpose, the bodies were placed in sternal recumbency, and the smart contact lenses were worn on the left pig’s eye. Conformality and biointegration were qualitatively assessed, whereas histopathologic and immunohistochemical analyses were not performed, considering the established biocompatibility of all the constituent materials of the contact lenses. Robustness of the wireless powering was tested, keeping the distance between the receiver coil embedded into the smart contact lens and the transmitting coil within 1 cm, with the parallel alignment of the two coils.

### Fabrication of tunable-height 3D MEAs for in vitro recording of neural organoid activity

The fabrication process of the 3D MEAs is illustrated in fig. S59. Starting from a block of medical-grade stainless steel (AISI 304, RS Components), laser ablation (MD-X2025A Keyence Laser) was performed to mill down the material and create conical pillars (MNs).

The MN electrodes used for these devices were fabricated with the following set of parameters: average power: 30%, scanning speed: 500 mm/s, pulse frequency: 100 kHz, spot variable: 0, repetitions for a single layer: 100, number of layers: 11 (or 15), *z* steps: 100 (or 50) μm.

The heights were controlled by adjusting the focal point of the laser beam. MNs with the same height were obtained just by subsequently lowering the focal point (*z* direction) by preset steps (from 10 up to 200 μm). MNs with radially decreasing heights were obtained by lowering the focal point selectively, starting to mill down the ones in the array that were radially farther from the center of the array. MNs with tunable heights were obtained by setting the focal point of the laser beam along a preset 3D surface, plugged into the software as .stl file. Spherical or ellipsoidal surfaces were used as proof of concept. After achieving the desired heights, the engraved cavity hosting the MNs was filled with a water-dissolvable PVA (mol wt, 30,000 to 70,000; Sigma-Aldrich) matrix and then flipped upside-down to undergo a second step of laser ablation. This process was used to extricate the MNs from the rest of the steel block. The array of MN electrodes was then integrated onto a laser-patterned Au planar MEA through a room temperature ACF tape (RF EMI shielding tape 9703, 3M). To mechanically fix the MNs and avoid any undesired sliding, bending, or breakage, a thin layer of biocompatible silicone (Dowsil, 734) was injected in the empty spaces among the electrodes and let cure. After that, electrical connections were ensured through flexible flat cables (RS Components) attached to the device’s pads using a heat bonding machine (3T Frontiers Pte. Ltd.) and a high-temperature ACF tape (AC-7813KM-25, Hitachi Chemical). Next, a 5-μm parylene C deposition was carried out using the PDS 2010 Labcoter 3 system (SCS). To expose the tip of each parylene-encapsulated MN, a two-step process was performed. First, a mild laser ablation (d-3DPLM) was performed over the envelope area tangential to the tips of the 3D electrodes and defocused by 10 μm above the tips. Second, a fs pulsed laser system (Laser Conversion Carbide CB5-06-0100-10-H laser with second harmonic CBM04-2H; wavelength: 515 nm; pulse duration: 290 fs; frequency range: single shot to 1 MHz; maximum average power: 3 W at 60 kHz; maximum pulse energy: 0.05 mJ at 60 kHz; Oxford Lasers) was used to polish and outline accurately the electrode’s tips, focusing the laser beam on each tip and ablating a 20-μm-diameter projected area. This second step was necessary to achieve the desired size of the exposed electrode area, which was not possible with the bigger beam spot (30 μm in diameter) of the ns laser system. In addition, it created a smoother morphology for the exposed area which enabled a low-noise detection of neural signals. The array was lastly integrated within a PDMS fluidic well, as illustrated in fig. S59 (step 12). The well also had an inlet and outlet to enable the integration of the device into a system for recirculating the culture medium. The complete device comprised the 3D electrode array, the fluidic well, and the electrical connections.

### Characterization of tunable-height 3D MEAs for in vitro recording of neural organoid activity

#### Optical and SEM

The 3D MN electrodes were observed through 3D optical microscopy (Keyence VHX-7000 Digital Microscope) and through a scanning electron microscope (Zeiss Sigma 300 FEG-SEM). The heights, tip diameters, and base diameters of each MN were measured optically using the 3D mapping functionality of the microscope. Energy dispersive x-ray (EDX) analysis was performed to assess the material recasting and resolidification during laser ablation: The SEM microscope was equipped with Oxford Inst. EDX detector. The EDX scanning was performed with an accelerating voltage of 10 kV.

#### Electrochemical characterization

All measurements (for planar and 3D MEAs) were performed in PBS (0.01 M, Sigma-Aldrich) in a three-electrode setup with an Ag/AgCl reference electrode (Mettler Toledo) and a Platinum Iridium (PtIr) gauze, 150 mesh woven from 0.043-mm DIA wire (Thermo Scientific Chemicals), as the counter electrode. The EIS was recorded with an impedance analyzer (PalmSens4 potentiostat/galvanostat, Alvatek) with PSTrace 5.9 data analysis software, for frequencies between 1 Hz and 1 MHz, with a driving ac voltage amplitude of 0.1 V, a stabilization time of 200 s. CV characterization was performed with the same instrument and using the same three-electrode setup. The measurements were carried out using a 0.1 V s^−1^ sweep rate and 10-mV step within a window of −1.0 to 1.0 V, 0.0 to 1.0 V, or −1.0 to 0.0 V. A minimum of 10 cycles were performed for each measurement to allow the recording to stabilize, and only the final recorded cycle was analyzed.

#### Mechanical characterization of the 3D MEAs

The mechanical bending stiffness of the 3D MN electrodes was measured using a force displacement machine (Mark-10 Test Frame F-105) with a load cell of 500 N. For these measurements, isolated MNs were produced through laser ablation on stainless steel square blocks of 1 cm by 1 cm by 5 mm. Each block was then fixed on the bottom plate of the mechanical system. The MN was positioned perpendicularly to the loading axis and then pressed with the upper movable clamp of the system. The mechanical stiffness was measured after integrated the 3D MN electrodes into the MEA, to assess the adhesion of the ACF tape.

#### Human pluripotent stem cell culture and genome editing

H9 (chromosomically XX) human embryonic stem cells (obtained from WiCell and approved for use in this project by the UK Stem Cell Bank Steering Committee) were maintained in StemFlex (Thermo Fisher Scientific, catalog no. A3349401) on Matrigel (Corning, catalog no. 354230) coated plates and passaged twice a week using EDTA. Unless otherwise specified, organoids and ALI-COs shown were generated from H9 cells.

#### Generation of cerebral organoids

Human brain organoids were provided by the MRC Laboratory of Molecular Biology and cultured according to the enCOR method, as previously described (*68*, *69*). Briefly, 18,000 cells were plated with PLGA microfilaments prepared from Vicryl sutures, and the protocol for cerebral organoids generation described in (*68*, *69*) was followed. For the organoid culture, the set of media mentioned in (*69*) was used. From day 35 to 40 onward, the medium was supplemented with 2% dissolved Matrigel basement membrane (Corning, catalog no. 354234) to achieve establishment of the cortical plate. At day 55, the organoids were processed for ALI-CO culture or were allow to grow in 3D for 6 months.

#### ALI-CO culture

ALI-CO cultures were prepared using a method presented previously (*68*, *70*). Mature organoids (55 days old) were collected using a cut plastic P1000 pipette tip, washed in Hanks’ balanced salt solution (HBSS) without Ca^2+^ and Mg^2+^ (Thermo Fisher Scientific, catalog no. 14175095), and embedded in 3% low-gelling temperature agarose (Sigma-Aldrich, catalog no. A9414) at ~40°C in peel-a-way embedding molds (Sigma-Aldrich, catalog no. E6032), typically including four to six organoids per mold. Agarose blocks were incubated on ice for 10 to 15 min and processed on a Leica VT1000S vibrating microtome in cold HBSS. Sections (300-μm thick) were collected onto Millicell-CM cell culture inserts (Millipore, catalog no. PICM0RG50) in six-well plates and left to equilibrate for 1 hour at 37°C in serum-supplemented slice culture medium (SSSCM): Dulbecco’s modified Eagle’s medium (Invitrogen, catalog no. 10566016), 10% fetal bovine serum, and 0.5% (w/v) glucose, supplemented with penicillin-streptomycin and Fungizone. SSSCM was then replaced with serum-free slice culture medium (SFSCM): neurobasal (Invitrogen, catalog no. 21103049), 1:50 (v/v) B-27 supplement (Invitrogen, catalog no. 17504044), 0.5% (w/v) glucose, and 1 × (v/v) GlutaMAX supplemented with antibiotic-antimycotic (Thermo Fisher Scientific, catalog no. 15240062). ALI-CO cultures were maintained in SFSCM at 37°C and 5% CO_2_ with daily media changes. Media was provided only below the filter insert so that sections stayed at the ALI and were not submerged.

#### Insertion of cerebral organoids into the MEAs

Once formed, the cerebral organoids were kept in the set of media described in (*69*) at 37°C with 5% CO_2_ under agitation. Before the insertion of the organoid, the 3D MEAs were sterilized by UV light illumination and spraying with ethanol for 30 min. Human brain organoids were collected from the culture wells by gently pipetting them along with <200-μl volume of organoid media and were subsequently released from the pipette at the center of the dry 3D MEAs. Further culture media (to reach 200 μl) at 37°C was added to fill the fluidic well of the device after the insertion of the organoid. The organoids were left inside the fluidic well of the MEAs for 10 min, to allow a smooth and slow settling and adaptation of the biological tissue to the array’s topography. Before starting the electrophysiological recordings, the 3D MEAs with the seeded organoid were transferred to an incubator and maintained at 37°C with 5% CO_2_ for at least 10 min to ensure environment stabilization. The electrophysiological measurements took ~30 min, during which time the organoids were kept on the MEAs in the incubator. Therefore, the overall time for the organoids to settle on the MEAs was at least 50 min.

#### Electrophysiological data acquisition

To electrically characterize the devices, an Intan 128-channel RHS Stimulation/Recording system was used with IntanRHX v3.0.4. software. The 3D MEA devices were connected to the system with a 32-channel RHS stim/record head stage (Intan) with a custom PCB. To ensure the functionality of the electrodes, impedance was detected at 1 kHz, with the electrode arrays soaked in PBS solution with an Au reference electrode, before and after the electrophysiological measurements with the organoids. Electrophysiology recordings were acquired in the same setup enclosed in the incubator, acting as a grounded Faraday cage, and carried out at a sampling rate of 30 kHz, followed by filtering using a notch filter at 50 Hz and a 0.5- to 300-Hz bandpass filter.

Neuronal field potentials were detected for each channel using a threshold-based peak detection method, with the threshold set to be 6σ_n_ away from the mean of the filtered signal. As in previous works (*67*, *71*), a robust estimation of the average noise level of each channel was obtained through σ*_n_* = median [|*S*(*t*)|/0.6745], where |*S*(*t*)| represents the absolute amplitude of the filtered signal (*12*). Peaks less than 2 ms away were discarded, as well as peaks detected synchronously by more than one electrode. Detected events were isolated by windows of 6 ms.

#### Immunohistochemical and analysis and microscopy

Organoids and ALI-COs were fixed in 4% paraformaldehyde (PFA) for 20 min at room temperature or overnight at 4°C and washed three times in PBS. For cryostat processing, samples were incubated overnight in 30% sucrose. Embedding, cryosectioning, and staining were performed as previously described (*72*) on whole organoids or ALI-COs after removal from the cell culture insert by cutting the filter around the ALI-CO. For higher-magnification staining, cryosectioning of ALI-COs was necessary due to their thickness (>300 μm). Whole ALI-COs were stained using a modified protocol. All staining steps were done in permeabilization buffer (0.25% Triton X and 4% normal donkey serum in PBS) at 4°C, and their duration was extended as follows: permeabilization, overnight; primary and secondary antibody incubation, 2 days; and wash steps (3×), 8 hours each. Primary antibodies used with corresponding dilutions were as follows: mouse anti-TUBB3 (1:500; BioLegend, 801202) and rabbit anti–glial fibrillary acidic protein (1:200; Abcam, ab7260). All primary antibodies are well described and validated for immunohistochemistry and were checked for compatibility for human tissue on the basis of similarity to the human sequence. No novel antibodies were generated in this study. Alexa Fluor 405–, 488–, 568–, and 647–labeled secondary antibodies (Thermo Fisher Scientific and Abcam) were used for detection. A 15-min staining step after incubation with the secondary antibody with 4′,6-diamidino-2-phenylindole was used to stain cell nuclei. Organoids or ALICOs were then imaged on a Nikon Ti2-W1 spinning disk confocal microscope.

#### Scanning electron microscopy

The organoids were also observed through 3D optical microscopy (Keyence VHX-7000 Digital Microscope) and through a scanning electron microscope (Zeiss Sigma 300 FEG-SEM). Before imaging, the organoids were prepared following a protocol of chemical drying and critical point drying. Specifically, the organoids were removed from the culture medium and soaked in 4% PFA for chemical fixation. The samples were washed in 0.1 M Pipes buffer (pH 7.4) for three times, incubating for 5 to 10 min between each wash. Then, they were incubated in 1% OsO_4_ in 0.1 M buffer at 4°C for ~1.5 hours. The samples were washed in MilliQ water for three times, incubating for 5 to 10 min between each wash. Afterward, they were dehydrated in 30% ethanol (15 min), 50% ethanol (15 min), and 70% ethanol overnight at 4°C. The following day, the samples were further dehydrated in 80% ethanol (15 min), 90% ethanol (15 min), 95% ethanol (15 min), and 100% ethanol (30 min). Next, ethanol was removed and replaced by 1 ml of hexamethyldisilazane. After that, a Critical Point Drying machine (Tousimis Autosamdri-815) was used to flush the ethanol from the tissue with liquid CO_2_ and dry it without surface tension artifacts. The tissues were lastly coated with 7-nm Au for the SEM imaging.

### Statistical analysis

All statistical analyses were performed using appropriate parametric or nonparametric tests, depending on data distribution, as assessed by the Shapiro-Wilk test. Data are presented as mean ± SD or median with interquartile range where applicable. A significance threshold of *P* value (*P* < 0.05) was applied.

## Supplementary Materials

### This PDF file includes:

Supplementary Notes S1 to S10 Tables S1 to S10 Figs. S1 to S79 References
