# Supplementary material for: Dynamically autofocused 3D pulsed laser micromachining enables advanced 3D bioelectronics
Source: Sci Adv. 2025 Nov 7;11(45):eadz4084. doi: 10.1126/sciadv.adz4084 (PMC12594197; doi:10.1126/sciadv.adz4084)
Supplement: Supplementary file 1 — Supplementary Notes S1 to S10 Tables S1 to S10 Figs. S1 to S79 References [file sciadv.adz4084_sm.pdf]

Science Advances
AAAS

Supplementary Materials for

Dynamically autofocused 3D pulsed laser micromachining enables advanced 3D bioelectronics

Massimo Mariello *et al.*

Corresponding author: Christopher M. Proctor, christopher.proctor@eng.ox.ac.uk

Sci. Adv. **11**, eadz4084 (2025)
DOI: 10.1126/sciadv.adz4084

This PDF file includes:

Supplementary Notes S1 to S10
Tables S1 to S10
Figs. S1 to S79
References

Supplementary Note 1

<u>Choice of laser wavelength in laser micromachining materials for bioelectronics</u>

When selecting laser wavelength (λ) for micromachining bioelectronic materials, the dominant considerations are optical absorption, thermal diffusion/heat affected zone (HAZ), and achievable feature size, all of which vary strongly with λ and material class. We provide here a high-level analysis of the trade-offs associated with different wavelengths (e.g., UV, visible, IR) in terms of absorption, thermal effects, and achievable resolution for the target materials.

Short-wavelength UV (~100–400 nm) couples efficiently to most polymers (π–π* and n–σ* transitions), enabling "cold" ablation with minimal collateral heating and a smaller diffraction-limited spot, which favors sub-micron fidelity on films such as parylene C, polyimide, and cellulose-based composites; the trade-offs are lower pulse energy/throughput, more complex/fragile optics, and ozone/lens-fouling concerns.

Visible (400-700 nm) provides intermediate absorption and simpler beam delivery, useful for materials semi-transparent in NIR yet less UV-responsive.

NIR (e.g., 1064 nm) generally penetrates deeper and is weakly absorbed by many pristine polymers (PDMS, parylene C), increasing reliance on heat accumulation and raising the risk of larger HAZ and edge taper, but it benefits from robust, high-power sources and fast galvo scanning—attractive for prototyping and industrial translation.

For metals, thin Ti/Au and Cu films exhibit higher absorption at shorter wavelengths, improving edge quality and reducing redeposition; stainless steel is more forgiving but still shows cleaner kerfs under shorter-λ exposure. Composites or transparent polymers can be tailored for NIR by adding absorbers (e.g., carbon black, dyes, nanoparticles), and wavelength choice should be co-optimized with pulse duration and scan strategy: shorter λ reduces spot size and fluence threshold, whereas NIR ns systems, like the one used here, can deliver practical speed and alignment robustness while maintaining sufficient fidelity via pulsed operation, hatch optimization, and repetition control.

The following table covers laser wavelength ranges, suitable materials (based on absorption characteristics), typical laser types (including pulse duration and source), and the key advantages and disadvantages of each range for micromachining applications, based on previous literature(*73–81*).

Table S1. Laser wavelength ranges, suitable materials, typical laser types, advantages and disadvantages.

Wavelength range	Suitable materials (based on optical absorption)	Laser Sources (typical pulses)	Advantages	Disadvantages
UV: 100–400 nm	Polymers, plastics, ceramics, glasses, semiconductors. Generally strong UV absorption allows direct photoablation. UV photons break molecular bonds (cold ablation).	• Excimer gas lasers (ArF 193, KrF 248 nm; pulses ≈ 10–30 ns)<br>• Diode-pump solid-state (DPSS) Nd:YAG 4th-3rd harmonic lasers (266, 355 nm; ns pulses);<br>• UV ultrafast lasers (e.g., ~200–400 nm, fs pulses).	• Very small focus spot (diffraction-limited)<br>• Minimal HAZ (cold photoablation yields clean edges)<br>• High precision on delicate/transparent materials	• Limited average power (esp. <300 nm) – low throughput in thick materials<br>• High system cost and maintenance (excimer gas replenishment, UV-grade optics degrade)
Visible: 400–700 nm	Many materials have moderate absorption (metals, polymers, certain glasses). Visible light is absorbed better than IR in some metals (e.g., Cu) and dyes.	• Frequency-doubled solid-state lasers (e.g. Nd:YAG/Nd:YVO_4 at 532 nm, Q-switched ~5–20 ns or CW)<br>• Frequency-doubled fiber lasers (515 nm); visible diode lasers.<br>• Picosecond/femtosecond visible (via OPO).	• Smaller spot size than IR (higher resolution)<br>• Good for metals and color-sensitive materials (e.g., green is well absorbed by copper)<br>• Higher repetition rates possible (up to MHz).	• Still relies on thermal ablation (some HAZ) – less "cold" than UV<br>• Lower output power than fundamental IR (harmonic conversion losses)<br>• Requires frequency-doubling optics (expensive)<br>• Moderate absorption: not as universal as UV
NIR: 700–1200 nm	Metals (stainless steel, Cu, Al), silicon, many ceramics. Most metals absorb well at 1μm; some transparent materials (glass, polymer) cannot be ablated at high frequency (> 100 kHz) but may be partially absorptive at lower frequencies or if doped/coated.	• Nd:YAG lasers (1064 nm; CW, Q-switched ns, or mode-locked ps/fs);<br>• Fiber (Yb-doped ~1070 nm; CW or pulsed ns/ps/fetch)<br>• Diode lasers (808–980 nm CW/long pulse)<br>• Ultrafast Ti:sapphire (800 nm fs).	• Very high power availability (multi-kW CW)<br>• Excellent beam quality (diffraction-limited) and very small focus (larger than visible/UV)<br>• Efficient metal cutting (fibers "couple extremely well" to metals)<br>• Pulsed options span ns–fs (enable cold ablation if ultrashort)	• Thermal HAZ if using long pulses (>ns or CW)<br>• Less effective on transparent materials (glass transmits NIR) or pure organics (no inherent absorption)<br>• Fiber lasers: might struggle with thick or organic substrates (e.g., wood, acrylic)
Mid-IR: 1200–5000 nm	Water and biological tissue (strong H_2O absorption), bone, many polymers (C–H, O–H stretch bands around 2–3 µm), silica glass (OH absorption).	• Er:YAG lasers (2940 nm; typically µs–ms pulses or Q-switched ns)<br>• Thulium/holmium fiber lasers (1900–2100 nm; CW or pulsed)<br>• CO lasers (5.3 µm).	• High absorption in water/tissue: precise ablation of biological/organic materials<br>• Minimal penetration depth (ultra-localized removal, useful in medical/dental micromachining)<br>• Can machine materials (e.g., silicon, oxide films) with specialized mid-IR systems.	• Specialized optics/fibers needed (silica is opaque >2 µm), using fluoride, chalcogenide or hollow fibers<br>• Lower available average power (many mid-IR lasers are flashlamp-pumped): slower ablation<br>• Often large pulses (ns–µs) lead to thermal effects unless fs modes are used<br>• Expensive and complex (Er:YAG crystals, cooling systems, etc.)
Far-IR: >5000 nm	Organic materials (wood, paper, fabrics, leather, biological tissue) -C-H and C-O bonds absorb strongly; many polymers (PMMA, ABS, polyimide, etc.); glasses with OH groups.	• CO_2 lasers (10.6 µm; high-power CW or pulsed ~ms)<br>• CO lasers (5.3 µm)<br>• Emerging quantum-cascade lasers (mid-to-far IR, CW).	• Versatile on organics and plastics: excels at cutting/etching wood, acrylic, leather, etc.<br>• High continuous power; can cut thick materials with smooth edges<br>• Mature, robust technology (reliable multi-kW lasers)	• Inefficient (gas laser; high energy loss)<br>• Poor on reflective metals (most metals reflect/absorb minimally at 10 µm)<br>• Large spot size (diffraction limits resolution), thus lower precision<br>• Bulky, requires gas handling and mirror replacement; significant thermal HAZ

Supplementary Note 2

<u>Analytical model of the intensity distribution of a NIR ns-pulsed Laser</u>

A simple, empirical model assumes that the intensity (and optical power density) distribution of a NIR ns-pulsed laser follows a Gaussian profile (Fig. S2(A)), mathematically expressed as

$$p(r) = p_{max} \exp(-r^2/(2\sigma^2)) \quad \text{(S1)}$$

where σ is the standard deviation of the function, correlated with the beam diameter d_0; p_{max} is the peak intensity at the center; r is the radial distance from the beam axis. The width of the laser beam varies along the z axis above the focal spot as follows:

$$w(z) = w_0 \sqrt{1 + \left(\frac{\lambda z}{\pi w_0^2}\right)^2} \quad \text{(S2)}$$

where $w_0 = 2\sqrt{2 \ln 2}\,\sigma = 24.976\mu m$ is the full-width at half-maximum (FWHM), i.e., the actual laser beam diameter on the focal spot. The beam diameter ($d_0 = 30\mu m$) is considered at the point where the intensity drops at $1/e^2$ of its peak value.

The maximum material removal per pulse (D_{max}) occurs at the central region of the beam spot, where the fluence is highest. The depth of ablation increases with the pulse energy E, where $E = P_{avg}/f$ (where f is the pulse repetition rate), and is influenced by material properties such as thermal conductivity (k), heat capacity (C_p), density (ρ), vaporization temperature (T_v), and latent heat of vaporization (H_v), as well as laser parameters including wavelength (λ), pulse duration (τ), peak fluence (F), and repetition rate.

In particular, heat capacity can depend not only on the bulk properties of a material but also on factors such as geometry, layer thickness, microstructure, and interfacial thermal conductance, all of which influence heat distribution during laser exposure. In our model, Cp was treated as a bulk, material-specific parameter, using literature values under standard conditions. This quasi-bulk approximation is justified by the nanosecond pulse regime employed here, where the thermal diffusion time across individual layers is comparable to or longer than the pulse duration, making bulk-like Cp a reasonable first-order estimate of transient thermal behavior. While Cp itself was held constant, the finite element model explicitly incorporated layer thickness and geometry, allowing depth-resolved gradients and local hot spots to emerge. Importantly, model predictions of ablation thresholds for stainless steel across different geometries agreed with experimental measurements within 5–10%, supporting the validity of this assumption in the present regime. Nonetheless, we acknowledge that in ultrathin films, multilayers, or nanostructured materials, Cp may deviate significantly from bulk values, and future modeling could account for spatially varying Cp or couple to phase-change dynamics to more accurately capture such effects.

Empirical fitting from experimental data (Fig. S11) yields a power-law relation,

$$D_{max} = \alpha F^{\beta} \quad \text{(S3)}$$

where α and β depend on the material and the duration of the ablation (e.g., $\alpha = 19.08\ \mu m\, J^{-1/\beta} cm^{-2/\beta}$ and $\beta = 0.50$ for stainless steel singe-spot-ablated in pulsed mode for 1s; $\alpha = 77.98\ \mu m\, J^{-1/\beta} cm^{-2/\beta}$ and $\beta = 0.48$ for stainless steel single-spot-ablated in pulsed mode for 10s). This experiment cannot be quantified accurately for Ti/Au thin films.

Ablation by multiple laser pulses follows a cumulative process, where the total removed depth is the sum of single-pulse ablation contributions. A similar relation can be established between the

depth and the cumulative laser fluence and for instance, $\alpha = 6.86 \cdot 10^{-11}$ $\mu m\, J^{-1/\beta} cm^{-2/\beta}$ and $\beta = 1.82$ for stainless steel single-spot-ablated in pulsed mode for 10s. However, material redeposition, heat accumulation, and variations in optical penetration due to surface roughness and oxidation can alter the ablation efficiency over successive pulses.

The radial variation of ablation depth follows the intensity profile of the laser beam and is well approximated by a Gaussian function:

$$D(r) = D_{max} \exp(-r^2/k^2) \quad \text{(S4)}$$

Where k is an empirical parameter that characterizes the thickness profile, typically found to be $D_{max} = 58.4\mu m, k \approx 12.4\ \mu m$, for single-spot ablation at average power 30%, pulse frequency 100 kHz, and duration 10s (Fig. S12).

Theoretical models describing the laser-material interaction predict a transition between different removal regimes, such as thermal melting, vaporization, and plasma-induced ablation, depending on fluence and pulse duration. At nanosecond pulse durations, compared to shorter pulses (ps, fs), thermal diffusion effects can play a significant role, as heat conduction spreads energy beyond the focal spot, potentially affecting neighboring regions. Fig. S2(B) shows other key parameters related to the temporal distribution of the laser pulses, such as pulse duration, peak power, average power, period.

Supplementary Note 3

Computational model of the ablation process with a NIR ns-pulsed laser

When a solid material is heated, its temperature increases until it undergoes a phase transition. This transition may involve melting into a liquid before vaporizing into a gas or, in some cases, directly transitioning from solid to gas. Here, we focus solely on materials that undergo direct sublimation. We assume that heating occurs primarily at the surface, with no internal heating that could create gas-filled voids within the solid. This ensures that sublimation is confined to the material's surface. Additionally, once the material transitions to the gas phase, it is no longer considered thermally significant, provided that an external gas flow efficiently removes the vaporized material. This process of heating a material's surface until it vaporizes and swiftly removing the resulting gas from the vicinity of the solid is referred to as ablation.
A computational model of laser ablation and heat transfer was developed using COMSOL Multiphysics to analyze the thermal effects involved in laser-material interactions with a NIR ns-pulsed laser. The model is based on solving the heat conduction equation,

$$\frac{\partial T}{\partial t} = \alpha \nabla^2 T + \frac{Q}{\rho c} \tag{S5}$$

where T is the temperature, α is the thermal diffusivity, Q is the heat source term representing laser energy absorption, ρ is the material density, c is the specific heat capacity.
The heat source term is modeled using a Gaussian spatial distribution to represent the laser beam intensity profile, and the laser energy absorption follows the Beer-Lambert law. The model accounts for heat transfer and phase transitions, such as melting and sublimation. The material removal process is simulated by implementing a moving boundary condition, where elements exceeding the vaporization temperature are progressively removed, mimicking the ablation process.
For a nanosecond-pulsed NIR laser, thermal diffusion plays a significant role in the energy transfer process, influencing the ablation efficiency and collateral heating effects. The thermal diffusion length (δ_T) represents the characteristic distance over which heat spreads during the laser pulse duration and is given by:

$$\delta_T = \sqrt{\alpha\tau} = \sqrt{\frac{k}{\rho c_p}\tau} \tag{S6}$$

where α is the thermal diffusivity of the material, τ is the laser pulse duration, k is the material thermal conductivity, ρ is the material density, c_p is the specific heat capacity at constant pressure. For ns pulses (τ ~1-100 ns), the thermal diffusion length typically falls within the range of several hundred nanometers to a few microns, depending on the material. In the following table we summarize these values for the materials mentioned in this study.

Table S2. Values of thermal diffusivity and thermal diffusion lengths.

**Material**	$\boldsymbol{\alpha\ (10^{-6} m^2 s^{-1})}$	$\boldsymbol{\delta_T\ (nm)}$ **at** $\boldsymbol{\tau = 1\ ns}$	$\boldsymbol{\delta_T\ (nm)}$ **at** $\boldsymbol{\tau = 10\ ns}$	$\boldsymbol{\delta_T\ (nm)}$ **at** $\boldsymbol{\tau = 100\ ns}$
Stainless steel	40 (*82*)	200	632	2000
Au	127	357	1127	3563
Cu	111 (*83*)	333	1052	3326
Parylene C	0.12 (*84*)	11	38	120

The thermal diffusion length is thus generally larger than that observed for ultrafast pulsed lasers (*6*), due to the longer pulse duration. In fact, for picosecond pulses (1-10 ps), $\delta_T \sim 10 - 100\ nm$, whereas for femtosecond pulses (10-100 fs), $\delta_T \sim 1 - 10\ nm$. This means that heat conduction extends beyond the directly irradiated zone, leading to a larger heat-affected zone (HAZ) compared

to shorter pulse durations (<1 ns). However, the thermal diffusion length is still lower or at most comparable to the lateral patterning resolution (~10 μm). Therefore, for a ns-pulsed NIR laser, heat diffusion occurs within the pulse duration, leading to some melting and recast layer formation around the ablation crater (as it is seen with the formation of microneedles). The following table summarizes these concepts for a ns-pulsed laser in comparison with other pulse durations.

Table S3. Comparison between laser pulse durations.

Pulse duration	τ	δ_T	Effects
Microsecond (μs)	1-100 μs	1-10 μm	Significant heat diffusion, leading to melting, heat accumulation, and larger HAZ rather than precise material removal.
Nanosecond (ns)	1-100 ns	100 nm – 10 μm	Thermal diffusion occurs within the pulse duration, leading to melt formation and residual heat effects, although confined within the lateral patterning resolution.
Picosecond (ps)	1-10 ps	10-100 nm	Heat conduction is significantly reduced, resulting in less thermal damage and minimal recast layers. Ablation occurs primarily through plasma formation and photomechanical effects.
Femtosecond (fs)	10-100 fs	1-10 nm	The pulse duration is shorter than the electron-phonon relaxation time (~1–10 ps). This means the electrons absorb laser energy, but the lattice remains cold during the pulse, leading to nonthermal ablation. Energy deposition happens much faster than thermal diffusion, leading to ultrafast ionization and Coulomb explosion rather than heat-driven material removal. The process is almost entirely athermal, eliminating heat-affected zones and enabling high-precision material structuring with minimal damage.
Attosecond (as)	1-1000 as	1-10 fm	The laser-matter interaction occurs primarily via strong-field physics and electron excitation dynamics rather than thermal diffusion. Thermal diffusion is negligible in this regime, as heat transfer is orders of magnitude slower than the interaction time. Instead of thermal ablation, attosecond pulses induce direct electron ejection, extreme ultraviolet (XUV) interactions, and high-harmonic generation, making them valuable for studying fundamental electronic processes at atomic timescales rather than conventional material processing.

Fig. S3 reports the results of FEM simulations for the temperature distribution induced by a single laser pulse (with beam diameter 30μm) hitting the surface of a stainless-steel block. The distribution follows the Gaussian profile of the laser power density, thus the temperature gradually drops from its maximum (in the center) radially outwards. With the same beam power, different pulse durations (i.e., different pulse energies) induce different values of maximum temperatures (Fig. S3(C, D)): longer pulses contribute to increased heat accumulation. Length scale values with different durations, from 1ns to 1μs, appear in Fig. S3(E) for a single laser pulse on stainless steel. The length scale is computed considering the drop of the maximum temperature by a factor of $1/e^2$. Thus, we obtained the following maximum temperatures and length scales.

Table S4. Maximum temperatures and thermal diffusion lengths extracted from FEM.

Pulse duration	Maximum temperature (˚C)	Thermal diffusion length
1 ns	49.002	~200 nm
5 ns	119.055	~250 nm
10 ns	171.259	~700 nm
100 ns	315.776	~2 μm
500 ns	989.485	~2.5 μm
1 μs	1448.158	~6.5 μm

We should note that the maximum temperatures listed in the table are for an ideal single laser pulse on the free surface of a material block. However, temperature distribution during NIR laser

ablation is highly geometry- and context-dependent, influenced by local curvature, multilayered structures, and the thermal properties of adjacent materials. The simplified 2D axisymmetric model used here provides conservative estimates and highlights material- and fluence-dependent trends but does not capture device-level complexities. In real structures, geometry and layering can alter heat flow, shifting ablation thresholds and collateral effects. We therefore emphasize that our model serves as a baseline framework, while precise predictions in heterogeneous or 3D architectures may require case-specific FEM calibration.

Supplementary Note 4

Occurring physical mechanisms during NIR ns laser ablation

During nanosecond-pulsed NIR laser ablation, the rapid delivery of energy produces steep spatial and temporal thermal gradients, and the dominant removal mechanism depends critically on whether the local temperature exceeds the melting point, boiling point, or sublimation temperature of the material. At the laser focus, finite element modeling (Supplementary Note 3, Fig. S3) shows that peak temperatures (for single pulses) rise within a few nanoseconds to hundreds of ˚C. For ablation processes longer than 1µs, values rise well above the sublimation/melting thresholds of metals such as stainless steel (sublimation ~3200–3300 °C, melting ~1450 °C), gold (sublimation ~2800 °C, melting ~1064 °C), and copper (sublimation ~2800–2900 °C, melting ~1085 °C). This is also true for longer ablation processes in ns pulsed mode, where the heat transferred is the result of the cumulative effects of multiple pulses.
These peak values, combined with the short thermal diffusion length characteristic of ns pulses (hundreds of nm–few µm), indicate that sublimation or explosive ablation dominates in the spot center, since heating is highly localized and energy deposition occurs faster than melt flow or resolidification can take place. This interpretation is consistent with experimental evidence: SEM images (Fig. S4(A)) show sharply defined craters with minimal recast layers or reflow rims, which would be expected if melting and vaporization were prevalent. In contrast, in the periphery of the irradiated zone, where local temperatures do not exceed the sublimation point but may surpass the melting point, partial melting, resolidification, and thermal degradation are likely. Such peripheral effects can be observed in (Fig. S4(B)) and can also occur in multilayered systems or 3D microstructures, where heterogeneous thermal conductivities alter local heat flow and can lead to collateral damage, redeposition, or delamination.
In rare cases, as observed in the ablation of 3D MEA electrodes, localized melting and recast were detected, underscoring that while sublimation is the dominant mechanism at the beam center, a continuum of thermally driven processes—from melting to evaporation—contributes to the overall ablation morphology.
The dominance of a particular ablation mechanism—whether sublimation, melting, or vaporization—depends critically on the laser parameters, including pulse duration, fluence, repetition rate (Fig. S5(A,B)). Shorter pulses and higher peak fluences tend to favor rapid, localized heating that can drive direct sublimation or explosive ablation, minimizing melt formation. Conversely, lower fluences or longer pulses increase the role of thermal diffusion, allowing the material to reach its melting point without immediately vaporizing, which promotes melting, resolidification, or the formation of recast layers. Additionally, the spatial distribution of laser intensity and the cumulative effects of multiple pulses can shift the balance between mechanisms, producing complex gradients where different physical processes coexist across the laser spot and its surrounding heat-affected zone.

Supplementary Note 5

Analysis of 2D NIR ns laser ablation on Parylene C/Ti/Au and Cu foils on PDMS

The ablation of Parylene C/Ti/Au thin films exhibits distinct characteristics compared to stainless steel due to the differences in thermal conductivity, optical absorption, and melting/sublimation behavior. Given the high reflectivity of Au at near-infrared (NIR) wavelengths (*85*), efficient energy absorption relies on the presence of the Ti adhesion layer and the underlying Parylene C substrate. In this case, the ablation is successful as long as the exposed area is totally clear and with no metal residues: this can be achieved by adjusting the laser parameters (Fig. S13). The ablation area increases with power while decreasing with scanning speed and frequency. Notably, lower pulse frequencies at constant power enhance peak fluence, improve ablation efficiency, but also increase the risk of thermal accumulation in the polymeric substrate. In fact, due to the lower thermal conductivity of Parylene C compared to metallic substrates, localized heating effects can lead to polymer degradation. The selected parameter ranges in Fig. S13 were optimized to avoid unwanted delamination or surface damage (under-ablation) in the polymer. Using optimized parameters (average power 30%, scanning speed 1500 mm/s, pulse frequency 100 kHz, repetitions 3), it is possible to achieve different shapes whose edge quality decreases with decreasing the characteristic dimension (Fig. 2(A)). In particular, considering sample circles, squares and serpentines, consistency with the ideal 1:1 ratio between the projected area and the effective post-ablation area is retained with characteristic dimensions down to ~50 µm (Fig. 2(B) and Fig. S14). Scaling down the projected size reduces the in-plane resolution and edge quality (inset in Fig. 2(B)), with minimum detectable pattern sizes of ~100µm (circle diameter) and ~50µm (square side).

Laser ablation of Cu laminate foils (116 µm) on PDMS is strongly influenced by copper's high thermal conductivity and reflectivity, as well as the thermal insulation properties of the underlying PDMS (see the sampling-matrix characterization in Fig. S17-Fig. S18). Compared to stainless steel, the ablation of Cu requires higher fluence to achieve similar depth per pulse due to its superior thermal diffusivity, which dissipates heat rapidly, reducing localized vaporization (*86*). The PDMS substrate, on the other hand, has a significantly lower thermal conductivity, but also a very low NIR absorption (*87*), preventing localized heating effects that may cause undesired melting or decomposition. Resulting optimized parameters for Cu ablation were: average power 80%, scanning speed 200 mm/s, pulse frequency 100 kHz, repetitions 20.

The calculated thermal diffusion lengths for ns-pulse duration are approximately 63-200 nm for stainless steel, 333-1052 nm for Cu and 357-1127 nm for Au thin films on Parylene C (see Supplementary Note 3). These values indicate that heat spreads more efficiently in Cu and Au, leading to a larger affected zone, while stainless steel exhibits more confined thermal effects. The presence of the Parylene C or PDMS substrates further influences heat dissipation, as their lower thermal conductivity limits lateral heat spread, enhancing localized ablation.

Supplementary Note 6

Computational model of the 3D electrical stimulation on the skin

To demonstrate the enhanced perception of the 3D electro-haptic devices, compared to flat electrodes, a computational model was developed in COMSOL Multiphysics. This was used to analyze the electric field distribution and current density of a 3D electrode system designed for electrical stimulation of the skin. The model consists of a conical active electrode and a ring-shaped ground electrode, which together create a focused electric field that enhances stimulation efficiency while minimizing unwanted current spread. The governing equations for the electric potential distribution are derived from Laplace's equation:

$$\nabla \cdot (\sigma \nabla V) = 0 \tag{S7}$$

where σ represents the spatially varying conductivity of the skin and deeper tissue layers.
The electrical conductivity and relative permittivity of the skin and underlying tissue layers (at 100 Hz) are incorporated into the model to account for inhomogeneous current distribution and impedance variations. In particular, the following ranges and selected values were used.

Table S5. Properties of the different skin layers used in the FEM simulations.

Skin layer	Thickness	Electrical conductivity (S/m)	Relative permittivity
Stratum corneum	20 μm	10^{-6}-10^{-5} (sel. value: 5x10^{-6})	1000-2000 (sel. value: 1.5 x10^{3})
Epidermis	1.5 mm	10^{-3}-10^{-2} (sel. value: 5 x10^{-3})	1000-5000 (sel. value: 2.5 x10^{3})
Dermis	4 mm	10^{-2}-10^{-1} (sel. value: 5 x10^{-2})	2000-8000 (sel. value: 5.0 x10^{3})
Inner layer (muscle)	6 mm	0.05-0.15 (sel. value: 1x10^{-1})	5000-10000 (sel. value: 7.5 x10^{3})

Finite element analysis (FEA) is employed to solve for the electric field vectors and current density distribution, providing insights into the penetration depth and spatial selectivity of the stimulation. The conical electrode design allows for higher current density concentration at the apex, which is beneficial for targeted stimulation. Meanwhile, the ring-shaped ground electrode establishes a return path that mitigates current dispersion. Tetrahedral (free triangular in the cross section) mesh and proper refinement techniques ensure numerical accuracy, particularly near the electrode-tissue interface where field gradients are highest.
The primary reason for electro-haptic stimulation on the skin is current density. It works by delivering electrical currents through the skin, activating nerve endings (such as mechanoreceptors or nociceptors) and eliciting a tactile sensation. While voltage (applied potential) is necessary to drive the current and electric field influences the distribution of charge within the tissue, it is the current density that directly determines whether nerve fibers are sufficiently stimulated to produce a sensory response. For effective electro-haptic stimulation, current density must reach a threshold level to depolarize the nerve endings; electric field plays a role in determining how deeply and broadly the current penetrates the tissue.
Therefore, Fig. S36 shows the distribution of electric field and current density with an applied current pulse of 50 mA. The results demonstrate that the conical active electrode with a ring-shaped ground provides superior localization of stimulation effects compared to conventional planar electrodes (Fig. S36). It should be noted that this model does not take into account the skin deformations when it is in contact with the 3D electrodes: in the model the skin is in contact with all the points of the 3D electrodes' surface. Additionally, the model does not take into account (out of scope) the transient response and any undesired capacitive effects owing to the higher-frequency pulses (pulse duration of 200μs, i.e., 5×10^{3} Hz): more detailed simulations would require time-dependent analyses.
These simulations provide a theoretical foundation for optimizing 3D electrode designs in clinical and wearable bioelectronic applications.

Supplementary Note 7

Fundamental theory of inductive coils and wireless power transfer by inductive coupling

Inductive coils are fundamental components in wireless power transfer (WPT) systems, operating based on electromagnetic induction principles. When an alternating current (AC) flows through a primary coil, it generates a time-varying magnetic field, which induces an electromotive force (EMF) in a secondary coil according to Faraday's law of induction. The induced voltage in the secondary coil depends on the mutual inductance between the two coils, which is influenced by parameters such as coil geometry, separation distance, alignment, and the operating frequency of the AC signal. The mutual inductance ("M") is given by $M = k\sqrt{L_1 L_2}$, where k is the coupling coefficient (ranging from 0 to 1), and L_1 and L_2 are the self-inductances of the primary and secondary coils. The coupling coefficient is a key parameter affecting WPT efficiency, with higher values indicating stronger magnetic coupling and reduced energy losses.

The inductance of a coil is determined by its physical characteristics, such as the number of turns (N), coil radius (r), and wire spacing. For a simple air-core solenoid, the inductance is approximated as

$$L = \frac{\mu_0 N^2 A}{l} \tag{S8}$$

where μ_0 is the permeability of free space, A is the cross-sectional area of the coil, and l is the coil length.

For planar spiral coils commonly used in microfabricated wireless systems, Wheeler's formula provides a practical estimation of inductance as

$$L \approx \frac{\mu_0 N^2 r^2}{8r + 11d} \tag{S9}$$

where d is the trace width. The quality factor (Q) of the coil, given by

$$Q = \frac{\omega L}{R} \tag{S10}$$

where ω is the angular frequency and R is the coil resistance, determines how efficiently the coil stores energy relative to its resistive losses. A higher Q factor improves power transfer efficiency by minimizing resistive losses.

Wireless power transfer (WPT) efficiency depends not only on coil design but also on the operating frequency. At lower frequencies, power transfer is governed by near-field magnetic coupling, where the efficiency decreases significantly with distance due to weaker coupling. At higher frequencies, resonant inductive coupling can enhance efficiency by utilizing resonance matching between the transmitter and receiver coils. The resonant frequency (ω_0) is determined by

$$\omega = \frac{1}{\sqrt{LC}} \tag{S11}$$

where C is the capacitance of the resonant circuit. Proper tuning of the resonance condition is crucial for maximizing power transfer efficiency and minimizing energy loss.

The skin effect and proximity effect also influence coil performance, especially at higher frequencies. The skin effect increases the effective resistance of the coil by forcing current to flow near the conductor's surface, while the proximity effect causes non-uniform current distribution in adjacent coil turns, increasing resistive losses. These effects necessitate careful material selection and coil geometry optimization to reduce energy dissipation. Additionally, coil misalignment can significantly degrade coupling efficiency, requiring adaptive tuning or multiple-coil architectures for robust performance.

For our specific setup, to characterize the electromagnetic coupling between the transmitting (TX) and receiving (RX) coils, we first measured their mutual inductance. The coils were connected in series and their equivalent inductance was recorded in two configurations (Fig. S57(A)): positive coupling (same polarity, mutually aiding flux) and negative coupling (reverse polarity, mutually opposing flux). From these measurements, the mutual inductance was extracted according to:

$$M = \frac{L_+ - L_-}{4} \tag{S12}$$

where $L_+ = L_{TX} + L_{RX} + 2M$ and $L_- = L_{TX} + L_{RX} - 2M$ are the equivalent inductances in the positive and negative coupling cases, respectively. For instance, we obtained, at a selected frequency of 2 MHz, for design D2: $L_+^{D_2} = 742.62\, nH, L_-^{D_2} = 622.78\, nH, M^{D_2} = 29.96\, nH$, and for design D3: $L_+^{D_3} = 1103\, nH, L_-^{D_3} = 742.53\, nH, M^{D_3} = 90.12\, nH$. Therefore, with the following measured values of $L_{TX} = 630.23\, nH, L_{D_2} = 6.49\, nH, L_{D_3} = 271.42\, nH$, the coupling coefficients for these designs, at the same frequency, are: $k^{D_2} = 0.47, k^{D_3} = 0.22$.

The input power delivered to the transmitting coil was determined by simultaneously recording the coil voltage $V_{TX}(t)$ and current $I_{TX}(t)$ waveforms (Fig. S57(B)). The coil was driven within the operating frequency range for each design of receiving coil, with the voltage (V_{TX}) across the coil acquired with an oscilloscope voltage probe connected in parallel, whereas the current (I_{TX}) detected through an oscilloscope magnetic flux-based current probe (the detected waveforms are reported in Fig. S57(C)). From the measured waveforms, the root-mean-square (RMS) values ($V_{TX}^{RMS}, I_{TX}^{RMS}$) and phase angle ϕ between voltage and current were extracted. The instantaneous power is given by:

$$p_{TX}(t) = V_{TX}(t) \cdot I_{TX}(t) \tag{S13}$$

and the real (active) input power was calculated as the time average over one period:

$$\overline{p_{TX}} = V_{TX}^{RMS} \cdot I_{TX}^{RMS} \cdot \cos\phi \tag{S14}$$

where $\cos\phi$ accounts for the reactive power contribution. This procedure was repeated across the operating frequency range of each coil design to obtain representative ranges of input power, listed in Table S8. Input electrical powers in the range of ~1 to ~6 W were obtained. It is important to note that the input power measured across the transmitting coil is not an isolated property, but also depends on the presence and characteristics of the receiving coil. When the receiving coil is placed in proximity, the mutual inductance alters the effective impedance seen by the source, modifying both the current amplitude and the phase relationship between voltage and current. As a result, the input power drawn by the transmitting coil reflects not only its own parameters but also the coupling strength and loading conditions of the receiving coil.

Direct experimental measurement of the electrical power in the receiving coil is, in fact, not feasible in our configuration, because the receiving circuit is a closed loop. Any attempt to insert a probe, series resistor, or measurement device would necessarily alter the circuit parameters, changing the resistance, inductance, or even the mutual coupling, and thus invalidate the measurement. Moreover, part of the energy induced in the receiving coil is inevitably dissipated as resistive (Joule) heating within the conductor and surrounding medium. These distributed losses, which would also change when altering the circuit for the sake of measurement, cannot be easily separated from the usable electrical power without perturbing the system. For this reason, the output electrical power was instead estimated indirectly, through the measured coupling coefficient and input power, while acknowledging that this approach may overestimate the real transferable power due to unaccounted dissipation effects.

As reported above, the coupling coefficient was computed as

$$k = \frac{M}{\sqrt{L_{TX} L_{RX}}} \tag{S15}$$

The output power was then inferred from the relation

$$\overline{p_{RX}} \approx k^2 \overline{p_{TX}} \tag{S16}$$

which represents the maximum transferable power under ideal conditions (values listed in Table S8 for the different coil designs, in the range between 0.02 and 0.81 W). Efficiencies ($\eta = \overline{p_{RX}}/\overline{p_{TX}}$) of 3.3%, 4.8%, 12.3% and 22.0% were obtained for the designs D4, D3, D1 and D2, respectively. This approach, however, systematically overestimates the real delivered power, as resistive Joule heating (I^2R losses) in the coils and additional dielectric losses dissipate part of the transmitted energy as heat. Therefore, while coupling-based estimates provide an upper bound for the receiving coil power, the actual usable output will be lower, depending on the specific resistance and geometry of the system. An indirect estimation of the dissipated heat could, in principle, be obtained by correlating the optical power output and operating temperature of the μLED integrated with the receiving coil. Since the difference between the total electrical power delivered to the LED and its emitted optical power is largely dissipated as heat, this approach would provide an experimental handle on power losses within the receiving coil–μLED system. However, such measurements would require a dedicated setup capable of simultaneously and accurately quantifying both the μLED's thermal response and its optical emission under controlled conditions. Establishing this framework is beyond the scope of the present work, but it represents a valuable direction for future studies aimed at a complete energy balance of the wireless power transfer system.

As a final note, to benchmark our approach against conventional resonant coil systems, we carried out measurements comparing non-resonant coils with slightly modified designs incorporating a coupling capacitor for magnetic resonance inductive coupling (Fig. S57(D)). As expected, the resonant coils exhibited a narrower operational voltage range, especially at the resonance frequency, whereas the non-resonant coils displayed a broader but frequency-unlinked and less efficient response. Due to limitations in our instrumentation, which could not apply signals with frequencies higher than 3 MHz, we could not characterize the peak optical power at the coils' self-resonance frequency (which was measured to be beyond 6 MHz), thus we tuned one selected design (D4) at a fixed resonance frequency of ~3 MHz: this was achieved by integrating a coupling capacitor of 2.5 nF. The plots in Fig. S57(D) show that the resonant coils exhibit optical power curves in a narrower voltage range, especially at the resonance frequency, due to the resonance tuning, but across different frequencies lower voltages induce the same levels of optical power as higher voltages in the non-resonant coils. Moreover, the optical power exhibits an increasing trend as a function of the frequency, with the maximum occurring at the resonance.

Comparing other resonant designs with non-resonant counterparts also indicated that at the same operational frequency, but outside resonance, the capacitor-coupled coils were requiring slightly higher current (and therefore higher power) than the ones without capacitors. This can be explained by the impedance of the system:

$$Z(\omega) = R + j \cdot \left(\omega L - \frac{1}{\omega C}\right) \tag{S17}$$

here R is the effective series resistance, L the coil inductance, and C the added capacitance. Unless tuned precisely, the reactive term $(\omega L - 1/\omega C)$ does not cancel, which increases the reactive current component and can lead to higher power drawn from the source without a proportional increase in useful power delivered to the load.

It is well-known in literature that at the resonance frequency ($\omega_0 = 1/\sqrt{LC}$), the input impedance is dominated by R, the reactive power is minimized, and the overall transfer efficiency is maximized. This resonant condition can significantly enhance output power for a fixed operating frequency.

This comparison highlights the trade-off between adaptability and efficiency: resonant coils can achieve higher power transfer but require precise tuning and alignment, while the non-resonant coils maintain greater robustness across frequencies and geometries.

That said, a full investigation of resonance in such coupled coil systems would require addressing additional complexities such as the quality factor of the coils, the effect of coupling coefficients on split resonances, bandwidth constraints, and the system's sensitivity to detuning. Exploring these aspects goes well beyond the scope of the present work, which focuses only on the fabrication of curved coils, regardless if in non-resonant or resonant operation.

Supplementary Note 8

Computational model of the μLED emission on the eye bulb

A computational model was developed in COMSOL Multiphysics to analyze the optical propagation, energy distribution, and thermal effects of the μLED embedded in the soft contact lens, positioned at the center of the cornea, emitting isotropic red light (λ = 635 nm) towards the retina through the body of the eye. The model solves Maxwell's equations for electromagnetic wave propagation using the Helmholtz wave equation:

$$\nabla^2 E + \kappa^2 n^2 E = 0 \qquad \text{(S18)}$$

where E represents the electric field vector, $\kappa = 2\pi/\lambda$ is the wave number, and n is the spatially varying refractive index of the ocular tissues. The refractive indices of the cornea ($n \approx 1.376$), aqueous humor ($n \approx 1.336$), lens ($n \approx 1.410$), and vitreous body ($n \approx 1.336$) were incorporated into the model to account for the refraction, reflection, and absorption of the emitted light. We considered a unique body including the cornea, aqueous and vitreous humor, plus the lens.
The micro-LED emission was modeled as an isotropic point source, emitting light uniformly in all directions. The light propagation was computed using the Radiative Transfer Equation (RTE) for highly scattering media and the Beer-Lambert law for absorption losses:

$$I(r) = I_0 \exp(-\mu_a r) \qquad \text{(S19)}$$

where $I(r)$ is the intensity after traveling distance r, and μ_a is the absorption coefficient of the ocular tissues. In particular, for the cornea $\mu_a \approx 0.2 - 0.5\, cm^{-1}$, for the aqueous humor $\mu_a \approx 0.02 - 0.05\, cm^{-1}$, for the lens $\mu_a \approx 0.3 - 0.6\, cm^{-1}$, and for the vitreous body $\mu_a \approx 0.02 - 0.04\, cm^{-1}$. Light scattering was modeled using the Henyey-Greenstein phase function to approximate the forward scattering behavior in biological media.

In addition to optical modeling, heat transfer simulations were performed to evaluate the thermal effects of μLED operation within the ocular environment. The transient heat conduction equation was solved to compute the temperature distribution in the different eye media:

$$\rho c \frac{\partial T}{\partial t} = k\, \nabla^2 T + \frac{Q}{\rho c} \qquad \text{(S20)}$$

where $T$is the temperature, ρ is the tissue density, c is the specific heat capacity, k is the thermal conductivity, and Q is the heat source term representing heat dissipation from the micro-LED. The thermal conductivities of the cornea ($k \approx 0.58\ W/mK$), aqueous humor ($k \approx 0.58\ W/mK$), lens ($k \approx 0.4\ W/mK$), and vitreous body ($k \approx 0.58\ W/mK$), were included to capture heat diffusion effects.
Simulation results provided insights into the steady-state temperature rise due to the micro-LED operation, ensuring that tissue heating remained within safe limits to prevent thermal damage. The combined optical and thermal modeling framework enables optimization of micro-LED power levels for applications in ocular photostimulation, retinal therapy, and optogenetic modulation while maintaining physiological temperature stability.

Supplementary Note 9

Geometrical considerations for the 3D conical shapes used for the 3D angular resolution study

To study the angular resolution, we used 3D conical shapes with a fixed height H and different radii R. The radiant angle at the cone's apex is twice the angle formed between the cone's axis and its slant height, defined as follows:

$$\tan(\alpha) = \frac{R}{H} \quad \text{(S21)}$$

The full-apex angle is thus given by:

$$\theta = 2\alpha = 2\tan^{-1}\frac{R}{H} \quad \text{(S22)}$$

The solid angle of a cone is given by:

$$\Omega = 2\pi(1 - \cos\theta) \quad \text{(S23)}$$

where

$$\cos\theta = \frac{H}{\sqrt{H^2 + R^2}} \quad \text{(S24)}$$

In our study, we kept the height of the cones constant ($H = 2.5mm$), and we varied the radius. Therefore, in the following table we report the values of radius, full apex angle and solid angles for the surfaces that we used (see Fig. S26).

Table S6. Radius, full apex angle and solid angles for the 3D conical surfaces.

**Radius**	**Full-apex angle (θ, rad)**	**Full-apex angle (θ, deg)**	**Solid angle (Ω, sr)**
2.5	1.5708	90.00	1.8403
2.0	1.3495	77.32	1.3768
1.5	1.0808	61.93	0.8954
1.0	0.7610	43.60	0.4494
0.5	0.3948	22.62	0.1220
0.1	0.0800	4.58	0.0050

Supplementary Note 10

Processing times, yield and automation for NIR laser ablation

The processing performance of the NIR nanosecond laser ablation process was evaluated across the three material systems presented in this work: (i) Ti/Au thin films on parylene C, (ii) Cu foils on PDMS, and (iii) stainless steel substrates. The experiments consisted of ablating circular features with radii ranging from 30 to 2000 μm, using the optimal laser parameters used to pattern the final devices, or systematically varying the laser parameters.
Feature size contributed significantly to processing time: the duration increased with the circle radius, following a mixed quadratic–linear trend. This scaling behavior reflects the combined influence of two geometric factors: (i) the length of the laser scanning path, which scales linearly with radius and dictates the time required for contour passes, and (ii) the ablated area, which scales quadratically with radius and determines the duration of hatch filling. For small features, the contour component dominates, leading to an almost linear relationship between radius and processing time. As feature size increases, the relative contribution of area coverage becomes more significant, producing a stronger-than-linear dependence. The observed trend (Fig. S78) therefore represents a superposition of these two effects, modulated by the chosen hatching strategy and scan speed. This interplay highlights that optimizing path planning, such as reducing redundant passes or adjusting hatch spacing, can yield disproportionate time savings for larger features.
The choice of hatching strategy further influenced throughput. Among the tested strategies, contour hatching, slant hatching, and cross hatching, processing time increased progressively in that sequence, with cross hatching being the most time-intensive due to the higher path density (Fig. S78(A, B); Fig. S79(A)).
Across all three material systems, the pulse repetition frequency was found to have no influence on the total processing time (Fig. S79(B)). With a fixed pulse frequency, processing time scaled linearly with the number of repetitions required to complete a given feature (Fig. S79(C)).
In contrast, the scan speed exhibited a strong inverse relationship with processing duration, with slower scan speeds resulting in longer fabrication times (Fig. S79(D)).
Processing time also scales linearly with the number of distinct designs ablated sequentially in a batch process (Fig. S79(E)) as well as the number of ablation layers for the same design patterned at different z levels (Fig. S79(F)).
Fig. S79(G) reports the specific processing times for ablating the electrode designs used in the devices presented in this work: in particular, the ablation took 182.1 s and 237.1 s for the 1-electrode and 4-electrode electro-haptic patches; 20298.1 s (5.64 h) for the Cu-foil coils in the wireless contact lenses (design D1); 9998.1 s (2.78 h) for one MN electrode 4-mm-diameter array. It should be noted that ablating thin films is much faster than thicker materials where multiple ablation steps are needed to engrave features and ensure optimal resolution.

In terms of yield, the three material systems demonstrated high reproducibility (>98%) for feature sizes above ~100 μm, with minor reductions in yield for smaller radii, primarily due to localized debris or incomplete material removal. Additionally, yields were consistently above 98% for flat and moderately curved substrates.
The reproducibility slightly decreases (~95%) when employing sequential laser ablation across multiple ablation planes or in the case of d-3DPLM. This reduction is primarily attributed to the cumulative effects of thermal loading, beam refocusing tolerances, and debris redeposition between successive layers. In multi-plane ablation, any small deviation in focal alignment or stage positioning can propagate through subsequent layers, resulting in minor dimensional inconsistencies or incomplete removal in localized regions. Similarly, in d-3DPLM, the requirement for precise z-axis control to sculpt varying depths increases the sensitivity of the

process to system calibration, substrate flatness, and material-specific ablation thresholds. While these effects are modest and do not significantly impair overall device functionality, they underscore the importance of fine-tuning the layer-to-layer registration and debris management strategies, such as intermediate cleaning steps or optimized scan path ordering, when targeting high-precision structures.

The process is inherently compatible with automation: once parameters are optimized for a given material-geometry combination, the system can execute multi-pattern jobs with minimal operator intervention, enabling high-throughput prototyping or scaled manufacturing. The linear scaling of time with the number of patterns and repetitions allows straightforward prediction of batch processing durations, a key advantage for process scheduling in automated workflows.

Table S9 lists the processing times and yields for the main ablation patterns, while Table S10 includes the same metrics for the devices used in this work, comprising a quantitative evaluation of the manual interventions for device assembly.

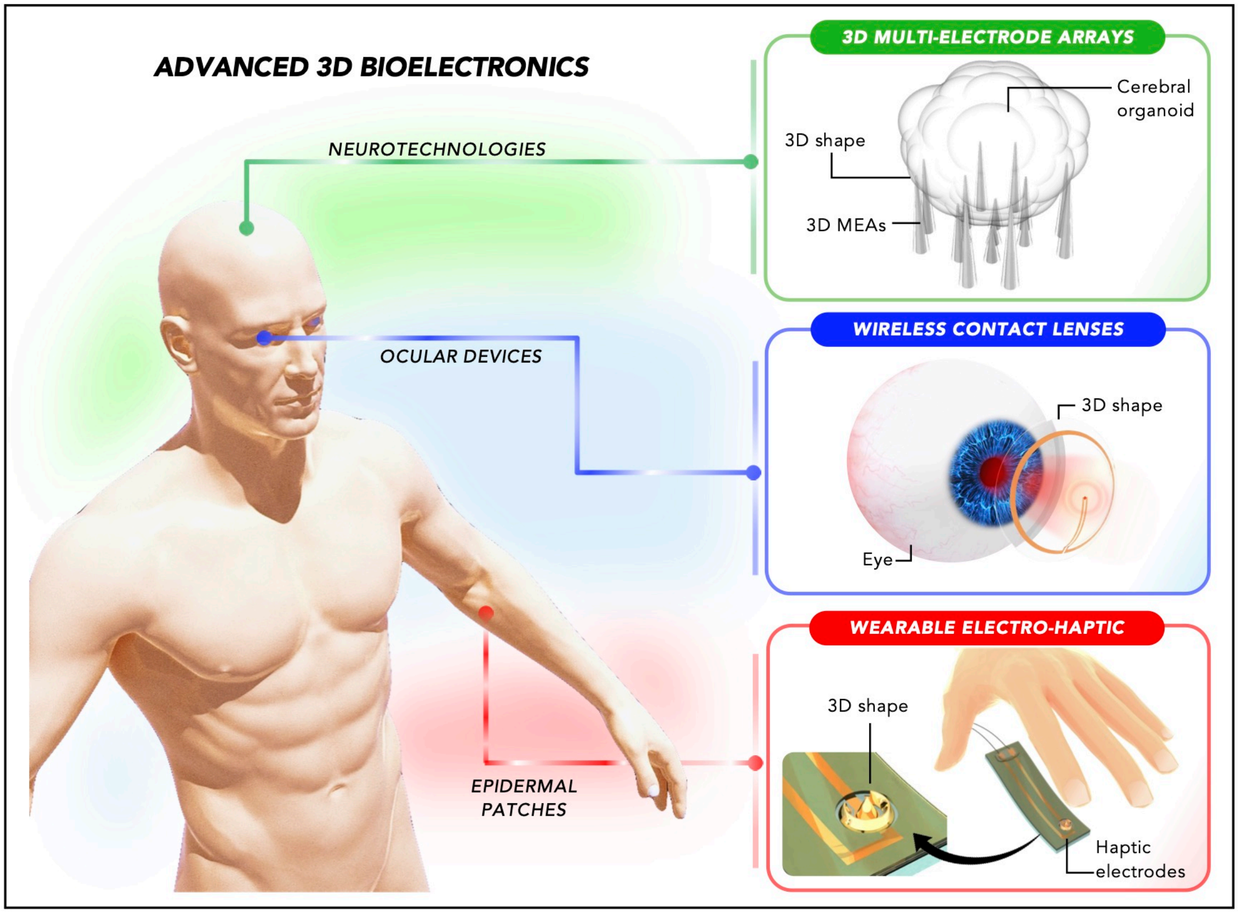

Fig. S1. Applications of the d-3DPLM process presented in this work.

Illustration of the examples of 3D bioelectronics demonstrated in this study: 3D multi-electrode arrays for neural in vitro diagnostics, wireless contact lenses for ocular diseases, wearable electro-haptic patches for tactile stimulation. Illustrations entirely created by authors (software: Blender).

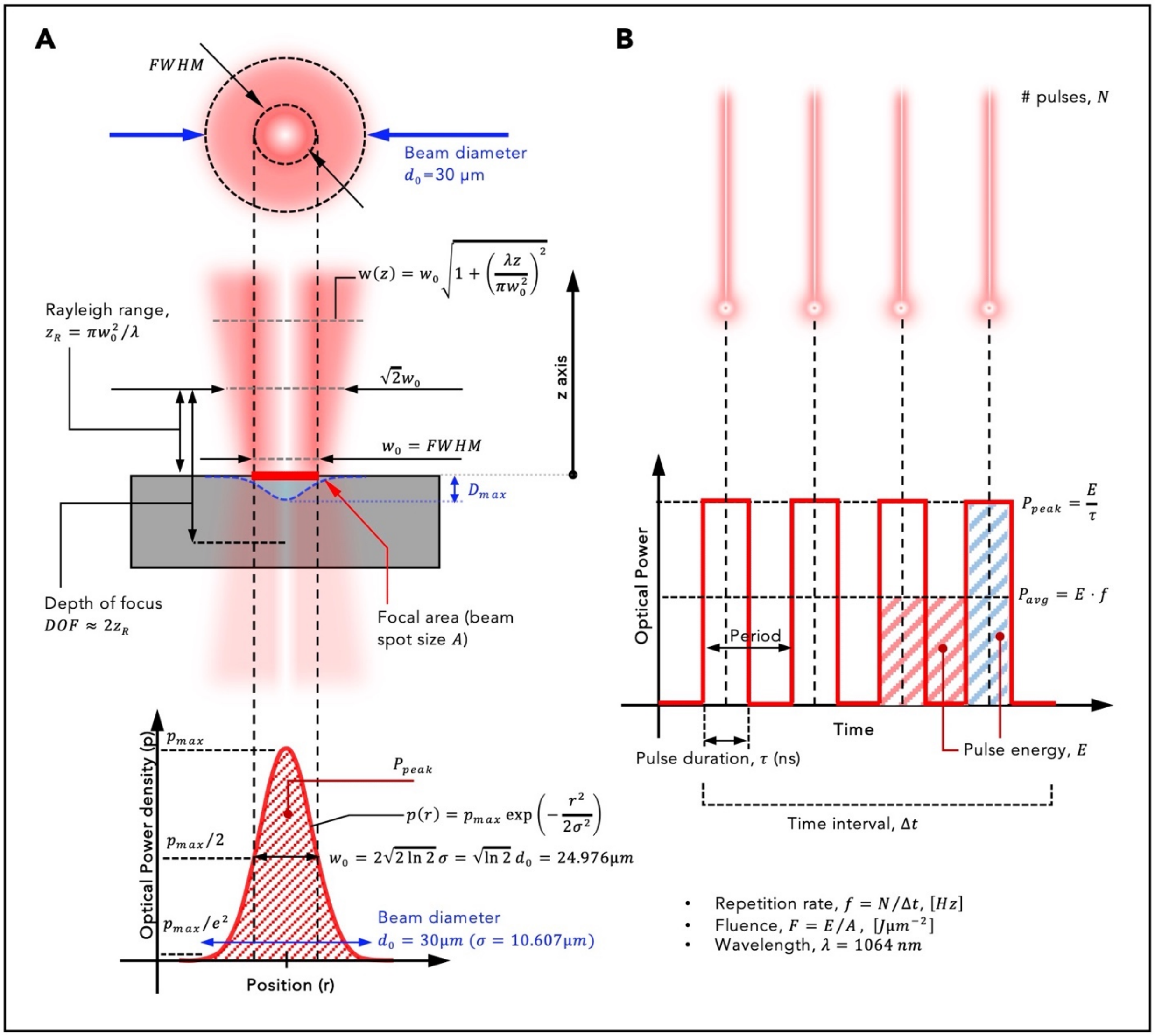

Fig. S2. Schematic of the model of intensity distribution and beam diameter for the NIR ns-pulsed laser.

(**A**) Gaussian distribution of the intensity or optical power density along the radial direction of the laser spot, with indication of beam diameter, FWHM and other key parameters to uniquely identify the laser beam. The profile of ablated thickness is also illustrated with indication of the maximum depth D_{max}. (**B**) Temporal distribution of several consecutive laser pulses with indication of pulse energy, pulse duration, peak and average power and other key parameters.

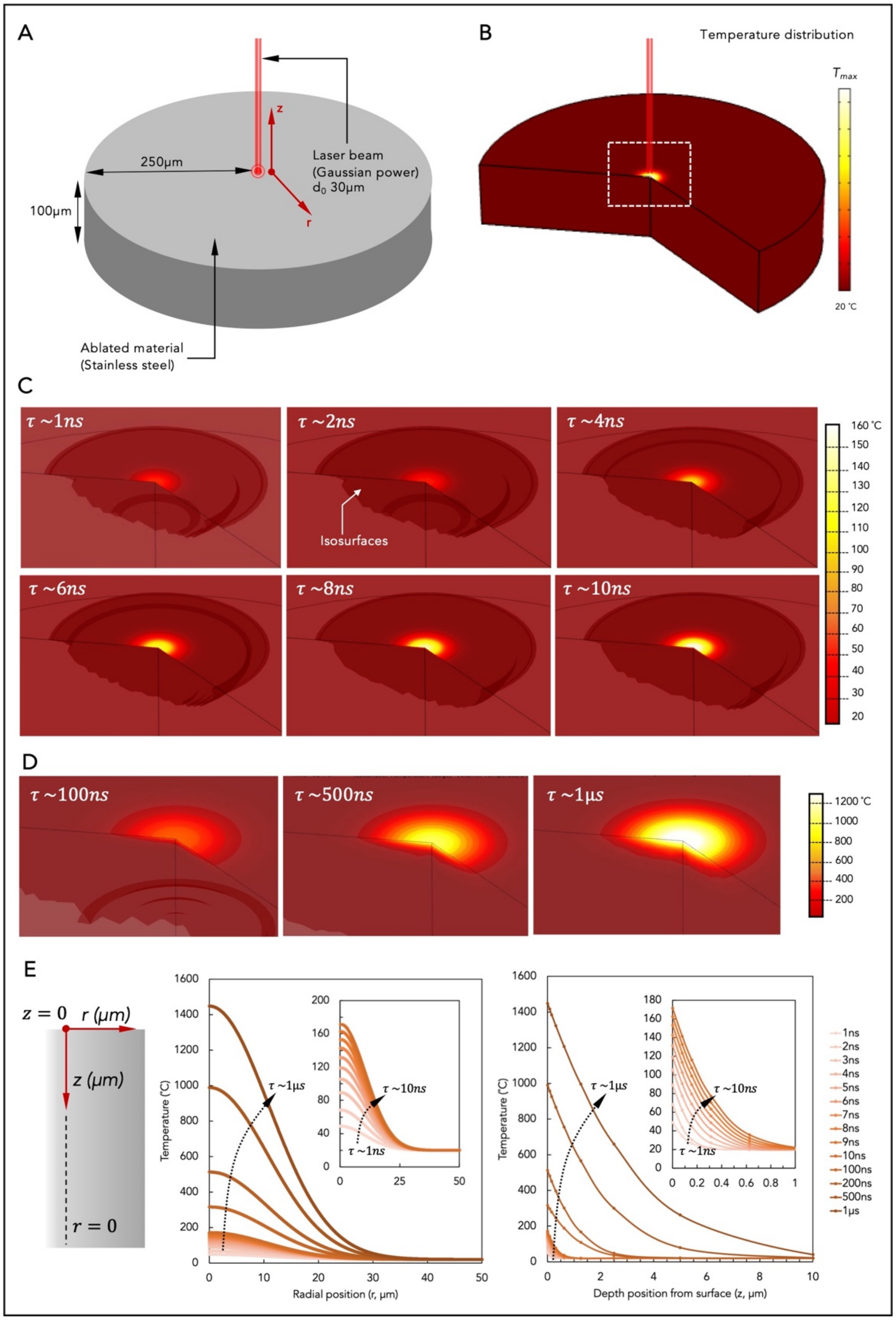

Fig. S3. FEM simulations of NIR nanosecond-pulsed laser ablation.

(**A**) Domain used for the simulation: cylindrical block of stainless steel. (**B**) Temperature distribution induced in the steel block by one laser pulse. (**C, D**) Temperature distribution induced by one laser pulse with the same power and different pulse durations, from 1ns to 10ns (**C**) and from 100ns to 1μs (**D**). (**E**) Radial and depth distribution of temperature in the steel block due to one laser pulse with different pulse durations, from 1ns to 1μs.

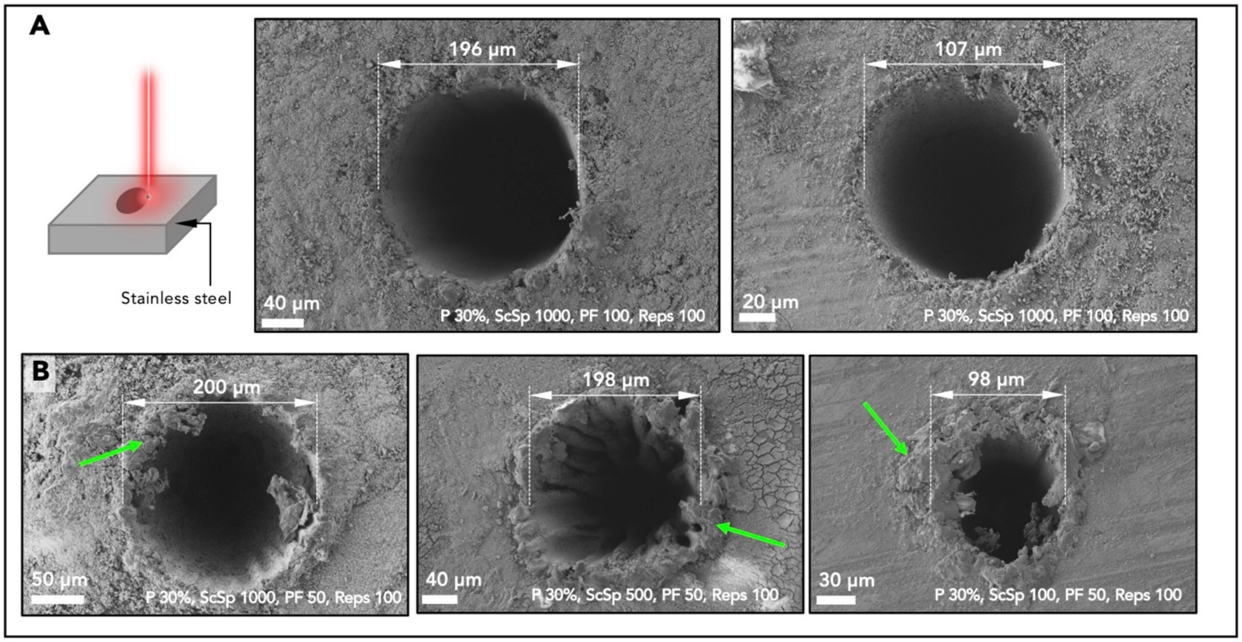

Fig. S4. Different scenarios of laser ablation on stainless steel, showing different physical mechanisms occurring in the material.

(**A**) Circles of set diameters of 200 µm and 100 µm, ablated on stainless steel with certain laser parameters (power 30%, scan speed 1000 mm/s, pulse frequency 100 kHz, repetitions 100). The sharp and clean edges denote that the dominant ablation mechanism is material sublimation. (**B**) Circles of set diameters of 200 µm and 100 µm ablated on stainless steel with different laser parameters: the edges present signs of material melting and recast/resolidification (indicated by the green arrows), due to the more aggressive laser setting than the ones used for (**A**).

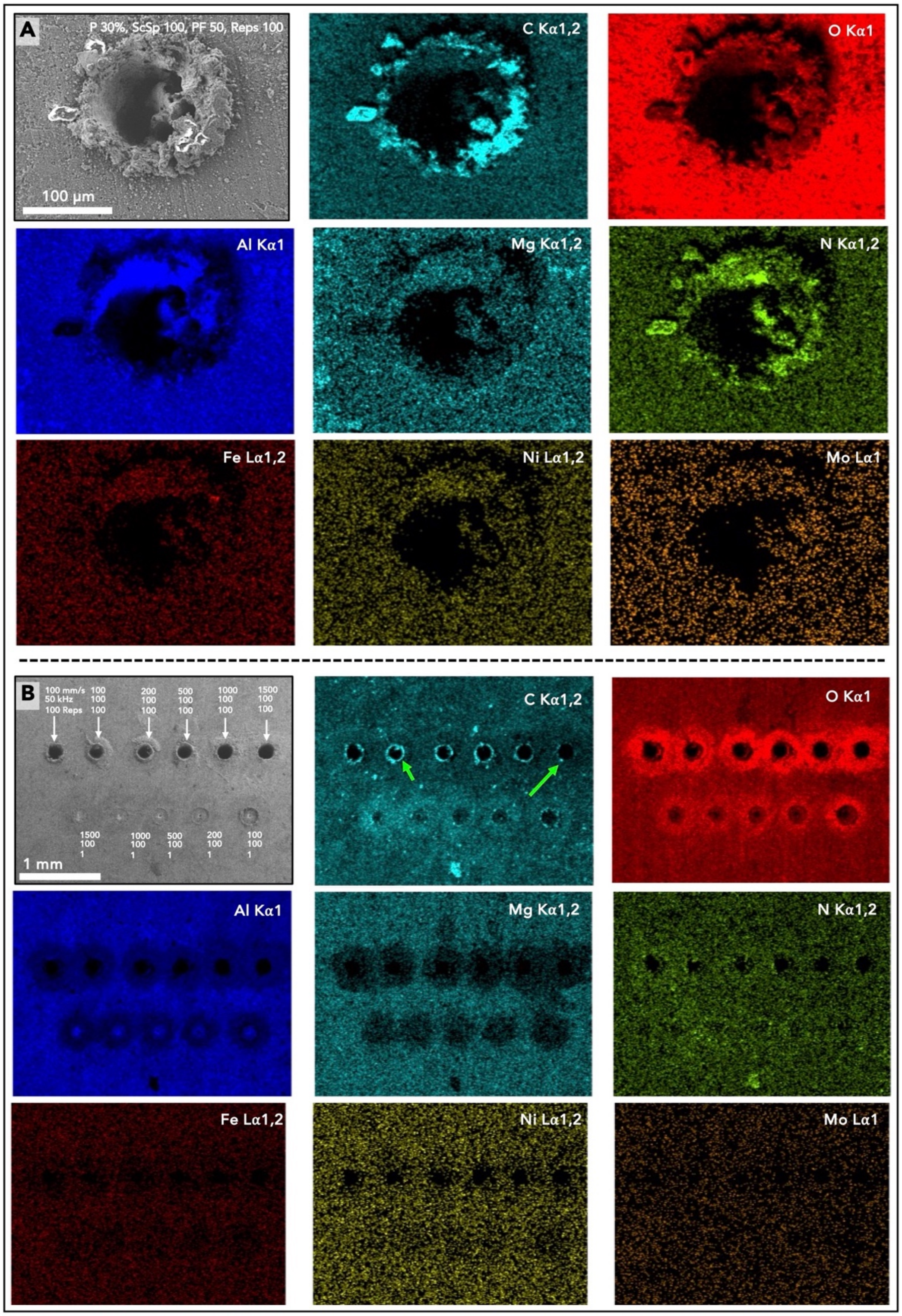

Fig. S5. EDX analysis of the edges of an ablated pattern, after the process, to investigate dominant ablation mechanisms.

(**A**) Circle of set diameter of 100 μm ablated on stainless steel with certain laser parameters (power 30%, scan speed 100 mm/s, pulse frequency 50 kHz, repetitions 100). Electron image and EDX maps for different elements (C, O, Al, Mg, N, Fe, Ni, Mo) are reported. The edges of ablation, in this case produced by melting and resolidification, present the same composition as the base substrate but with more pronounced amounts of C, O, N, due to ablation. (**B**) Circles of 100 μm

set diameter, ablated on stainless steel with different sets of laser parameters, EDX images show clearly the different relative composition of the ablation edges depending on the laser parameters.

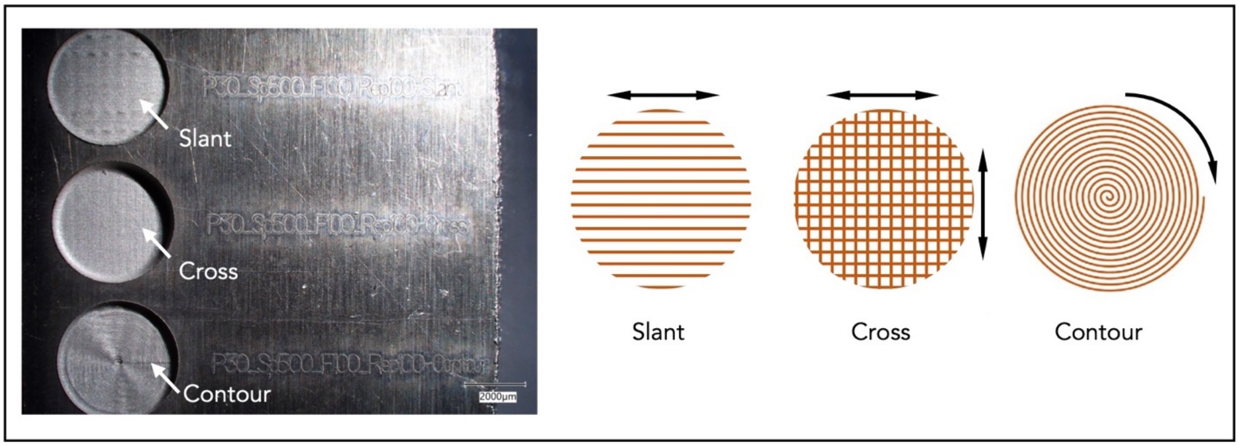

Fig. S6. Laser ablation of stainless steel with different hatching patterns.
Different hatching patterns for laser ablation of stainless steel (slant, cross, contour).

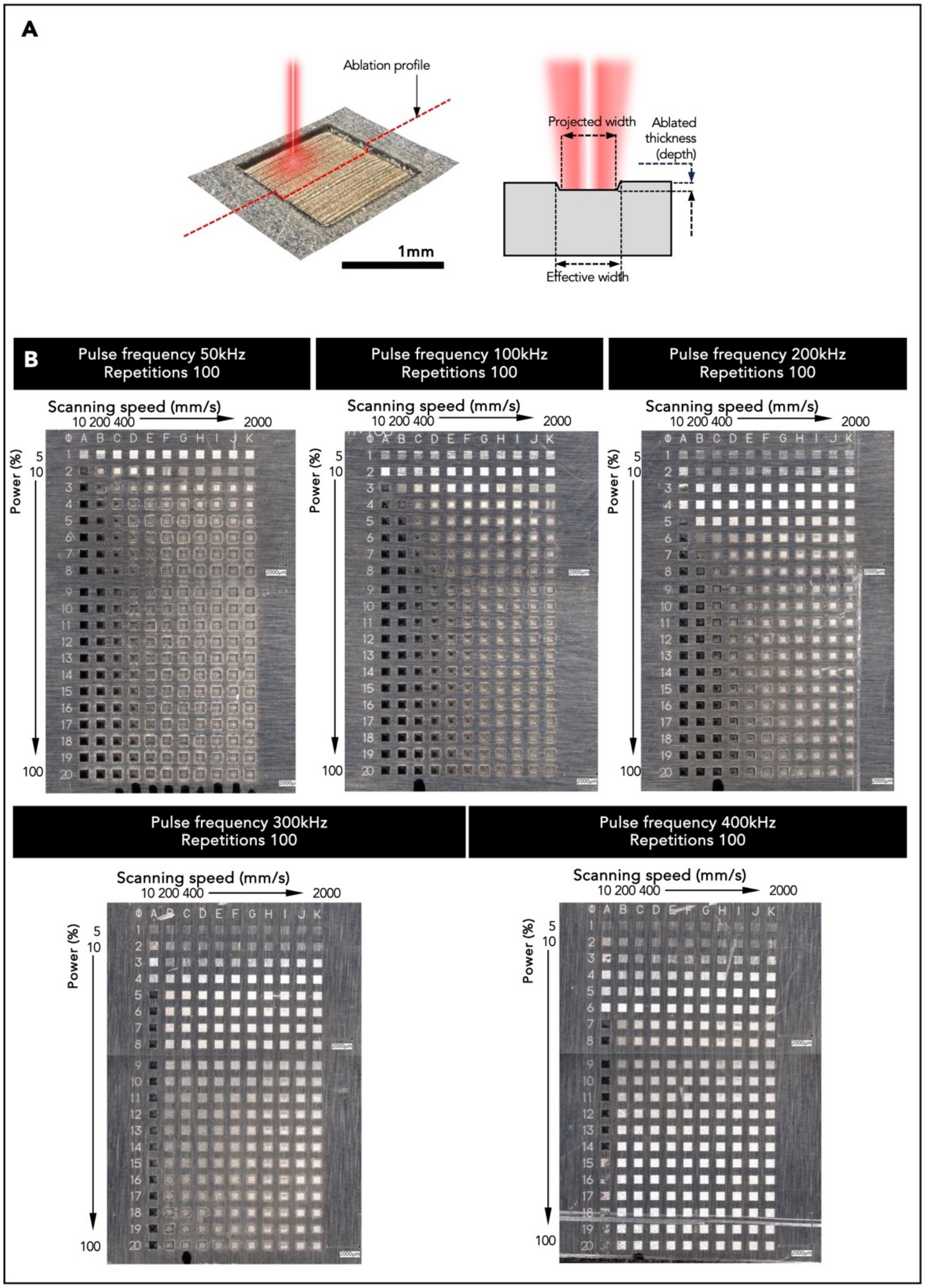

Fig. S7. Laser ablation of stainless steel: sampling matrix characterization.

(**A**) Optical 3D image of a square created by NIR laser ablation on stainless steel. The scheme shows the ablated thickness (depth), the projected width and effective width of the ablated area. (**B**) Sampling matrix of laser ablation on stainless steel with different sets of laser parameters, i.e., pulse frequency (50-400 kHz), power (0-100%), scanning speed (10-2000 mm/s), and repetitions (100).

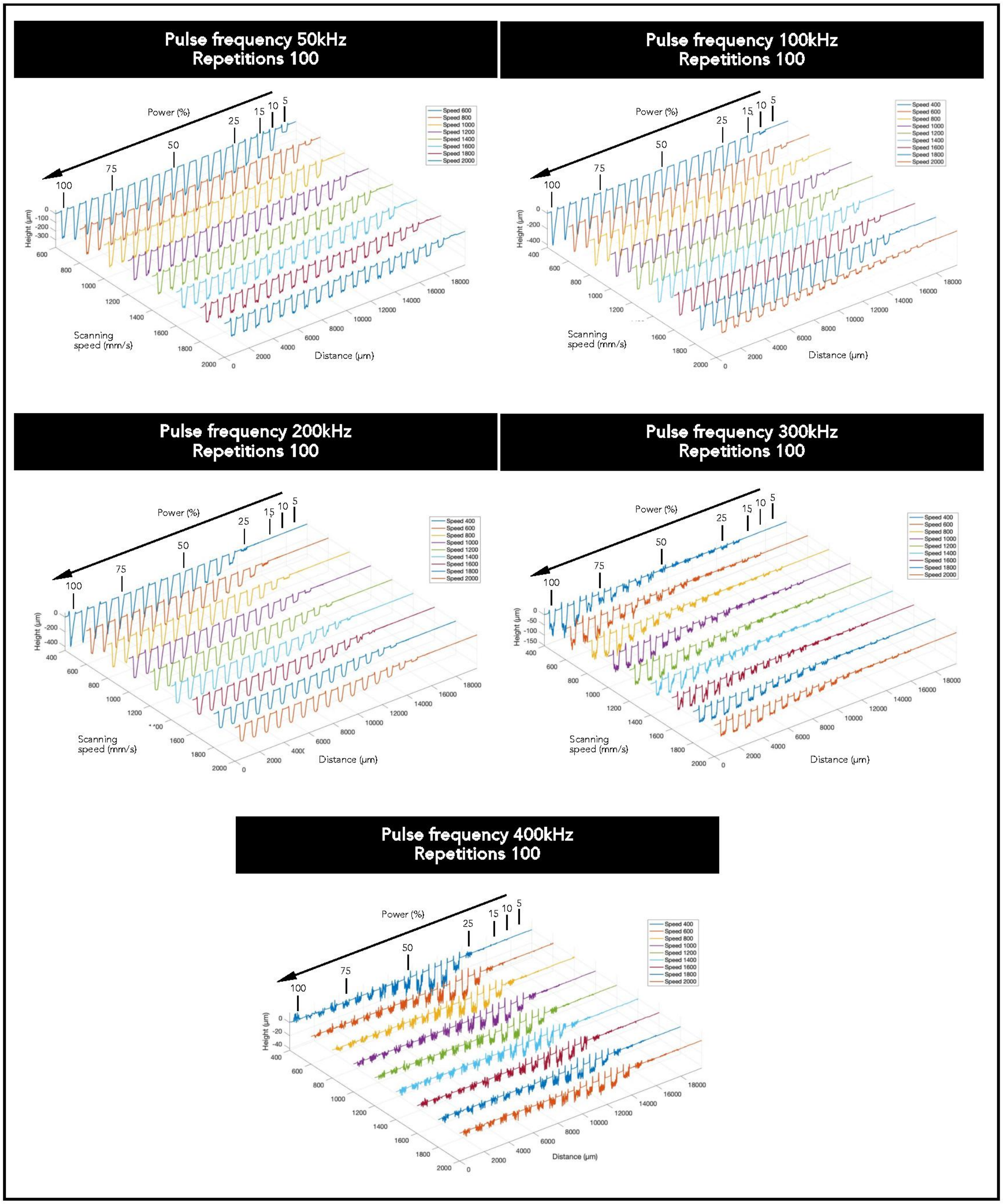

Fig. S8. Cross-sectional profiles and width measurements of the sampling matrix pattern created by laser ablation on stainless steel.

Profile measurements (raw data) of the squares ablated on stainless steel with different sets of laser parameters.

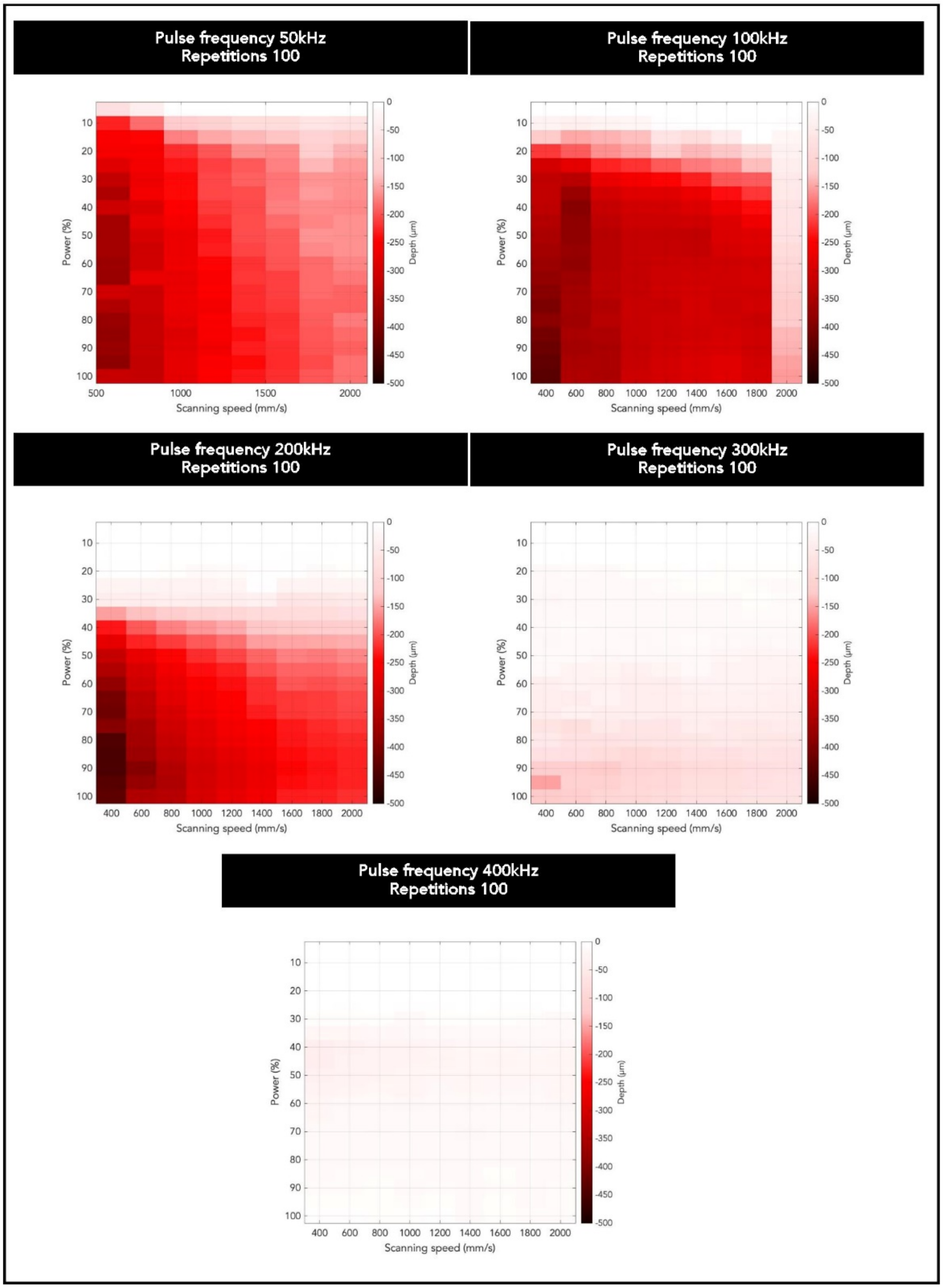

Fig. S9. Cross-sectional profiles and width measurements of the sampling matrix pattern created by laser ablation on stainless steel.

Heatmaps of the profile measurements of the squares ablated on stainless steel with different sets of laser parameters.

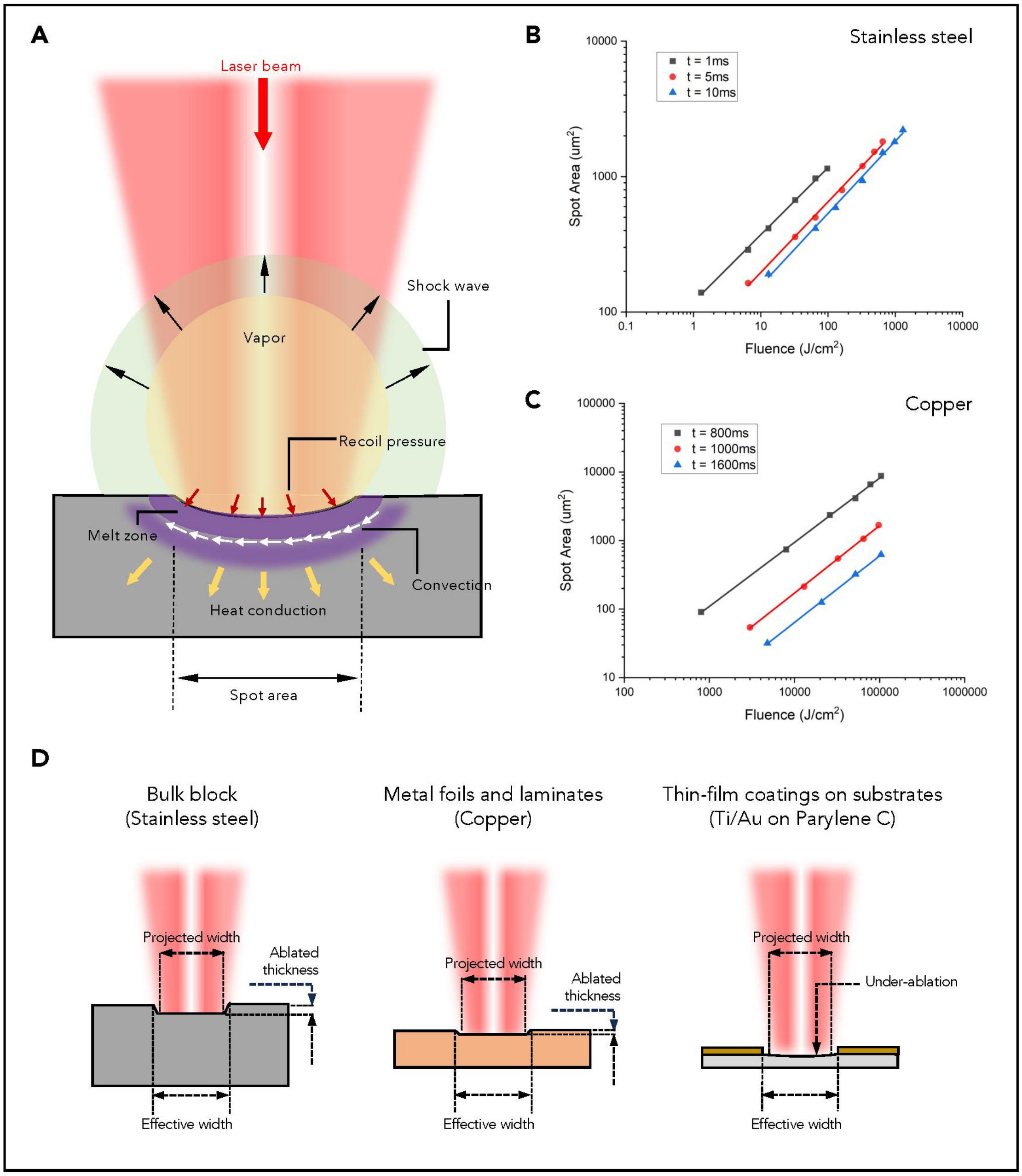

Fig. S10. Single point damage threshold of laser ablation on different materials.

(**A**) Schematic illustration of a laser ablation spot, with indication of the melt zone, vapor, heat conduction and convection. (**B, C**) Single point damage threshold measurements of the laser spot area as function of the laser fluence for different durations of ablation, for (**B**) stainless steel and (**C**) copper. (**D**) Illustration of the different response of different material systems to the laser ablation: bulk blocks of stainless steel, metal foils and laminates, thin-film coatings on substrates.

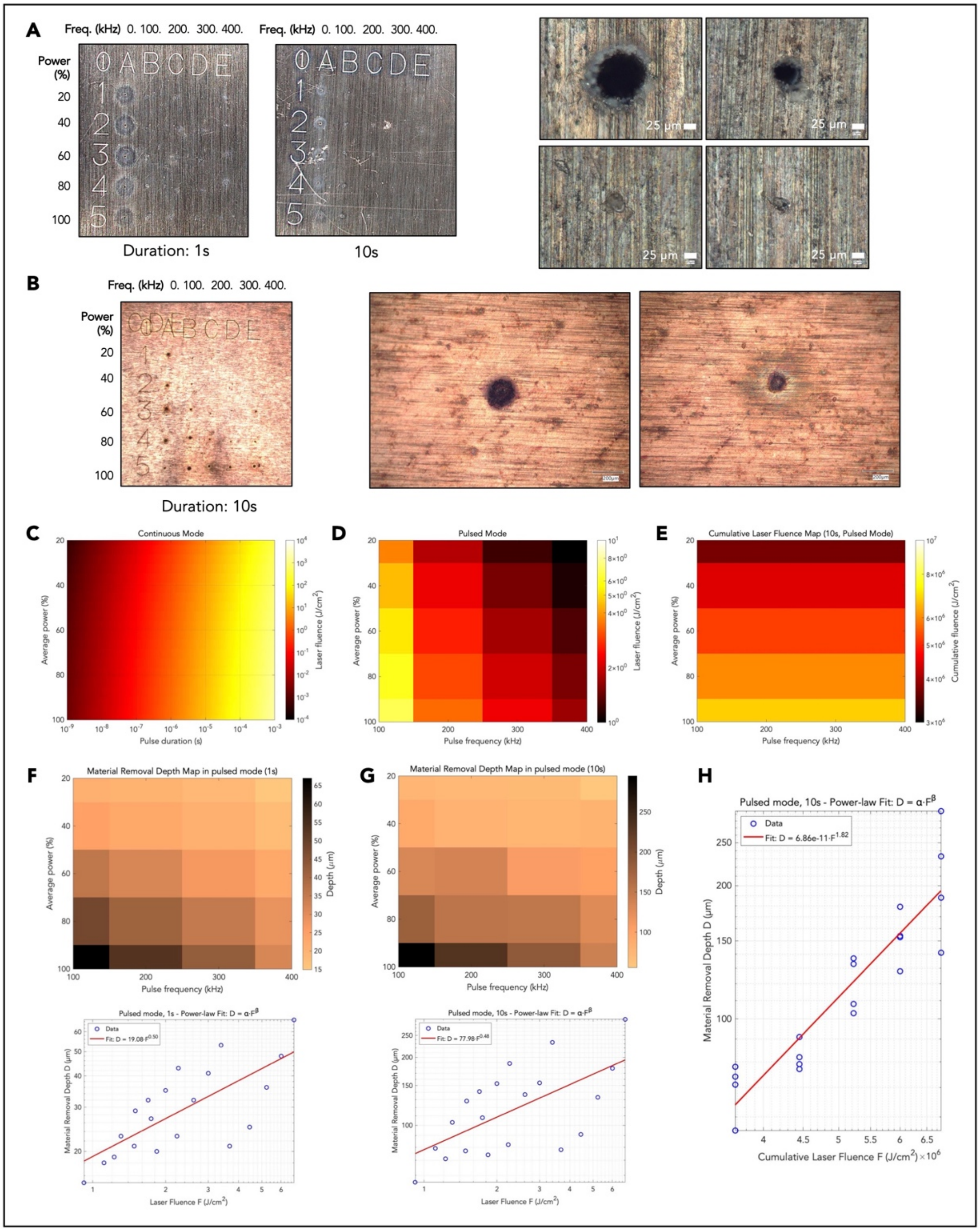

Fig. S11. Single point damage threshold of laser ablation on different materials (part 2). (**A**) Single-spot laser ablation on stainless steel using different power values and pulse frequencies. (**B**) Single-spot laser ablation on copper foil. (**C**) Heatmap of the laser fluence vs average power and pulse duration, in continuous mode. (**D**) Heatmap of the laser fluence for a single pulse vs average power and pulse frequency (in pulsed mode). (**E**) Heatmap of the cumulative laser fluence vs average power and pulse frequency, in pulsed mode for a specific time interval (10s). (**F, G**) Heatmap of the material removal depth for single-spot laser ablation on stainless steel in pulsed mode for 1s (**F**) and 10s (**G**). The removal depth follows the trend imparted by the laser fluence

(**D**), although the cumulative fluence does not depend on the pulse frequency (**E**). The plots in (**F**) and (**G**) report the depth data vs the laser fluence and the fitting curves are used for parameters extrapolation. (**H**) Depth vs cumulative fluence plot for single-spot laser ablation on stainless steel in pulsed mode for 10s, with fitting curve for parameters extrapolation.

The scattering in (F–G) mainly reflects single-shot variability (surface roughness, absorption heterogeneity, residual debris), which is particularly pronounced in stainless steel; other materials may show tighter fluence–depth trends. We nevertheless apply a power-law fit as the most established and physically meaningful model for laser ablation, enabling comparison across materials and conditions. While more datapoints would refine the fit statistically, the degree of scatter would likely persist. Importantly, this variability is strongly mitigated under cumulative exposure (H), where local fluctuations average out and the effective ablation rate aligns more closely with the fit.

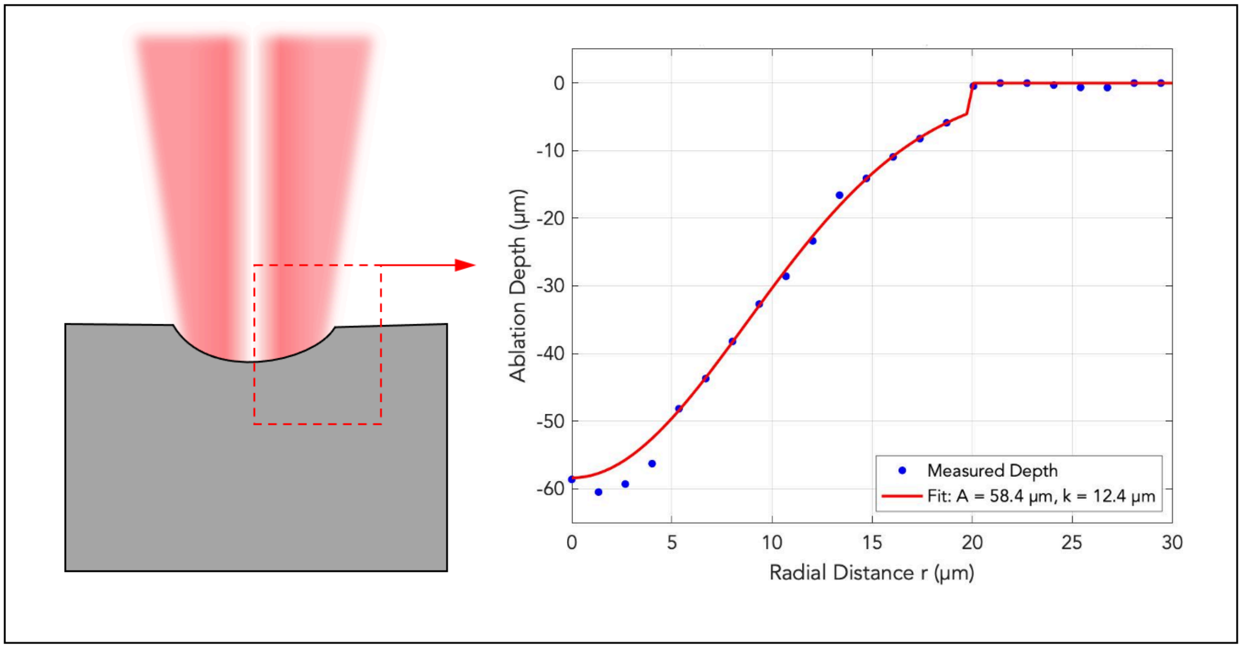

Fig. S12. Depth radial profile for single-spot ablation of stainless steel.

Measured depth radial profile of a single-spot ablation of stainless steel for the following laser parameters: average power 30%, pulse frequency 100 kHz, ablation duration 10s. The fitting curve is used to extrapolate the Gaussian parameters for the measured depth profile. The visual artifact (apparent step or singularity) at the edge of the ablation profile arises from the choice of a fitting function optimized to capture the curvature within the ablated region, rather than to provide a continuous transition to the pristine (non-ablated) surface.

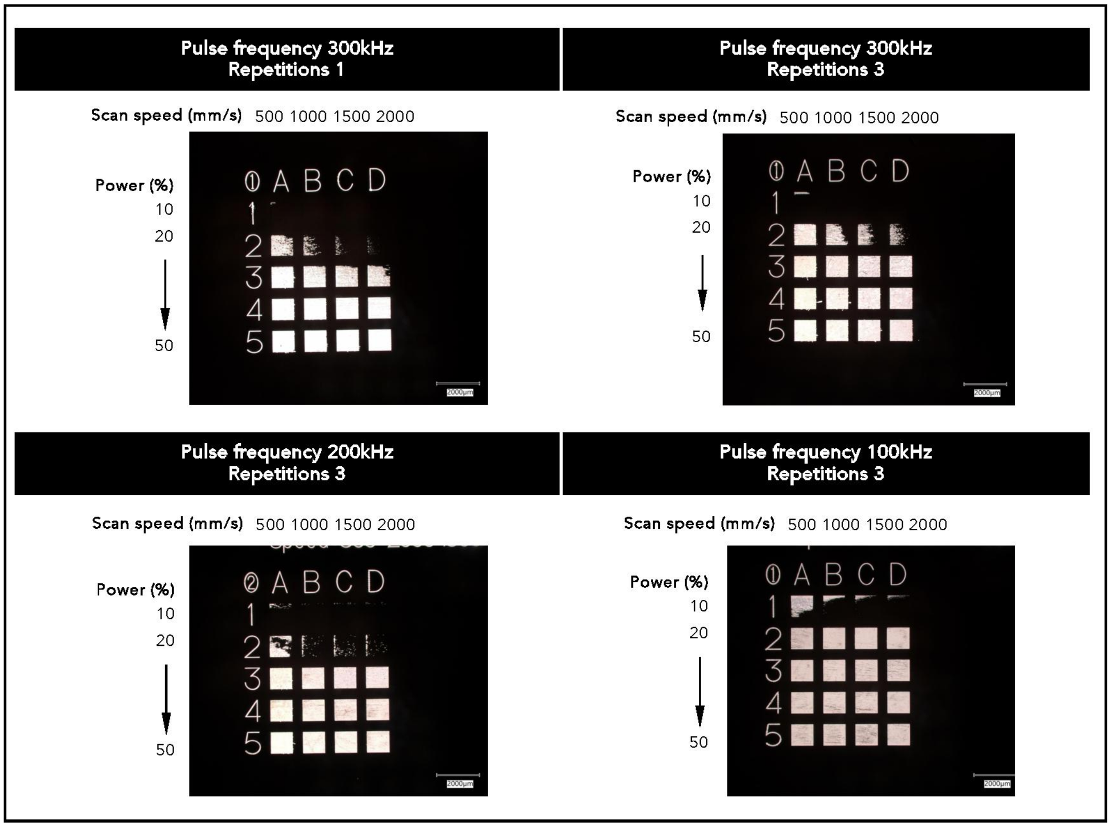

Fig. S13. Sampling matrix for characterizing the laser ablation on Ti/Au thin films on Parylene C substrate.

Laser ablation of Ti/Au thin films on Parylene C, with different representative sets of laser parameters, i.e., pulse frequency (100-300 kHz), power (10-50%), scanning speed (500-2000 mm/s), repetitions (1, 3). The bright regions are exposed areas where the Ti/Au metallization was removed by laser ablation.

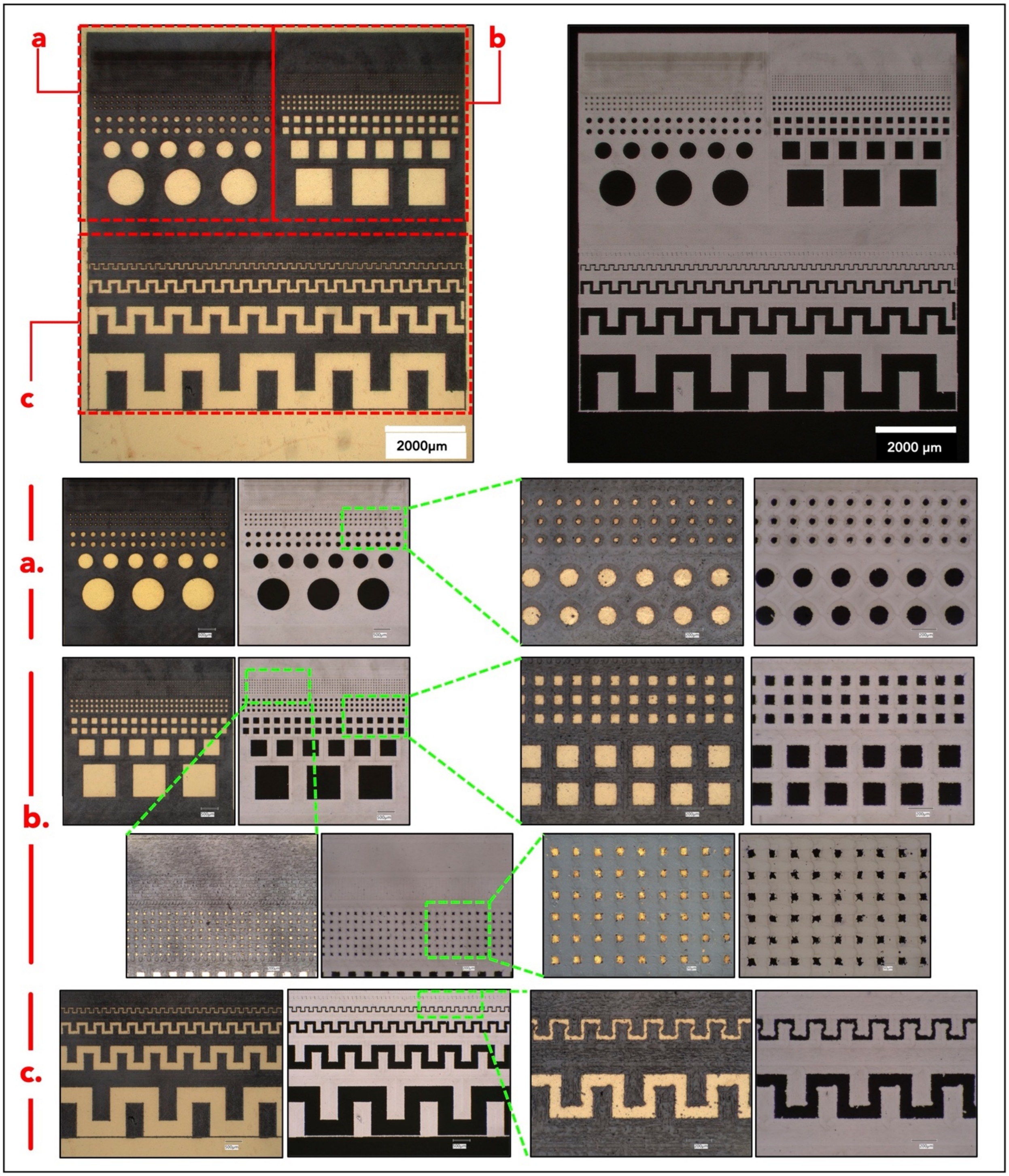

Fig. S14. Ablation of different shapes on Parylene C/Ti/Au films.

Optical images (top view) in reflection and transmission mode, of different shapes obtained by laser ablation of Ti/Au thin films on Parylene C: circles (a), squares (b) and serpentines (c). The magnified images show the resolution and edge quality of the shapes obtained by ablating the surrounding areas. The edge roughness was extracted through an image processing Python script.

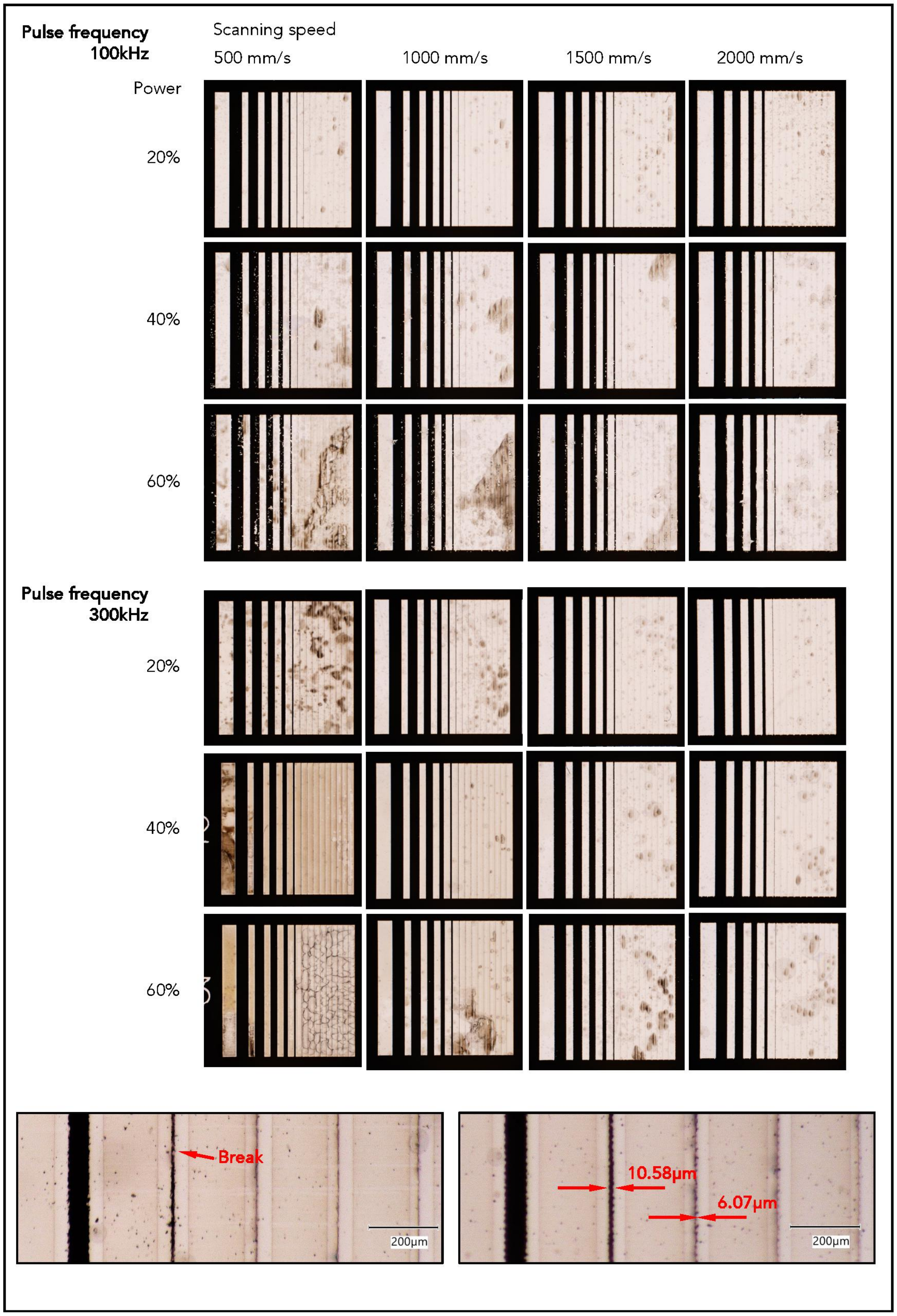

Fig. S15. Ribbon shapes created by single-layer laser ablation on Parylene C/Ti/Au.

Creation of ribbon shapes with different widths by laser ablation on Parylene C/Ti/Au to determine the in-plane lateral resolution of the ablation process on thin films. Different representative sets of laser parameters were used: pulse frequency (100, 300 kHz), power (20-60%), scanning speed (500-2000 mm/s). the minimum achievable ribbon width is indicated in the bottom micrographs.

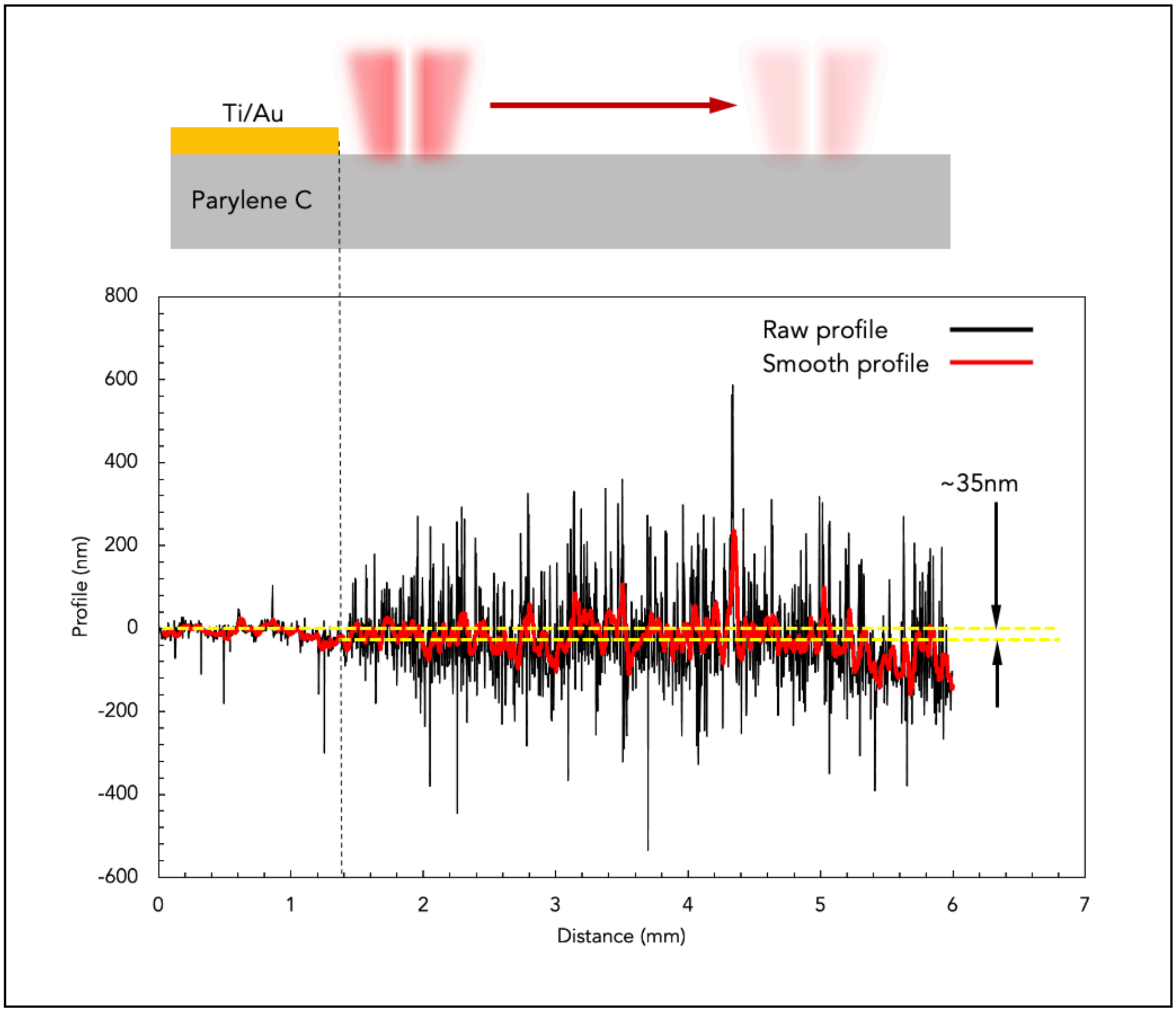

Fig. S16. Thickness reduction in Parylene C due to ablation of Ti/Au thin films.

Profile measurement of a non-ablated Parylene C/Ti/Au layer (left side) and an ablated region (Parylene C) after removal of Ti/Au (right side), with the optimized laser parameters used in this study.

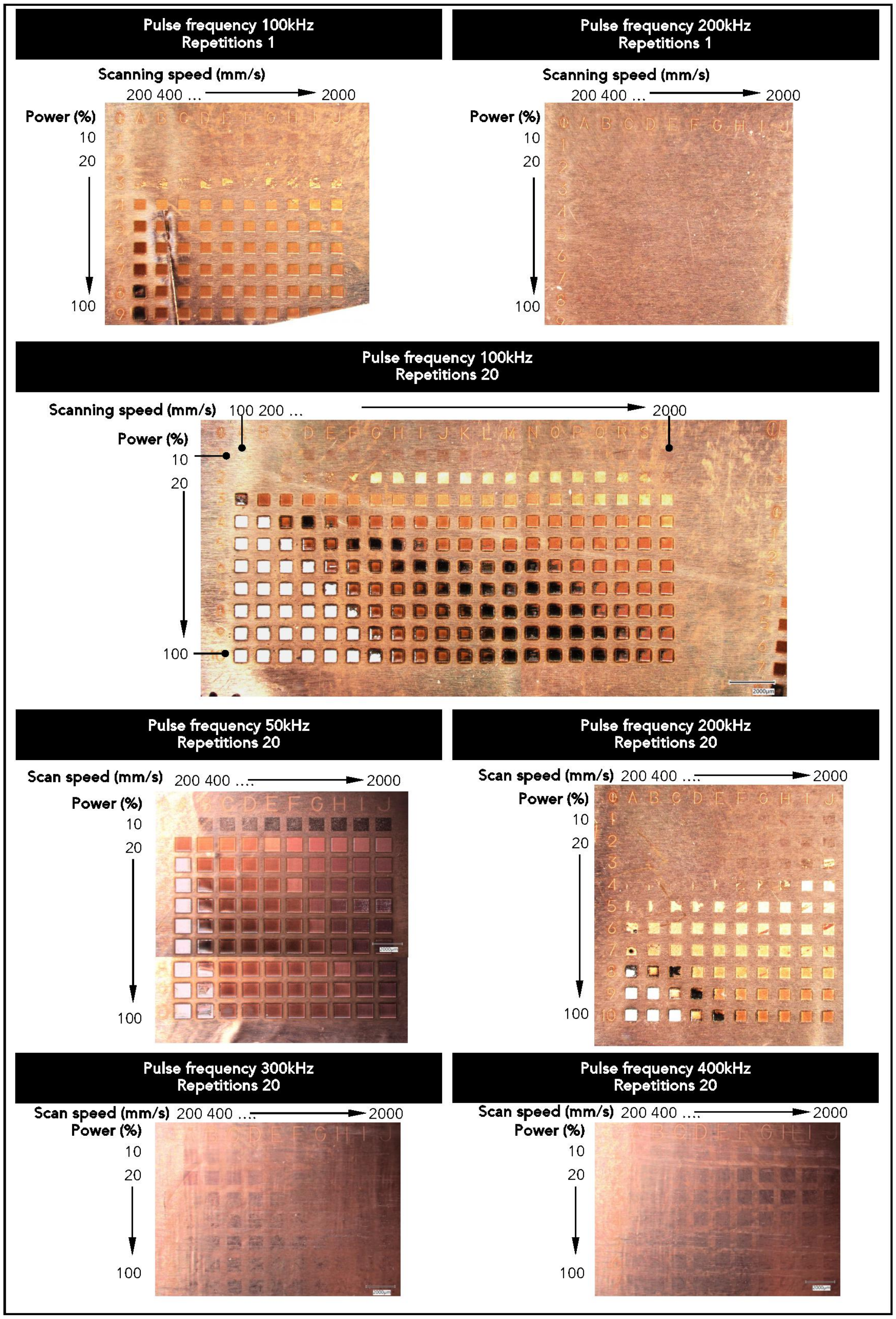

Fig. S17. Sampling matrix for characterizing the laser ablation on Cu foils.

Laser ablation of Cu foils, with different representative sets of laser parameters, i.e., pulse frequency (50-400 kHz), power (10-100%), scanning speed (200-2000 mm/s), repetitions (1, 20).

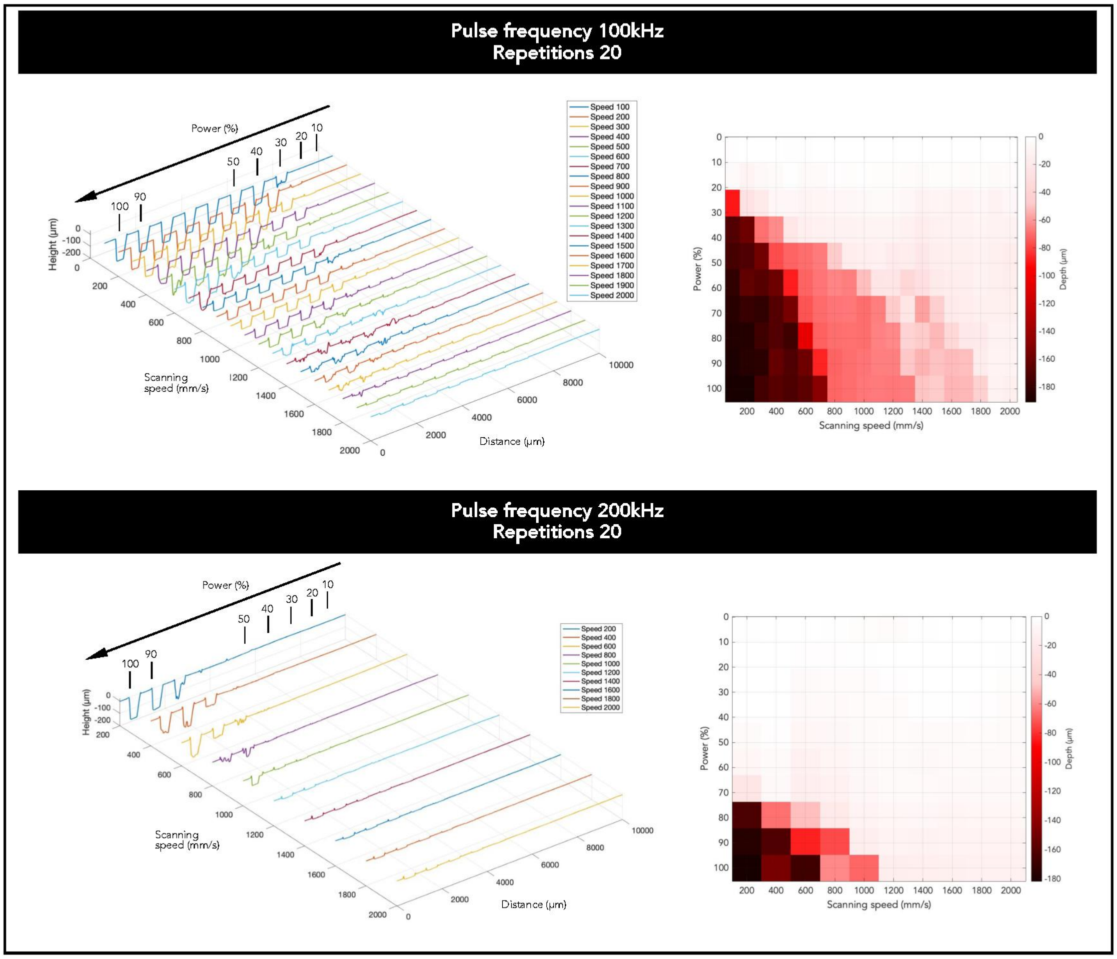

Fig. S18. Profile measurements for laser ablation on Cu foils.

Profile measurements of for laser ablation of Cu foils, with different representative sets of laser parameters, i.e., pulse frequency (100, 200 kHz), power (10-100%), scanning speed (200-2000 mm/s), repetitions (20).

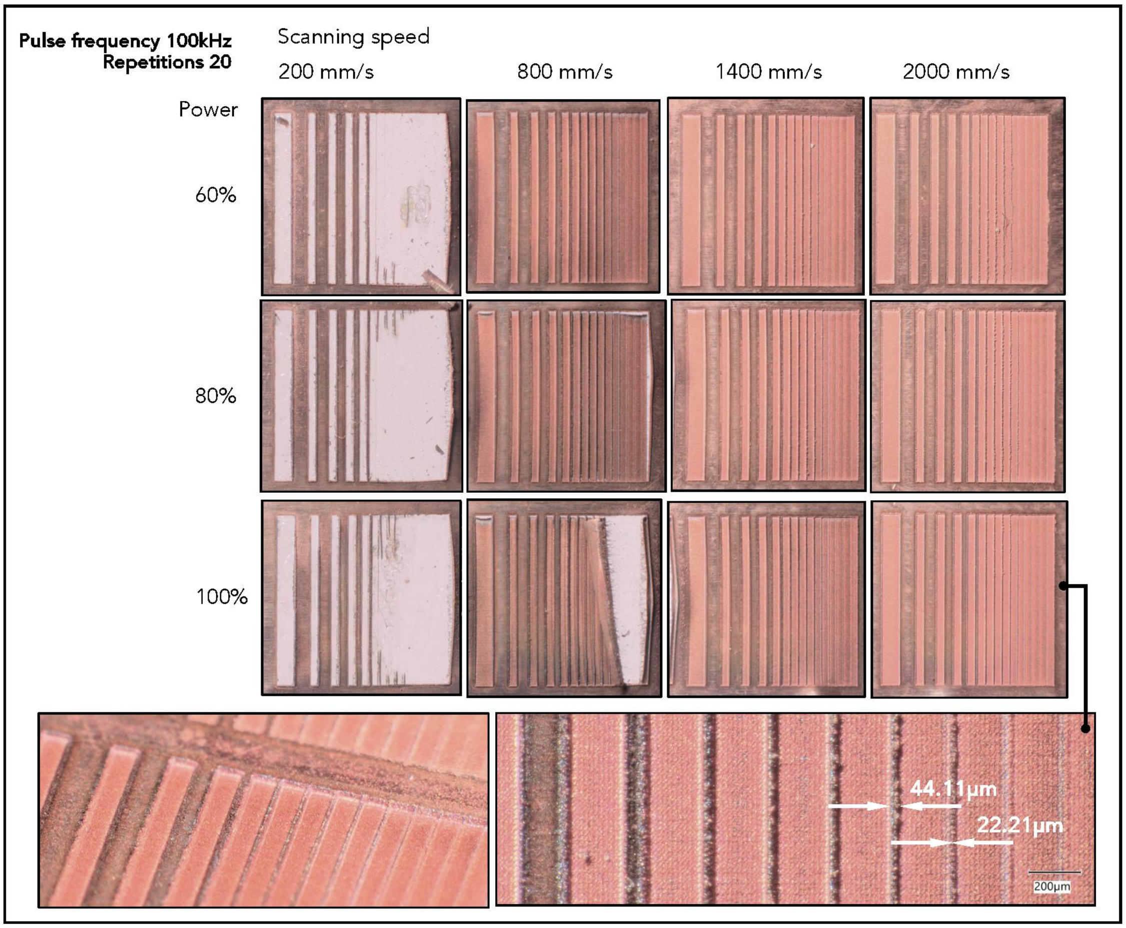

Fig. S19. Ribbon shapes created by single-layer laser ablation on Cu foils.

Creation of ribbon shapes with different widths by laser ablation on Cu foils to determine the in-plane lateral resolution of the ablation process on metal foils. Different representative sets of laser parameters were used: pulse frequency (100 kHz), power (60-100%), scanning speed (200-2000 mm/s). The minimum achievable ribbon width is indicated in the bottom micrographs.

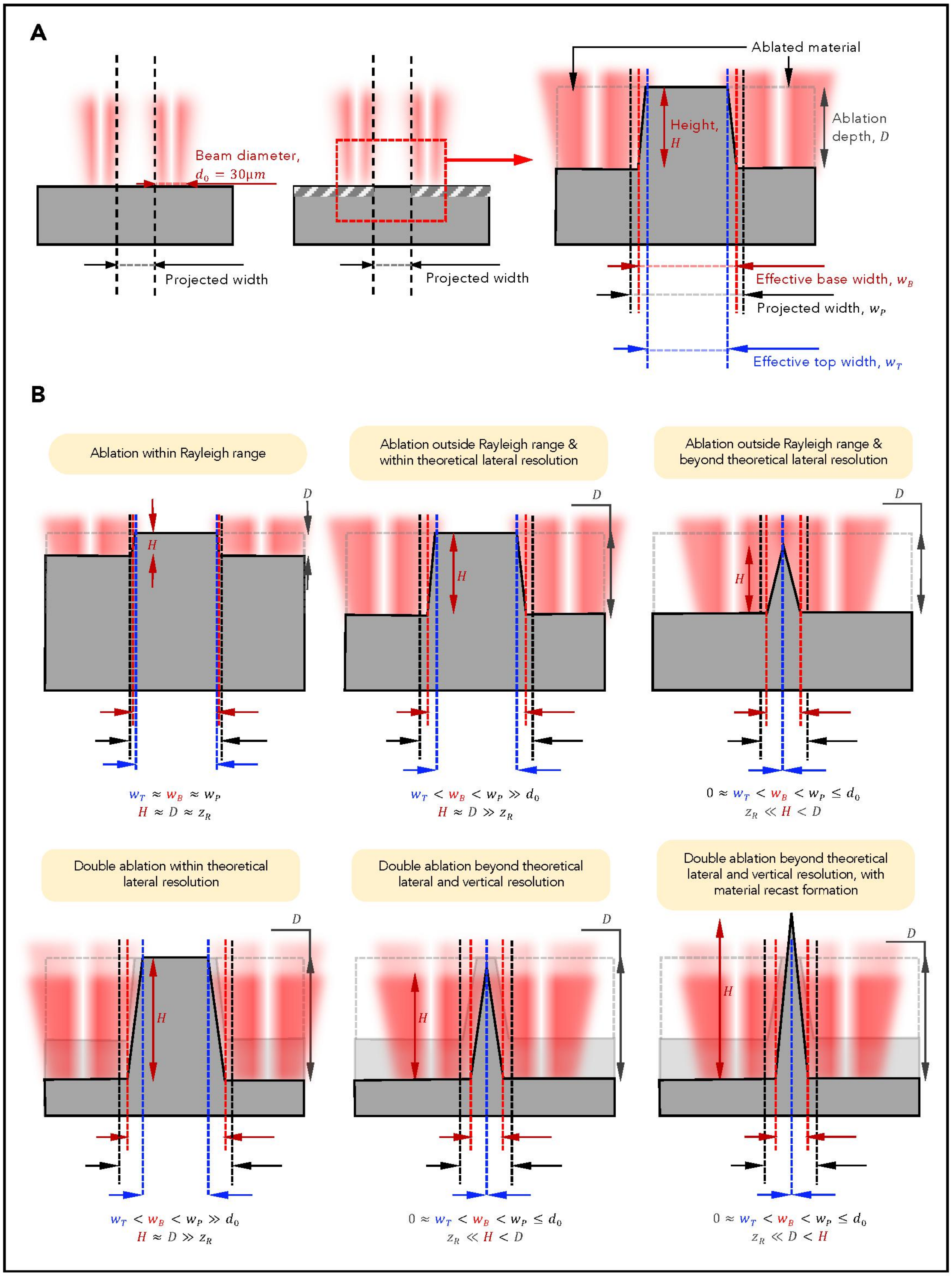

Fig. S20. Illustrations of the ablated ribbon shapes used to determine the lateral resolution. (**A**) Illustration showing two parallel laser beams separated by a ribbon with a projected width (w_P). The ablation creates a ribbon with a trapezoidal cross section, where the following parameters can be identified: effective base width (w_B), effective top width (w_T), height (H) and ablation depth (D). (**B**) Illustrations of different scenarios of ablation.

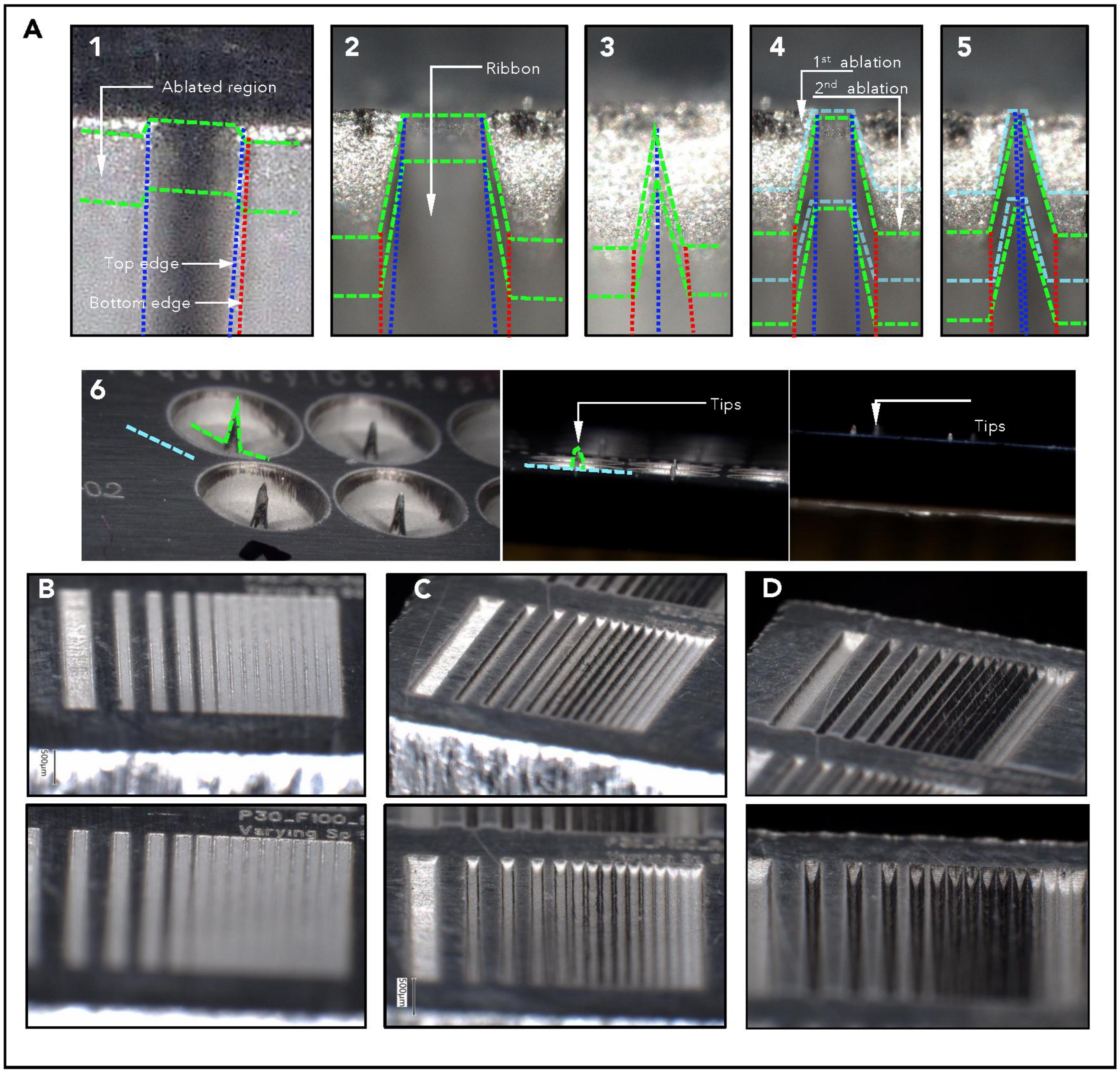

Fig. S21. Different experimental scenarios of ribbon shapes created by single-layer and multi-layer ablation processes.

(**A**) Six different cases of ablation of ribbon shapes (see previous figure). (**B**) Scenario 1. (**C**) Scenario 2. (**D**) Scenario 4.

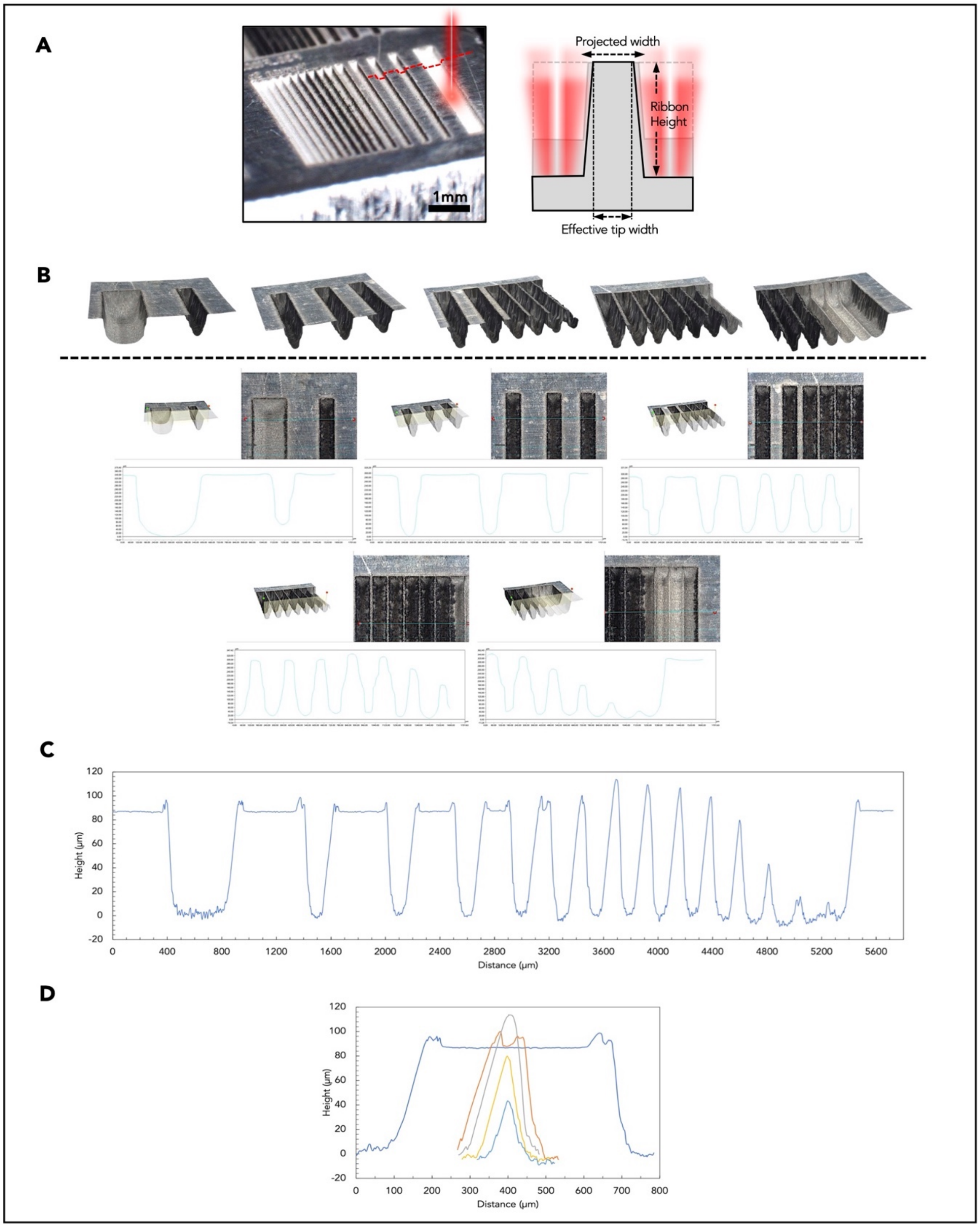

Fig. S22. Profile measurements of the ribbon shapes created by laser ablation on stainless steel.

(**A**) Optical 3D image of a sequence of ribbon shapes created by NIR laser ablation on stainless steel. The scheme shows the ribbon height, the projected width and the effective tip width. (**B**) Optical measurement of the ribbon shape, width and height. (**C**) Example of a profile measurement of a series of ribbons with progressively decreasing width. (**D**) Overlaid profiles of ribbons with different widths, corresponding to the six scenarios shown in the previous figure.

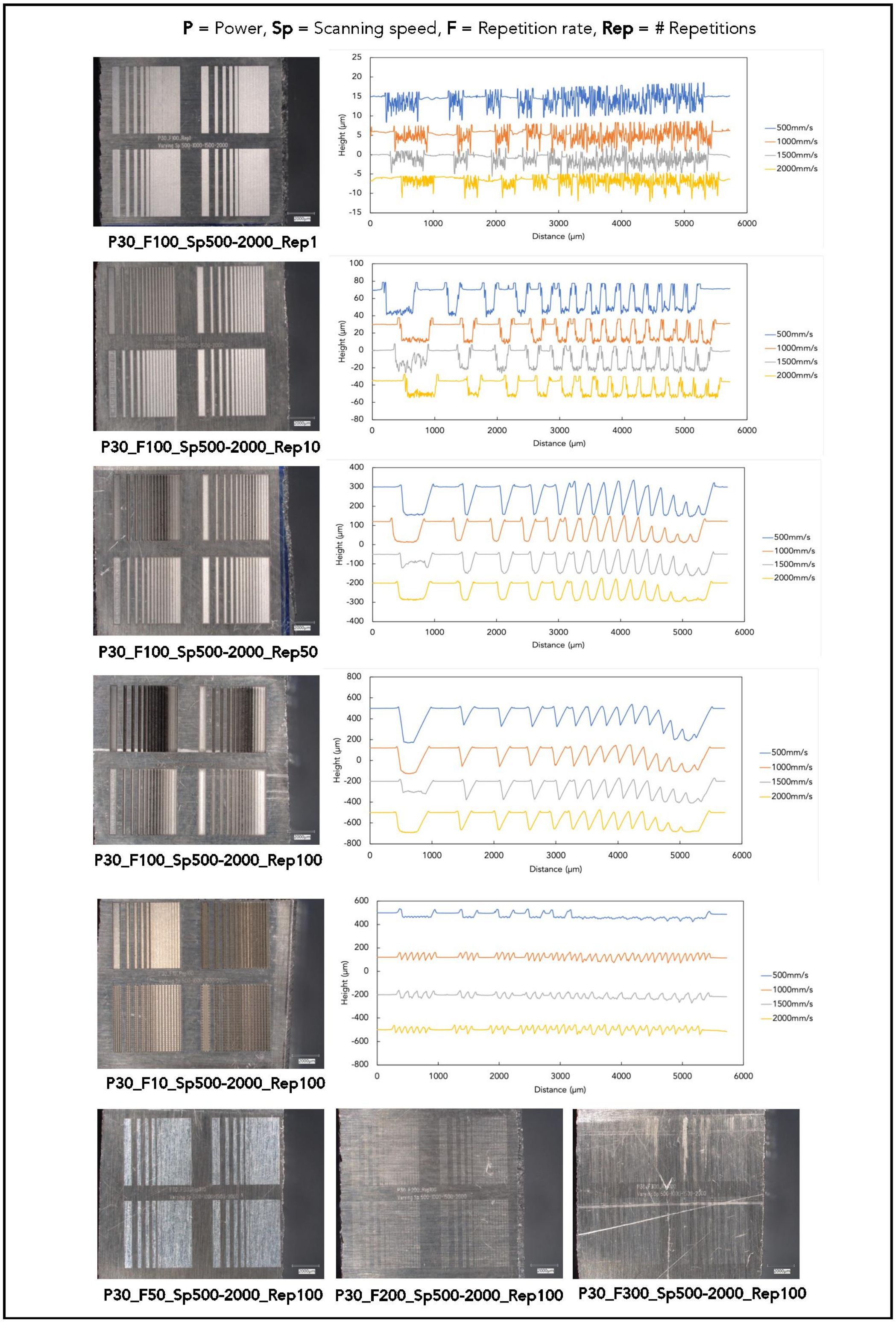

Fig. S23. Different experimental scenarios of ribbon shapes created by single-layer and multi-layer ablation processes.

Profile measurements of several ribbon shapes for different representative sets of laser parameters.

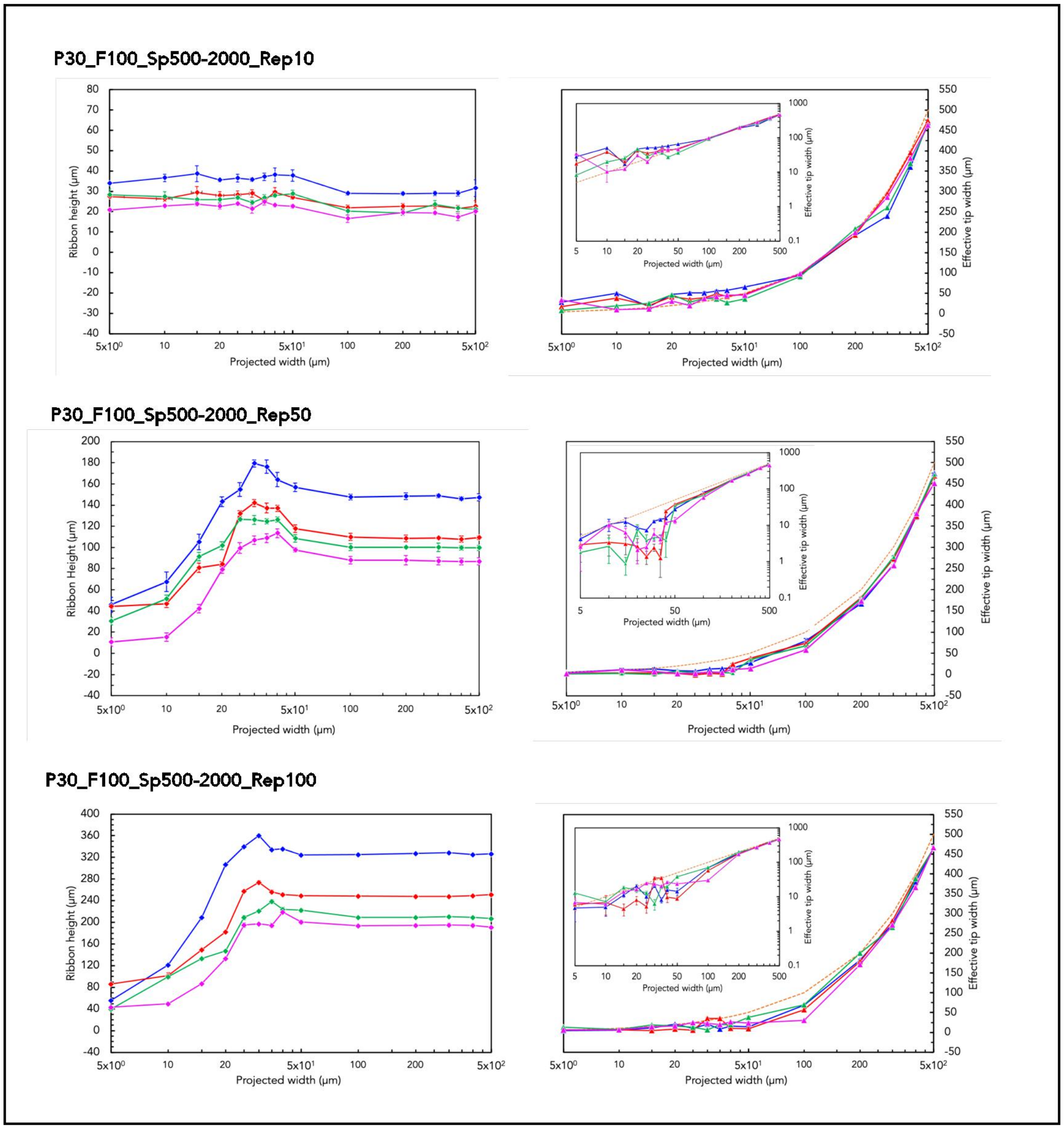

Fig. S24. Ribbon height and effective tip width of ribbon shapes created by single-layer and multi-layer ablation processes.

Ribbon height vs projected width (left) and effective tip width vs projected width (right) for the ribbon shapes created by ablation for different representative sets of laser parameters. The insets show the plots on the right in a logarithmic scale for both axes. The point where deviations from the ideal 1:1 ratio start to appear is taken as the lateral resolution.

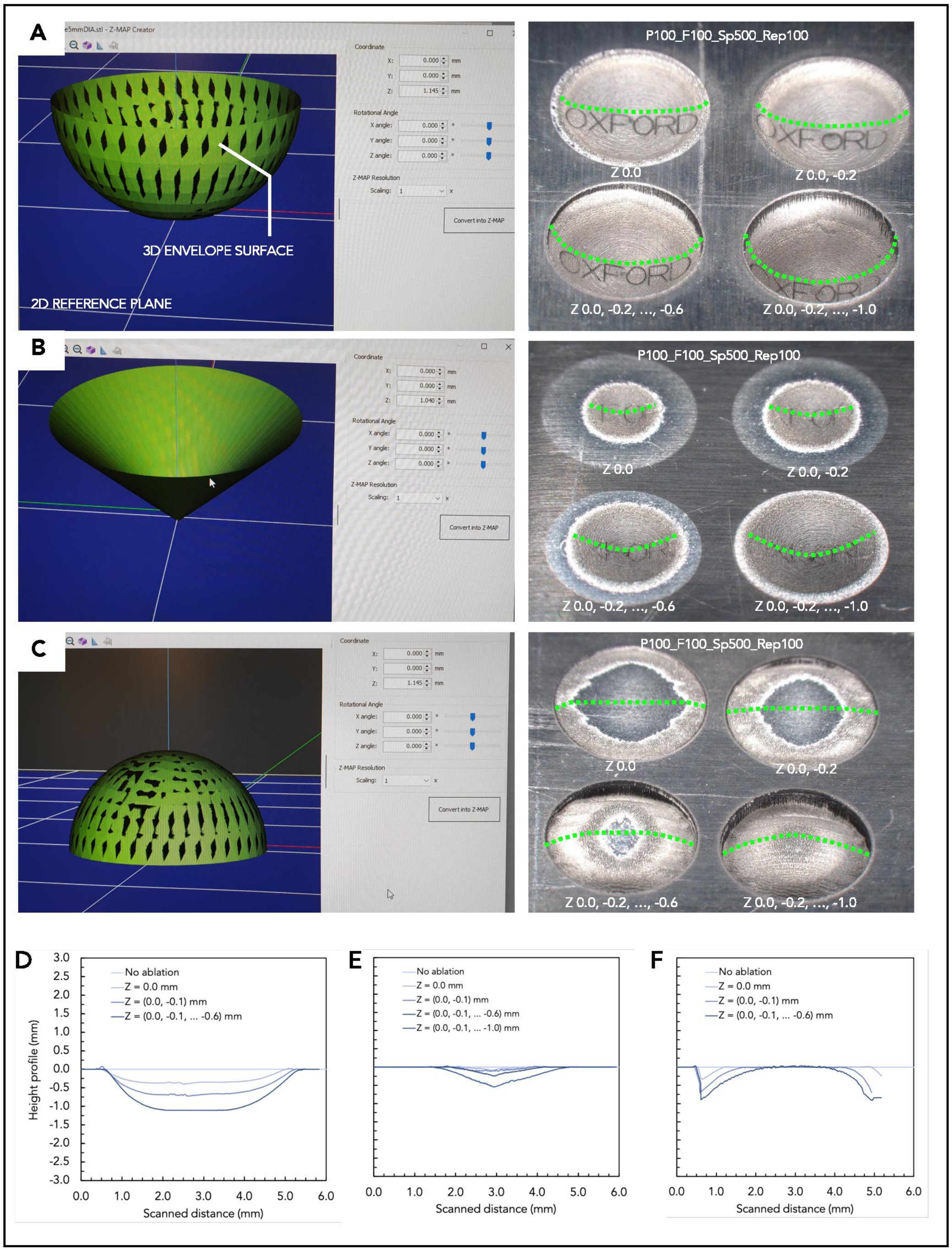

Fig. S25. Examples of 3D shapes created on stainless steel by d-3DPLM.

Sequential ablation at different z steps along a 3D envelope surface, i.e., a convex hemisphere (**A**), a cone (**B**) and a concave hemisphere (**C**). The photos show subsequent shapes created by d-3DPLM: increasing the number of layers with progressive z steps allows to engrave the full 3D envelope surface in the steel block. The green dotted lines indicate the profiles of the real ablated 3D surface. (**D-F**) Profiles measured by mechanical profilometry or optical 3D microscopy of the 3D shapes ablated on stainless steel by d-3DPLM with the same set of parameters (power 30%, scanning speed 500 mm/s, repetition rate 100 kHz, repetitions 100, z step 0.1 mm), for a convex hemisphere (**D**), a cone (**E**) and a concave hemisphere (**F**). The profiles correspond to intermediate steps of sequential ablation at different z steps, from which the creation of the target shape is evident.

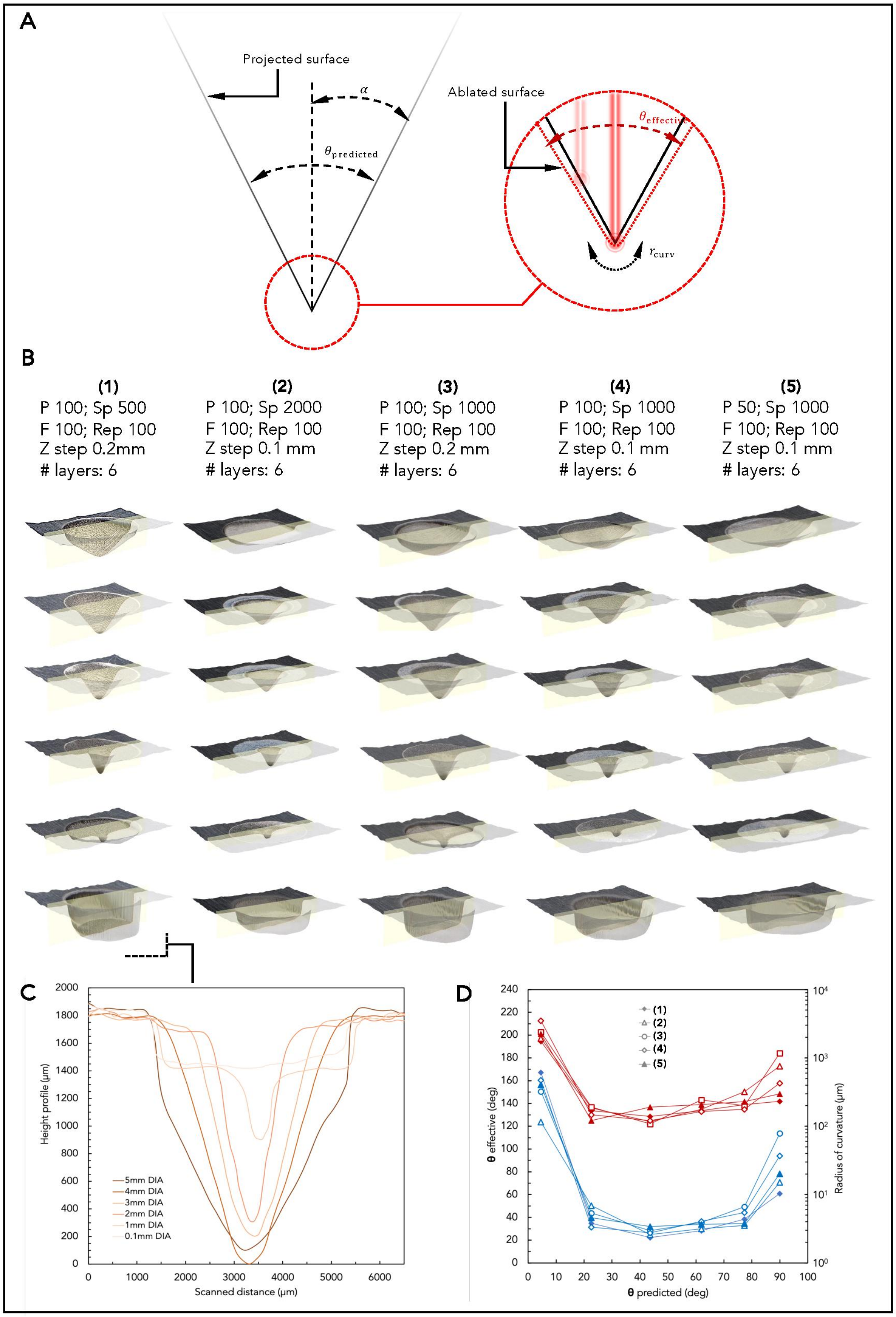

Fig. S26. Ablated 3D conical shapes to assess the 3D angular resolution of d-3DPLM.
Sequential ablation at a set number of z steps (with fixed z step) along a 3D conical surface, with different full apex angles θ (or solid angles Ω). (**A**) Schematic of the conical profile with indication of some key geometrical parameters. (**B**) Optical images of the cross sections of the conical shapes after d-3DPLM. (**C**) Profile of the conical shapes with different full-apex angles and for a specific set of laser parameters. (**D**) Effective full-apex angles and tip radius of curvature for the ablated conical shapes, as function of the predicted full-apex angle, for different sets of laser parameters.

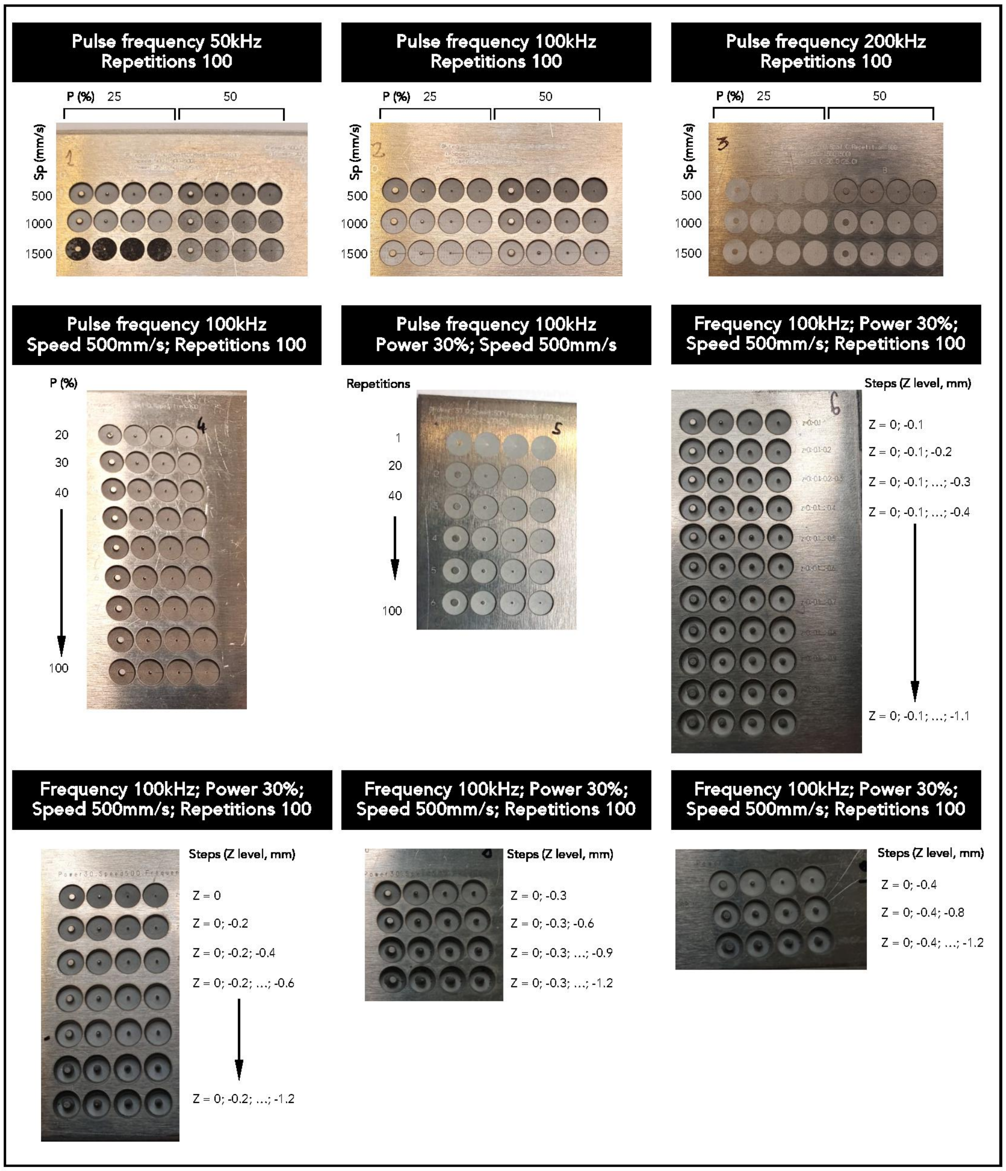

Fig. S27. Creation of MN electrodes with different diameters and laser parameters.

Ablation of MNs with different diameters (50, 100, 200, 1000 µm), with different laser parameters and ablation step-sizes.

Top-left to right: pulse frequency (50-200 kHz), scanning speed (500-1500 mm/s), power (25, 50 %), z = 0.

Middle-left: pulse frequency 100 kHz, scanning speed 500 mm/s, repetitions 100, power 20-100 %, z = 0. **Middle-center**: pulse frequency 100 kHz, scanning speed 500 mm/s, power 30%, repetitions 1-100, z = 0. **Middle-right**: pulse frequency 100 kHz, scanning speed 500 mm/s, power 30%, repetitions 100, z steps 0.1 mm.

Bottom-left: pulse frequency 100 kHz, scanning speed 500 mm/s, power 30%, repetitions 100, z steps 0.2 mm. **Bottom-center**: pulse frequency 100 kHz, scanning speed 500 mm/s, power 30%, repetitions 100, z steps 0.3 mm. **Bottom-right**: pulse frequency 100 kHz, scanning speed 500 mm/s, power 30%, repetitions 100, z steps 0.4 mm.

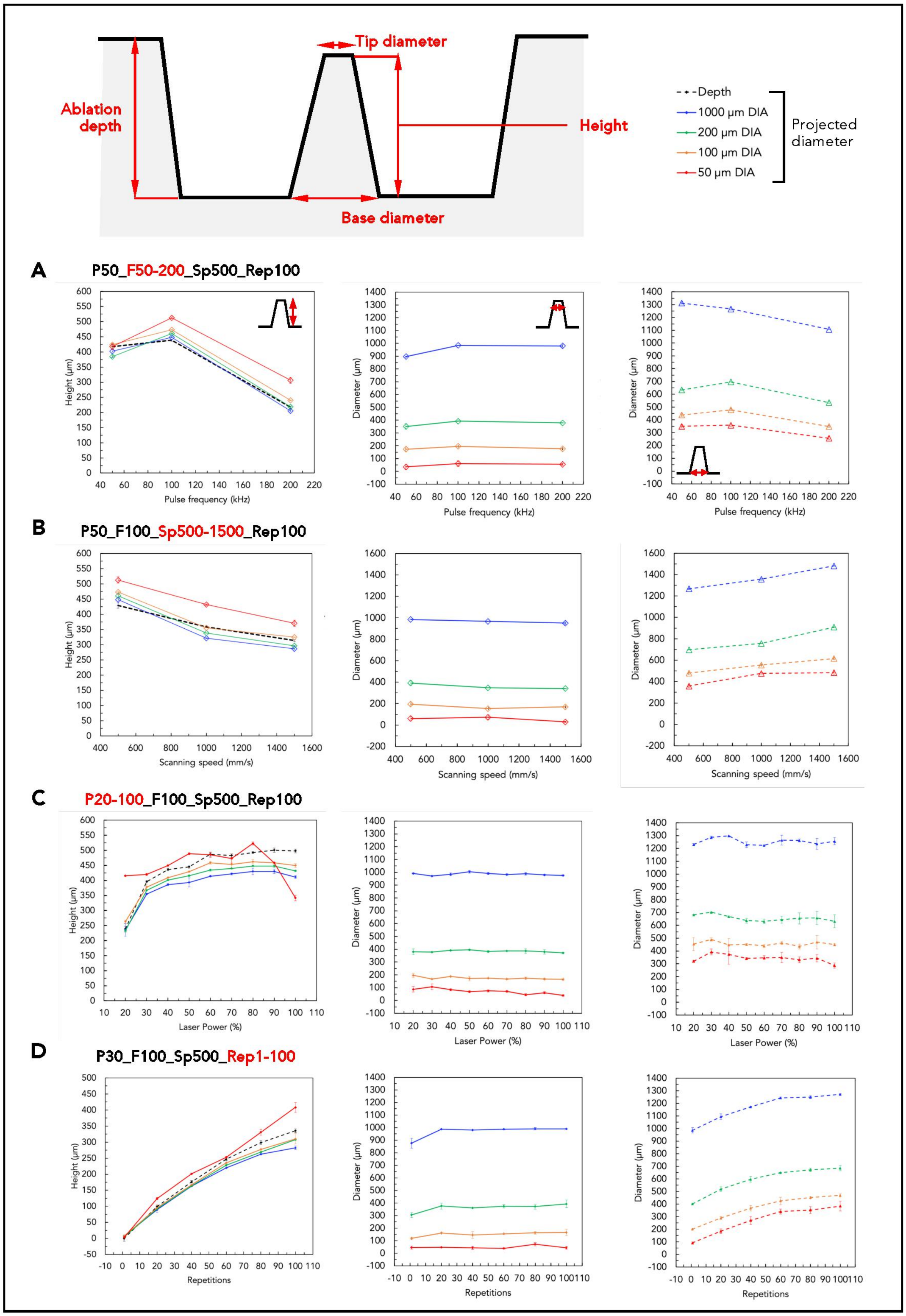

Fig. S28. Height, base/tip diameter for the MN created by ablation on stainless steel (z=0).
Height (left plot), tip diameter (middle plot) and base diameter (right plot) of the MNs created by ablation with different laser parameters, and with different projected diameters: (**A**) P50, F50-200, Sp500, Rep100; (**B**) P50, F100, Sp500-1500, Rep100; (**C**) P20-100, F100, Sp500, Rep100; (**D**) P30, F100, Sp500, Rep1-100.
P: power, Sp: scanning speed, F: pulse frequency, Rep: repetitions.

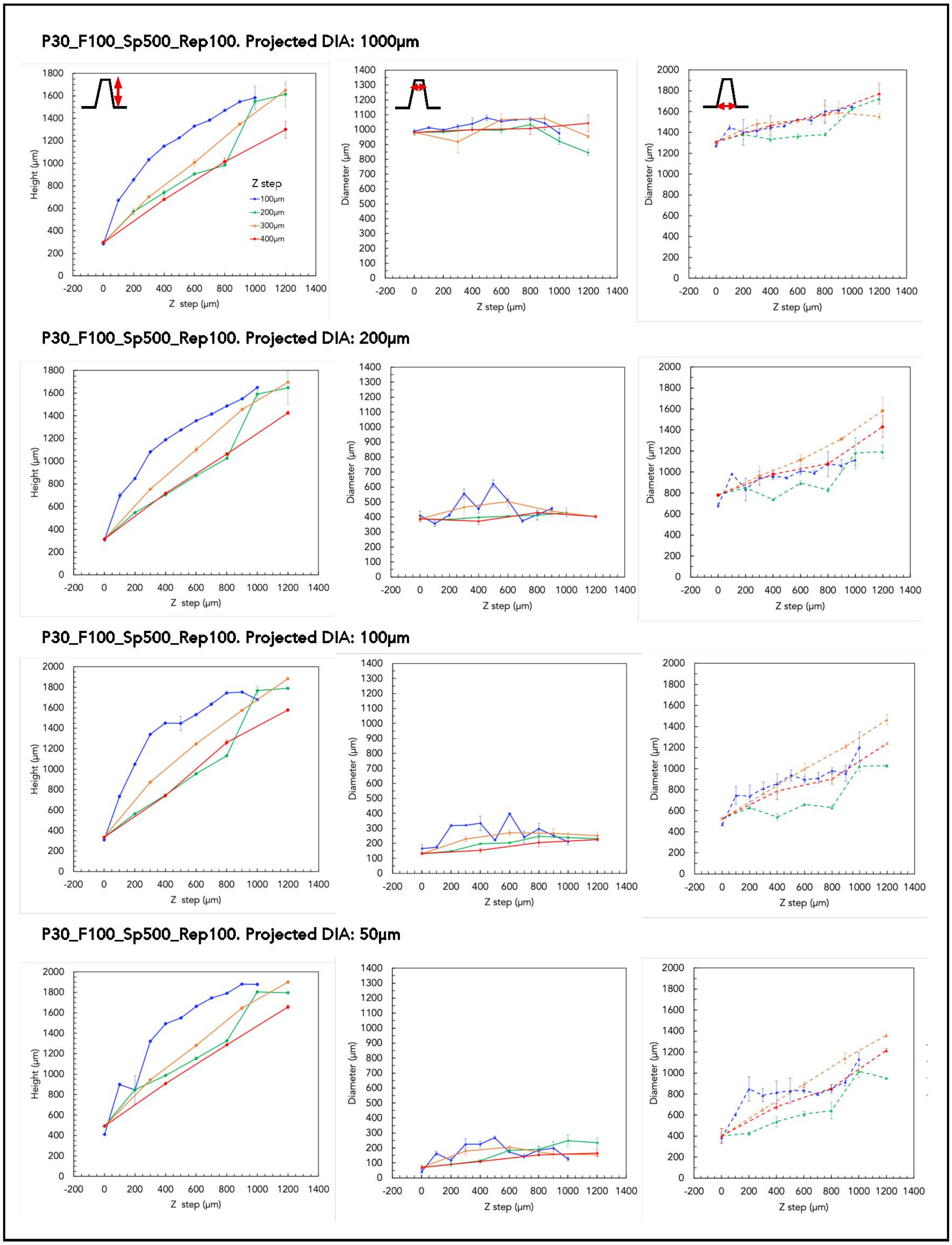

Fig. S29. Height, base and tip diameter for the MN created by ablation on stainless steel, varying the z steps (part 1).

Height (left plot), tip diameter (middle plot) and base diameter (right plot) of the MNs created by ablation at different z steps (100, 200, 300, 400 µm) with set laser parameters P30, F100, Sp500, Rep100, for different projected diameters (50, 100, 200, 1000 µm).

P: power, Sp: scanning speed, F: pulse frequency, Rep: repetitions.

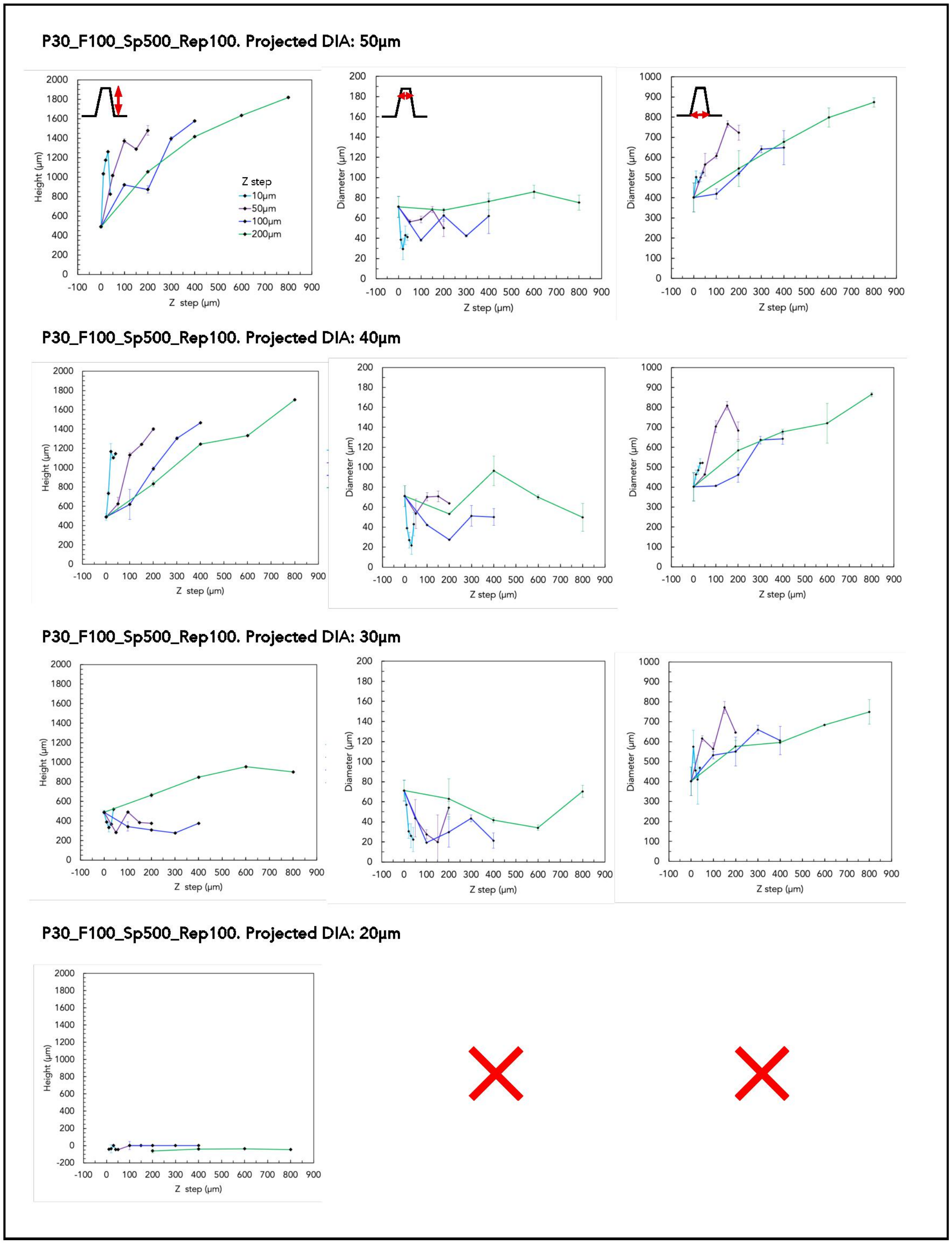

Fig. S30. Height, base and tip diameter for the MN created by ablation on stainless steel, varying the z steps (part 2).

Height (left plot), tip diameter (middle plot) and base diameter (right plot) of the MNs created by ablation at different z steps (10, 50, 100, 200 µm) with set laser parameters P30, F100, Sp500, Rep100, for different projected diameters (20, 30, 40, 50 µm). The red crosses indicate absence of detectable measurements.

P: power, Sp: scanning speed, F: pulse frequency, Rep: repetitions.

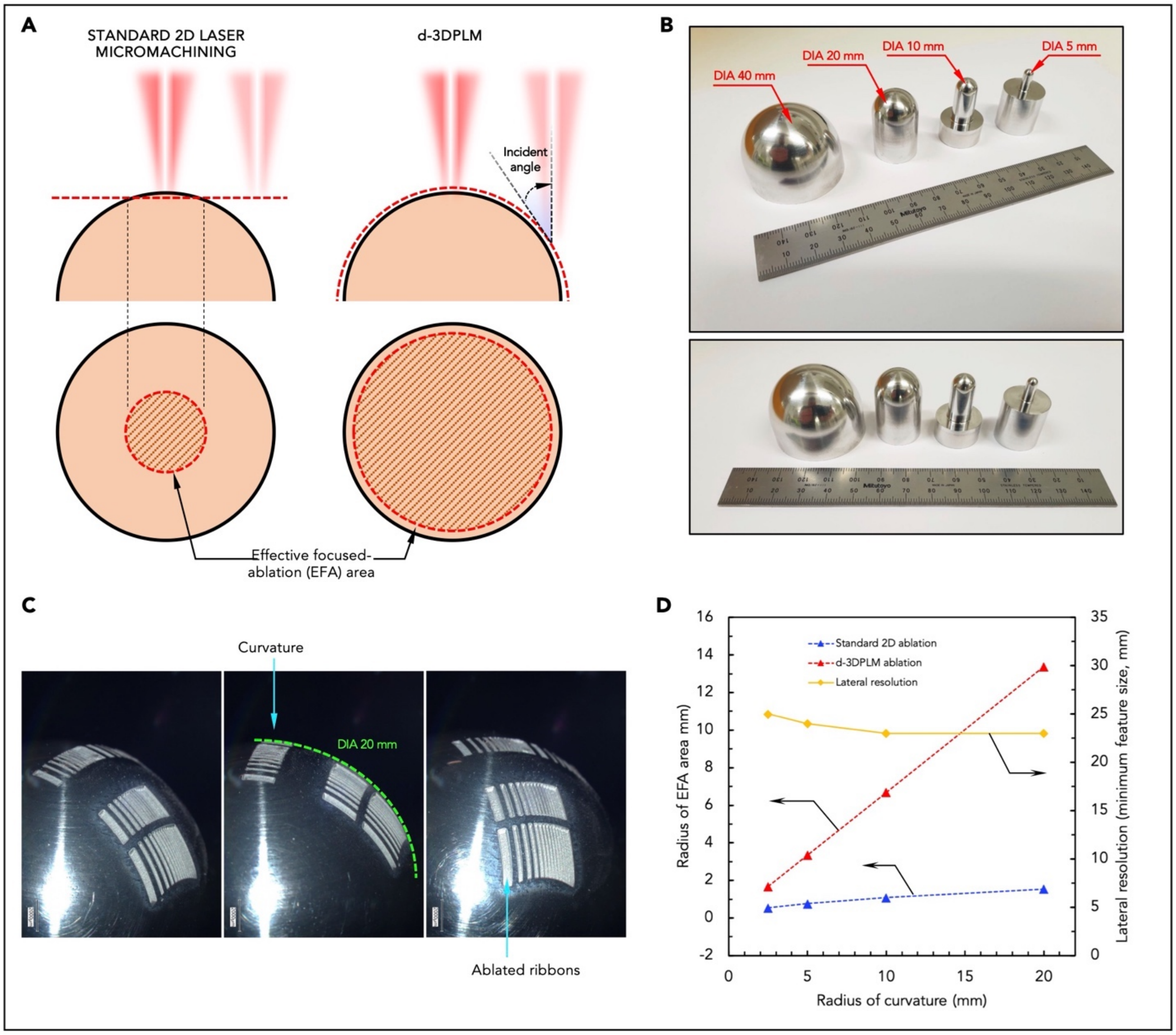

Fig. S31. Effect of substrate curvature on the lateral resolution of laser ablation and d-3DPLM.

(**A**) The scheme illustrates the difference between a standard 2D laser micromachining process and the d-3DPLM process on a curved substrate. With the first one, only a fraction of the ablation plane is in focus with the material surface, whereas with the d-3DPLM process, the focal point travels along the curved surface thus the effective focused-ablation (EFA) area is remarkably larger. (**B**) Machined stainless-steel specimens with different radii of curvature (5, 10, 20, 40 mm). (**C**) Photos of the curved specimen (DIA 20 mm) after ablation of ribbons (as used for assessing the lateral resolution on flat substrates) via d-3DPLM. The features are reproduced on different lateral positions, maintaining the lateral resolution, with a magnified ablation area. (**D**) Lateral resolution (minimum feature size) for the ribbons ablated on the surface of the curved specimens for a set of laser parameters (power 30%, scan speed 1000 mm/s, pulse frequency 100 kHz, repetitions 100). The lateral resolution is maintained, with a slight increase, with decreasing radius of curvature. The EFA area increases with the increasing radius of curvature: for standard 2D process, the EFA area depends only on the depth of focus and the radius of curvature, whereas in case of the d-3DPLM, it depends on the radius of curvature and the angular resolution, which is specific to the material (~42˚ for stainless steel, see Fig. S26).

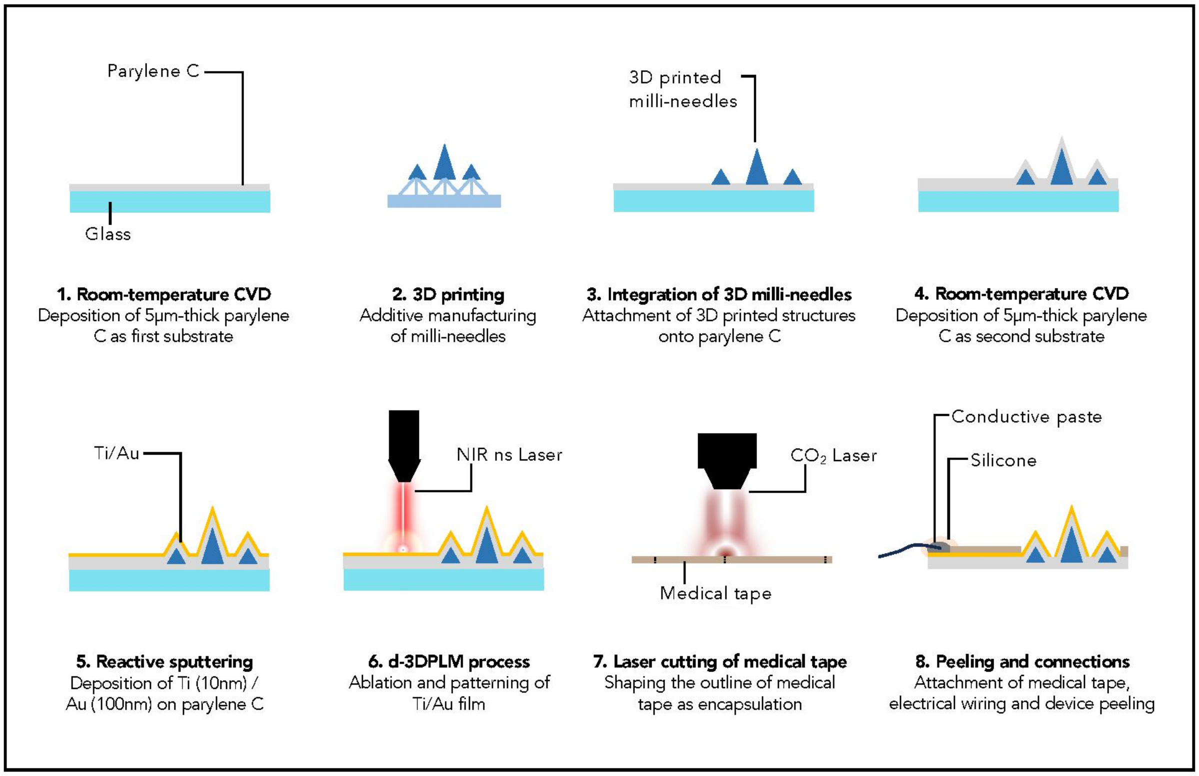

Fig. S32. Microfabrication process for the wearable electro-haptic patches.
Schematic representation of the main steps of the process flow followed to fabricate the wearable electro-haptic patches with 3D milli-needles for tactile stimulation.

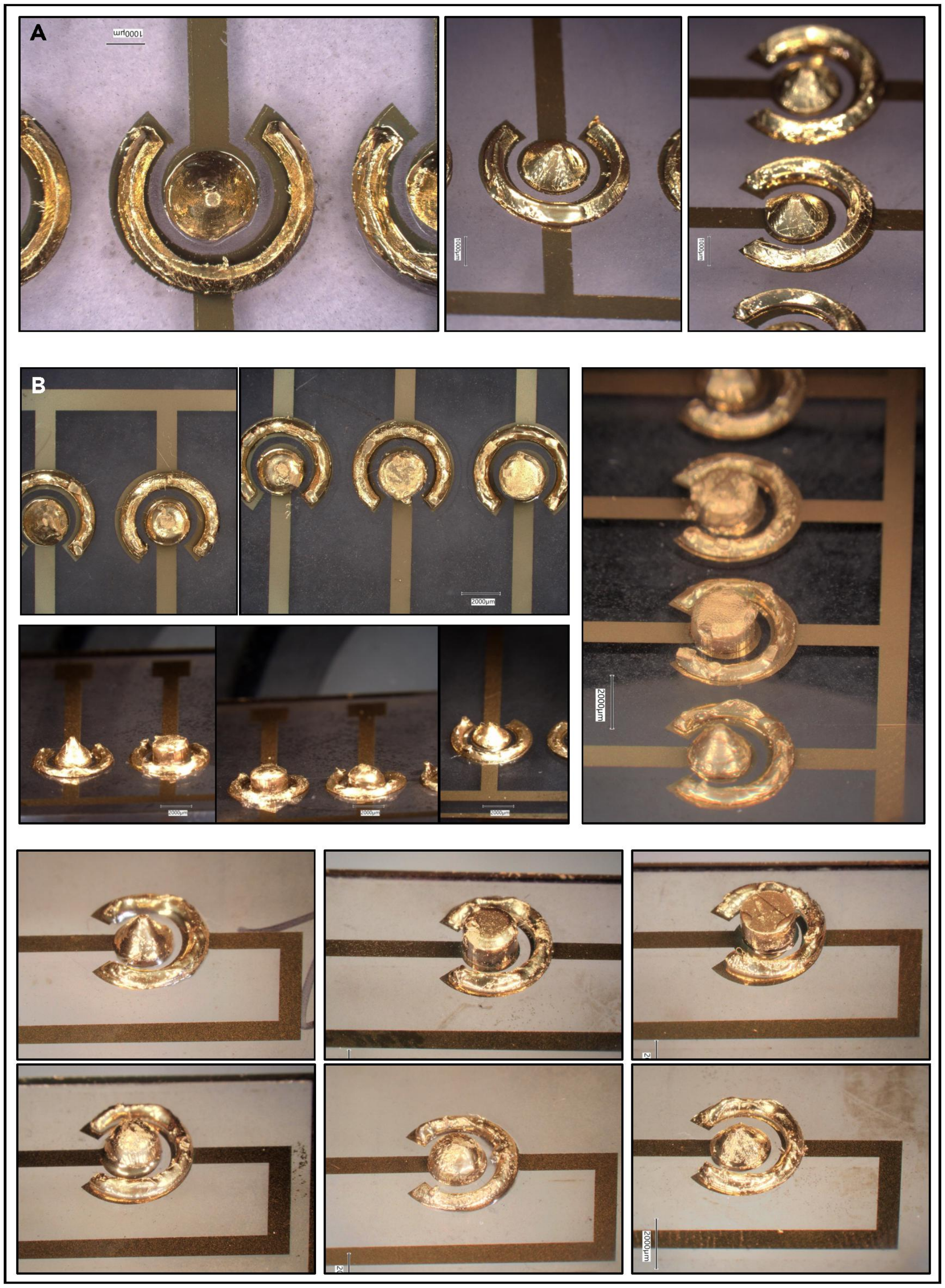

Fig. S33. Photos of the 3D electrodes for haptic stimulation.

(**A**) Optical images of the 3D electrodes fabricated for haptic stimulation based on an active electrode with conical shape and an outer ring as ground electrode. (**B**) Photos of different 3D shapes for the electrodes fabricated for haptic stimulation: cone, dome, cylinder.

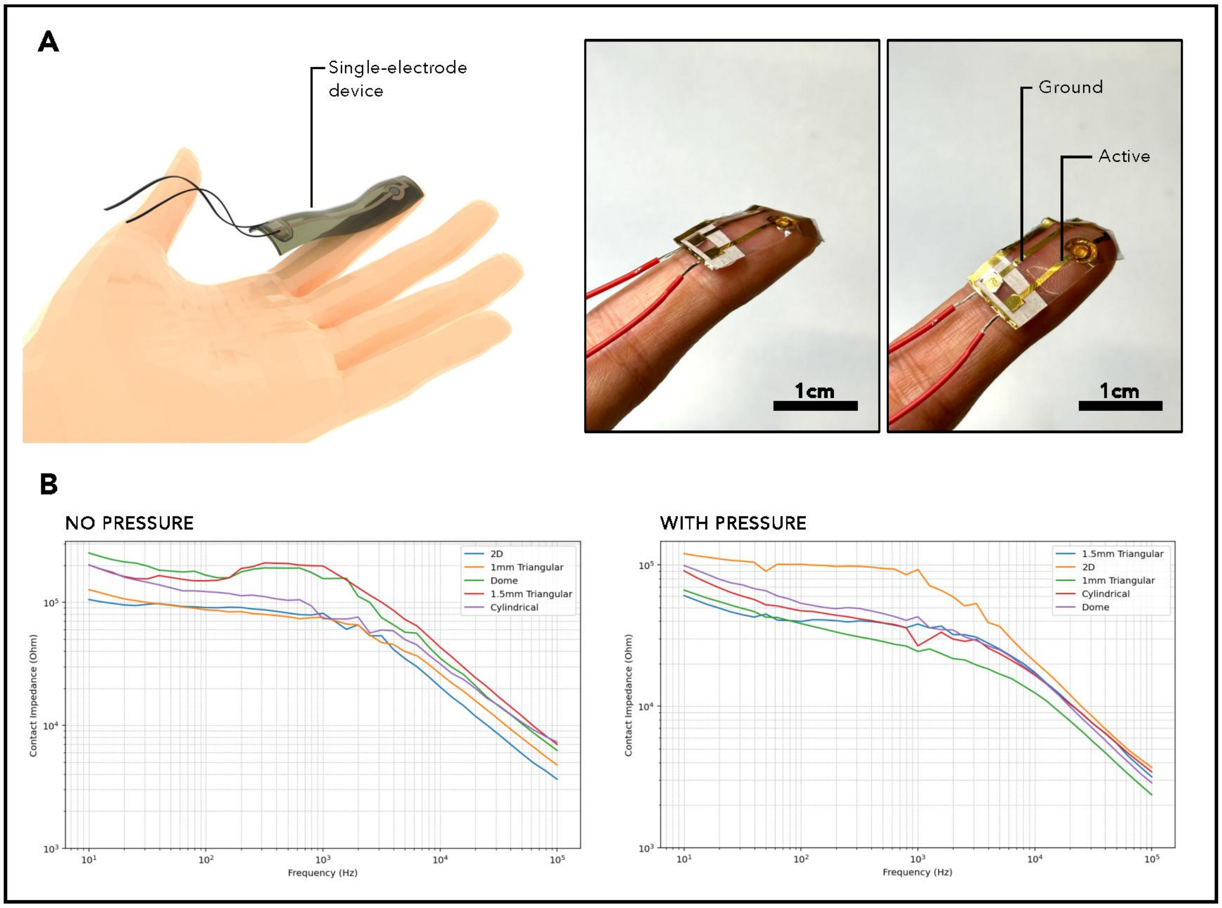

Fig. S34. Single-electrode electro-haptic devices applied on the volunteers' finger.

(**A**) Illustration and photo of the single-electrode electro-haptic device applied on the finger. (**B**) Contact dry impedance of the single-electrode electro-haptic devices applied on the finger without (left) or with (right) pressure, for different electrode 3D shapes: 2D, 1mm-height cone, dome, 1.5mm-height cone, cylindrical.

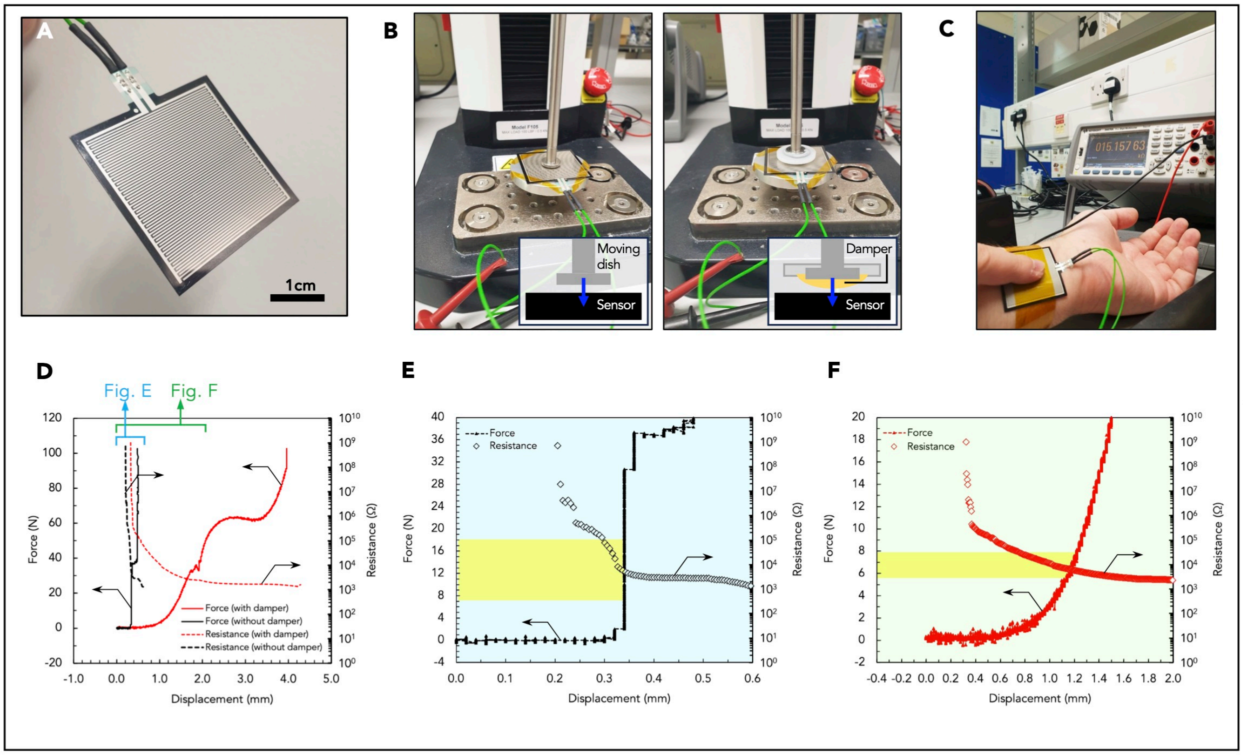

Fig. S35. Measurement of the applied pressure used to characterize the electro-haptic patches.

(**A**) Commercial thin-film pressure sensor used to quantify the pressure applied onto the electro-haptic devices. (**B**) Setup used to calibrate the commercial pressure sensor, consisting of a mechanical uniaxial tester in compression mode, with a moving disk-like probe (diameter ~1cm), without or with a damper; the damper simulates the softness of the finger skin and allows a slower transfer of mechanical loads. (**C**) Resistance measured when the pressure sensor is worn on the wrist, as for the electro-haptic 4-electrode patches. (**D**) Force-displacement and Resistance-displacement curves for the commercial pressure sensor, acquired without (black curves) and with (red curves) damper. (**E, F**) Force-displacement and resistance-displacement curves for the commercial pressure sensor tested without (**E**) and with (**F**) damper. Testing the sensor with damper allows to obtain less steep curves enabling a more accurate identification of the real pressure applied in the case of the electro-haptic patches. Resistances in the range of 1-100 kΩ were detected with the commercial pressure sensor used simultaneously with the electro-haptic patches on top. These resistances corresponded to applied forces of 5-8 N, and average pressures of 50-80 kPa.

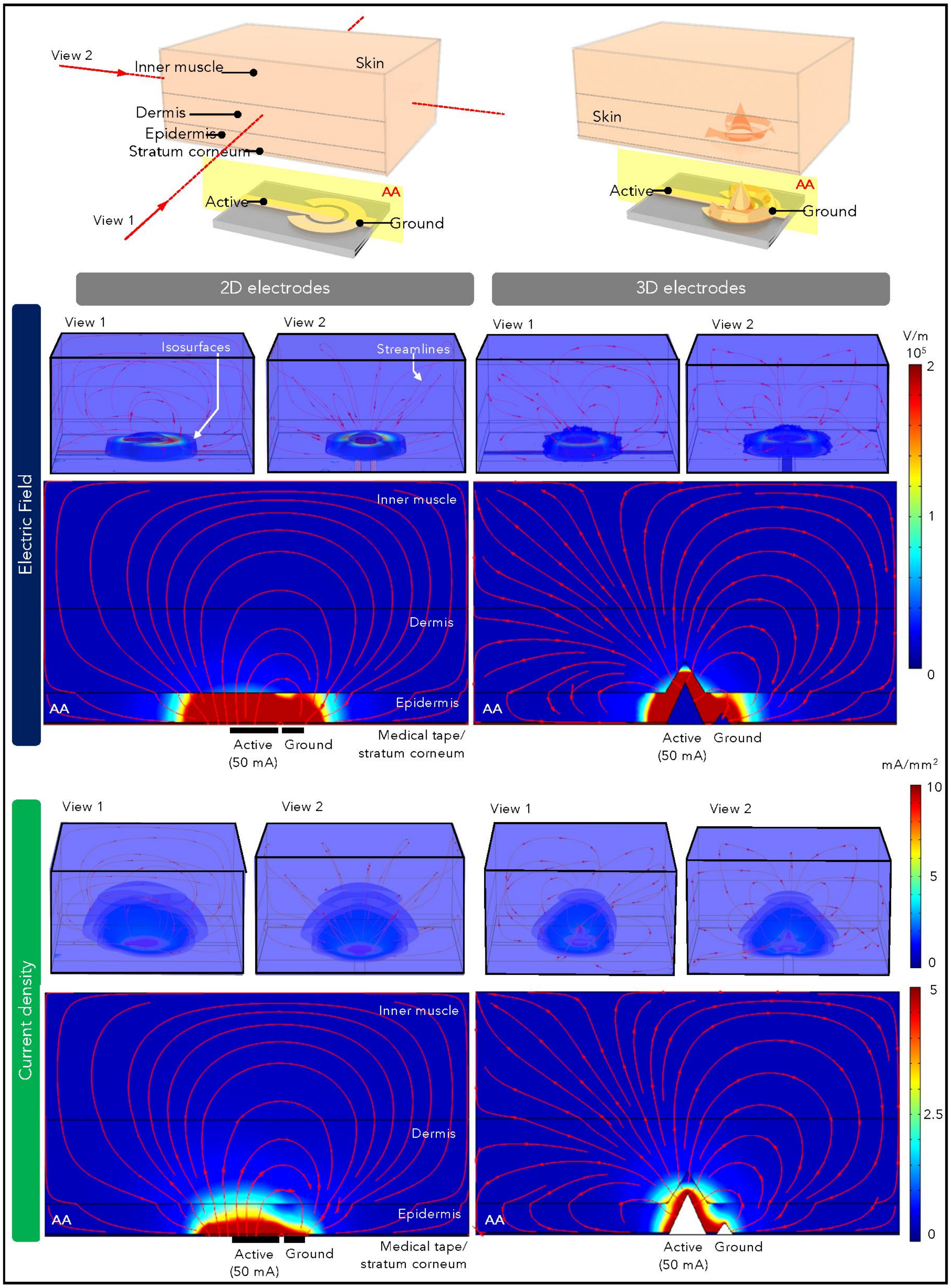

Fig. S36. FEM simulations of electrical stimulation performed with 2D and 3D electrodes. Comparison between 2D and 3D electrodes in terms of simulated electric field and current density distributions within a multilayer model of the skin. With 3D electrodes, the epidermis deformation is taken into account in the model, without any perforation.

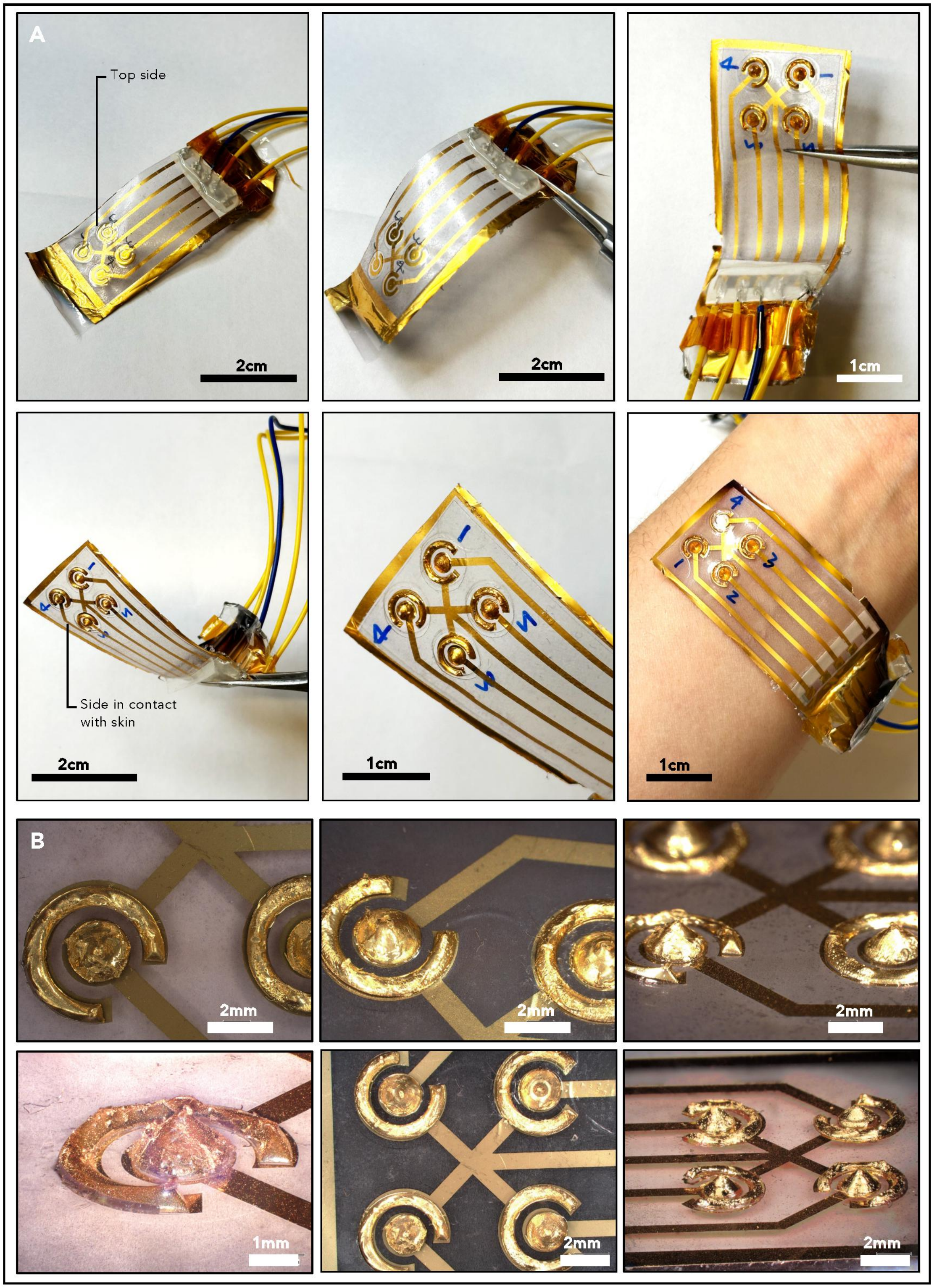

Fig. S37. Flexible electro-haptic devices based on four electrodes.

(**A**) Photos of a flexible four-electrode electro-haptic device with electrodes numbered from 1 to 4. The device is also wrapped around the wrist of a volunteer. (**B**) Magnified images of the electrodes in the four-electrode electro-haptic devices.

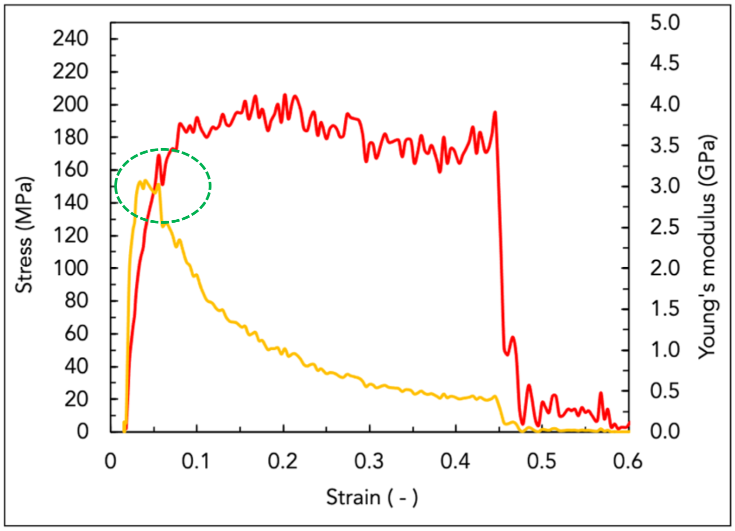

Fig. S38. Tensile tests of the flexible electro-haptic patches.

Representative stress-strain curve (red) and Young's modulus (yellow) of one flexible electro-haptic device (n=3). The dotted circle indicates the value of the Young's modulus extracted from the stress-strain curve.

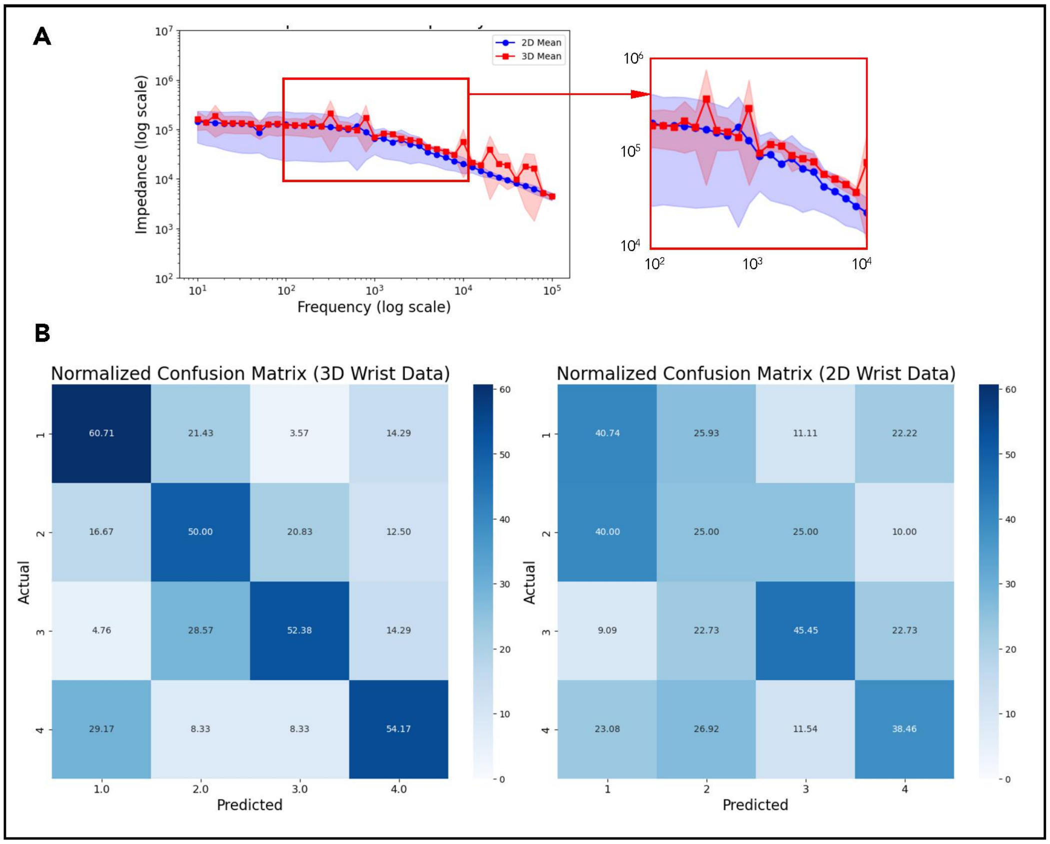

Fig. S39. Dry impedance spectroscopy of the electrodes used in the electro-haptic devices.

(**A**) Bode plots of the impedance magnitude of the electrodes (2D and 3D) in the electro-haptic devices in dry contact with the skin. (**B**) Heatmaps (normalized confusion matrixes), for 2D and 3D electrodes, of the stimulation threshold for the four electrodes activated singularly.

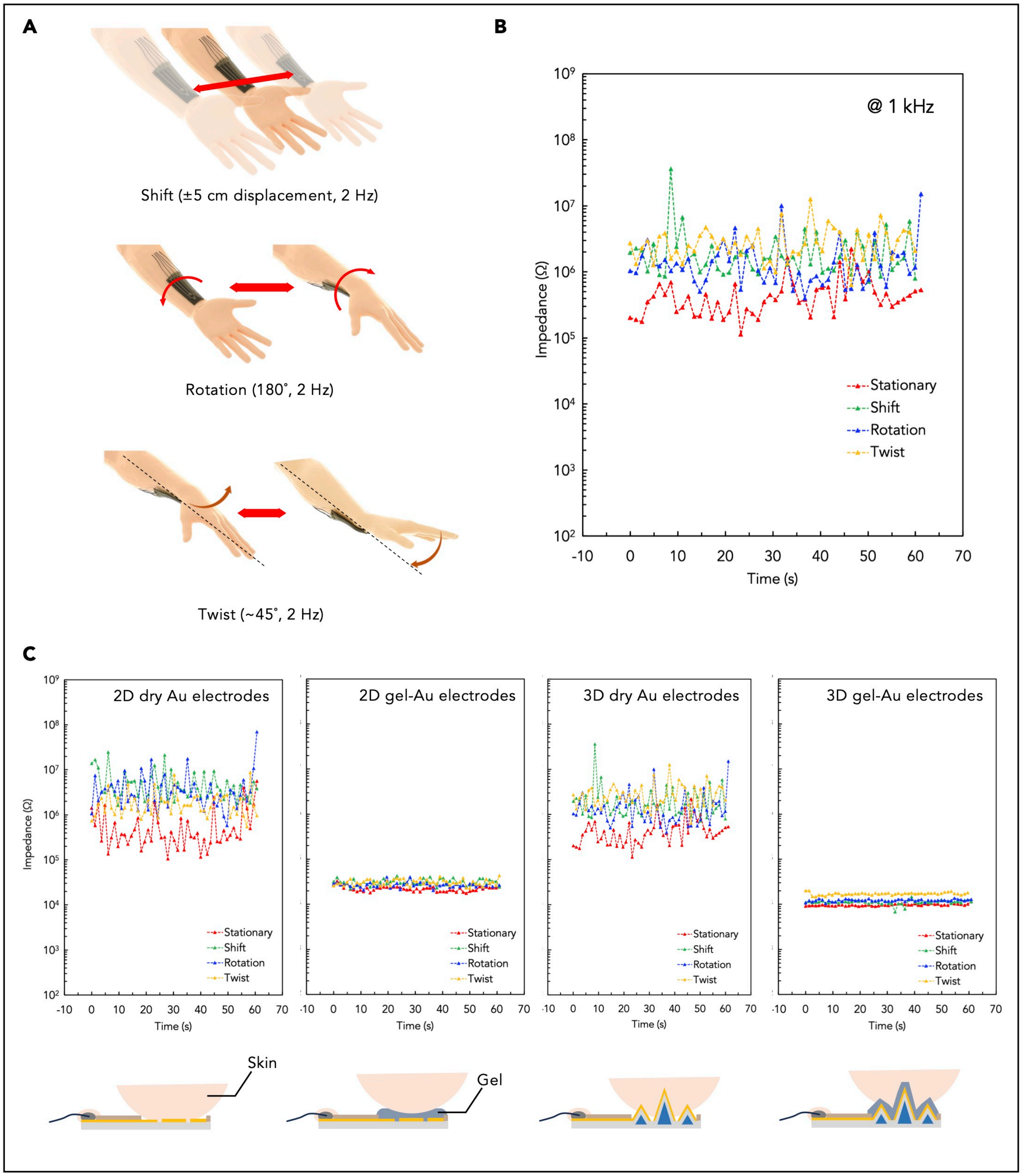

Fig. S40. Performance of 3D electrodes against motion artifacts.

Contact impedance (at 1 kHz) over time for 3D electrodes worn on the participant's wrist during controlled movements, i.e., shift, rotation and twist (illustrations in (**A**). The plot in (**B**) shows the impedance over time compared to the device in stationary state: based on the impedance stability during the movements, the devices exhibit robustness against motion artifacts. (**C**) Contact impedance (at 1kHz) over time for 2D dry, 2D gel-coated, 3D dry, 3D gel-coated electrodes worn during controlled movements (same as in (A)).

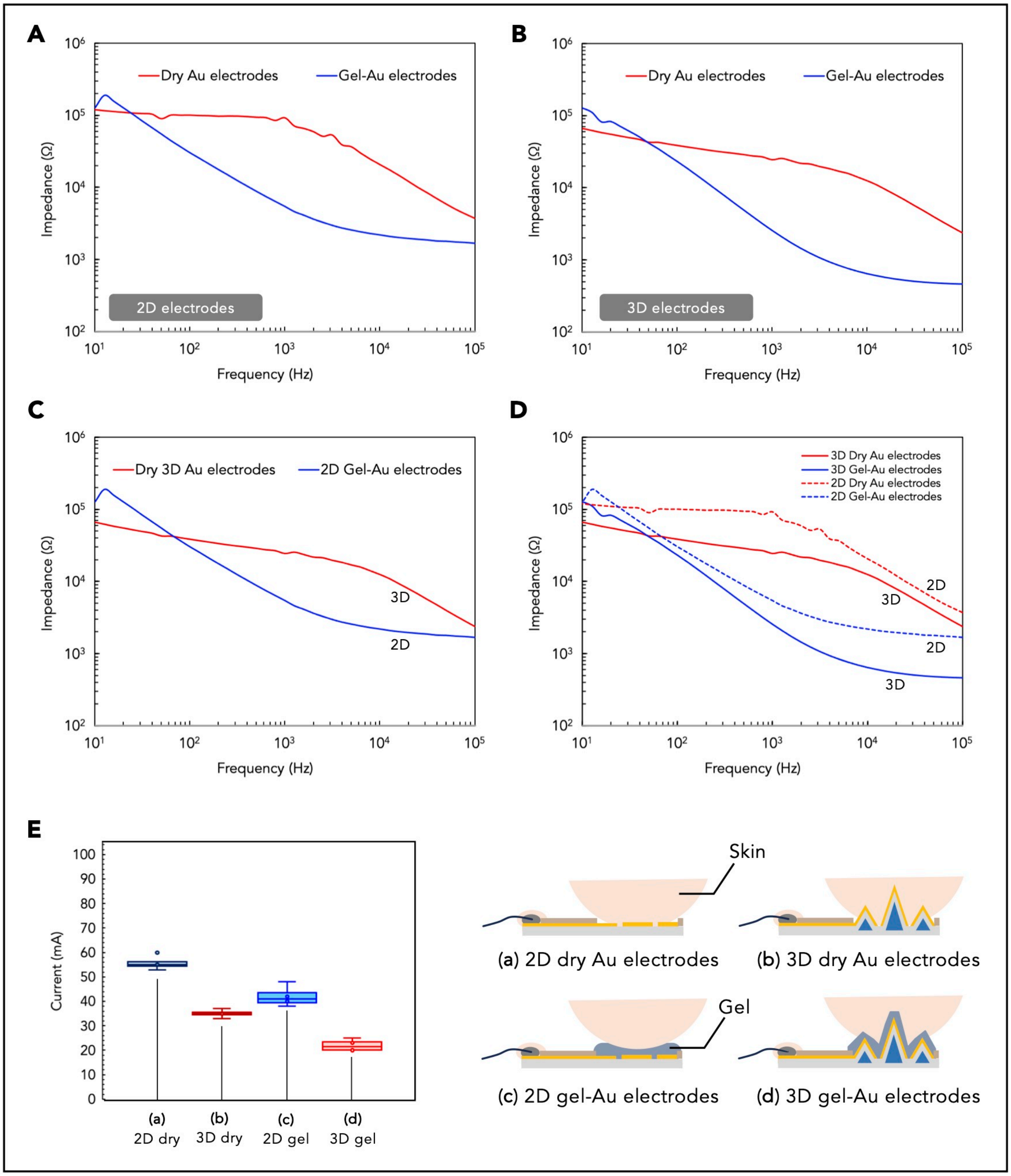

Fig. S41. Comparison between 2D and 3D electrodes with and without skin gel.

(**A-D**) Contact impedance spectroscopy, comparison between 2D dry Au electrodes and 2D gel-Au electrodes (**A**); 3D dry Au electrodes and 3D gel-Au electrodes (**B**); 3D dry Au electrodes and 2D gel-Au electrodes (**C**); 2D and 3D electrodes, with or without gel (**D**). (**E**) Current threshold for 3D gel-Au electrodes, compared to the current thresholds of the 2D gel-Au electrodes and of the dry (2D and 3D) electrodes, tested on a single participant. The 3D dry electrodes exhibit lower current threshold (35.0±1.6 mA) compared to the 2D gel-Au electrodes (42.0±4.3 mA). The addition of the gel onto the 3D electrodes allows to reduce further the current threshold (22.0±2.4 mA).

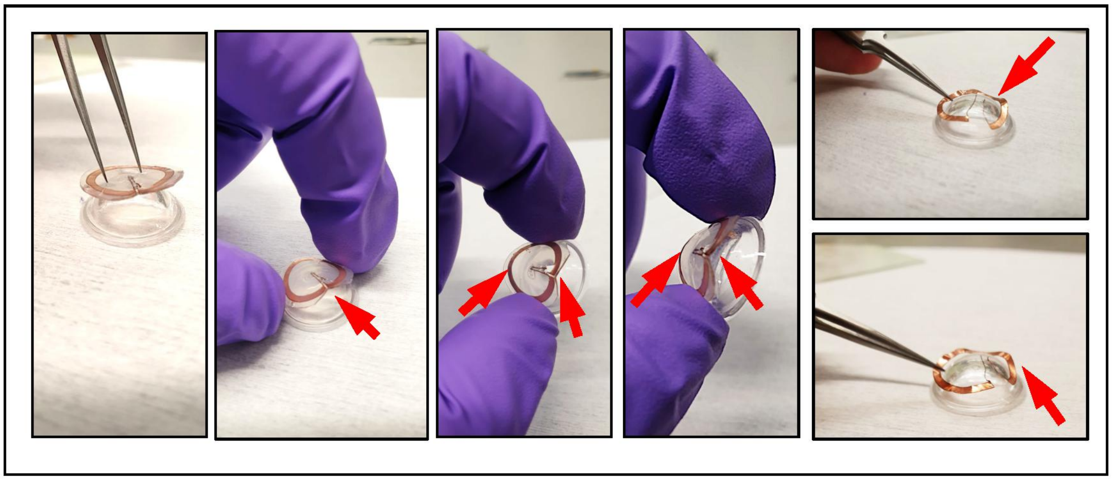

Fig. S42. Conformality issues for flat inductive coils for contact lenses.
The images show that a, inductive coil fabricated in a flat configuration does not offer an optimal conformality when used for wireless contact lenses and placed on a preformed elastomeric shape of a contact lens.

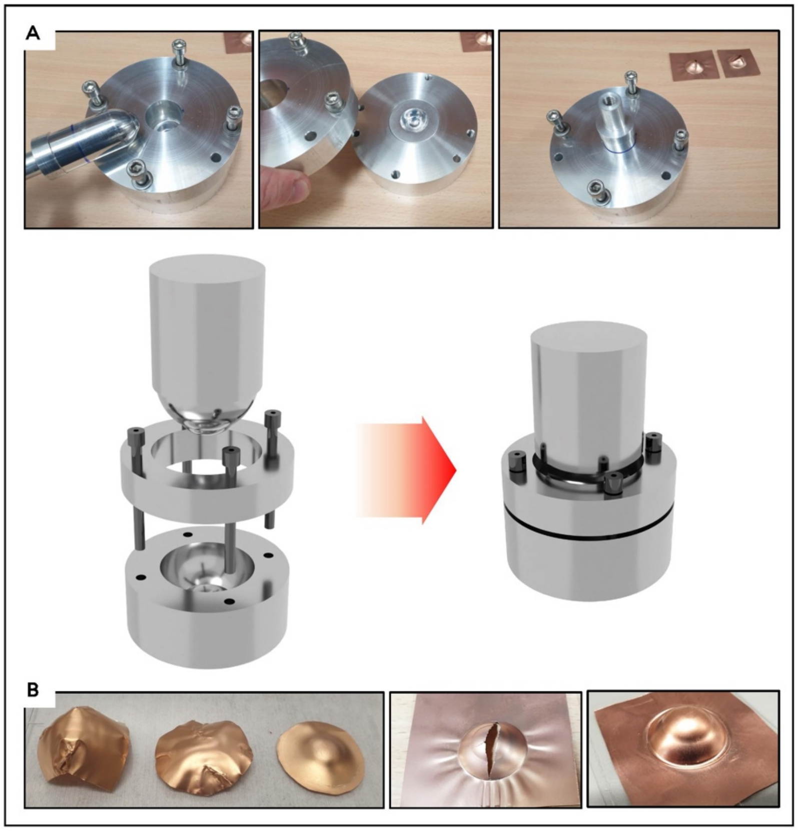

Fig. S43. Setup for thermoforming Cu foils and produce the target eye-bulb profile.
The customized mechanical setup is shown in (**A**), consisting of a piston with the eye-bulb profile and two cylinders to keep the Cu foil in place during the thermoforming step. Several trials with different types of Cu laminates yielded different non-optimal results, leading eventually to select the best material option (**B**).

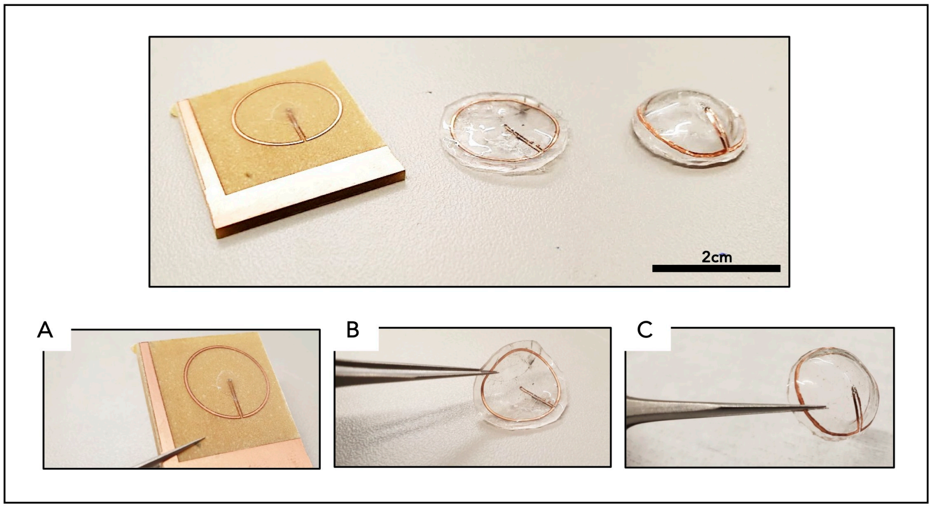

Fig. S44. Comparison between flat rigid, flat flexible, curved flexible wireless contact lenses. The photos show the different conformality offered by flat rigid (**A**), flat flexible (**B**) and curved flexible (**C**) wireless contact lenses. The device in (**A**) is laser patterned onto an FR4 substrate, whereas the other two (**B, C**) are based on Cu coils encapsulated in PDMS.

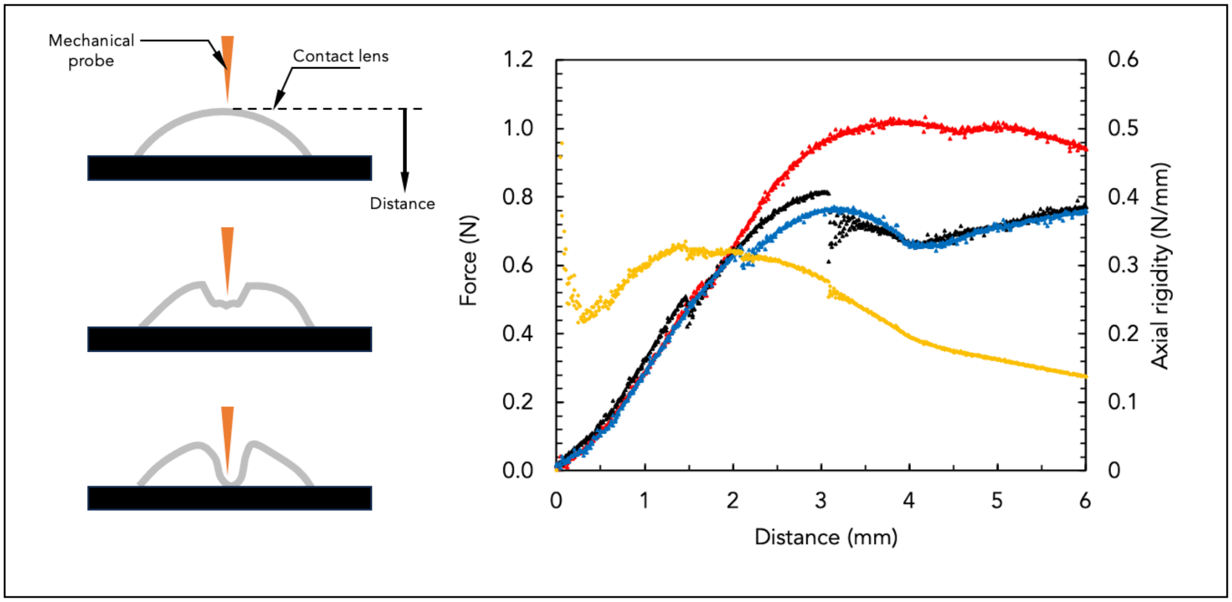

Fig. S45. Mechanical compression test on the contact lenses.
Uniaxial compression test on the contact lens: the scheme shows the mechanical probe deforming the lens downwards. The plot shows the force vs distance curves for three samples (red, blue, black) and the average axial rigidity (yellow).

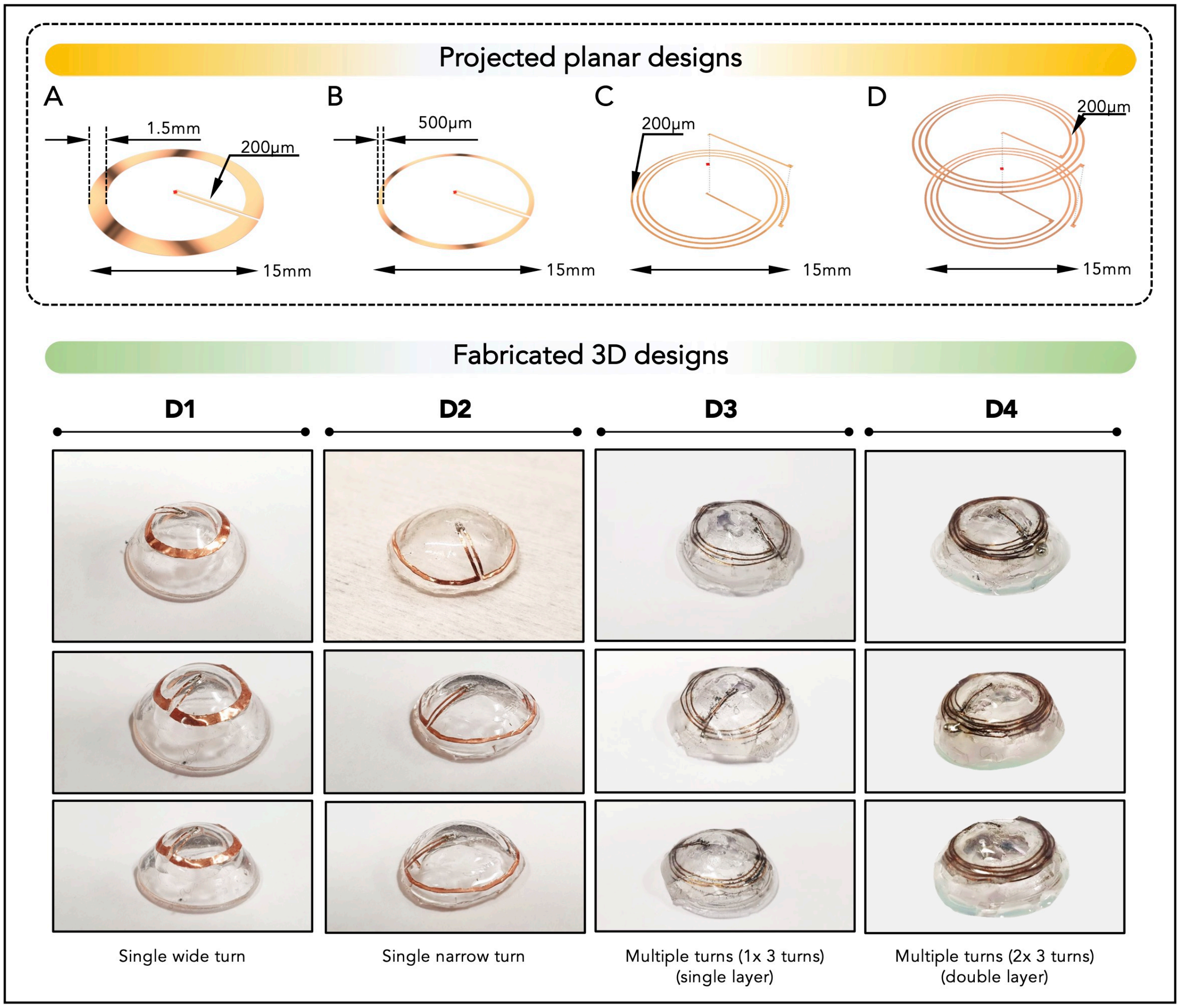

Fig. S46. Different designs for the inductive coils fabricated by d-3DPLM.

Four representative designs were selected to validate the d-3DPLM process for the microfabrication of the wireless contact lenses: single wide turn (**A**), single narrow turn (**B**), single-layer 3-turn (**C**), double-layer 2x3-turn (**D**). The designs were named respectively D1, D2, D3, D4.

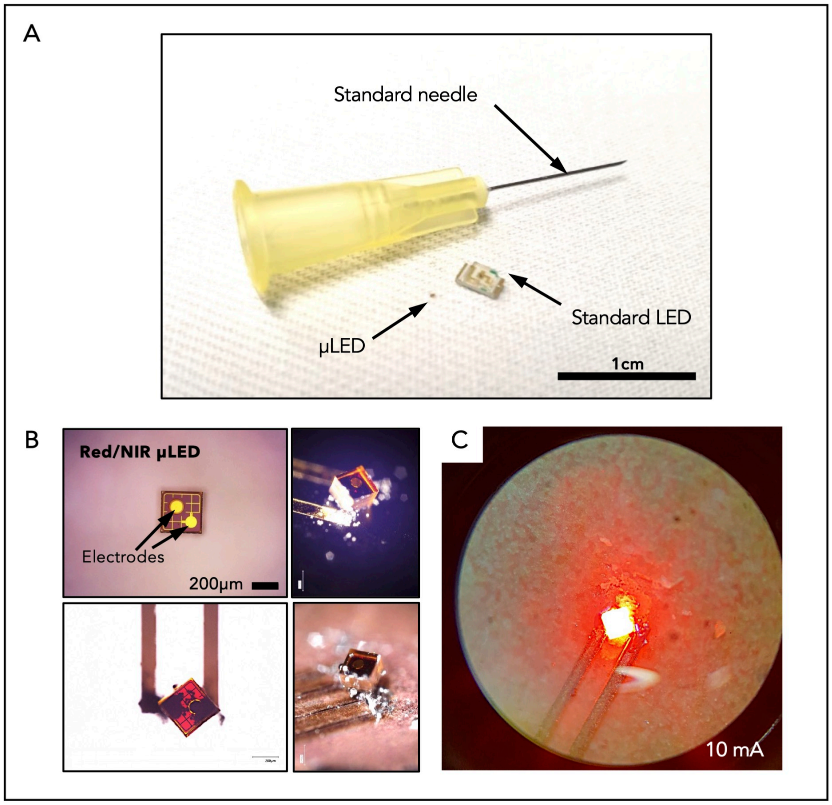

Fig. S47. Red/NIR µLED used for the microfabrication of the wireless contact lenses.

The µLED used for the wireless contact lenses are much smaller than standard LEDs (**A**). The optical images in (**B**) show the µLED soldered onto the electrodes of the coil within the contact lens. The red illumination under the application of a 10mA current is visible in (**C**).

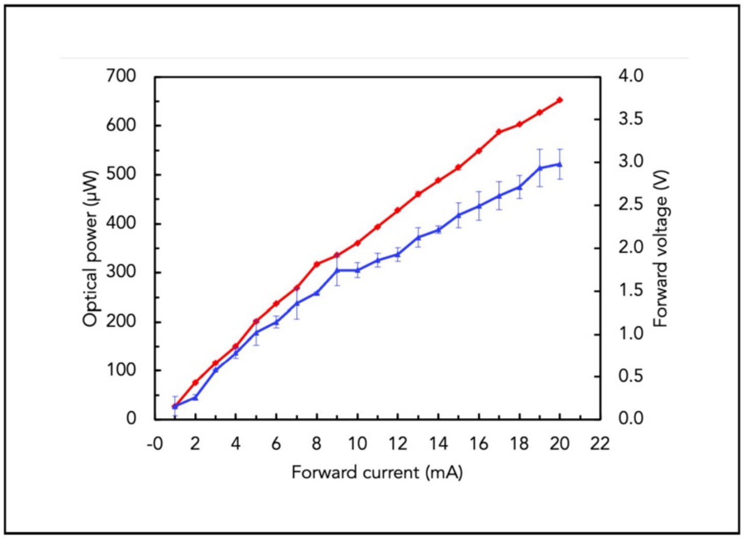

Fig. S48. Characterization of the red/NIR µLED used for the microfabrication of the wireless contact lenses.

Optical power and forward voltage of the µLEDs as functions of the forward DC current applied at their terminals.

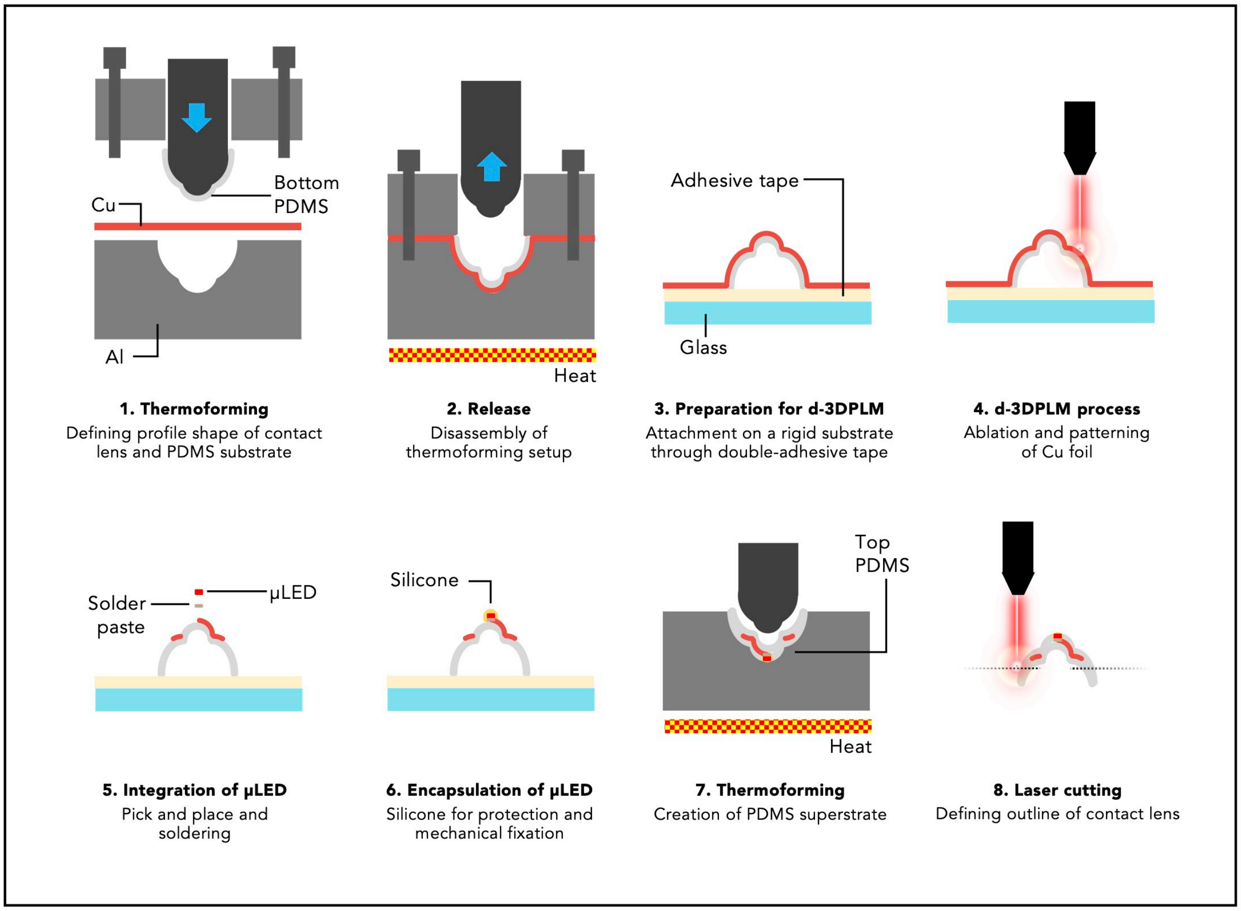

Fig. S49. Microfabrication process for the wireless µLED-integrated contact lenses.
Schematic representation of the main steps of the process flow followed to fabricate the wireless µLED-integrated contact lenses.

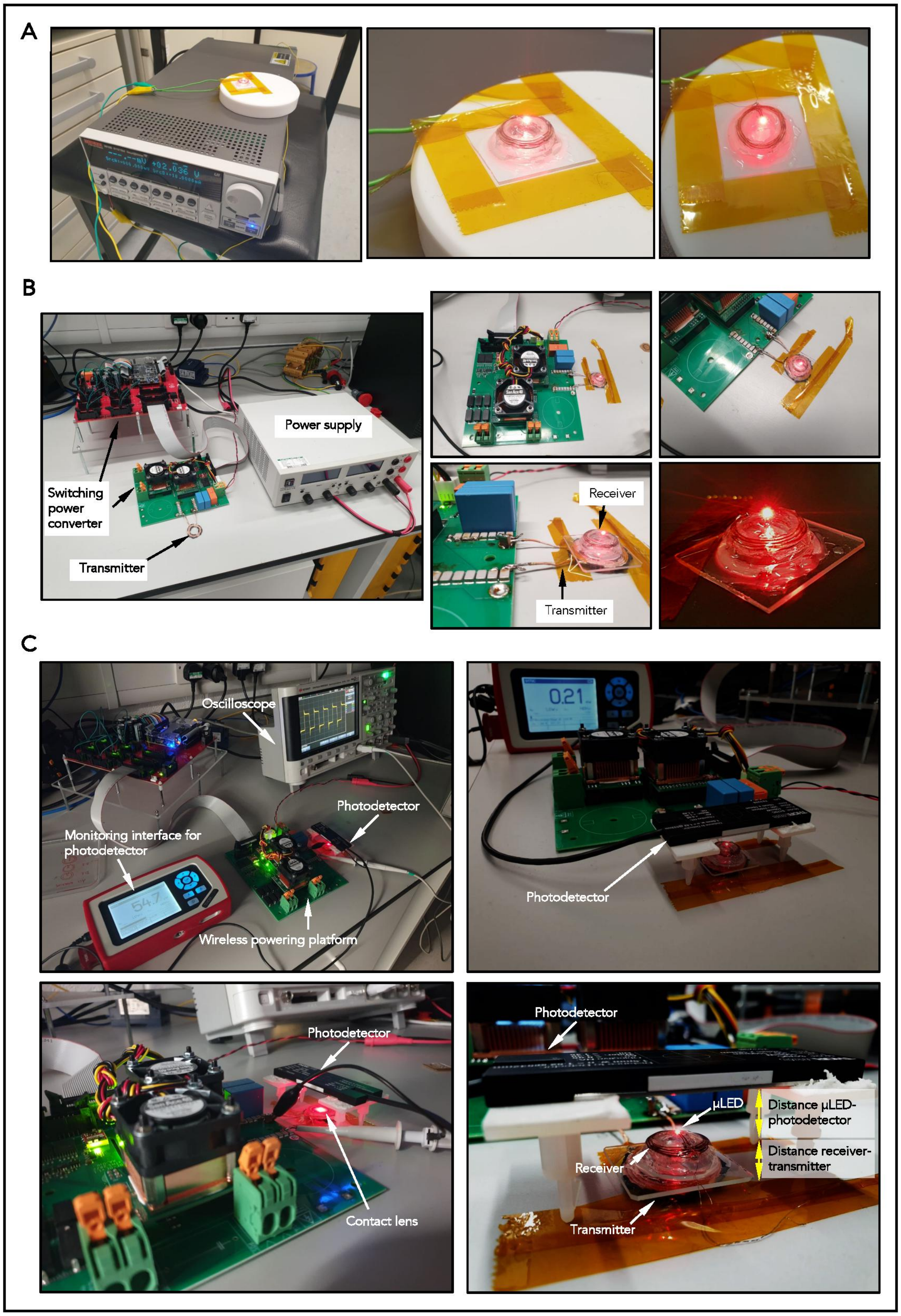

Fig. S50. Wired and wireless operation of the contact lenses.

The microfabricated contact lenses were tested in a wired configuration (**A**), using a Keithley source-measure unit, and in a wireless configuration, whose setup is shown in (**B**). (**C**) Setup used for characterizing the light intensity emitted by the µLED during wireless operation. The setup included a photodetector placed on top of the µLED.

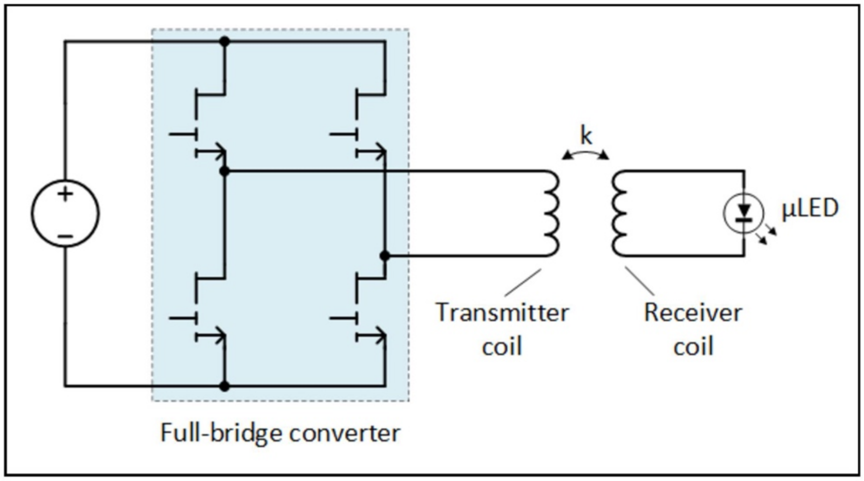

Fig. S51. Circuital scheme of the wireless powering setup for the contact lenses.

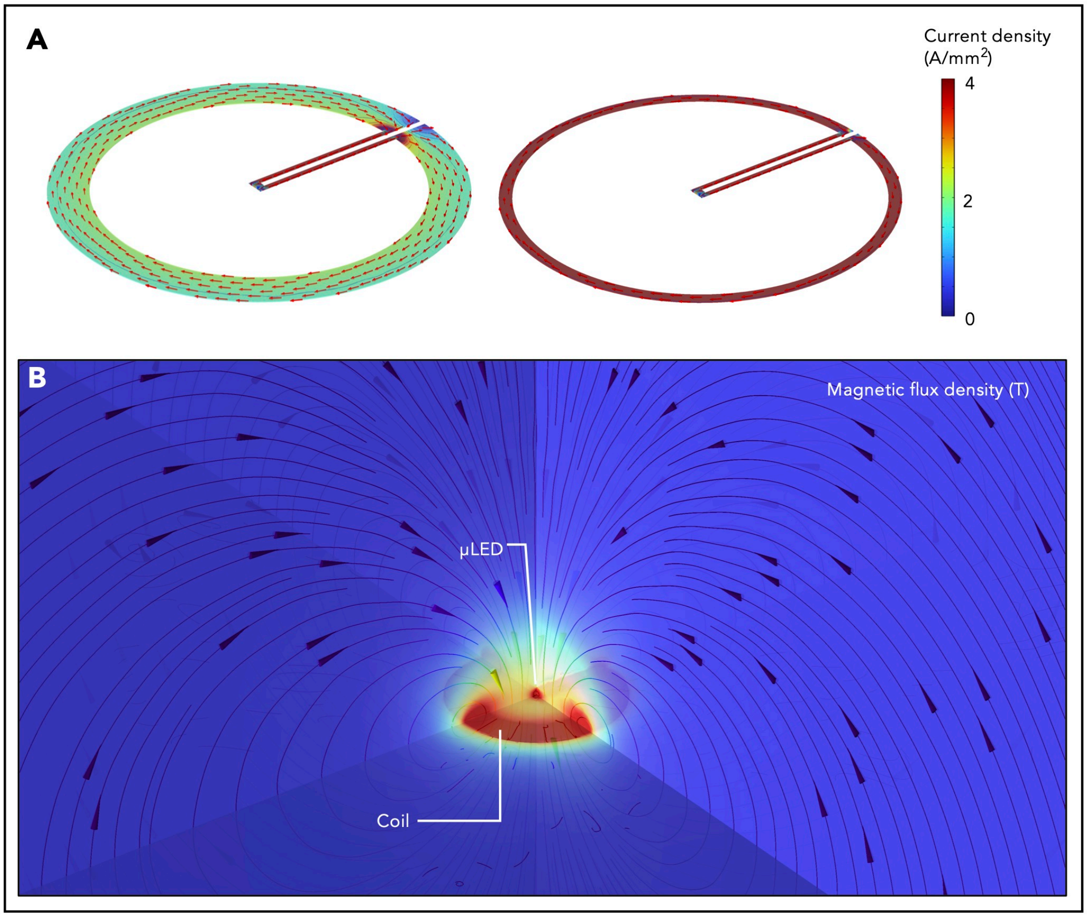

Fig. S52. FEM simulations of the current density and magnetic flux generated by the inductive coils.

(**A**) Current density distribution for two designs of the inductive coils, i.e., single wide turn and single narrow turn (planar configuration). (**B**) Magnetic flux distribution around the inductive coil under wireless operation.

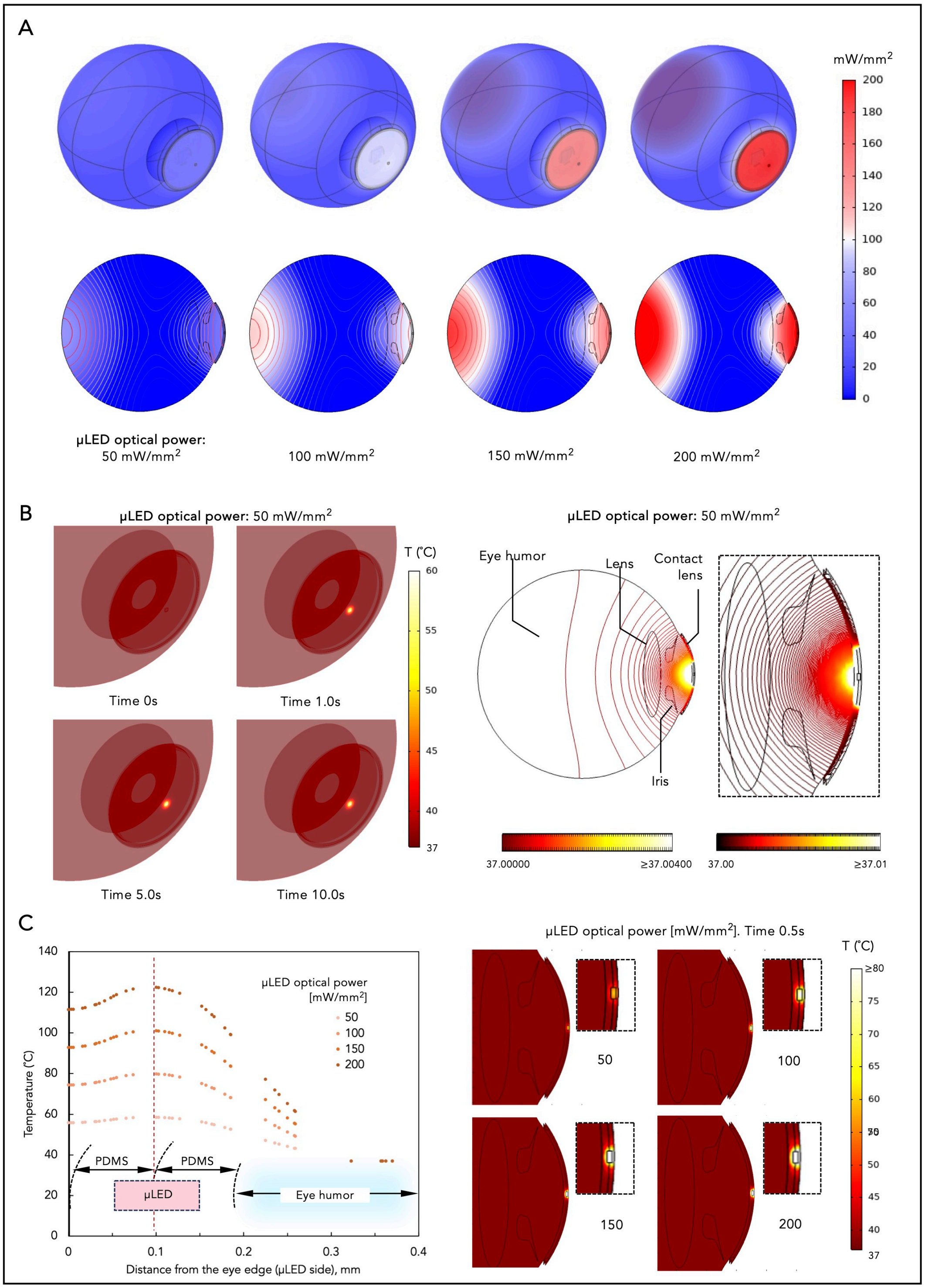

Fig. S53. FEM simulations of the µLED light intensity and temperature distribution when integrated on the contact lens.

(**A**) Light intensity distribution within the eye bulb for different optical power densities emitted by the µLED. (**B**) Temperature distribution within the eye bulb induced by the optical emission of the µLED (with the set power density of 50 mW/mm^2) at different time steps. (**C**) Temperature distribution within the eye bulb at 0.5s after the µLED activation, with different emitted optical power densities. The plot shows the temperature distribution along the longitudinal axis of the eye bulb.

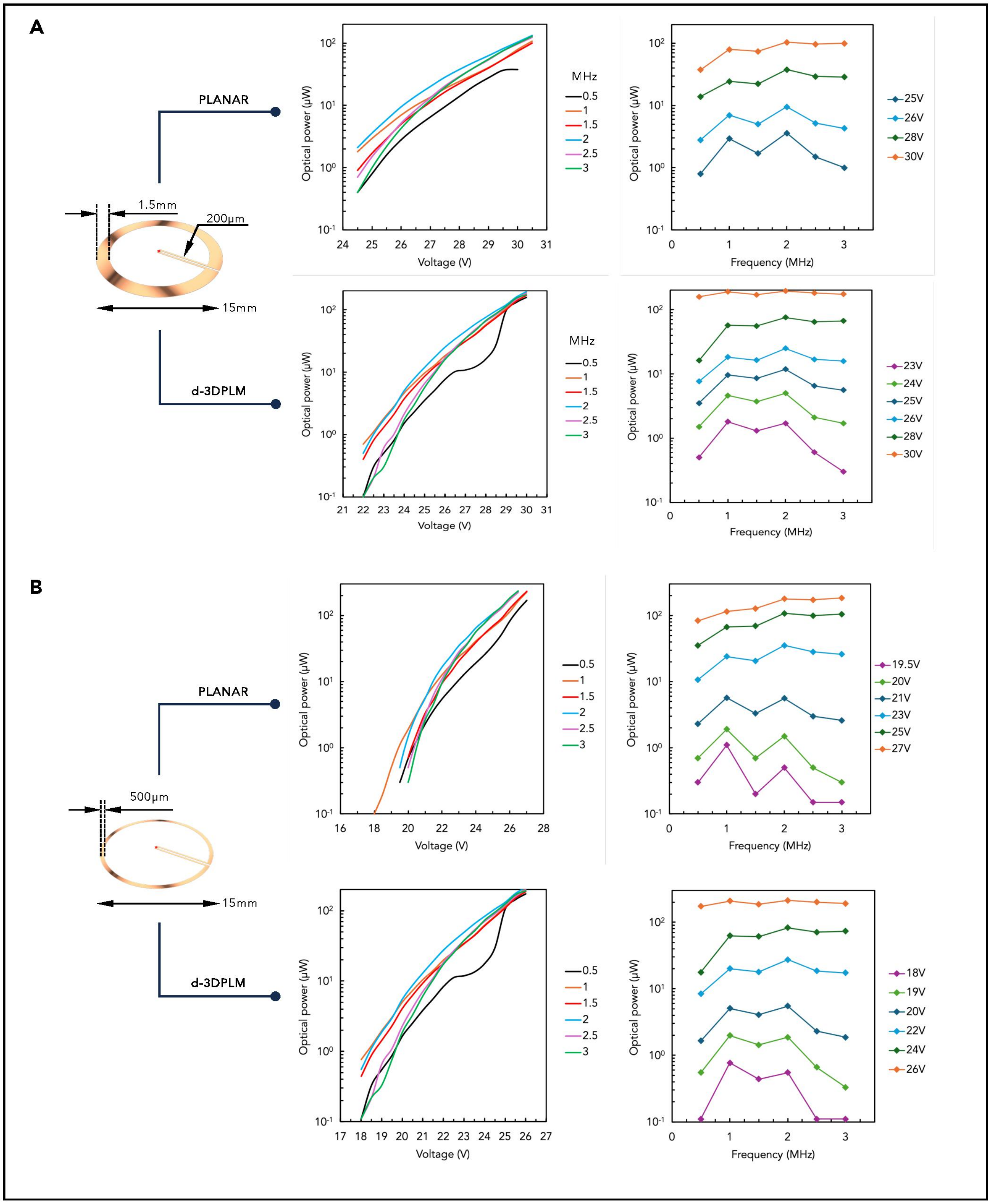

Fig. S54. Characterization of the wireless contact lenses (part 1).

Optical power emitted by the µLED integrated into the receiver coil, as a function of the applied voltage and the AC frequency of the input signal in the wireless powering setup through the transmitting coil. The results are shown for (**A**) design D1 and (**B**) deign D2. The planar and d-3DPLM coils are compared.

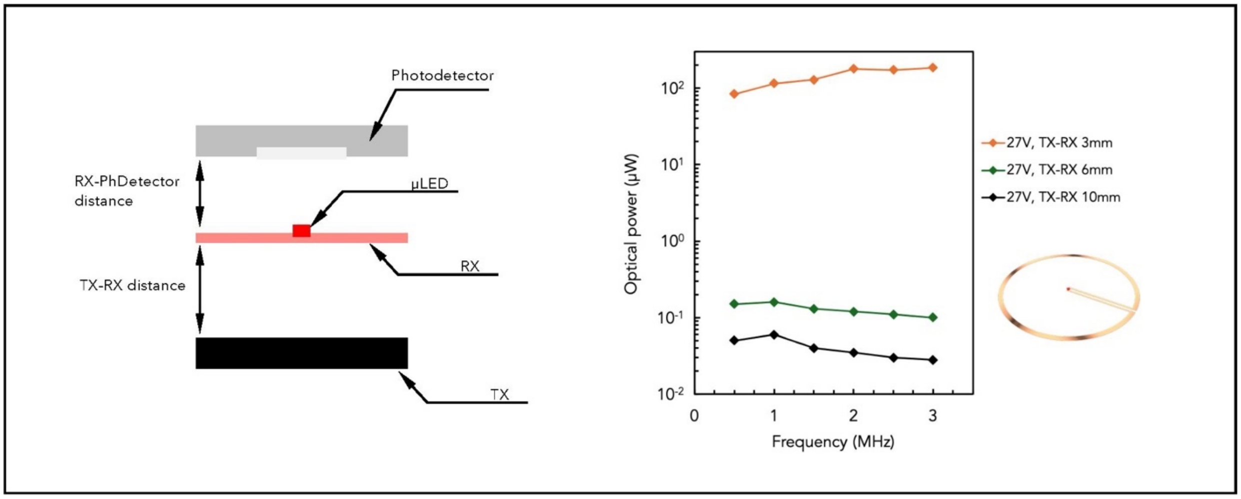

Fig. S55. Relative distances between transmitting and receiving coils during the wireless powering characterization.

Left: illustration of the relative distances between the receiving coil (with the µLED) and the photodetector (RX-PhDetector), and between the receiving coil and the transmitting coil (TX-RX). Right: optical power emitted by the µLED integrated into the receiver coil, as a function of the AC frequency, at 27V applied voltage, for the design D2, at three different TX-RX distances.

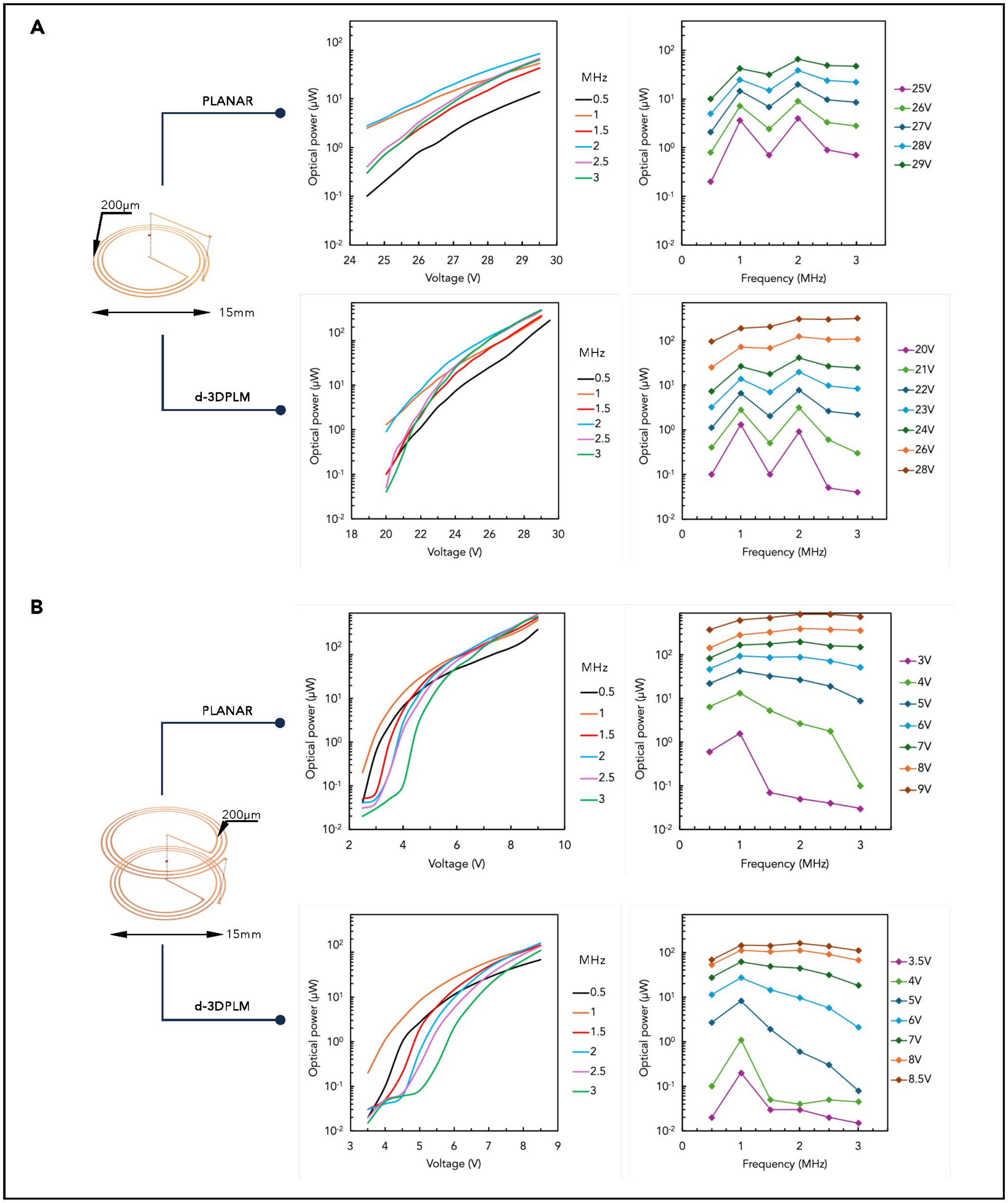

Fig. S56. Characterization of the wireless contact lenses (part 2).

Optical power emitted by the μLED integrated into the receiver coil, as a function of the applied voltage and the AC frequency of the input signal in the wireless powering setup through the transmitting coil. The results are shown for (**A**) design D3 and (**B**) deign D4. The planar and d-3DPLM coils are compared.

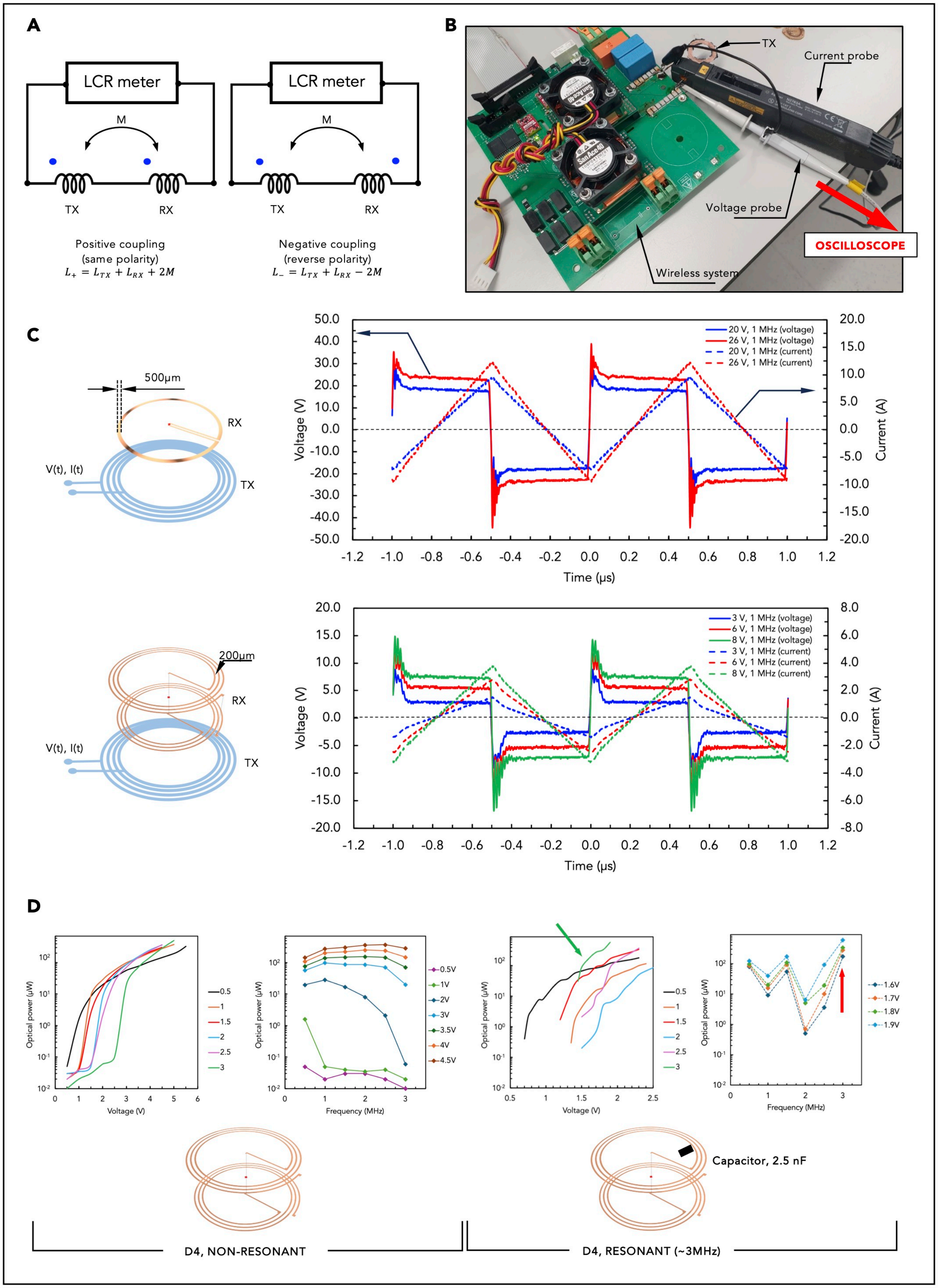
A
LCR meter
M
TX
RX
Positive coupling
(same polarity)
$L_+ = L_{TX} + L_{RX} + 2M$
Negative coupling
(reverse polarity)
$L_- = L_{TX} + L_{RX} - 2M$
B
TX
Current probe
Voltage probe
OSCILLOSCOPE
Wireless system
C
500μm
RX
V(t), I(t)
TX
20 V, 1 MHz (voltage)
26 V, 1 MHz (voltage)
20 V, 1 MHz (current)
26 V, 1 MHz (current)
Voltage (V)
Current (A)
Time (μs)
200μm
3 V, 1 MHz (voltage)
6 V, 1 MHz (voltage)
8 V, 1 MHz (voltage)
3 V, 1 MHz (current)
6 V, 1 MHz (current)
8 V, 1 MHz (current)
D
Optical power (μW)
Voltage (V)
Frequency (MHz)
Capacitor, 2.5 nF
D4, NON-RESONANT
D4, RESONANT (~3MHz)

Fig. S57. Electrical power characterization and comparison of optical power between non-resonant and resonant coils for wireless contact lenses.

(**A**) Scheme of the setup used for the characterization of the mutual inductance between transmitting and receiving coils. (**B**) Setup used to measure the voltage and current waveforms in the transmitting coil, to extract the input power. (**C**) Voltage and current waveforms in the transmitting coil for two designs of the receiving coils (D2 and D4), for 1 MHz applied frequency and different applied voltages. (**D**) Optical power emitted by the µLED integrated into the receiver coil, as a function of the applied voltage and the AC frequency of the input signal in the wireless powering setup through the transmitting coil. The comparison is made between a non-resonant design (D4) and the same design modified to include a coupling capacitor, at resonance frequency of ~ 3MHz. It can be observed that with the resonant coil, the operational voltage range becomes narrower, especially at the resonance frequency of 3 MHz (green arrow). Additionally, the optical power exhibits an increasing trend as a function of frequency, with the maximum achieved at the resonance (red arrow).

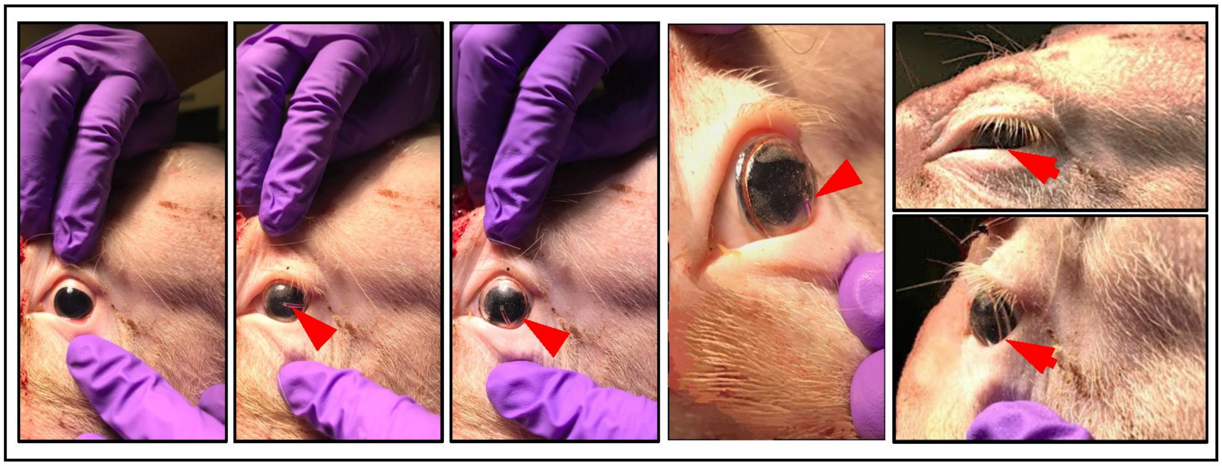

Fig. S58. In vivo application of fabricated contact lenses.

The contact lenses fabricated through the d-3DPLM process were applied on the eye of pigs. The images show the conformal implementation.

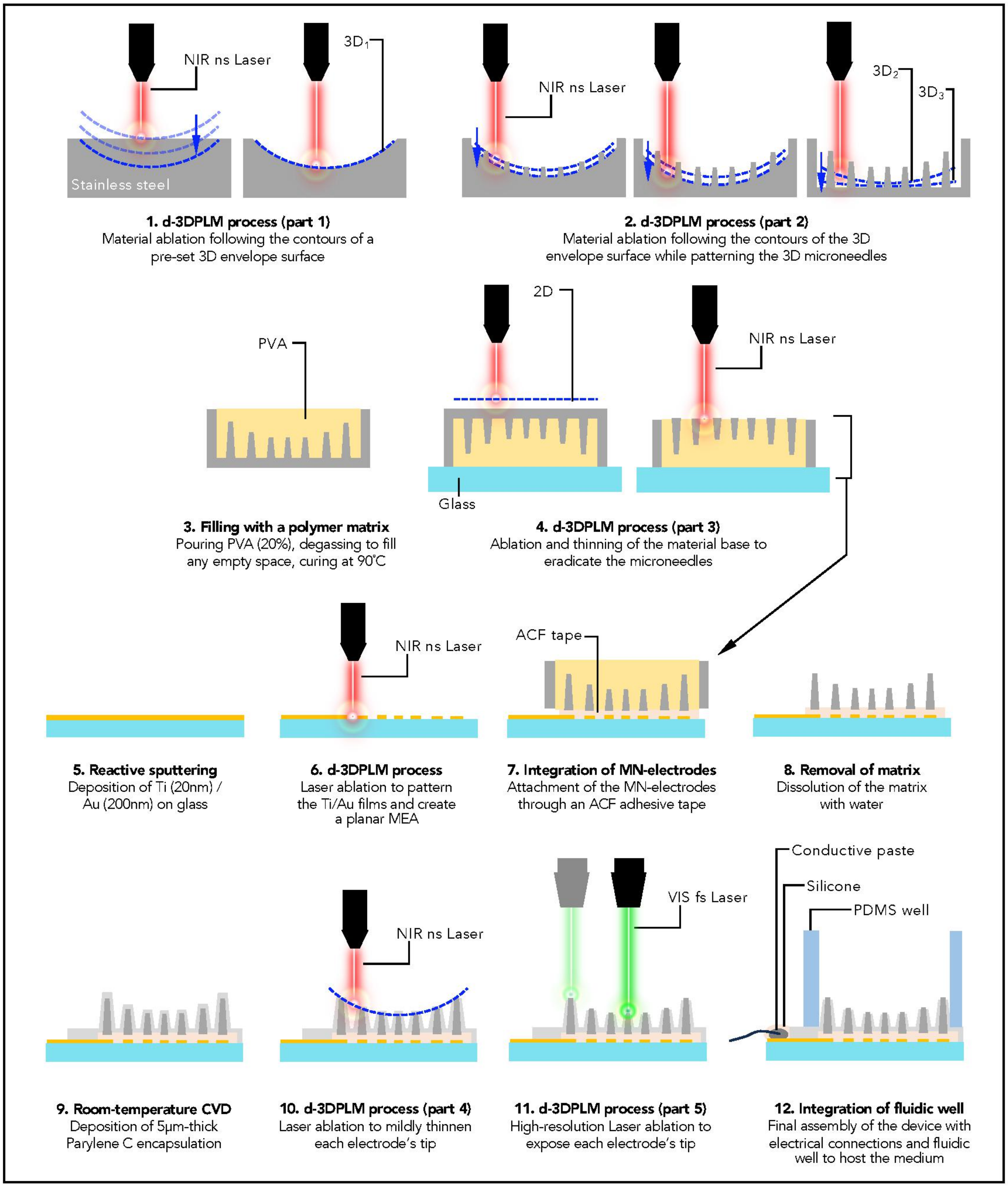

Fig. S59. Microfabrication process for the tunable-height 3D MEAs.

Schematic representation of the main steps of the process flow followed to fabricate the tunable-height 3D MEAs for in vitro diagnostics of cerebral organoids.

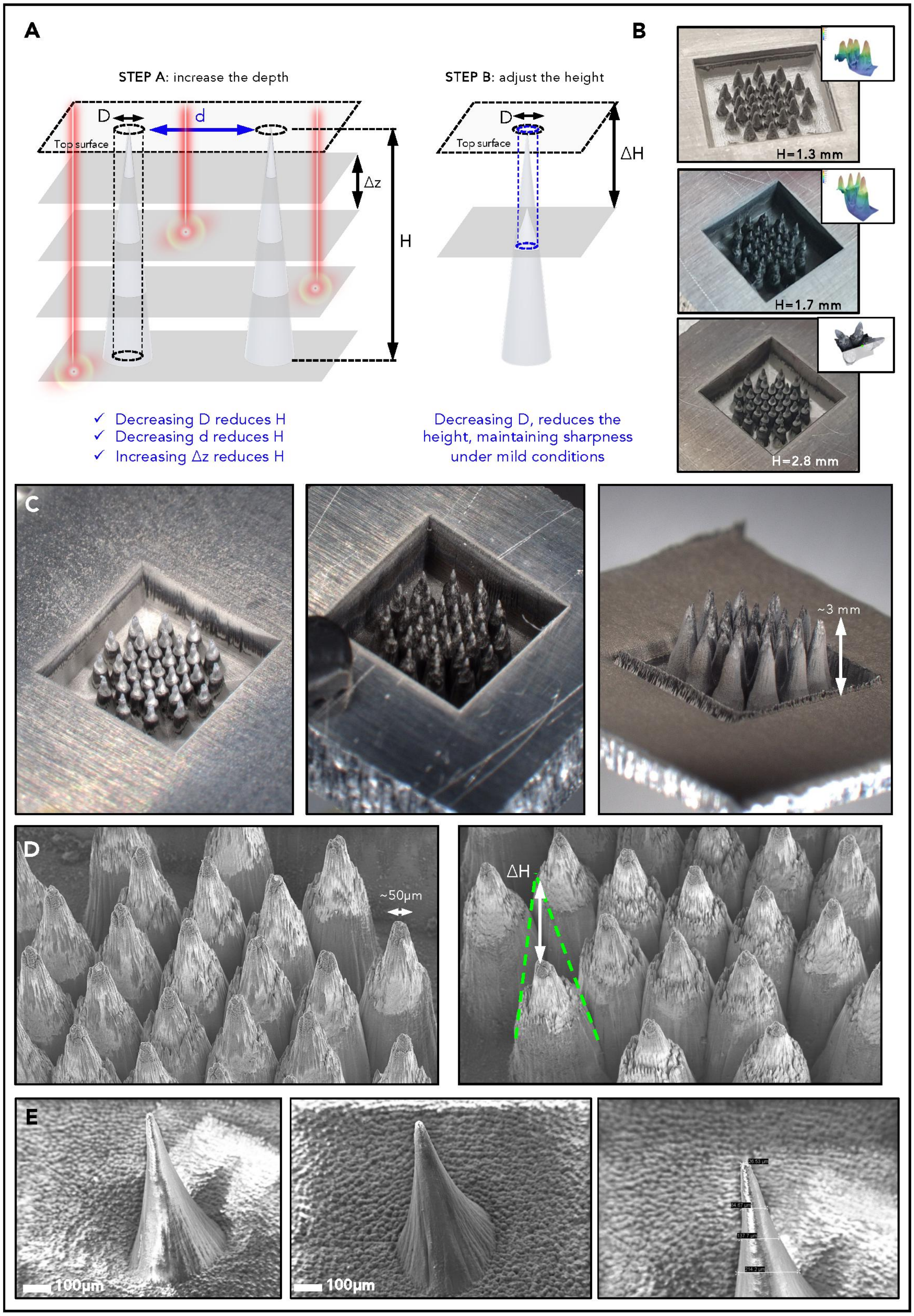

Fig. S60. Examples of MN arrays for 3D MEAs.

(**A**) Illustration of two main steps for the creation of MNs, which consist of increasing the depth (step A) or adjusting the height (step B). (**B, C**) Photos of some MN arrays obtained after laser ablation. (**D**) SEM micrographs of an array, where the effect of step B is visible.

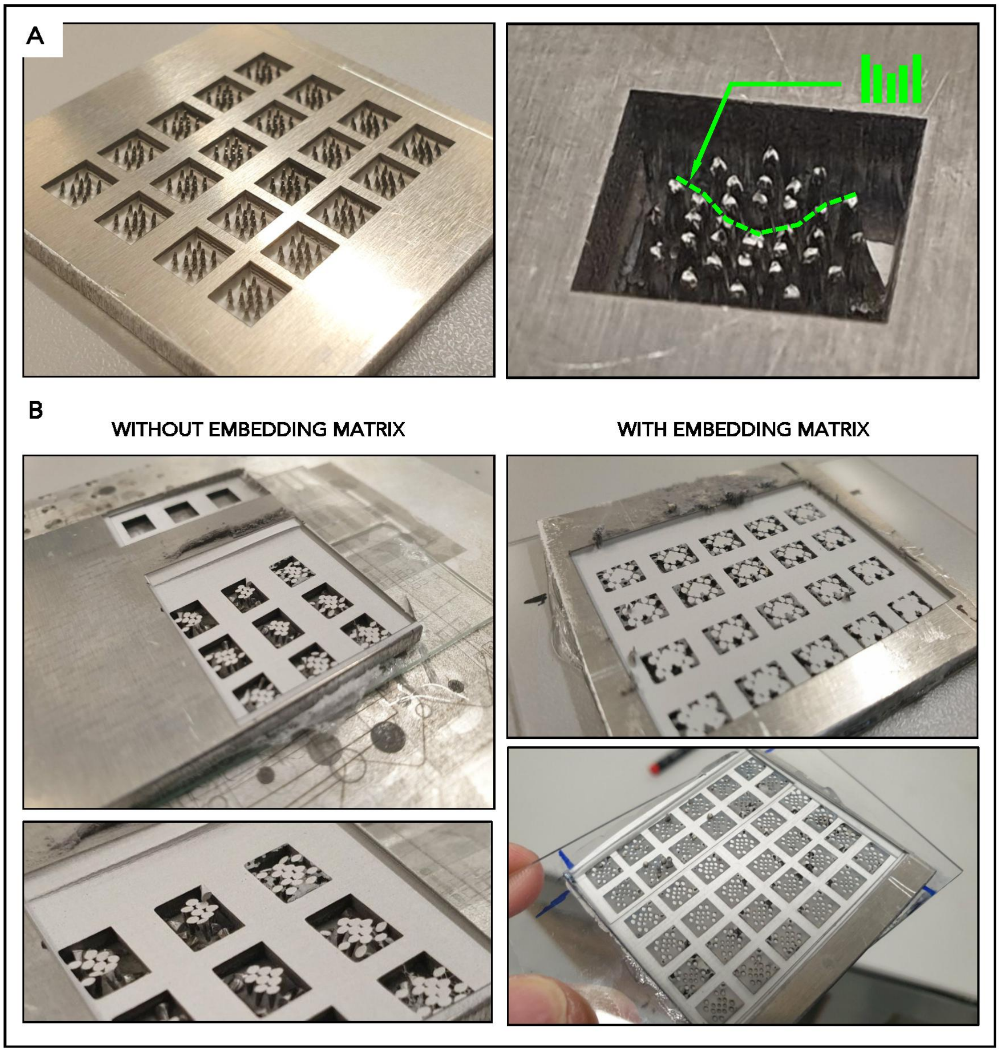

Fig. S61. Preparation of the MN-electrodes.

The images show the MNs obtained after the d-3DPLM process (**A**), and after the eradication (**B**). The presence of the embedding matrix allows to keep the MNs in place during the ablation.

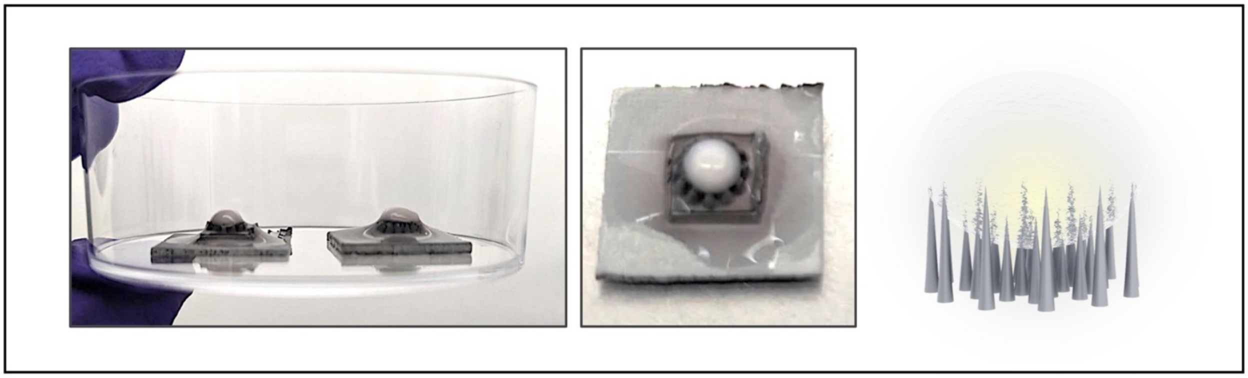

Fig. S62. Human cerebral organoids placed on top of the MN array.

The images show two stainless steel block after the d-3DPLM process, with organoids on top of them.

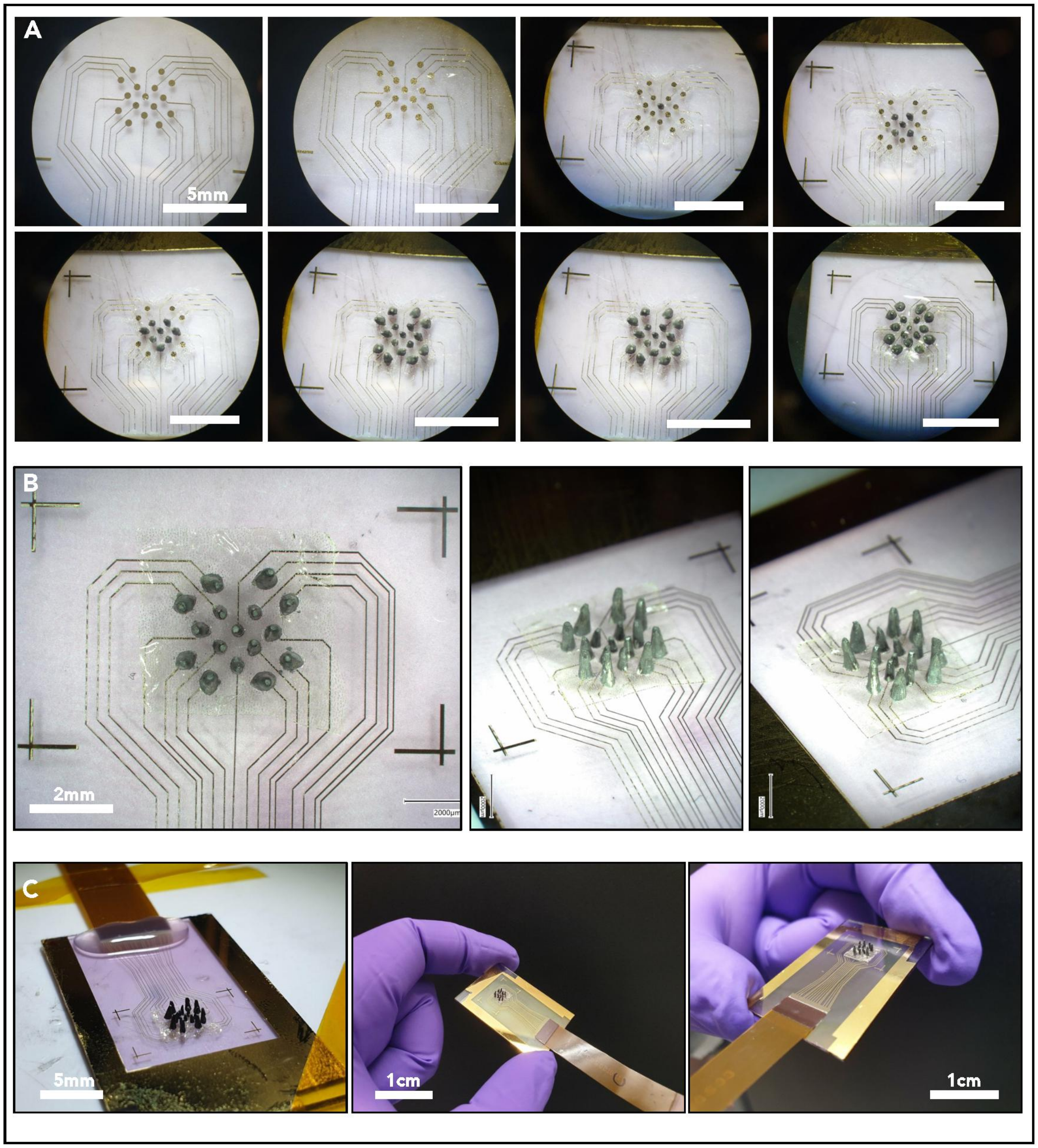

Fig. S63. Integration of MN electrodes into the MEA device.

(**A**) Optical micrographs of the MN electrodes integrated onto the ACF tape on the planar MEA. (**B**) Photos of the 16 MN electrodes integrated into the MEA device. (**C**) Photos of the 3D MEA device connected with fat flexible cable.

Slight variations in MN height and tilt, as observed in (**B**), arise from tolerances inherent to the laser ablation process used to release the structures from the stainless-steel block. In particular, localized material recast at the MN base can introduce a slight angular deviation, resulting in tilted electrodes. While this tilt does not compromise the tunability or accuracy of the tip heights, since the apex remains well defined, it highlights the importance of further refining both the fabrication parameters and post-processing protocols. We note that such optimization can become even more critical when adapting the approach to different materials or substrate systems, where the ablation dynamics may vary significantly.

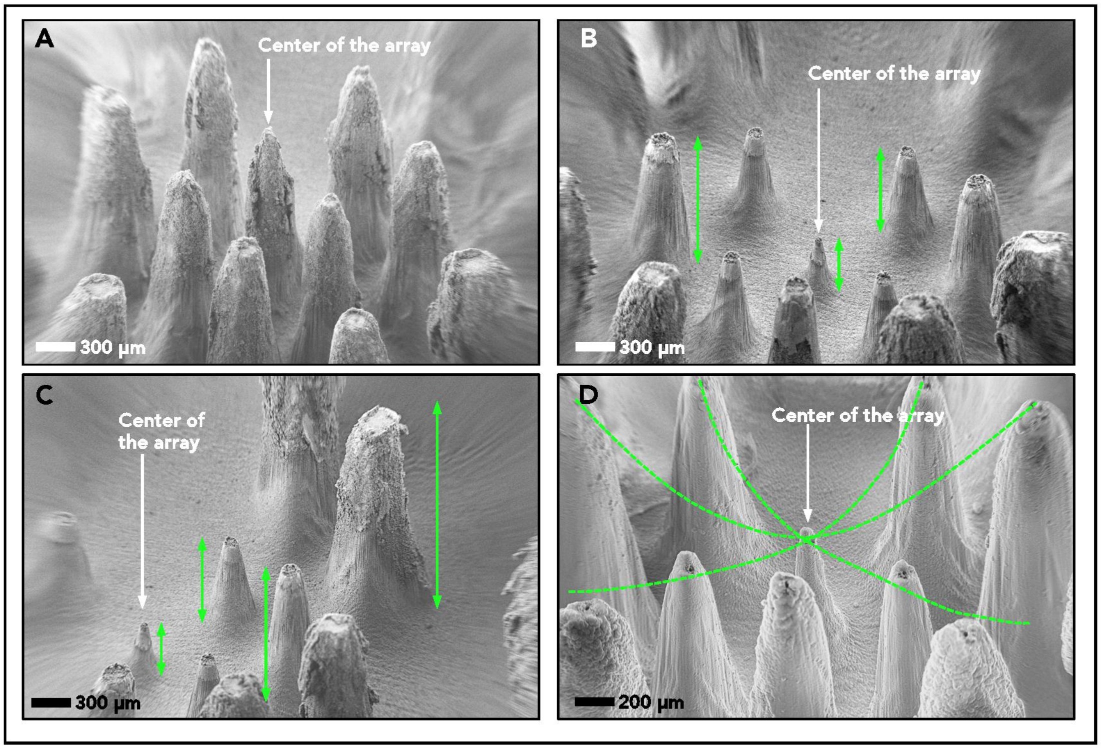

Fig. S64. SEM micrographs of MN electrodes after laser ablation.

SEM micrographs of the MN electrodes after a standard sequential laser ablation (obtaining MNs with the same height) (**A**), and after the d-3DPLM process (obtaining MNs with different heights) (**B, C**). (**D**) SEM micrographs of the tunable-height MN electrodes coated with the Parylene C encapsulation.

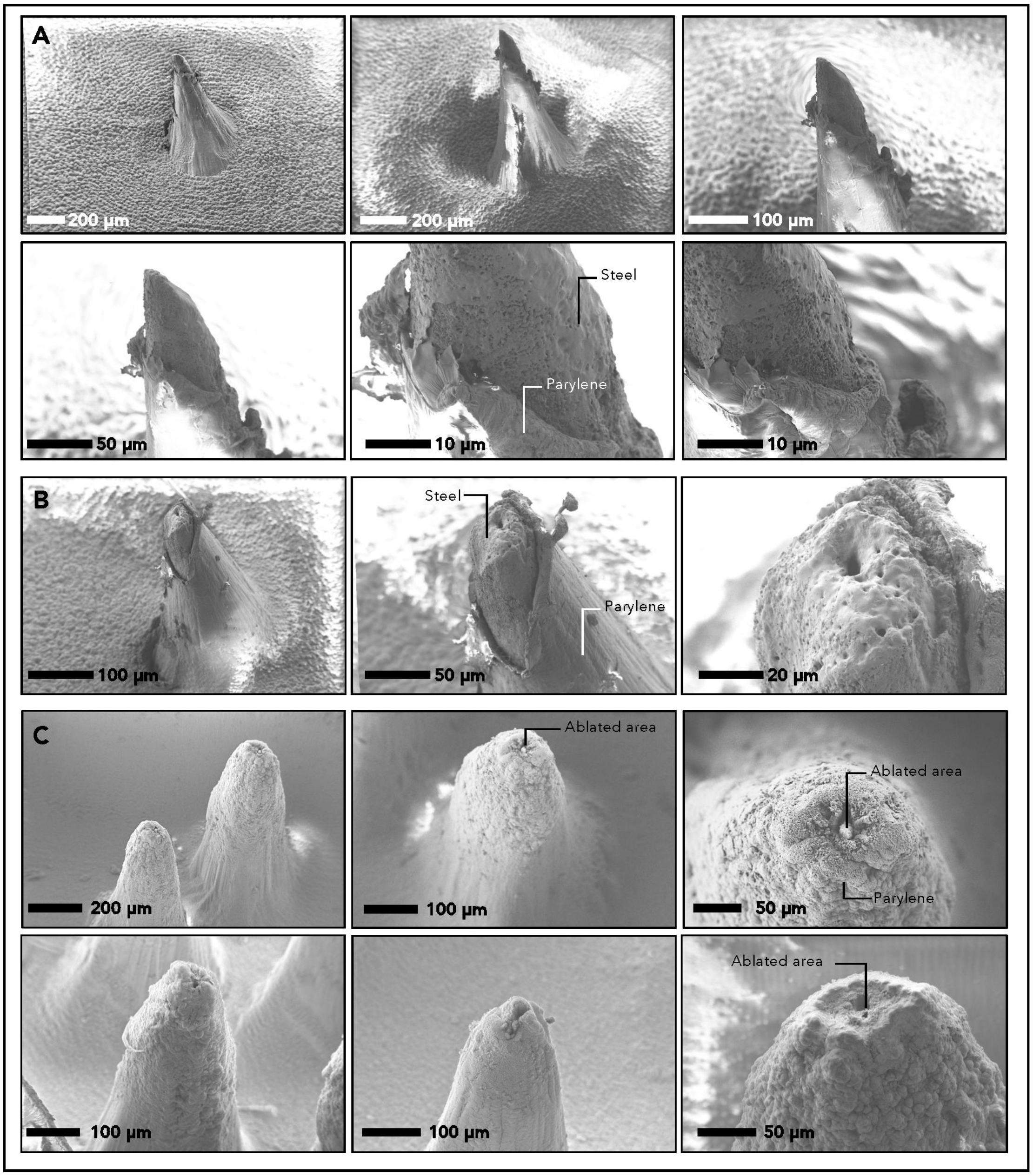

Fig. S65. SEM micrographs of the MN electrodes encapsulated with Parylene C and with the tips exposed after laser ablation.

(**A, B**) SEM micrographs of the tips of the MN electrodes exposed after the ablation performed with the last two steps of the fabrication process (d-3DPLM with NIR ns-pulsed laser, and focused ablation with green fs-pulsed laser). (**C**) SEM micrographs of the tips of the MN electrodes exposed after ablation only with green fs-pulsed laser. Although the steel is exposed, the resulting shape of the exposed area is irregular.

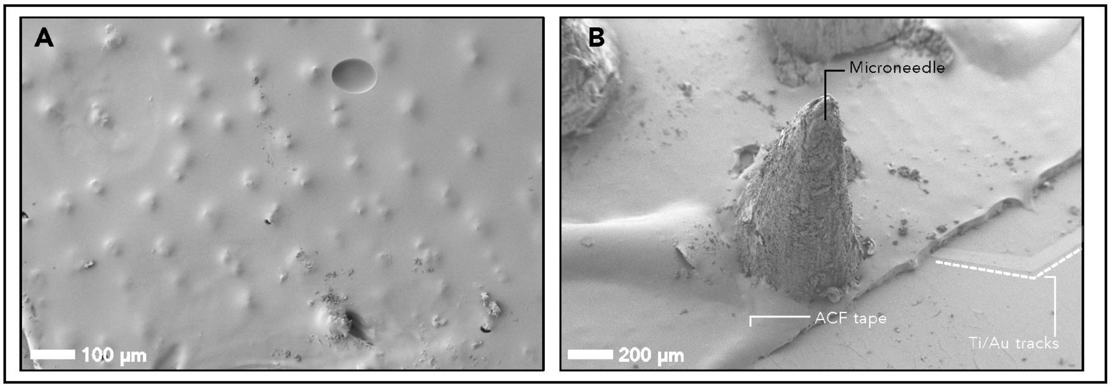

Fig. S66. Integration of the MN electrodes through the ACF tape.

(**A**) ACF tape used to integrate the MN electrode on the flat MEA. The conductive fillers can be observed. (**B**) Example of one MN electrode adhering onto the ACF tape.

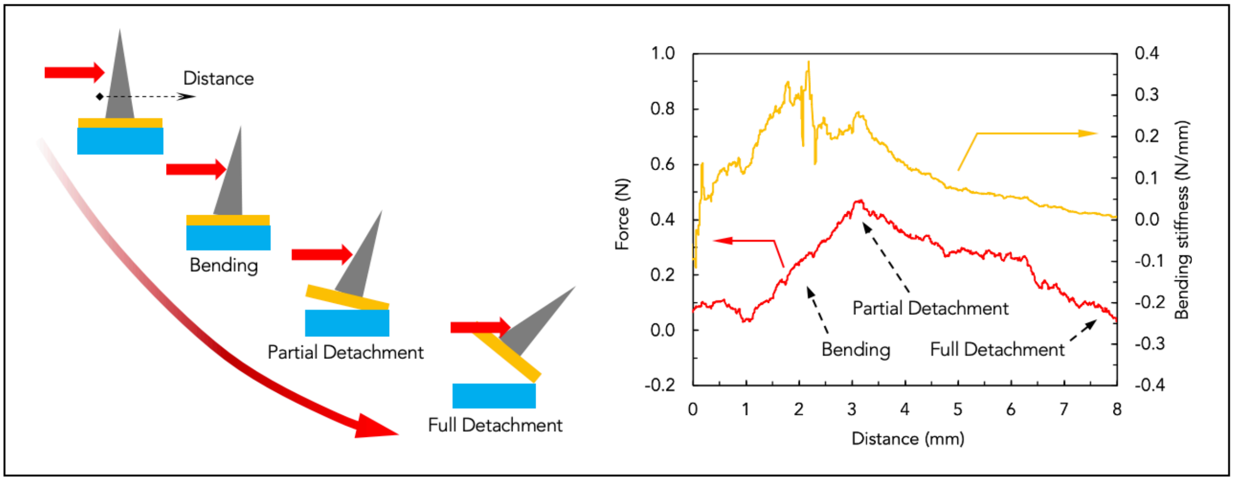

Fig. S67. Mechanical testing of the adhesion between MN and ACF tape.

The scheme shows the configuration of the mechanical test. The plot shows the force vs distance curve (red) and the bending stiffness (or adhesion rigidity, yellow) for the MN/tape material system.

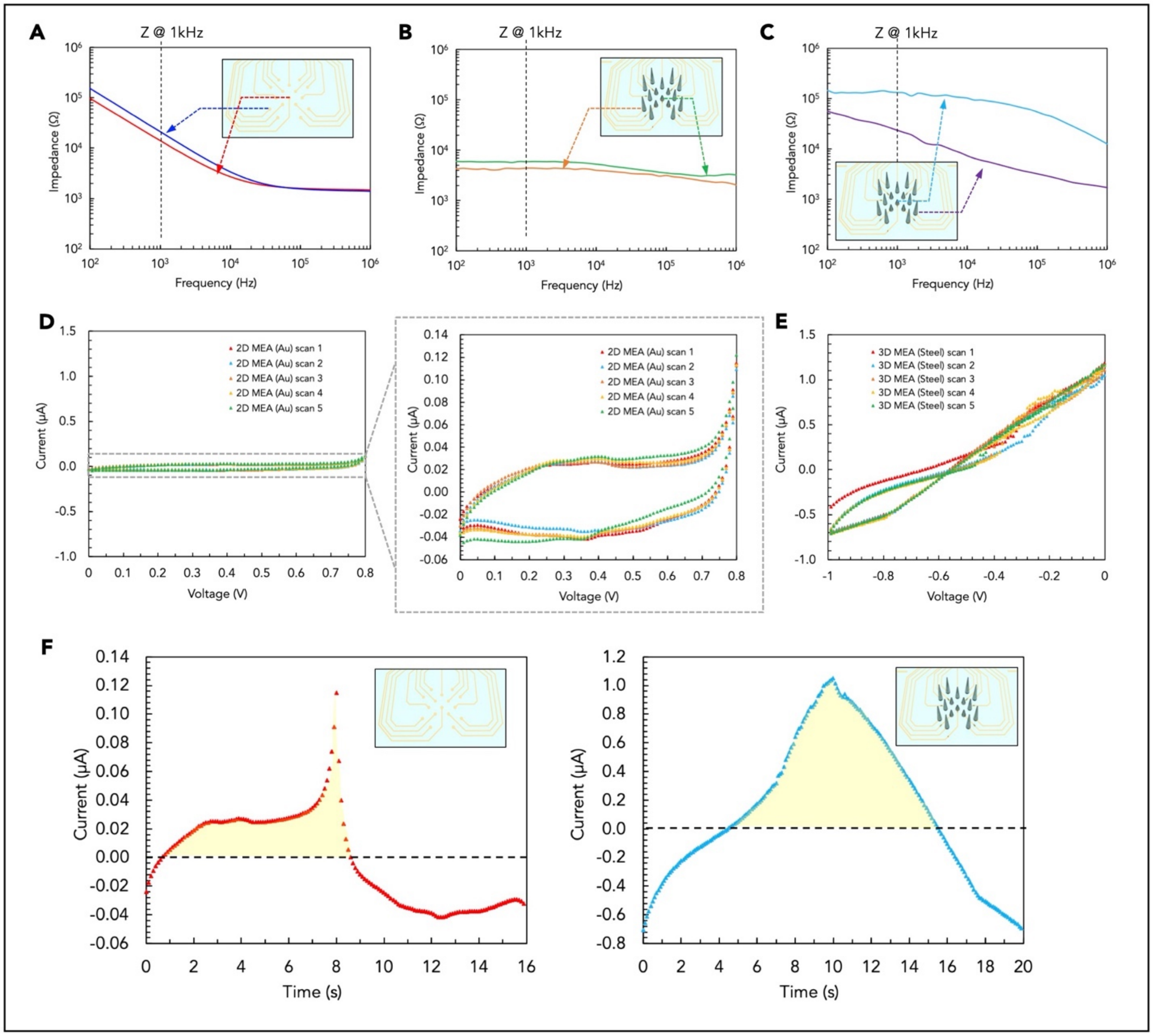

Fig. S68. EIS and CV measurements of 2D and 3D MEAs.

EIS measurements for an electrode in the middle and the outer layer of the array, for (**A**) the flat Au MEAs, (**B**) the 3D MEAs with non-encapsulated MN-electrodes, (**C**) the 3D MEAs with encapsulated MN-electrodes. (**D, E**) CV curves for the 2D MEAs (**D**) and 3D MEAs (**E**). (**F**) Current vs time curves for the CV scans on the electrodes of the 2D and 3D MEAs. The yellow area indicates the charge storage process during the voltage sweep.

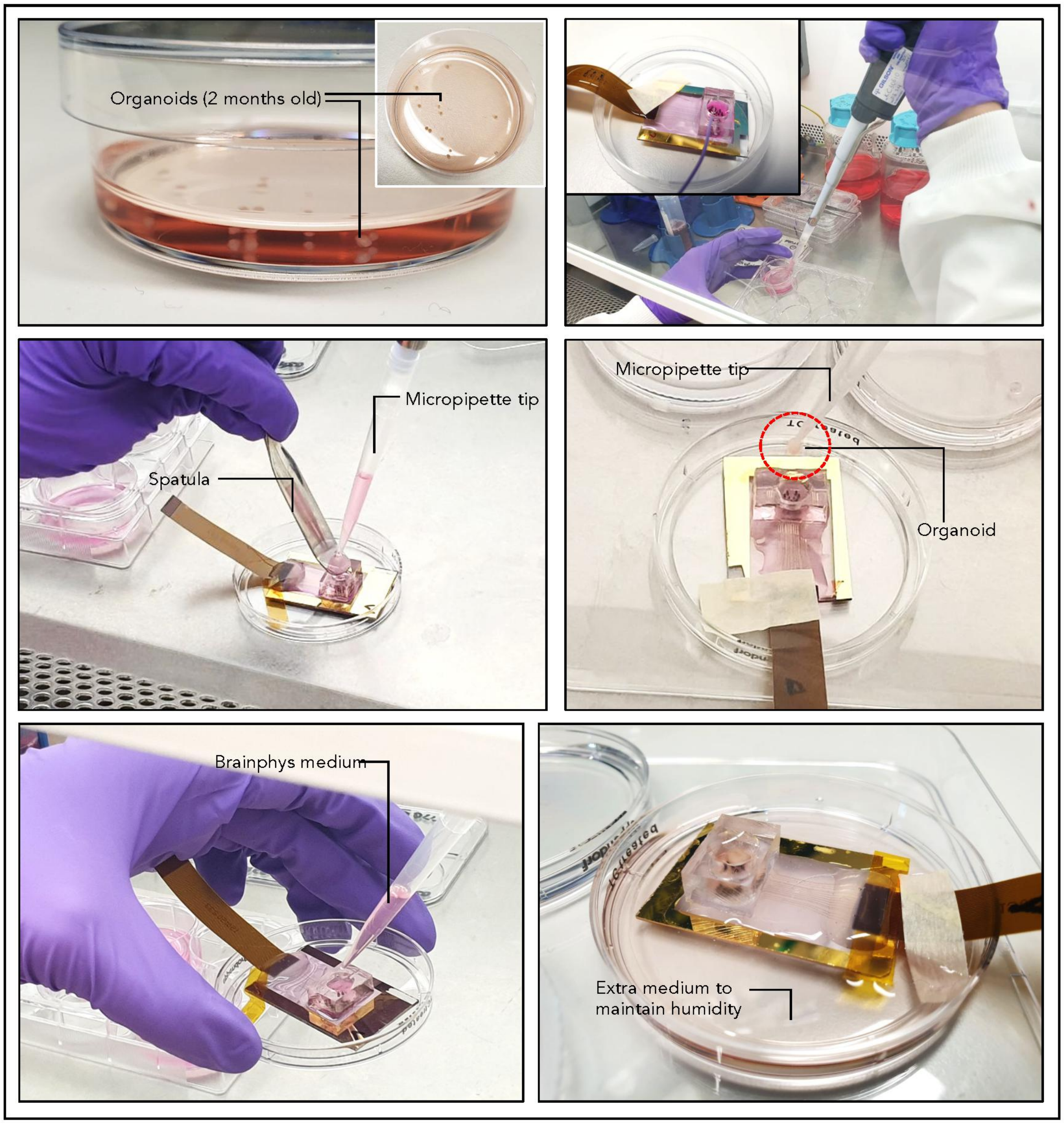

Fig. S69. Photos of the integration of the cerebral organoids onto the 3D MEAs.

Photos of the organoids grown in the liquid culture medium and then integrated onto the 3D MEAs using the micropipette and a spatula. The organoid is placed inside the fluidic well of the device and additional medium is injected with the micropipette in the fluidic well and in the space surrounding the device.

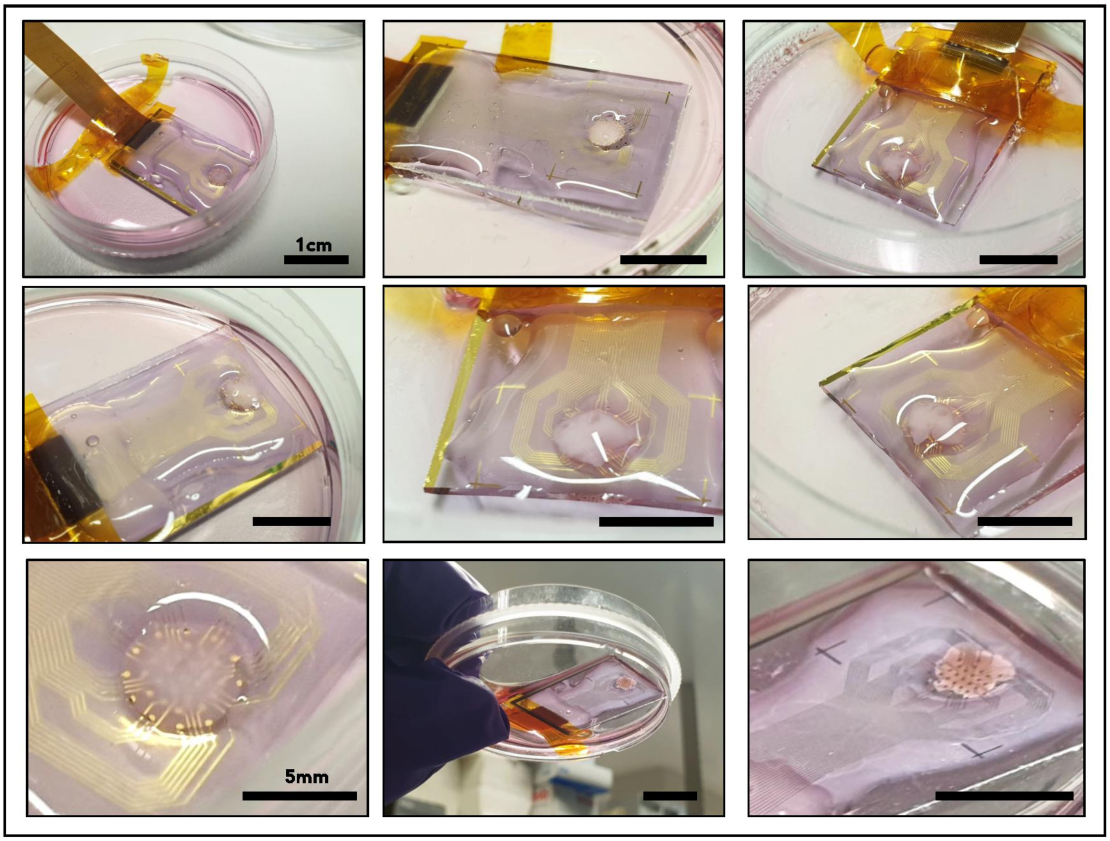

Fig. S70. Cerebral organoid on the flat MEA device.

The photos show the whole cerebral organoid placed on the flat MEA device. The organoid is evidently squeezed on the device. Unlabeled scale bars: 1cm.

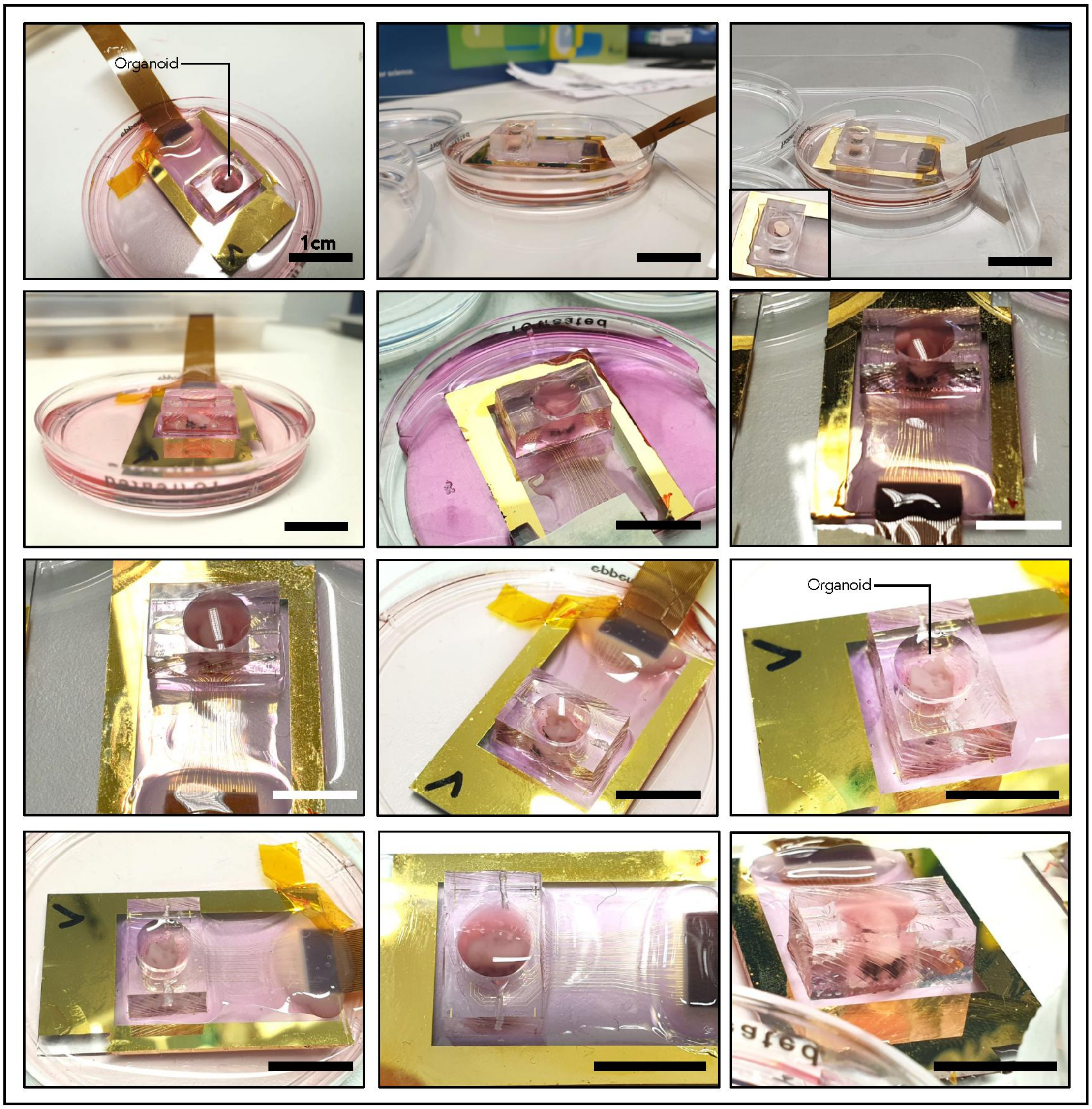

Fig. S71. Cerebral organoid on a 3D MEA.

The photos show the whole cerebral organoid placed within the fluidic well of the 3D MEA device. Unlabeled scale bars: 1cm.

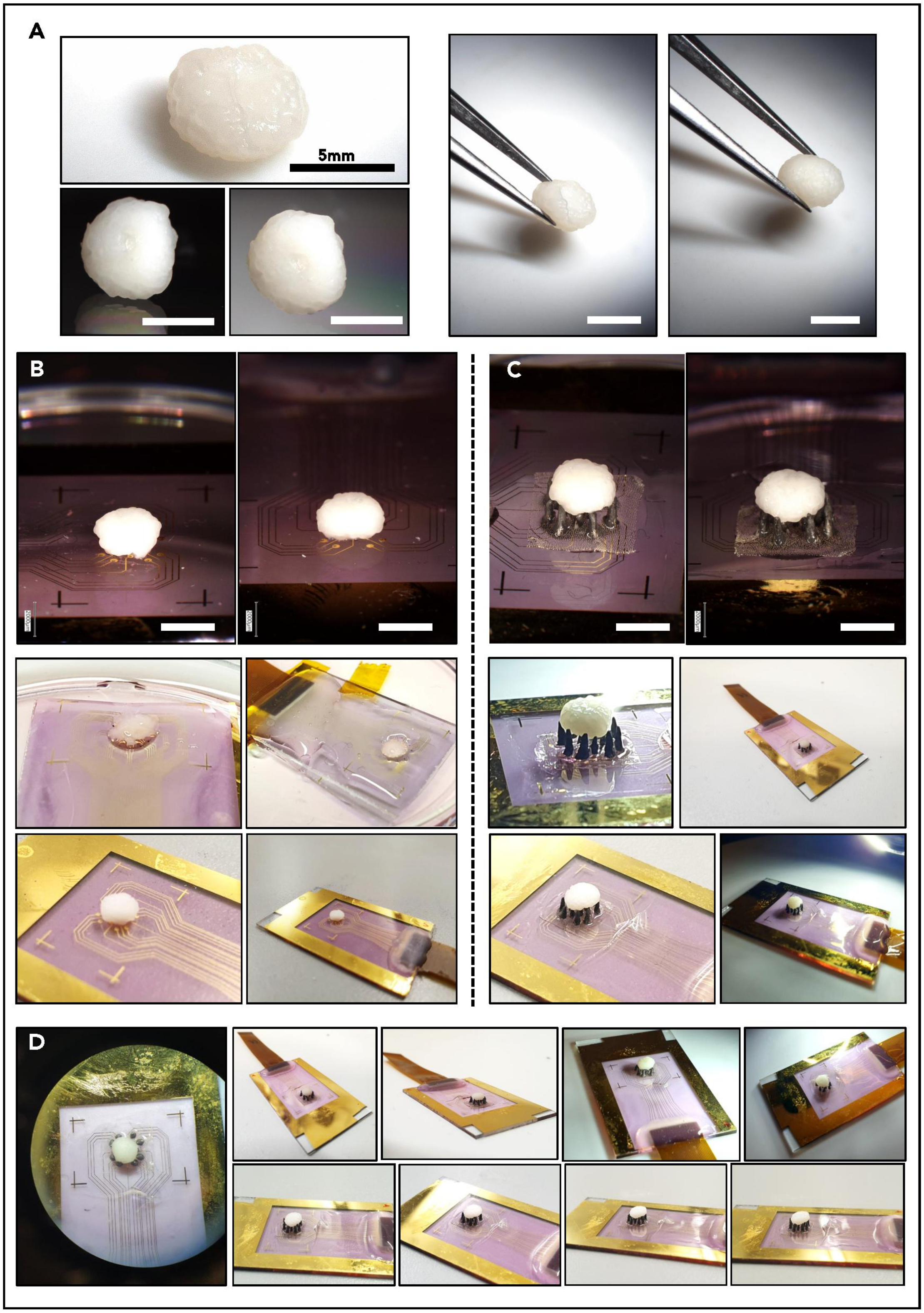

Fig. S72. Fixed cerebral organoid after the growth.

(**A**) Photos of a whole cerebral organoid fixed after the growth protocol. Unlabeled scale bars: 5mm. (**B, C**) Comparison between the 2D and 3D MEAs in terms of placement of the cerebral organoid (fixed). The contact is more irregular and less intimate with the 2D MEA. Unlabeled scale bars: 5mm. (**D**) Photos showing the organoid on the 3D MEA from different perspectives.

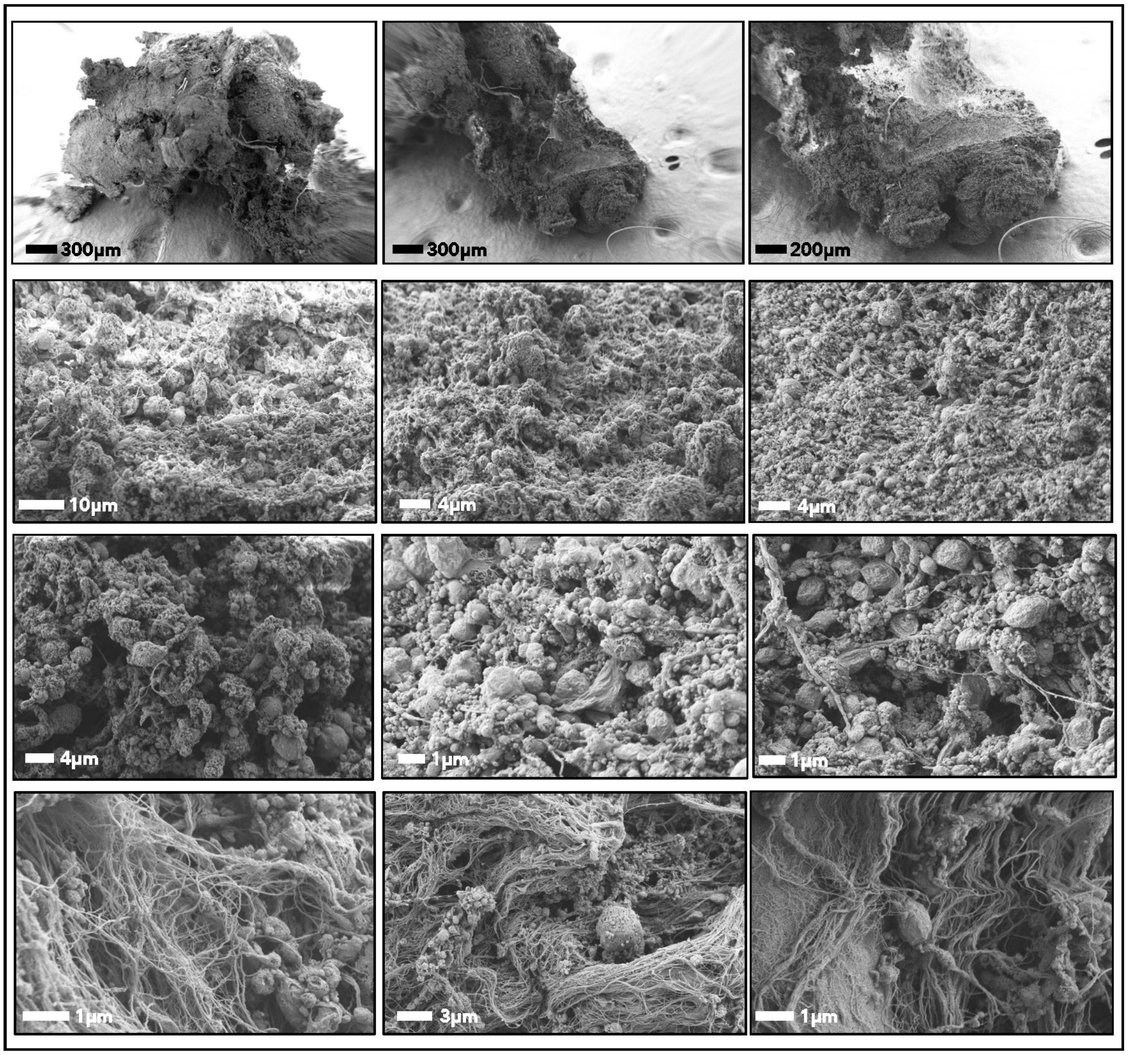

Fig. S73. SEM micrographs of the cerebral organoid after electrophysiological recordings on 2D MEAs.

SEM micrograph showing the structure of the cerebral organoid after being placed on the 2D MEA. The magnified micrographs show the degraded microstructure with an irregular network of filaments and neuronal cells.

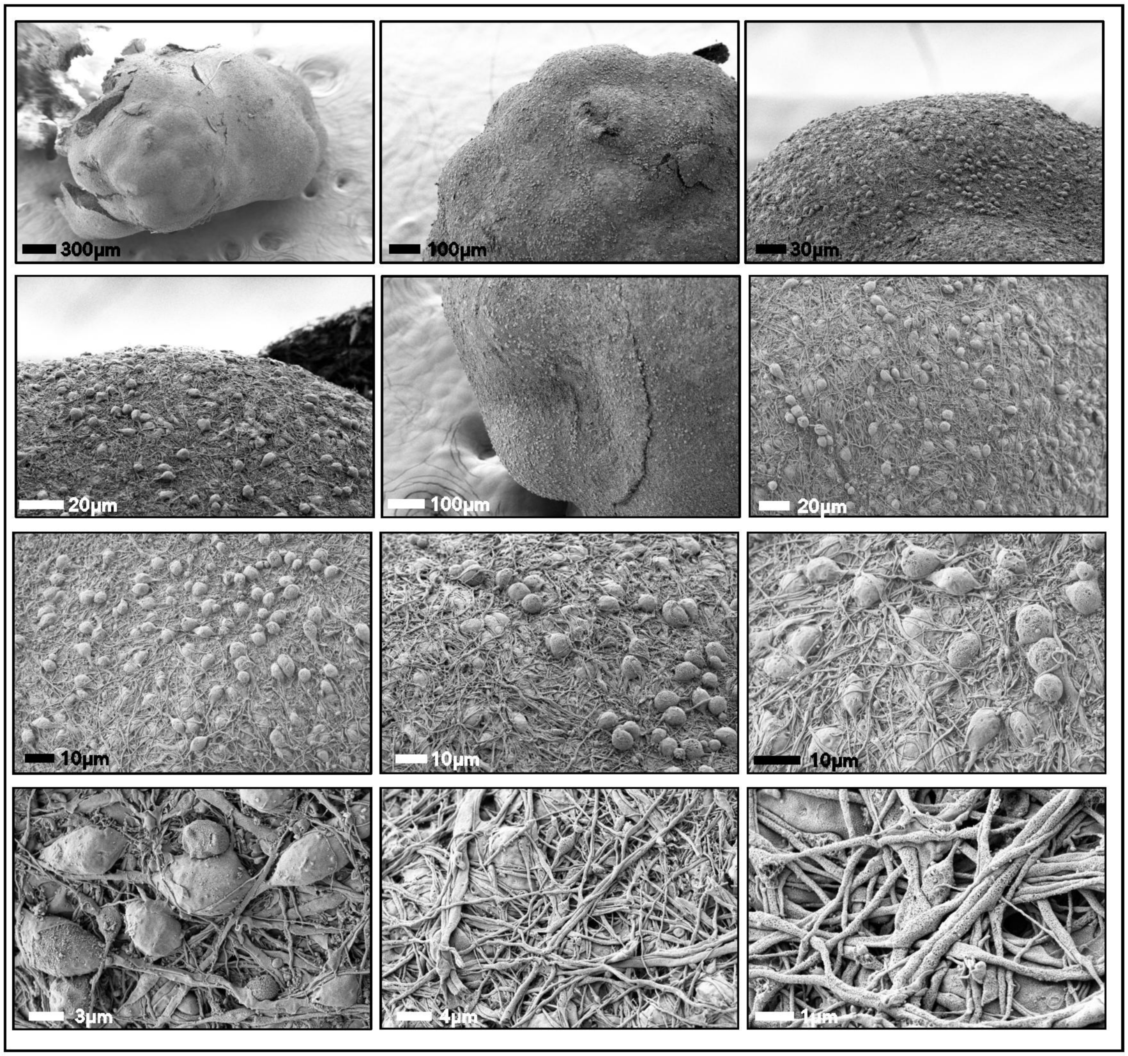

Fig. S74. SEM micrographs of the cerebral organoid after electrophysiological recordings on 3D MEAs.

SEM micrograph showing the structure of the cerebral organoid after being placed on the 3D MEA. The magnified micrographs show the intact microstructure with the regular network of filaments and neuronal cells, in contrast with that of the organoid on the 2D MEA (see previous figure).

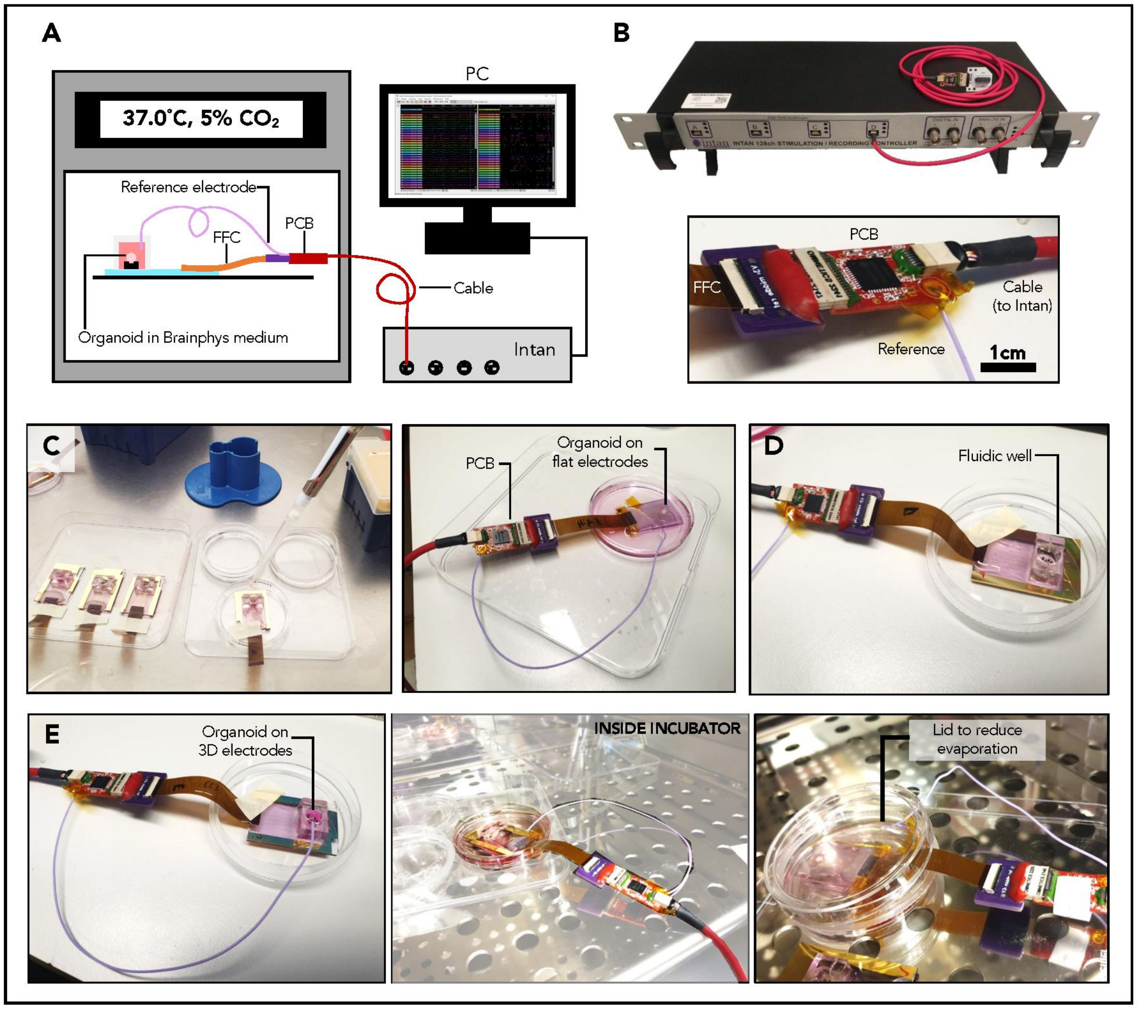

Fig. S75. Setup used for electrophysiological recording of neural activity of cerebral organoids.

(**A**) Setup including: incubator, Intan electrophysiology platform, PC and the device connected through a customized PCB. (**B**) Intan platform with the associated connector and customized PCB. The reference electrode is connected through a wire to the culture medium. (**C-E**) MEA devices connected to the Intan cable: flat electrodes (**C**), 3D electrodes (**D**). (**E**) Device placed on the shelf inside the incubator.

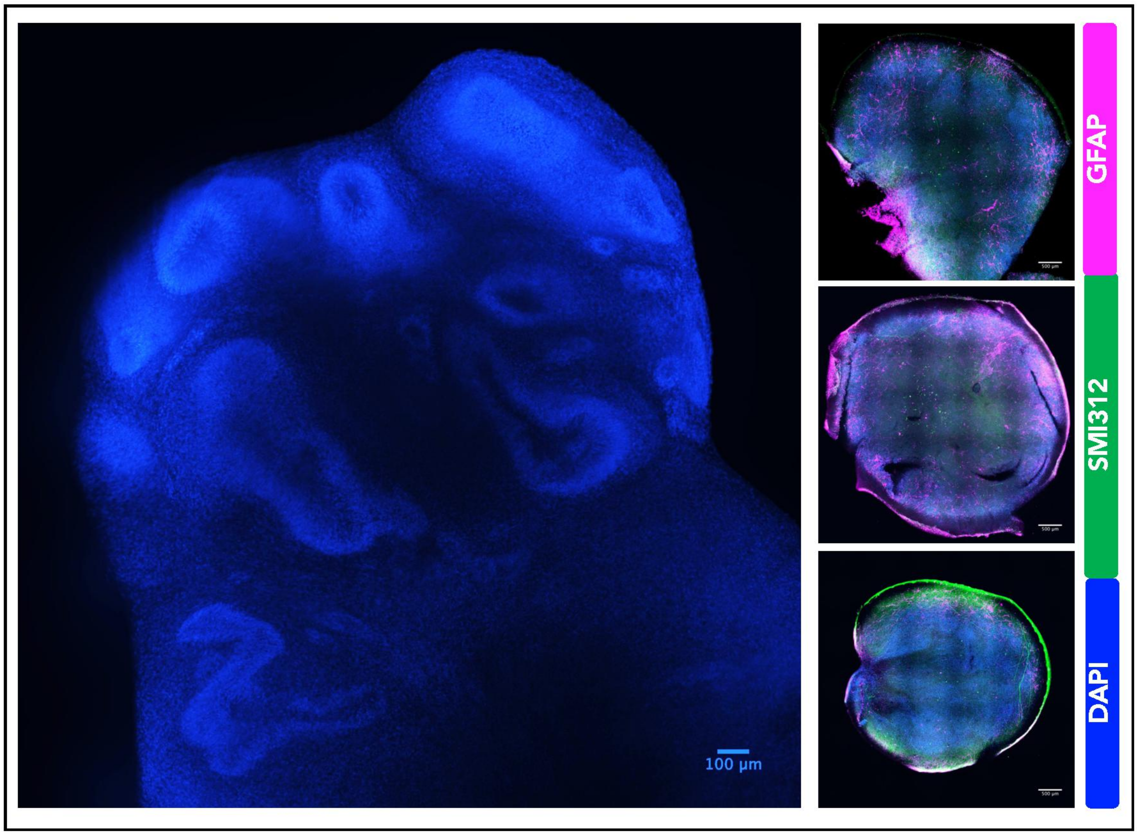

Fig. S76. Immunohistochemistry for different markers on whole cerebral organoids and some representative sections.

Immunohistochemistry for the marker DAPI (blue) to stain nuclei, SMI312 (green) to stain axons, GFAP (purple) to stain astrocytes, on a whole cerebral organoid and some representative sections and ALICOs.

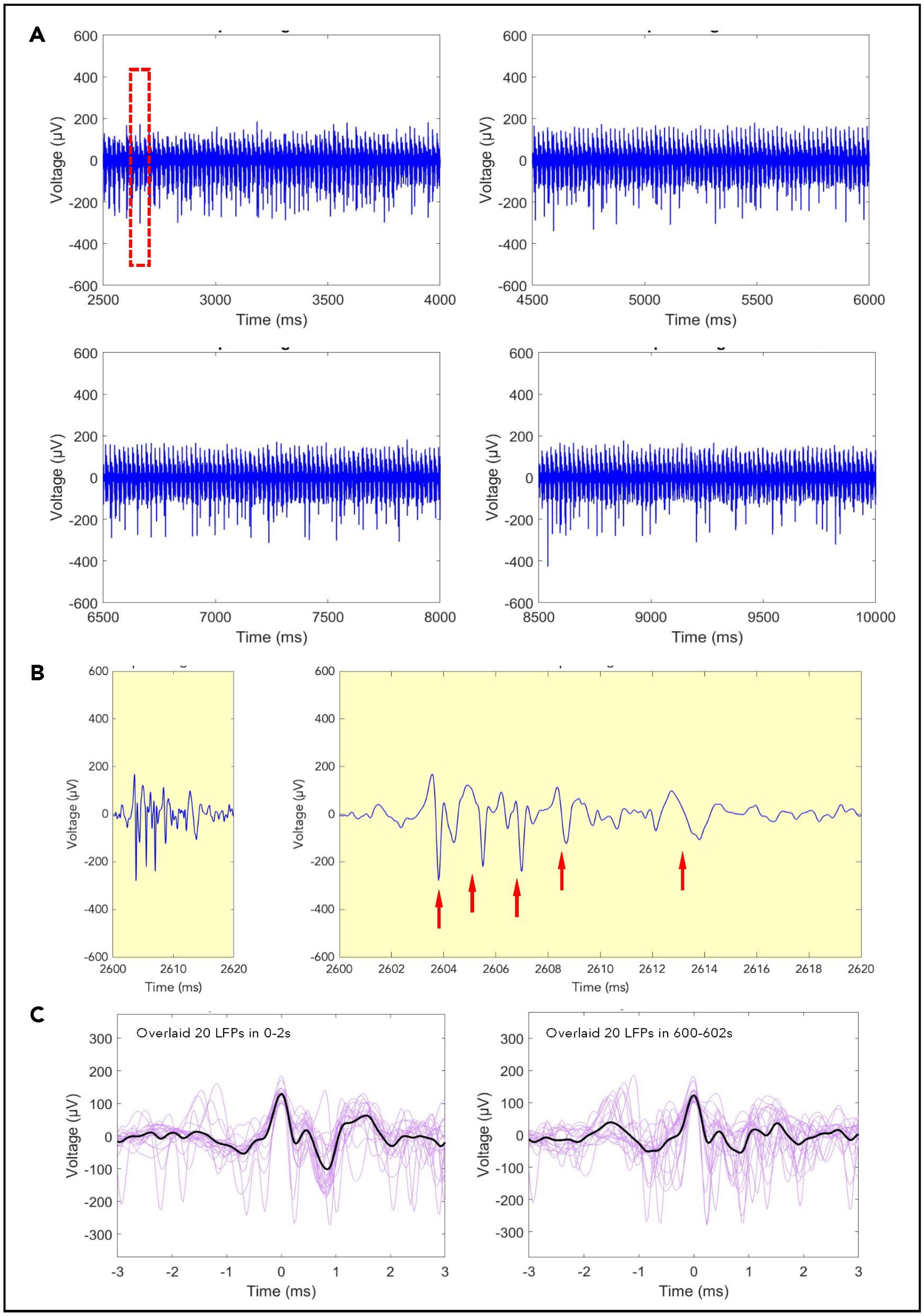

Fig. S77. Electrophysiological recordings of the spontaneous activity of cerebral organoids on the 3D MEAs.

(**A**) Spontaneous activity recorded from a single 3D MN electrode in a MEA device in the first 10 s of measurements. The local field potentials (LFP) can be observed. (**B**) Expanded 20ms-long signal from the recordings in (**A**). LFPs are marked with arrows. (**C**) Representative neural event detected during the electrophysiological recordings. Overlay of 20 LFPs (purple) detected in 2s in the beginning of the measurement (0-2s, left) and towards the end (600-602s, right), with the mean waveforms in black.

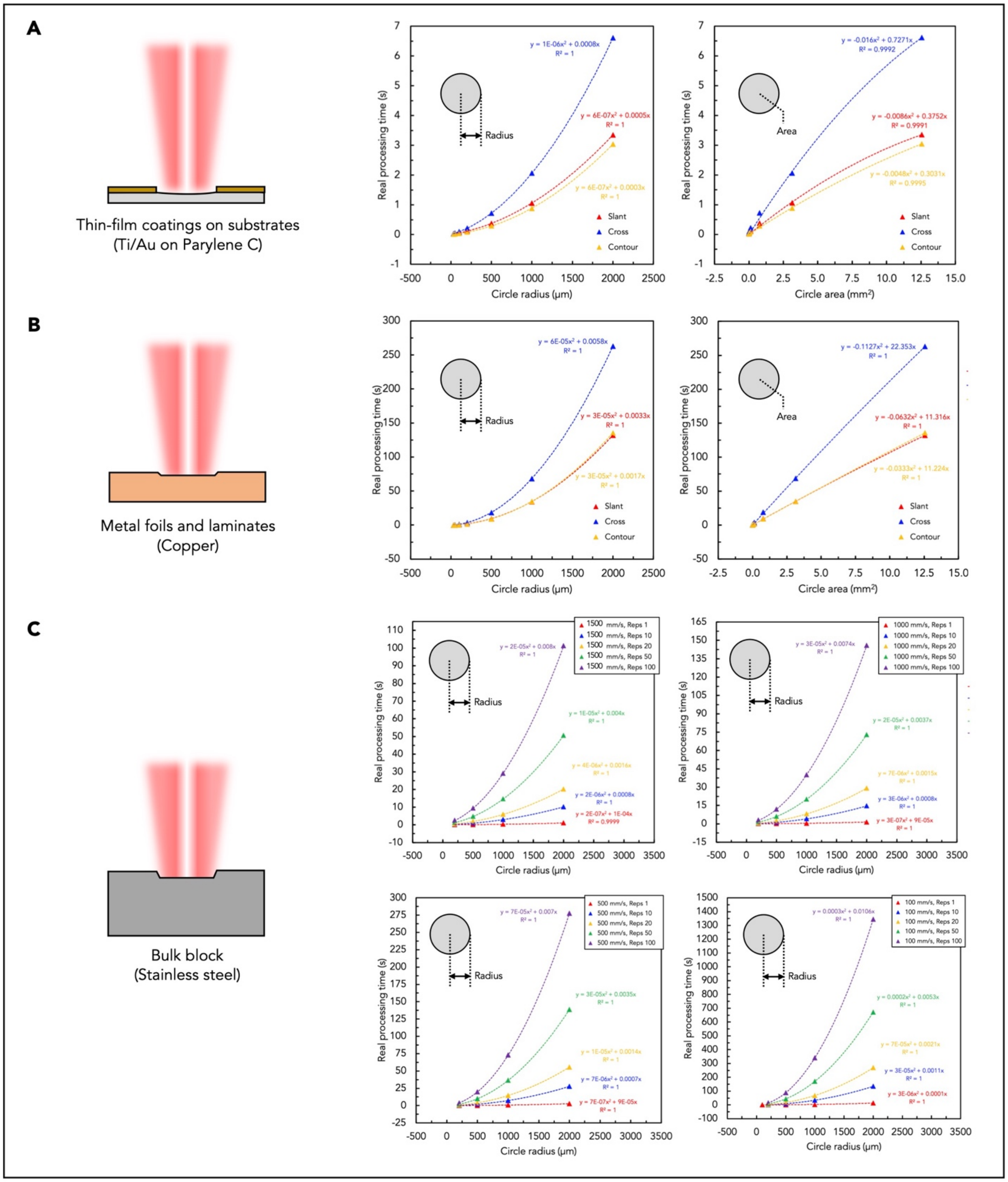

Fig. S78. Real (after actual ablation) processing times for ablation of circles with different radii (30µm to 2mm), on different material systems.

(**A**) Processing times for ablation of Ti/Au thin films on Parylene C, for slant, cross and contour hatching, vs circle radius and circle area, at the optimal laser parameter settings used for this material system. (**B**) Processing times for ablation of Cu foils, for slant, cross and contour hatching, vs circle radius and circle area. (**C**) Processing times for ablation of stainless-steel blocks, for contour hatching, vs circle radius, at 30% power and for different scan speeds (100-1500 mm/s) and repetitions (1 to 100). The fitting lines for all the plots are based on polynomial fitting (order 2).

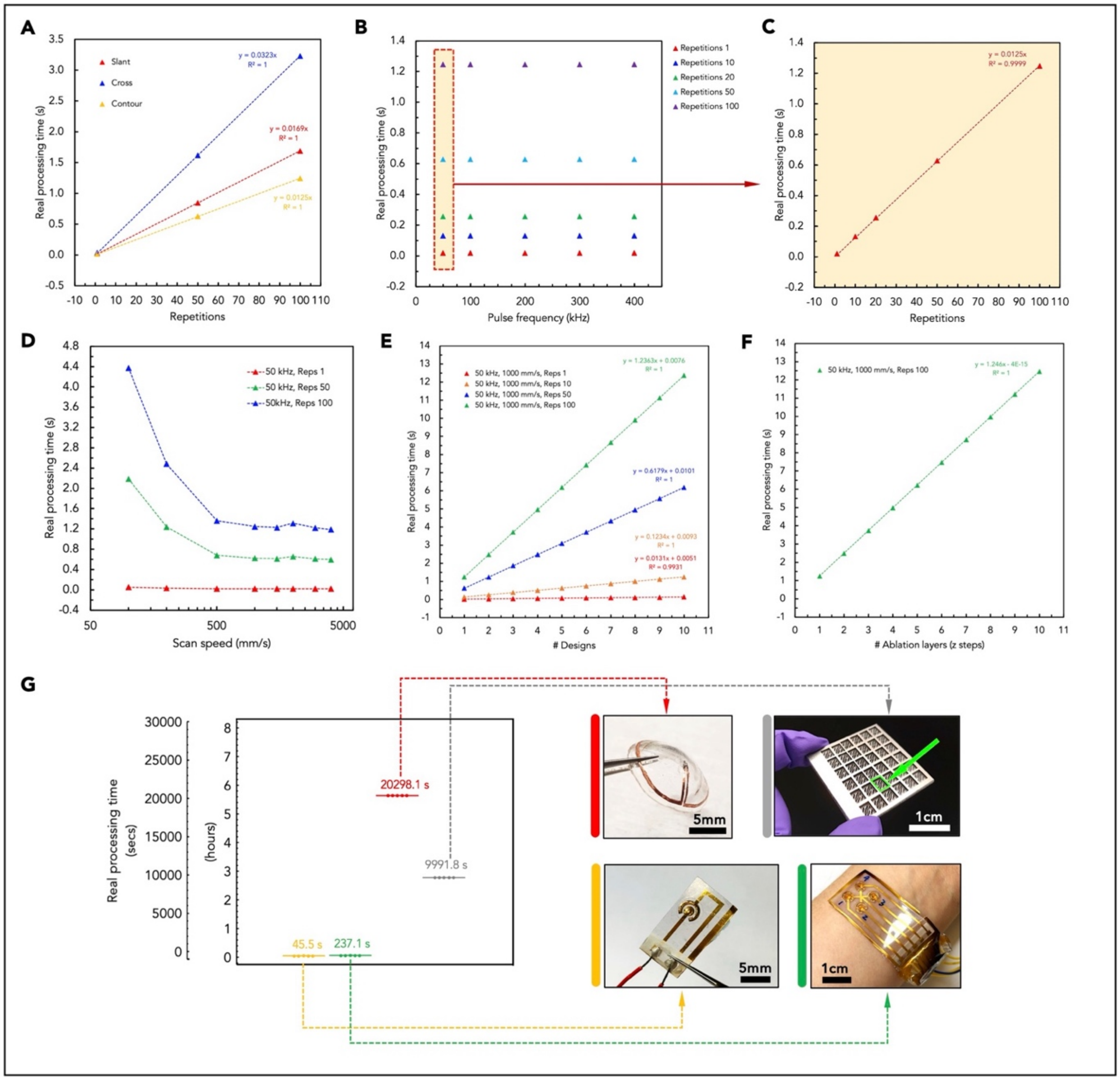

Fig. S79. Real (after actual ablation) processing times for ablation of circles with different radii (30µm to 2mm), on stainless steel, and processing times for the devices proposed in the study.

(**A**) Processing times for ablation of a 100µm-radius circle on stainless steel, for slant, cross and contour hatching, vs number of repetitions, at the optimal laser parameter settings used for this material system (power 30%, scan speed 1000 mm/s, pulse frequency 50 kHz, repetitions 1). (**B**) Processing times for ablation of a 100µm-radius circle on stainless steel, with contour hatching, vs pulse frequency and for different repetitions. (**C**) Processing times for ablation of a 100µm-radius circle on stainless steel, for contour hatching, vs number of repetitions. (**D**) Processing times for ablation of a 100µm-radius circle on stainless steel at power 30%, pulse frequency 50kHz, vs scan speed and for different repetitions. (**E**) Processing times for ablation of a 100µm-radius circle on stainless steel at power 30%, scan speed 1000 mm/s, pulse frequency 50 kHz, repetitions 1-100. The same design is ablated multiple times thus the curves are plotted vs the number of designs. (**F**) Processing times for sequential ablation of a 100µm-radius circle on stainless steel at 30%, 1000mm/s, 50 kHz, 100 repetitions, vs the number of ablation layers for different z steps. (**G**) Processing times for ablation of the electrode designs used in this work, i.e., electro-haptic electrodes for finger and wrist stimulation; coils for wireless contact lenses (design D1); MN electrodes for MEAs (single array).

Table S7. Comparison among the most common and widely used fabrication techniques for 3D bioelectronics.

Technique	UV photolithography	E-beam lithography	Soft lithography	Additive manufacturing	CNC micromachining	Standard Laser micromachining	d-3DPLM
**Type**	Wet and/or dry process	Wet and/or dry process	Dry process	Dry or wet process	Dry process	Dry process	Dry process
**Standard steps**	1. Substrate preparation<br>2. Photoresist coating<br>3. Exposure (UV)<br>4. Development<br>5. Wet/dry etching<br>6. Resist removal<br>7. Release or transfer printing	1. Substrate preparation<br>2. Electron beam exposure<br>3. Development<br>4. Wet/dry etching<br>5. Resist removal<br>6. Release or transfer printing	1. Master mold fabrication<br>2. PDMS casting<br>3. Curing<br>4. Mold release<br>5. Replica molding or microcontact printing<br>6. Release or transfer printing	1. CAD design<br>2. Layer-by-layer material deposition<br>3. Curing or sintering (if needed)	1. CAD design<br>2. Tool path programming<br>3. Material removal (milling, drilling, etc.)<br>4. Surface finishing	1. Laser setup<br>2. Material cutting or ablation<br>3. Surface finishing	1. 3D envelope surface programming<br>2. Laser setup<br>3. Material cutting or ablation<br>4. Surface finishing
**Spatial (in-plane) resolution**	~1 µm (~50 nm with advanced techniques, e.g., EUV)	<10 nm	~1 µm	~100 µm (it varies by method)	~10 µm	~1-10 µm (depends on the laser beam diameter)	~5-10 µm
**Process time**	Medium (hours)	Slow (hours to days)	Medium (hours)	Fast to slow (minutes to days)	Medium (hours)	Very fast to medium (seconds to hours)	Very fast to medium (seconds to hours)
**Advantages**	▪ Optimal to pattern thin film materials<br>▪ High throughput<br>▪ High scalability<br>▪ Well-established in industry	▪ Extremely high resolution<br>▪ Maskless process	▪ Cost-effective<br>▪ Flexible materials<br>▪ Easy fabrication<br>▪ Suitable for 3D geometries	▪ Suitable for complex 3D geometries<br>▪ Material versatility<br>▪ No masks required	▪ Suitable for complex 3D geometries<br>▪ High precision<br>▪ Works with many materials<br>▪ No chemical processing	▪ High precision<br>▪ Maskless process<br>▪ Works on various materials<br>▪ Negligible thermal effects with (ultra)short-pulsed laser systems	▪ High precision<br>▪ Maskless process<br>▪ Works on various materials<br>▪ Works on any 3D complex shape<br>▪ Possibility of customization
**Limitations**	▪ Based on solvents and water, thus not suitable for many biodegradable and some polymeric materials.<br>▪ Based on specific chemicals (photoresists) and thermal requirements.<br>▪ Limited by diffraction<br>▪ Requires masks and cleanroom<br>▪ Not suitable for 3D geometries	▪ Slow<br>▪ Expensive<br>▪ Limited to small areas<br>▪ Based on specific thermal requirements<br>▪ Based on solvents and specific chemicals (e-beam resists)<br>▪ Not suitable for 3D geometries	▪ Lower resolution than photo- and e-beam lithography<br>▪ Deformation over time	▪ Lower resolution than lithography<br>▪ Material limitations	▪ Limited to certain geometries<br>▪ Tool wear<br>▪ Material debris	▪ Thermal effects with long-pulsed laser systems<br>▪ Limited feature resolution (>1 µm)<br>▪ Not suitable with some materials	▪ Limited feature resolution (>10 µm)<br>▪ Not suitable with some materials

Table S8. Electrical and design parameters of the coils used in this study for demonstrating the wireless contact lenses.

Design	Outer diameter (mm)	# of layers	# of turns per layer	Turn width (μm)	Trackwidth to μLED (μm)	Self-induct. (nH) @ 1 MHz	Voltage amp. (V)	$I_{RMS}(A); V_{RMS}(V); \phi(deg)$	Input power ($\overline{p_{TX}}$) at TX (W) **	Mutual TX-RX inductance (M, nH); Coupling factor (k)	Output power ($\overline{p_{RX}}$) at RX (W)	Efficiency ($\eta = P_{out}/P_{in} \times 100$)
**TX ***	30	1	4	2000	-	630.2	-	-	-	-	-	-
**RX-D1**	15	1	1	1500	200	3.4	24.5	6.03; 23.2; 87.8	5.37	16.04; 0.35	0.66	12.3
							30.0	7.22; 27.7; 88.6	4.89		0.60	12.3
**RX-D2**	15	1	1	500	200	6.5	19.5	4.80; 18.7; 87.8	3.45	29.96; 0.47	0.76	22.0
							27.0	6.26; 24.0; 88.6	3.68		0.81	22.0
**RX-D3**	15	1	3	200	200	271.4	5.0	1.25; 5.08; 82.1	0.87	90.12; 0.22	0.04	4.84
							15.0	3.69; 14.3; 87.1	2.67		0.13	4.84
**RX-D4**	15	2	3	200	200	964.9	3.0	0.79; 3.41; 77.4	0.59	144.75; 0.19	0.02	3.39
							9.0	1.97; 7.85; 85.4	1.24		0.04	3.23

* The transmitting coil (TX) was placed always coaxially (center-to-center) with respect to the receiving coils (RX). The distance between RX and TX was kept fixed at ~2 mm for the sake of characterizing the input/output power with the different designs.

** The input power depends both on the applied voltage amplitude and frequency, however, for the sake of demonstration, we fixed the frequency at 1 MHz, and we scanned the applied voltage within the operational ranges identified for each design. It should also be noted that the estimation of the input power is affected by the accuracy of the phase delay measurement: when the phase is close to 90˚ (within 2-3 deg), the measurement has to be considered as an approximated result.

Table S9. Processing times and ablation yields for different NIR nanosecond laser ablation jobs. Circles are used as basic designs to test the laser parameters.
HP: hatching pattern; AL: ablation layers; P: power; ScSp: scan speed; PF: pulse frequency; Reps; repetitions.

Design	Radius (µm)	Area (µm^2)	HP	# AL	Laser parameters				Substrate	Processing time (s)	Yield (%) *
					P (%)	ScSp (mm/s)	PF (kHz)	Reps			
**Circle**	30	2826	Slant	1	30	1500	100	3	Ti/Au on Parylene C	0.021	~98
**Circle**	50	7850	Slant	1	30	1500	100	3	Ti/Au on Parylene C	0.031	100
**Circle**	100	31400	Slant	1	30	1500	100	3	Ti/Au on Parylene C	0.056	100
**Circle**	200	125600	Slant	1	30	1500	100	3	Ti/Au on Parylene C	0.121	100
**Circle**	500	785000	Slant	1	30	1500	100	3	Ti/Au on Parylene C	0.38	100
**Circle**	1000	3140000	Slant	1	30	1500	100	3	Ti/Au on Parylene C	1.06	100
**Circle**	2000	12560000	Slant	1	30	1500	100	3	Ti/Au on Parylene C	3.353	100
**Circle**	30	2826	Cross	1	30	1500	100	3	Ti/Au on Parylene C	0.031	~98
**Circle**	50	7850	Cross	1	30	1500	100	3	Ti/Au on Parylene C	0.051	100
**Circle**	100	31400	Cross	1	30	1500	100	3	Ti/Au on Parylene C	0.1	100
**Circle**	200	125600	Cross	1	30	1500	100	3	Ti/Au on Parylene C	0.219	100
**Circle**	500	785000	Cross	1	30	1500	100	3	Ti/Au on Parylene C	0.726	100
**Circle**	1000	3140000	Cross	1	30	1500	100	3	Ti/Au on Parylene C	2.066	100
**Circle**	2000	12560000	Cross	1	30	1500	100	3	Ti/Au on Parylene C	6.613	100
**Circle**	30	2826	Contour	1	30	1500	100	3	Ti/Au on Parylene C	0.016	~98
**Circle**	50	7850	Contour	1	30	1500	100	3	Ti/Au on Parylene C	0.023	100
**Circle**	100	31400	Contour	1	30	1500	100	3	Ti/Au on Parylene C	0.045	100
**Circle**	200	125600	Contour	1	30	1500	100	3	Ti/Au on Parylene C	0.091	100
**Circle**	500	785000	Contour	1	30	1500	100	3	Ti/Au on Parylene C	0.291	100
**Circle**	1000	3140000	Contour	1	30	1500	100	3	Ti/Au on Parylene C	0.883	100
**Circle**	2000	12560000	Contour	1	30	1500	100	3	Ti/Au on Parylene C	3.044	100
**Circle**	30	2826	Slant	1	30	200	100	3	Cu foil on PDMS	0.132	~98
**Circle**	50	7850	Slant	1	30	200	100	3	Cu foil on PDMS	0.247	>98
**Circle**	100	31400	Slant	1	30	200	100	3	Cu foil on PDMS	0.645	100
**Circle**	200	125600	Slant	1	30	200	100	3	Cu foil on PDMS	1.911	100
**Circle**	500	785000	Slant	1	30	200	100	3	Cu foil on PDMS	9.487	100
**Circle**	1000	3140000	Slant	1	30	200	100	3	Cu foil on PDMS	34.679	100
**Circle**	2000	12560000	Slant	1	30	200	100	3	Cu foil on PDMS	132.179	100
**Circle**	30	2826	Cross	1	30	200	100	3	Cu foil on PDMS	0.224	~98
**Circle**	50	7850	Cross	1	30	200	100	3	Cu foil on PDMS	0.441	>98
**Circle**	100	31400	Cross	1	30	200	100	3	Cu foil on PDMS	1.201	100
**Circle**	200	125600	Cross	1	30	200	100	3	Cu foil on PDMS	3.663	100
**Circle**	500	785000	Cross	1	30	200	100	3	Cu foil on PDMS	18.619	100
**Circle**	1000	3140000	Cross	1	30	200	100	3	Cu foil on PDMS	68.667	100
**Circle**	2000	12560000	Cross	1	30	200	100	3	Cu foil on PDMS	262.996	100
**Circle**	30	2826	Contour	1	30	200	100	3	Cu foil on PDMS	0.087	~98
**Circle**	50	7850	Contour	1	30	200	100	3	Cu foil on PDMS	0.173	>98
**Circle**	100	31400	Contour	1	30	200	100	3	Cu foil on PDMS	0.504	100
**Circle**	200	125600	Contour	1	30	200	100	3	Cu foil on PDMS	1.66	100
**Circle**	500	785000	Contour	1	30	200	100	3	Cu foil on PDMS	9.107	100
**Circle**	1000	3140000	Contour	1	30	200	100	3	Cu foil on PDMS	34.8	100

**Circle**	2000	12560000	Contour	1	30	200	100	3	Cu foil on PDMS	135.728	100
**Circle**	100	31400	Cross	1	30	1000	400	1	Stainless steel	0.04	>98
**Circle**	100	31400	Slant	1	30	1000	400	1	Stainless steel	0.024	>98
**Circle**	100	31400	Contour	1	30	1000	400	1	Stainless steel	0.02	>98
**Circle**	100	31400	Cross	1	30	1000	400	50	Stainless steel	1.62	~98
**Circle**	100	31400	Slant	1	30	1000	400	50	Stainless steel	0.849	~98
**Circle**	100	31400	Contour	1	30	1000	400	50	Stainless steel	0.628	~98
**Circle**	100	31400	Cross	1	30	1000	400	100	Stainless steel	3.23	~98
**Circle**	100	31400	Slant	1	30	1000	400	100	Stainless steel	1.688	~98
**Circle**	100		Contour	1	30	1000	400	100	Stainless steel	1.246	~98
**Circle**	100	31400	Contour	1	30	1000	400	1	Stainless steel	0.02	>98
**Circle**	100	31400	Contour	1	30	1000	300	1	Stainless steel	0.02	>98
**Circle**	100	31400	Contour	1	30	1000	200	1	Stainless steel	0.02	>98
**Circle**	100	31400	Contour	1	30	1000	100	1	Stainless steel	0.02	>98
**Circle**	100	31400	Contour	1	30	1000	50	1	Stainless steel	0.02	>98
**Circle**	100	31400	Contour	1	30	1000	400	10	Stainless steel	0.132	>98
**Circle**	100	31400	Contour	1	30	1000	300	10	Stainless steel	0.132	>98
**Circle**	100	31400	Contour	1	30	1000	200	10	Stainless steel	0.132	>98
**Circle**	100	31400	Contour	1	30	1000	100	10	Stainless steel	0.132	>98
**Circle**	100	31400	Contour	1	30	1000	50	10	Stainless steel	0.132	>98
**Circle**	100	31400	Contour	1	30	1000	400	20	Stainless steel	0.256	~98
**Circle**	100	31400	Contour	1	30	1000	300	20	Stainless steel	0.256	~98
**Circle**	100	31400	Contour	1	30	1000	200	20	Stainless steel	0.256	~98
**Circle**	100	31400	Contour	1	30	1000	100	20	Stainless steel	0.256	~98
**Circle**	100	31400	Contour	1	30	1000	50	20	Stainless steel	0.256	~98
**Circle**	100	31400	Contour	1	30	1000	400	50	Stainless steel	0.628	~98
**Circle**	100	31400	Contour	1	30	1000	300	50	Stainless steel	0.628	~98
**Circle**	100	31400	Contour	1	30	1000	200	50	Stainless steel	0.628	~98
**Circle**	100	31400	Contour	1	30	1000	100	50	Stainless steel	0.628	~98
**Circle**	100	31400	Contour	1	30	1000	50	50	Stainless steel	0.628	~98
**Circle**	100	31400	Contour	1	30	1000	400	100	Stainless steel	1.246	~98
**Circle**	100	31400	Contour	1	30	1000	300	100	Stainless steel	1.246	~98
**Circle**	100	31400	Contour	1	30	1000	200	100	Stainless steel	1.246	~98
**Circle**	100	31400	Contour	1	30	1000	100	100	Stainless steel	1.246	~98
**Circle**	100	31400	Contour	1	30	1000	50	100	Stainless steel	1.246	~98
**Circle**	100	31400	Contour	1	30	100	50	1	Stainless steel	0.051	~98
**Circle**	100	31400	Contour	1	30	200	50	1	Stainless steel	0.032	~98
**Circle**	100	31400	Contour	1	30	500	50	1	Stainless steel	0.021	>98
**Circle**	100	31400	Contour	1	30	1000	50	1	Stainless steel	0.02	>98
**Circle**	100	31400	Contour	1	30	1500	50	1	Stainless steel	0.02	>98
**Circle**	100	31400	Contour	1	30	2000	50	1	Stainless steel	0.02	~98
**Circle**	100	31400	Contour	1	30	3000	50	1	Stainless steel	0.02	~98
**Circle**	100	31400	Contour	1	30	4000	50	1	Stainless steel	0.02	~98

**Circle**	100	31400	Contour	1	30	100	50	50	Stainless steel	2.185	~98
**Circle**	100	31400	Contour	1	30	200	50	50	Stainless steel	1.2405	~98
**Circle**	100	31400	Contour	1	30	500	50	50	Stainless steel	0.68	~98
**Circle**	100	31400	Contour	1	30	1000	50	50	Stainless steel	0.6225	~98
**Circle**	100	31400	Contour	1	30	1500	50	50	Stainless steel	0.614	~98
**Circle**	100	31400	Contour	1	30	2000	50	50	Stainless steel	0.6565	~98
**Circle**	100	31400	Contour	1	30	3000	50	50	Stainless steel	0.6105	~98
**Circle**	100	31400	Contour	1	30	4000	50	50	Stainless steel	0.594	~98
**Circle**	100	31400	Contour	1	30	100	50	100	Stainless steel	4.37	~98
**Circle**	100	31400	Contour	1	30	200	50	100	Stainless steel	2.481	~98
**Circle**	100	31400	Contour	1	30	500	50	100	Stainless steel	1.36	~98
**Circle**	100	31400	Contour	1	30	1000	50	100	Stainless steel	1.245	~98
**Circle**	100	31400	Contour	1	30	1500	50	100	Stainless steel	1.228	~98
**Circle**	100	31400	Contour	1	30	2000	50	100	Stainless steel	1.313	~98
**Circle**	100	31400	Contour	1	30	3000	50	100	Stainless steel	1.221	~98
**Circle**	100	31400	Contour	1	30	4000	50	100	Stainless steel	1.188	~98
**Circle**	100	31400	Contour	1 (1 circle)	30	1000	50	100	Stainless steel	1.246	~98
**Circle**	100	31400	Contour	1 (2 circles)	30	1000	50	100	Stainless steel	2.492	~98
**Circle**	100	31400	Contour	1 (3 circles)	30	1000	50	100	Stainless steel	3.738	~98
**Circle**	100	31400	Contour	1 (4 circles)	30	1000	50	100	Stainless steel	4.984	~98
**Circle**	100	31400	Contour	1 (5 circles)	30	1000	50	100	Stainless steel	6.23	~98
**Circle**	100	31400	Contour	1 (6 circles)	30	1000	50	100	Stainless steel	7.476	~98
**Circle**	100	31400	Contour	1 (7 circles)	30	1000	50	100	Stainless steel	8.722	~98
**Circle**	100	31400	Contour	1 (8 circles)	30	1000	50	100	Stainless steel	9.968	~98
**Circle**	100	31400	Contour	1 (9 circles)	30	1000	50	100	Stainless steel	11.214	~98
**Circle**	100	31400	Contour	1 (10 circles)	30	1000	50	100	Stainless steel	12.46	~98
**Circle**	100	31400	Contour	1	30	1000	50	100	Stainless steel	1.245	~98
**Circle**	100	31400	Contour	2	30	1000	50	100	Stainless steel	2.483	~98
**Circle**	100	31400	Contour	3	30	1000	50	100	Stainless steel	3.718	~98
**Circle**	100	31400	Contour	4	30	1000	50	100	Stainless steel	4.954	~98
**Circle**	100	31400	Contour	5	30	1000	50	100	Stainless steel	6.18	~98
**Circle**	100	31400	Contour	6	30	1000	50	100	Stainless steel	7.425	~98
**Circle**	100	31400	Contour	7	30	1000	50	100	Stainless steel	8.663	~98
**Circle**	100	31400	Contour	8	30	1000	50	100	Stainless steel	9.899	~98
**Circle**	100	31400	Contour	9	30	1000	50	100	Stainless steel	11.135	~98
**Circle**	100	31400	Contour	10	30	1000	50	100	Stainless steel	12.372	~98
**Circle**	200	125600	Contour	1	30	100	50	1	Stainless steel	0.49	>98
**Circle**	200	125600	Contour	1	30	100	50	10	Stainless steel	5.32	>98
**Circle**	200	125600	Contour	1	30	100	50	20	Stainless steel	10.84	~98
**Circle**	200	125600	Contour	1	30	100	50	50	Stainless steel	27.65	~98
**Circle**	200	125600	Contour	1	30	100	50	100	Stainless steel	55.42	~98
**Circle**	200	125600	Contour	1	30	500	50	1	Stainless steel	0.04	>98
**Circle**	200	125600	Contour	1	30	500	50	10	Stainless steel	1.09	>98
**Circle**	200	125600	Contour	1	30	500	50	20	Stainless steel	2.22	~98
**Circle**	200	125600	Contour	1	30	500	50	50	Stainless steel	5.7	~98
**Circle**	200	125600	Contour	1	30	500	50	100	Stainless steel	11.6	~98
**Circle**	200	125600	Contour	1	30	1000	50	1	Stainless steel	0.01	>98

**Circle**	200	125600	Contour	1	30	1000	50	10	Stainless steel	0.65	>98
**Circle**	200	125600	Contour	1	30	1000	50	20	Stainless steel	1.33	~98
**Circle**	200	125600	Contour	1	30	1000	50	50	Stainless steel	3.49	~98
**Circle**	200	125600	Contour	1	30	1000	50	100	Stainless steel	7.1	~98
**Circle**	200	125600	Contour	1	30	1500	50	1	Stainless steel	0.01	>98
**Circle**	200	125600	Contour	1	30	1500	50	10	Stainless steel	0.55	>98
**Circle**	200	125600	Contour	1	30	1500	50	20	Stainless steel	1.2	~98
**Circle**	200	125600	Contour	1	30	1500	50	50	Stainless steel	3.08	~98
**Circle**	200	125600	Contour	1	30	1500	50	100	Stainless steel	6.29	~98
**Circle**	500	785000	Contour	1	30	100	50	1	Stainless steel	3.19	>98
**Circle**	500	785000	Contour	1	30	100	50	10	Stainless steel	33.05	>98
**Circle**	500	785000	Contour	1	30	100	50	20	Stainless steel	66.42	~98
**Circle**	500	785000	Contour	1	30	100	50	50	Stainless steel	166.1	~98
**Circle**	500	785000	Contour	1	30	100	50	100	Stainless steel	332.7	~98
**Circle**	500	785000	Contour	1	30	500	50	1	Stainless steel	0.62	>98
**Circle**	500	785000	Contour	1	30	500	50	10	Stainless steel	6.65	>98
**Circle**	500	785000	Contour	1	30	500	50	20	Stainless steel	13.4	~98
**Circle**	500	785000	Contour	1	30	500	50	50	Stainless steel	33.97	~98
**Circle**	500	785000	Contour	1	30	500	50	100	Stainless steel	68.18	~98
**Circle**	500	785000	Contour	1	30	1000	50	1	Stainless steel	0.3	>98
**Circle**	500	785000	Contour	1	30	1000	50	10	Stainless steel	3.47	>98
**Circle**	500	785000	Contour	1	30	1000	50	20	Stainless steel	7.09	~98
**Circle**	500	785000	Contour	1	30	1000	50	50	Stainless steel	17.81	~98
**Circle**	500	785000	Contour	1	30	1000	50	100	Stainless steel	35.81	~98
**Circle**	500	785000	Contour	1	30	1500	50	1	Stainless steel	0.18	>98
**Circle**	500	785000	Contour	1	30	1500	50	10	Stainless steel	2.48	>98
**Circle**	500	785000	Contour	1	30	1500	50	20	Stainless steel	5.01	~98
**Circle**	500	785000	Contour	1	30	1500	50	50	Stainless steel	12.81	~98
**Circle**	500	785000	Contour	1	30	1500	50	100	Stainless steel	25.77	~98
**Circle**	1000	3140000	Contour	1	30	100	50	1	Stainless steel	12.86	>98
**Circle**	1000	3140000	Contour	1	30	100	50	10	Stainless steel	130.6	>98
**Circle**	1000	3140000	Contour	1	30	100	50	20	Stainless steel	261.3	~98
**Circle**	1000	3140000	Contour	1	30	100	50	50	Stainless steel	655.5	~98
**Circle**	1000	3140000	Contour	1	30	100	50	100	Stainless steel	1310	~98
**Circle**	1000	3140000	Contour	1	30	500	50	1	Stainless steel	2.53	>98
**Circle**	1000	3140000	Contour	1	30	500	50	10	Stainless steel	26.41	>98
**Circle**	1000	3140000	Contour	1	30	500	50	20	Stainless steel	53.05	~98
**Circle**	1000	3140000	Contour	1	30	500	50	50	Stainless steel	132.8	~98
**Circle**	1000	3140000	Contour	1	30	500	50	100	Stainless steel	265.8	~98
**Circle**	1000	3140000	Contour	1	30	1000	50	1	Stainless steel	1.3	>98
**Circle**	1000	3140000	Contour	1	30	1000	50	10	Stainless steel	13.37	>98
**Circle**	1000	3140000	Contour	1	30	1000	50	20	Stainless steel	26.96	~98
**Circle**	1000	3140000	Contour	1	30	1000	50	50	Stainless steel	67.8	~98
**Circle**	1000	3140000	Contour	1	30	1000	50	100	Stainless steel	135.9	~98
**Circle**	1000	3140000	Contour	1	30	1500	50	1	Stainless steel	0.87	>98
**Circle**	1000	3140000	Contour	1	30	1500	50	10	Stainless steel	9.05	>98
**Circle**	1000	3140000	Contour	1	30	1500	50	20	Stainless steel	18.28	~98
**Circle**	1000	3140000	Contour	1	30	1500	50	50	Stainless steel	46.34	~98
**Circle**	1000	3140000	Contour	1	30	1500	50	100	Stainless steel	92.84	~98

**Circle**	2000	6280000	Contour	1	30	100	50	1	Stainless steel	51.82	>98
**Circle**	2000	6280000	Contour	1	30	100	50	10	Stainless steel	522	>98
**Circle**	2000	6280000	Contour	1	30	100	50	20	Stainless steel	1043	~98
**Circle**	2000	6280000	Contour	1	30	100	50	50	Stainless steel	2610	~98
**Circle**	2000	6280000	Contour	1	30	100	50	100	Stainless steel	5227	~98
**Circle**	2000	6280000	Contour	1	30	500	50	1	Stainless steel	10.21	>98
**Circle**	2000	6280000	Contour	1	30	500	50	10	Stainless steel	104.4	>98
**Circle**	2000	6280000	Contour	1	30	500	50	20	Stainless steel	208.2	~98
**Circle**	2000	6280000	Contour	1	30	500	50	50	Stainless steel	524.2	~98
**Circle**	2000	6280000	Contour	1	30	500	50	100	Stainless steel	1049	~98
**Circle**	2000	6280000	Contour	1	30	1000	50	1	Stainless steel	5.18	>98
**Circle**	2000	6280000	Contour	1	30	1000	50	10	Stainless steel	52.94	>98
**Circle**	2000	6280000	Contour	1	30	1000	50	20	Stainless steel	105.7	~98
**Circle**	2000	6280000	Contour	1	30	1000	50	50	Stainless steel	265.1	~98
**Circle**	2000	6280000	Contour	1	30	1000	50	100	Stainless steel	532.5	~98
**Circle**	2000	6280000	Contour	1	30	1500	50	1	Stainless steel	3.39	>98
**Circle**	2000	6280000	Contour	1	30	1500	50	10	Stainless steel	35.34	>98
**Circle**	2000	6280000	Contour	1	30	1500	50	20	Stainless steel	70.91	~98
**Circle**	2000	6280000	Contour	1	30	1500	50	50	Stainless steel	177	~98
**Circle**	2000	6280000	Contour	1	30	1500	50	100	Stainless steel	355	~98

* The laser ablation yield is defined here as the ratio of successfully fabricated features that meet the predefined geometric criteria (designed shape) to the total number of features attempted under a given set of laser processing conditions. In other words, yield quantifies the reproducibility and reliability of the ablation process, accounting for defects such as incomplete material removal, over-ablation, debris redeposition, delamination, or dimensional deviations beyond the acceptable tolerance. High yield indicates that the process is stable, well-controlled, and scalable, while reductions in yield of some units point to issues with parameter optimization, material heterogeneity, or system alignment. In this case, the yield also related to the accuracy and repeatability of the resulting shape after laser ablation.

Table S10. Processing times, yield and evaluation of automation/manual interventions for the devices presented in this work. HP: hatching pattern; AL: ablation layers; P: power; ScSp: scan speed; PF: pulse frequency; Reps; repetitions.

Device	Size (mm x mm)	HP	# AL	Laser parameters				Substrate	Proc. time (s)	Yield of laser ablation (%) (Y_1) *	Yield of device assembly (%) (Y_2) **	Overall yield (%) ($Y_1 \times Y_2$)	Laser-based steps	Manual interventions for device assembly	Overall automation (out of 5) ***
				P (%)	ScSp (mm/s)	PF (kHz)	Reps								
Electro-haptic 2D-electrode device (finger)	11.69 x 24.58	Slant	1	30	1500	100	3	Ti/Au on Parylene C	182.1	100	90	90	▪ Electrodes patterning<br>▪ Patterning of medical tape	▪ Device connections<br>▪ Lamination of medical tape<br>▪ Attachment of 3D milli-needles	4.5
Electro-haptic 3D-electrode device (finger)	11.69 x 24.58	Slant	1	30	1500	100	3		182.1	100	90	90			4
Electro-haptic 2D-electrode device (wrist)	22.50 x 45.00	Slant	1	30	1500	100	3		237.1	100	90	90			4.5
Electro-haptic 3D-electrode device (wrist)	22.50 x 45.00	Slant	1	30	1500	100	3		237.1	100	90	90			4
Wireless contact lens (Coil design D1)	24.00 x 24.00	Slant	4	30	200	100	20	Cu foil on PDMS	20298.1	95	90	85.5	▪ Sequential patterning of Cu foils on PDMS<br>▪ Shaping the outline of the final device (PDMS)	▪ Thermoforming of the Cu foils<br>▪ Integration of μLED<br>▪ Lamination of Cu foils and PDMS (depending on the design)	4.5
Wireless contact lens (Coil design D2)	24.00 x 24.00	Slant	4	30	200	100	20		17609.7	95	90	85.5			4.5
Wireless contact lens (Coil design D3)	16.00 x 16.00	Slant	4	30	200	100	20		7782.4 (bottom) 8092.5 (top)	95	90	85.5			4
Wireless contact lens (Coil design D4)	16.00 x 16.00	Slant	4	30	200	100	20		7782.4 (bottom) 7782.4 (top)	95	90	85.5			4
2D MEAs	18.00 x 30.00	Slant	1	30	1500	100	5	Ti/Au on Glass	200.4	100	90	90	▪ Ti/Au electrodes patterning<br>▪ Patterning of Parylene C encapsulation	▪ Device connections	4.5
3D MEAs	6.23 x 5.33	Slant	14	30	500	100	100	Stainless steel	9991.8	95	80	76	▪ Ti/Au electrodes patterning<br>▪ Patterning of stainless-steel MN electrodes<br>▪ Patterning of Parylene C encapsulation	▪ Device connections<br>▪ Integration of MN electrodes on flat MEAs	3.5

* Y_1 is defined in the same way as the laser ablation yield in Table S2.
** Y_2 is defined as the number of functional devices after overall assembly, out of the initial number of devices in the batch after laser ablation.
*** The scale and the quantitative evaluation of the overall automation are based on a personal assessment of the authors who fabricated the devices: 0 and 5 correspond to devices fabricated with a completely manual/ completely automated process.

REFERENCES AND NOTES

1. Y. S. Choi, R. T. Yin, A. Pfenniger, J. Koo, R. Avila, K. Benjamin Lee, S. W. Chen, G. Lee, G. Li, Y. Qiao, A. Murillo-Berlioz, A. Kiss, S. Han, S. M. Lee, C. Li, Z. Xie, Y.-Y. Chen, A. Burrell, B. Geist, H. Jeong, J. Kim, H.-J. Yoon, A. Banks, S.-K. Kang, Z. J. Zhang, C. R. Haney, A. V. Sahakian, D. Johnson, T. Efimova, Y. Huang, G. D. Trachiotis, B. P. Knight, R. K. Arora, I. R. Efimov, J. A. Rogers, Fully implantable and bioresorbable cardiac pacemakers without leads or batteries. *Nat. Biotechnol.* **39**, 1228–1238 (2021).

2. C. M. Boutry, L. Beker, Y. Kaizawa, C. Vassos, H. Tran, A. C. Hinckley, R. Pfattner, S. Niu, J. Li, J. Claverie, Z. Wang, J. Chang, P. M. Fox, Z. Bao, Biodegradable and flexible arterial-pulse sensor for the wireless monitoring of blood flow. *Nat. Biomed. Eng.* **3**, 47–57 (2019).

3. X. Yu, W. Shou, B. K. Mahajan, X. Huang, H. Pan, Materials, processes, and facile manufacturing for bioresorbable electronics: A review. *Adv. Mater.* **30**, 1707624 (2018).

4. F. Fallegger, G. Schiavone, S. Lacour, Conformable hybrid systems for implantable bioelectronic interfaces. *Adv. Mater.* **32**, 1903904 (2019).

5. S.-W. Hwang, J.-K. Song, X. Huang, H. Cheng, S.-K. Kang, B. H. Kim, J.-H. Kim, S. Yu, Y. Huang, J. A. Rogers, High-performance biodegradable/transient electronics on biodegradable polymers. *Adv. Mater.* **26**, 3905–3911 (2014).

6. Q. Yang, Z. Hu, M.-H. Seo, Y. Xu, Y. Yan, Y.-H. Hsu, J. Berkovich, K. Lee, T.-L. Liu, S. McDonald, H. Nie, H. Oh, M. Wu, J.-T. Kim, S. A. Miller, Y. Jia, S. Butun, W. Bai, H. Guo, J. Choi, A. Banks, W. Z. Ray, Y. Kozorovitskiy, M. L. Becker, M. A. Pet, M. R. MacEwan, J.-K. Chang, H. Wang, Y. Huang, J. A. Rogers, High-speed, scanned laser structuring of multi-layered eco/bioresorbable materials for advanced electronic systems. *Nat. Commun.* **13**, 6518 (2022).

7. L. Coles, D. Ventrella, A. Carnicer-Lombarte, A. Elmi, J. G. Troughton, M. Mariello, S. El Hadwe, B. J. Woodington, M. L. Bacci, G. G. Malliaras, D. G. Barone, C. M. Proctor, Origami-inspired soft fluidic actuation for minimally invasive large-area electrocorticography. *Nat. Commun.* **15**, 6290 (2024).

8. Y. H. Cho, Y.-G. Park, S. Kim, J.-U. Park, 3D electrodes for bioelectronics. *Adv. Mater.* **33**, 2005805 (2021).

9. A. J. Boys, A. Carnicer-Lombarte, A. Güemes-Gonzalez, D. C. van Niekerk, S. Hilton, D. G. Barone, C. M. Proctor, R. M. Owens, G. G. Malliaras, 3D bioelectronics with a remodellable matrix for long-term tissue integration and recording. *Adv. Mater. Deerfield Beach Fla* **35**, e2207847 (2023).

10. A. Savva, J. Saez, A. Withers, C. Barberio, V. Stoeger, S. Elias-Kirma, Z. Lu, C.-M. Moysidou, K. Kallitsis, C. Pitsalidis, R. M. Owens, 3D organic bioelectronics for electrical monitoring of human adult stem cells. *Mater. Horiz.* **10**, 3589–3600 (2023).

11. R. Y. Tay, Y. Song, D. R. Yao, W. Gao, Direct-ink-writing 3D-printed bioelectronics. *Mater. Today* **71**, 135–151 (2023).

12. D. J. Joe, S. Kim, J. H. Park, D. Y. Park, H. E. Lee, T. H. Im, I. Choi, R. S. Ruoff, K. J. Lee, Laser–material interactions for flexible applications. *Adv. Mater.* **29**, 1606586 (2017).

13. S. K. Sundaram, E. Mazur, Inducing and probing non-thermal transitions in semiconductors using femtosecond laser pulses. *Nat. Mater.* **1**, 217–224 (2002).

14. S. Hermann, N.-P. Harder, R. Brendel, D. Herzog, H. Haferkamp, Picosecond laser ablation of SiO_2 layers on silicon substrates. *Appl. Phys. A* **99**, 151–158 (2010).

15. J. H. Park, H. E. Lee, C. K. Jeong, D. H. Kim, S. K. Hong, K.-I. Park, K. J. Lee, Self-powered flexible electronics beyond thermal limits. *Nano Energy* **56**, 531–546 (2019).

16. B. N. Chichkov, C. Momma, S. Nolte, F. von Alvensleben, A. Tünnermann, Femtosecond, picosecond and nanosecond laser ablation of solids. *Appl. Phys. A* **63**, 109–115 (1996).

17. V. Kumar, R. Verma, S. Kango, V. S. Sharma, Recent progresses and applications in laser-based surface texturing systems. *Mater Today Commun* **26**, 101736 (2021).

18. W. J. Keller, N. Shen, A. M. Rubenchik, S. Ly, R. Negres, R. N. Raman, J.-H. Yoo, G. Guss, J. S. Stolken, M. J. Matthews, J. D. Bude, Physics of picosecond pulse laser ablation. *J. Appl. Phys.* **125**, 085103 (2019).

19. H. Mustafa, D. T. A. Matthews, G. R. B. E. Römer, Wavelength dependence of picosecond-pulsed laser ablation of hot-dip galvanized steel. *Appl. Phys. A* **128**, 296 (2022).

20. F. Vidal, T. W. Johnston, S. Laville, O. Barthélemy, M. Chaker, B. Le Drogoff, J. Margot, M. Sabsabi, Critical-point phase separation in laser ablation of conductors. *Phys. Rev. Lett.* **86**, 2573–2576 (2001).

21. D. von der Linde, K. Sokolowski-Tinten, The physical mechanisms of short-pulse laser ablation. *Appl. Surf. Sci.* **154–155**, 1–10 (2000).

22. D. von der Linde, H. Schüler, Breakdown threshold and plasma formation in femtosecond laser–solid interaction. *JOSA B* **13**, 216–222 (1996).

23. D. Bäuerle, "Nanosecond-laser ablation," in *Laser Processing and Chemistry*, D. Bäuerle, Ed. (Springer, 2000), pp. 221–257.

24. Y. Wang, M. Zhang, Y. Dong, J. Zhao, X. Zhu, Y. Li, L. Fan, H. Leng, Morphology modelling and validation in nanosecond pulsed laser ablation of metallic materials. *Precis. Eng.* **79**, 34–42 (2023).

25. J. Bian, L. Zhou, X. Wan, C. Zhu, B. Yang, Y. Huang, Laser transfer, printing, and assembly techniques for flexible electronics. *Adv. Electron. Mater.* **5**, 1800900 (2019).

26. W. Pfleging, A. Ludwig, K. Seemann, R. Preu, H. Mäckel, S. W. Glunz, Laser micromachining for applications in thin film technology. *Appl. Surf. Sci.* **154–155**, 633–639 (2000).

27. H.-L. Yeh, J. V. Garich, I. R. Akamine, J. M. Blain-Christen, S. A. Hara, "Laser micromachining of thin-film polyimide microelectrode arrays: Alternative processes to photolithography" (American Society of Mechanical Engineers Digital Collection, 2020); https://dx.doi.org/10.1115/DMD2020-9057.

28. B. Pecholt, M. Vendan, Y. Dong, P. Molian, Ultrafast laser micromachining of 3C-SiC thin films for MEMS device fabrication. *Int. J. Adv. Manuf. Technol.* **39**, 239–250 (2008).

29. A. Butkutė, T. Jurkšas, T. Baravykas, B. Leber, G. Merkininkaitė, R. Žilėnaitė, D. Čereška, A. Gulla, M. Kvietkauskas, K. Marcinkevičiūtė, P. Schemmer, K. Strupas, Combined femtosecond laser glass microprocessing for liver-on-chip device fabrication. *Materials* **16**, 2174 (2023).

30. A. Rizzo, E. D. Lemma, F. Pisano, M. Pisanello, L. Sileo, M. De Vittorio, F. Pisanello, Laser micromachining of tapered optical fibers for spatially selective control of neural activity. *Microelectron. Eng.* **192**, 88–95 (2018).

31. R. Bo, S. Xu, Y. Yang, Y. Zhang, Mechanically-guided 3D assembly for architected flexible electronics. *Chem. Rev.* **123**, 11137–11189 (2023).

32. C. W. Ha, Y. Son, Development of the multi-directional ablation process using the femtosecond laser to create a pattern on the lateral side of a 3D microstructure. *Sci. Rep.* **13**, 4781 (2023).

33. A. K. Verma, R. K. Soni, Laser-textured Pd-Au hierarchical superhydrophobic micro-arrays for ultralow SERS detection. *Surf Interfaces* **39**, 102896 (2023).

34. Y. Zhao, H. Liu, T. Yu, M. Hong, Fabrication of high hardness microarray diamond tools by femtosecond laser ablation. *Opt. Laser Technol.* **140**, 107014 (2021).

35. S. Amini, W. Seche, N. May, H. Choi, P. Tavousi, S. Shahbazmohamadi, Femtosecond laser hierarchical surface restructuring for next generation neural interfacing electrodes and microelectrode arrays. *Sci. Rep.* **12**, 13966 (2022).

36. H. Peng, I. Kopic, S. Ratnakar Potfode, T. F. Teshima, G. Al Boustani, L. Hiendlmeier, C. Wang, M. Zahid Hussain, B. Özkale, R. A. Fischer, B. Wolfrum, Laser-patterned epoxy-based 3D microelectrode arrays for extracellular recording. *Nanoscale* **16**, 14295–14301 (2024).

37. G. A. J. Markillie, H. J. Baker, F. J. Villarreal, D. R. Hall, Effect of vaporization and melt ejection on laser machining of silica glass micro-optical components. *Appl. Optics* **41**, 5660–5667 (2002).

38. V. R. Borkowska, A. McConnell, S. Vijayakumar, A. Stokes, A. D. Roche, A haptic sleeve as a method of mechanotactile feedback restoration for myoelectric hand prosthesis users. *Front. Rehabil. Sci.* **3**, 806479 (2022).

39. P. B. Shull, D. D. Damian, Haptic wearables as sensory replacement, sensory augmentation and trainer – A review. *J. NeuroEngineering Rehabil.* **12**, 59 (2015).

40. M. D. Fletcher, C. A. Verschuur, Electro-haptic stimulation: A new approach for improving Cochlear-implant listening. *Front. Neurosci.* **15**, 581414 (2021).

41. K. Kim, A review of haptic feedback through peripheral nerve stimulation for upper extremity prosthetics. *Curr. Opin. Biomed. Eng.* **21**, 100368 (2022).

42. T.-H. Yang, J. R. Kim, H. Jin, H. Gil, J.-H. Koo, H. J. Kim, Recent advances and opportunities of active materials for haptic technologies in virtual and augmented reality. *Adv. Funct. Mater.* **31**, 2008831 (2021).

43. Y. Gao, K. Yao, S. Jia, Y. Huang, G. Zhao, B. Zhang, Y. Liu, X. Yu, Advances in materials for haptic skin electronics. *Matter* **7**, 2826–2845 (2024).

44. P. B. Perera, H. Marasinghe, T. Takami, H. Kajimoto, A. Withana, "Integrating force sensing with electro-tactile feedback in 3D printed haptic interfaces," in *Proceedings of the 2024 ACM International Symposium on Wearable Computers* (Association for Computing Machinery, 2024), pp. 48–54; https://dl.acm.org/doi/10.1145/3675095.3676612.

45. N. Kitamura, J. Chim, N. Miki, Electrotactile display using microfabricated micro-needle array. *J. Micromech. Microeng.* **25**, 025016 (2015).

46. B. M. Dzidek, M. J. Adams, J. W. Andrews, Z. Zhang, S. A. Johnson, Contact mechanics of the human finger pad under compressive loads. *J. R. Soc. Interface* **14**, 20160935 (2017).

47. D. H. Keum, S.-K. Kim, J. Koo, G.-H. Lee, C. Jeon, J. W. Mok, B. H. Mun, K. J. Lee, E. Kamrani, C.-K. Joo, Wireless smart contact lens for diabetic diagnosis and therapy. *Sci. Adv.* **6**, eaba3252 (2020).

48. J. Kim, M. Kim, M.-S. Lee, K. Kim, S. Ji, Y.-T. Kim, J. Park, K. Na, K.-H. Bae, H. Kyun Kim, Wearable smart sensor systems integrated on soft contact lenses for wireless ocular diagnostics. *Nat. Commun.* **8**, 14997 (2017).

49. H. Yang, H. Zhu, H. Liu, Z. Mao, J. Luo, S. Zhu, Z. Hu, S. Yuan, F. Xu, Intraocular pressure monitoring smart contact lens with high environmental stability. *Adv. Funct. Mater.* **34**, 2400722 (2024).

50. M. Mariello, C. M. Proctor, Wireless power and data transfer technologies for flexible bionic and bioelectronic interfaces: Materials and applications. *Adv. Mater. Technol.* **10**, 2400797 (2025).

51. J. Park, J. Kim, S.-Y. Kim, W. H. Cheong, J. Jang, Y.-G. Park, K. Na, Y.-T. Kim, J. H. Heo, C. Y. Lee, Soft, smart contact lenses with integrations of wireless circuits, glucose sensors, and displays. *Sci. Adv.* **4**, eaap9841 (2018).

52. T. Takamatsu, Y. Chen, T. Yoshimasu, M. Nishizawa, T. Miyake, Highly efficient, flexible wireless-powered circuit printed on a moist, soft contact lens. *Adv. Mater. Technol.* **4**, 1800671 (2019).

53. J. Zhang, K. Kim, H. J. Kim, D. Meyer, W. Park, S. A. Lee, Y. Dai, B. Kim, H. Moon, J. V. Shah, K. E. Harris, B. Collar, K. Liu, P. Irazoqui, H. Lee, S. A. Park, P. S. Kollbaum, B. W. Boudouris, C. H. Lee, Smart soft contact lenses for continuous 24-hour monitoring of intraocular pressure in glaucoma care. *Nat. Commun.* **13**, 5518 (2022).

54. X. Ma, S. Ahadian, S. Liu, J. Zhang, S. Liu, T. Cao, W. Lin, D. Wu, N. R. de Barros, M. R. Zare, Smart contact lenses for biosensing applications. *Adv. Intell. Syst.* **3**, 2000263 (2021).

55. G.-H. Lee, C. Jeon, J. W. Mok, S. Shin, S.-K. Kim, H. H. Han, S.-J. Kim, S. H. Hong, H. Kim, C.-K. Joo, J.-Y. Sim, S. K. Hahn, Smart wireless near-infrared light emitting contact lens for the treatment of diabetic retinopathy. *Adv. Sci.* **9**, 2103254 (2022).

56. P. S. Yarmolenko, E. J. Moon, C. Landon, A. Manzoor, D. W. Hochman, B. L. Viglianti, M. W. Dewhirst, Thresholds for thermal damage to normal tissues: An update. *Int. J. Hyperth. Off. J. Eur. Soc. Hyperthermic Oncol. North Am. Hyperth. Group* **27**, 320–343 (2011).

57. S. Regal, J. Troughton, R. Delattre, T. Djenizian, M. Ramuz, Changes in temperature inside an optomechanical model of the human eye during emulated transscleral cyclophotocoagulation. *Biomed. Opt. Express* **11**, 4548–4559 (2020).

58. H.-Y. Tan, H. Cho, L. P. Lee, Human mini-brain models. *Nat. Biomed. Eng.* **5**, 11–25 (2021).

59. J. Song, H. E. Jeong, A. Choi, H. N. Kim, Monitoring of electrophysiological functions in brain-on-a-chip and brain organoids. *Adv. NanoBiomed Res.* **4**, 2400052 (2024).

60. M. J. Silvosa, N. R. Mercado, N. Merlock, S. Vidhate, R. Mejia-Alvarez, T. T. Yuan, A. M. Willis, Z. R. Lybrand, Understanding primary blast injury: High frequency pressure acutely disrupts neuronal network dynamics in cerebral organoids. *J. Neurotrauma* **39**, 1575–1590 (2022).

61. H. Cai, Z. Ao, C. Tian, Z. Wu, H. Liu, J. Tchieu, M. Gu, K. Mackie, F. Guo, Brain organoid reservoir computing for artificial intelligence. *Nat. Electron.* **6**, 1032–1039 (2023).

62. Y. Park, C. K. Franz, H. Ryu, H. Luan, K. Y. Cotton, J. U. Kim, T. S. Chung, S. Zhao, A. Vazquez-Guardado, D. S. Yang, K. Li, R. Avila, J. K. Phillips, M. J. Quezada, H. Jang, S. S. Kwak, S. M. Won, K. Kwon, H. Jeong, A. J. Bandodkar, M. Han, H. Zhao, G. R. Osher, H. Wang, K. Lee, Y. Zhang, Y. Huang, J. D. Finan, J. A. Rogers, Three-dimensional, multifunctional neural interfaces for cortical spheroids and engineered assembloids. *Sci. Adv.* **73**, eabf915 (2021).

63. J. Cools, Q. Jin, E. Yoon, D. Alba Burbano, Z. Luo, D. Cuypers, G. Callewaert, D. Braeken, D. H. Gracias, A micropatterned multielectrode shell for 3D spatiotemporal recording from live cells. *Adv. Sci.* **5**, 1700731 (2018).

64. X. Yang, C. Forró, T. L. Li, Y. Miura, T. J. Zaluska, C.-T. Tsai, S. Kanton, J. P. McQueen, X. Chen, V. Mollo, F. Santoro, S. P. Pașca, B. Cui, Kirigami electronics for long-term electrophysiological recording of human neural organoids and assembloids. *Nat. Biotechnol.* **42**, 1836–1843 (2024).

65. P. Le Floch, Q. Li, Z. Lin, S. Zhao, R. Liu, K. Tasnim, H. Jiang, J. Liu, Stretchable mesh nanoelectronics for 3D single-cell chronic electrophysiology from developing brain organoids. *Adv. Mater.* **34**, e2106829 (2022).

66. B. Fan, B. Wolfrum, J. T. Robinson, Impedance scaling for gold and platinum microelectrodes. *J. Neural Eng.* **18**, 056025 (2021).

67. E. Martinelli, O. Akouissi, L. Liebi, I. Furfaro, D. Maulà, N. Savoia, A. Remy, L. Nikles, A. Roux, L. Stoppini, S. P. Lacour, The e-flower: A hydrogel-actuated 3D MEA for brain spheroid electrophysiology. *Sci. Adv.* **10**, eadp8054 (2024).

68. S. L. Giandomenico, S. B. Mierau, G. M. Gibbons, L. M. D. Wenger, L. Masullo, T. Sit, M. Sutcliffe, J. Boulanger, M. Tripodi, E. Derivery, O. Paulsen, A. Lakatos, M. A. Lancaster, Cerebral organoids at the air-liquid interface generate diverse nerve tracts with functional output. *Nat. Neurosci.* **22**, 669–679 (2019).

69. M. A. Lancaster, N. S. Corsini, S. Wolfinger, E. H. Gustafson, A. W. Phillips, T. R. Burkard, T. Otani, F. J. Livesey, J. A. Knoblich, Guided self-organization and cortical plate formation in human brain organoids. *Nat. Biotechnol.* **35**, 659–666 (2017).

70. R. A. M. Daza, C. Englund, R. F. Hevner, Organotypic slice culture of embryonic brain tissue. *CSH Protoc.* **2007**, pdb.prot4914 (2007).

71. Q. Huang, B. Tang, J. C. Romero, Y. Yang, S. K. Elsayed, G. Pahapale, T.-J. Lee, I. E. Morales Pantoja, F. Han, C. Berlinicke, T. Xiang, M. Solazzo, T. Hartung, Z. Qin, B. S.

Caffo, L. Smirnova, D. H. Gracias, Shell microelectrode arrays (MEAs) for brain organoids. *Sci. Adv.* **8**, eabq5031 (2022).

72. M. A. Lancaster, J. A. Knoblich, Generation of cerebral organoids from human pluripotent stem cells. *Nat. Protoc.* **9**, 2329–2340 (2014).

73. S. Shin, W. Lee, J. Kab Park, Wavelength selection for femtosecond laser processing of materials: Comparison of ablation efficiency and surface quality. *Opt. Laser Technol.* **171**, 110428 (2024).

74. S. Ravi-Kumar, B. Lies, X. Zhang, H. Lyu, H. Qin, Laser ablation of polymers: A review. *Polym. Int.* **68**, 1391–1401 (2019).

75. P. Augustyn, P. Rytlewski, K. Moraczewski, A. Skibicki, A. Mazurkiewicz, Ablation of selected thermoplastic polymers using an Nd:YAG laser. *J. Mater. Sci.* **58**, 9073–9086 (2023).

76. L. Rapp, S. Madden, J. Brand, K. Maximova, L. J. Walsh, H. Spallek, O. Zuaiter, A. Habeb, T. R. Hirst, A. V. Rode, Investigation of laser wavelength effect on the ablation of enamel and dentin using femtosecond laser pulses. *Sci. Rep.* **13**, 20156 (2023).

77. H. Liu, W. Lin, M. Hong, Hybrid laser precision engineering of transparent hard materials: Challenges, solutions and applications. *Light Sci Appl* **10**, 162 (2021).

78. K. Sugioka, M. Meunier, A. Piqué, Eds., "Laser precision microfabrication" in *Springer Series in Materials Science* (Springer, 2010), vol. 135.

79. P. D. Haasbroek, A. Stumpp, R. Holtz, P. M. Kristiansen, A fundamental approach to high-precision 3D microstructuring of high-performance polymers with femtosecond lasers. *Lasers Manuf. Mater. Process.* **12**, 704–735 (2025).

80. D. Flamm, M. Sailer, D. G. Grossmann, S. Rübling, D. Decker, C. Gaida, M. Blothe, D. Sutter, S. Nolte, "Ultrafast laser micromachining using wavelengths from ~0.2 µm to ~2 µm," in *Frontiers in Ultrafast Optics: Biomedical, Scientific, and Industrial Applications XXV* (SPIE, 2025), vol. 13353, pp. 37–50; https://www.spiedigitallibrary.org/conference-

proceedings-of-spie/13353/1335308/Ultrafast-laser-micromachining-using-wavelengths-from-02%c2%b5m-to-2%c2%b5m/10.1117/12.3042674.full.

81. N. Faisal, D. Zindani, K. Kumar, S. Bhowmik, “Laser micromachining of engineering materials—A review” in *Micro and Nano Machining of Engineering Materials: Recent Developments*, K. Kumar, D. Zindani, N. Kumari, J. P. Davim, Eds. (Springer International Publishing, 2019), pp. 121–136; https://doi.org/10.1007/978-3-319-99900-5_6.

82. C. S. Kim, “Thermophysical properties of stainless steels” (Tech. Rep. ANL--75-55, Argonne National Lab., 1975).

83. Engineers Edge LLC, Thermal Diffusivity Table; https://engineersedge.com/heat_transfer/thermal_diffusivity_table_13953.htm.

84. Technical Library, Specialty Coating Systems; https://scscoatings.com/technical-library.

85. J. M. Bennett, E. J. Ashley, Infrared reflectance and emittance of silver and gold evaporated in ultrahigh vacuum. *Appl. Optics* **4**, 221–224 (1965).

86. I. Vladoiu, M. Stafe, C. Negutu, I. M. Popescu, The dependence of the ablation rate of metals on nanosecond laser fluence and wavelength. *J. Optoelectron. Adv. Mater.* **10**, 3177–3181 (2008).

87. N. Stankova, P. Atanasov, R. Nikov, R. G. Nikov, N. N. Nedyalkov, T. R. Stoyanchov, N. Fukata, K. N. Kolev, E. Valova, J. S. Georgieva, S. Armyanov, Optical properties of polydimethylsiloxane (PDMS) during nanosecond laser processing. *Appl. Surf. Sci.* **374**, 96–103 (2016).
